# Co-inoculum of *Beauveria brongniartii* and *B. bassiana* shows *in vitro* different metabolic behaviour in comparison to single inoculums

**DOI:** 10.1038/s41598-017-12700-0

**Published:** 2017-10-12

**Authors:** L. Canfora, N. Abu-Samra, M. Tartanus, B. H. Łabanowska, A. Benedetti, F. Pinzari, E. Malusà

**Affiliations:** 1 Council for Agricultural Research and Economics, Research Centre for Agriculture and Environment, Via della Navicella 2-4, 00184 Rome, Italy; 2 Research Institute of Horticulture, Konstytucji 3 Maja 1, 96-100 Skierniewice, Poland; 3 Department of Life Sciences, Natural History Museum, Cromwell Road, SW7 5BD London, UK; 4 Council for Agricultural Research and Economics, Research Centre for Engineering and Agro-Food Processing, Strada delle Cacce 73, 10135 Torino, Italy; 5 Cirad - av. Agropolis - Génétique et innovation variétale (GIV), TA A-108/01 - 34398, Montpellier Cedex 5, France

## Abstract

The use of entomopathogenic fungi for biocontrol of plant pests is recently receiving an increased interest due to the need of reducing the impact of agricultural practices on the environment. Biocontrol efficacy could be improved by co-inoculation of different microorganisms. However, interactions between the fungal species can trigger or depress the biocontrol activity. Co-inoculation of two entomopathogenic fungi (*Beauveria bassiana* and *B. brongniartii)* was performed *in vitro* to evaluate the effects of their joint behaviour on a range of different carbon sources in comparison to single inoculation. The two species showed a very different metabolic profile by Phenotype MicroArray^TM^. *B. bassiana* showed a broader metabolism than *B. brongniartii* on a range of substrates. *B. brongniartii* showed a greater specificity in substrate utilization. Several carbon sources (L-Asparagine, L-Aspartic Acid, L- Glutamic Acid, m- Erythritol, D-Melezitose, D-Sorbitol) triggered the fungal metabolism in the co-inoculum. SSR markers and Real Time qPCR analysis showed that different substrates promoted either the growth of one or the other species, suggesting a form of interaction between the two fungi, related to their different ecological niches. The methodological approach that combines Phenotype MicroArray^TM^ and SSR genotyping appeared useful to assess the performance and potential competition of co-inoculated entomopathogenic fungi.

## Introduction

Entomopathogenic fungi act as natural regulators of insects’ populations and in many cases have some very species-specific actions that can be exploited against insect pests in agriculture^1^. Hundreds of commercial formulations have been developed based on few species of these fungi and are used as inundative “Biological Control Agents” (BCAs)^2^. However, the wide application of BCAs is withheld by factors such as the formulation of the product, the stabilization of the biocontrol effect under field conditions or the use of single BCA strains^3,4^.

Naturally occurring biocontrol phenomena result from complex assemblages of species rather than from a single antagonist organism^5^. To increase the biocontrol efficacy, combinations of different antagonist organisms have been utilized: fungal mixtures^6,7^ and bacterial/fungal mixtures^5,8–10^ or only bacterial mixtures^11,12^. The application of a dual inoculum composed by *Beauveria bassiana* and *Metarhizium flavoviride* to control grasshoppers populations was used, for example, to overcome some of the temperature constraints encountered in the use of a single species^13^. Co-inoculating a recombinant strain of *Metarhizium acridum* expressing a sodium channel blocker and hybrid-toxin with wild type strain increased the biocontrol efficacy against grasshopper^14^. The co-cultivations of two or more microorganisms resulted in some cases in an increased activity for some enzymes, likely dependent on the carbon source used^15–17^, which might be useful in the biocontrol activity.

Entomopathogenic fungi infect their hosts often by an initial utilization of surface layers followed usually by their entry into the host. It is generally accepted that both mechanical force and enzymatic processes and perhaps certain metabolic acids mediate the initial interaction^18^. Many entomopathogenic fungi are, in fact, dependent on the production of cuticle-degrading enzymes (lipases, chitinases, proteases) to penetrate the host insect’s cuticle^19^. Furthermore, from the point of view of inoculums production, it is noteworthy that the ingredients and types of substrates can affect fungal development^20^. By expressed sequence tag (EST) analysis of a cDNA library, Cho *et al*.^21^ found a specific relationship between *B. bassiana* transcriptome from cells produced during different environmental and developmental conditions (aerial conidia, *in vitro* blastospores and submerged conidia) and the utilization of substrate for growth and development.

However, co-inoculation of biocontrol agents can lead to either synergic or inhibitory effects between the microorganisms^5,22^. In this study co-inoculation of two entomopathogenic fungi, *B. bassiana* (Bals.-Criv.) Vuill. and *B. brongniartii* (Saccardo) Petch (De Hoog 1972), was performed *in vitro* on 95 different carbon sources using the Phenotype MicroArray^TM^ system^23,24^ to evaluate their effect on the fungi metabolic behaviour in comparison to single inoculation. To quantify the mycelium of each *Beauveria* species on some key carbon sources in the co-inoculated microplates, a genotyping approach based on the use of Single Sequence Repeat (SSR) markers was utilized^4^.

## Results

### Respiration differences between the two fungal isolates and their co-inoculum

**Figure 1:**
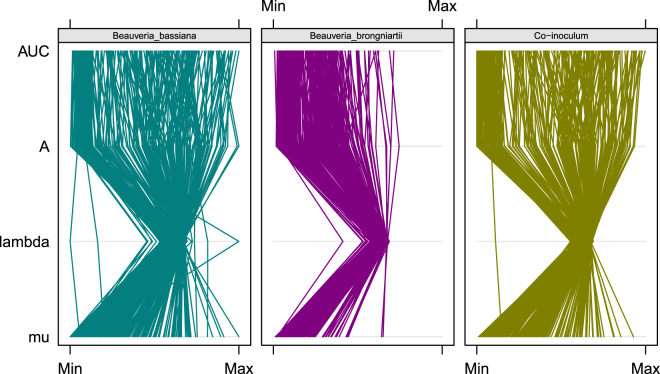
Visualisation of all four bootstrapped curve parameters for respiration data (OD 490 nm) across all substrates in one comprehensive parallel coordinate plot. The parameters are automatically scaled to a fixed range (here marked with “Min” and “Max”) and plotted by connecting lines. The descriptive curve parameters were: respiration rate (MU), lag phase (lambda), maximum curve height (A) and Area Under the Curve (AUC).

**Figure 2:**
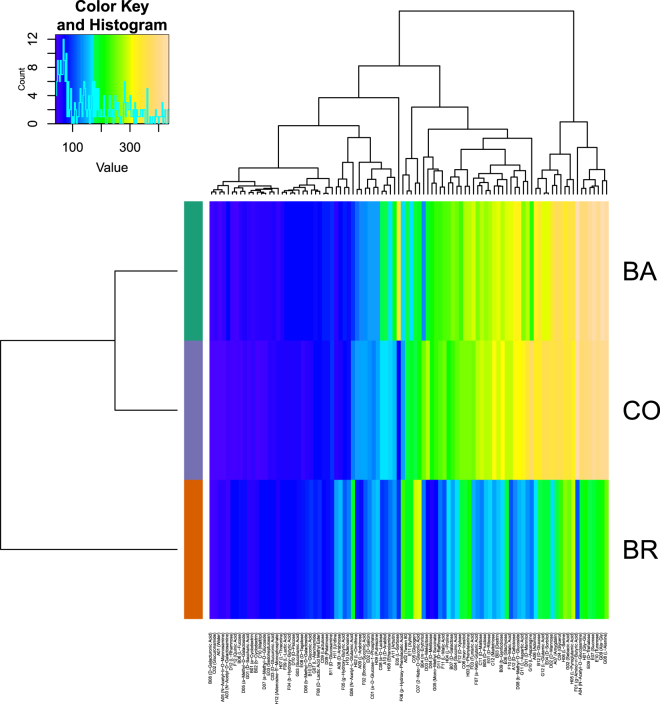
Heat-map of the clustered results from the Area Under the Curve parameter (AUC) for each substrate: respiration data (OD at 490 nm). The x-axis lists the substrates clustered above according to the Euclidean distance analysis over all the inoculums; the y-axis corresponds to the three inoculums clustered over all substrates. The central rectangle is a substrate × inoculum matrix in which the colours represent the classes of values: deep violet and blue indicate the lowest AUC values while light brown indicates the highest AUC values.

**Figure 3:**
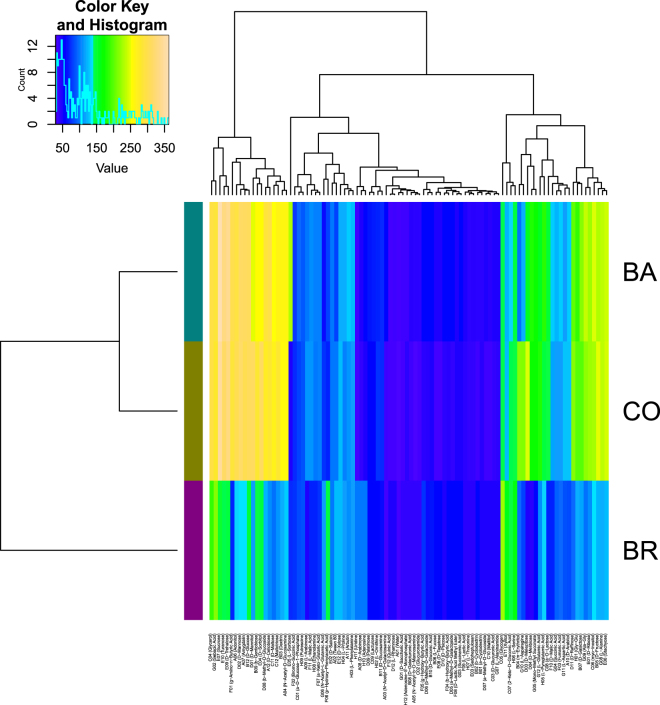
Heat-map of the clustered results from the Area Under the Curve parameter (AUC) for each substrate: growth data (OD at 750 nm). The x-axis lists the substrates clustered above according to the Euclidean distance analysis over all the inoculums; the y-axis corresponds to the three inoculums clustered over all substrates. The central rectangle is a substrate × inoculum matrix in which the colours represent the classes of values: deep violet and blue indicate the lowest AUC values while light brown indicates the highest AUC values.

The descriptive curve parameters for respiration kinetics (OD at 490 nm) measured for all the substrates differed between CO, BA and BR (Fig. 1). CO showed, in general, a different, frequently higher, metabolic response (respiration), than either BA and BR, with different substrates inducing a divergent metabolic response (mean respiration curves for each substrate and inoculum, obtained plotting mean optical density over time are reported as Supplementary Fig. S1). Clustering of the estimated aggregate area under the curve (AUC) data showed these differences across C-sources and between all three inoculums (Figs 2 and 3). CO and BA clustered together and separately from BR, underlining larger metabolic differences between CO and BR than between CO and BA. This pattern could be also broadly observed for aggregate AUC estimates across carbon sources (Fig. 1). Two main clusters of substrates resulted from the hierarchical Euclidean distance analysis (Fig. 2). Those exhibiting low AUC values grouped on the left of the graph (these comprise for example Quinic Acid, L-Rhamnose, D-Galacturonic Acid, Glucuronamide, N-Acetyl-b-D-Mannosamine, a-Cyclodextrin, b-Cyclodextrin, Adenosine-5′-Monophosphate, D-Saccharic Acid, Maltitol, etc.), and those highly metabolized by the inocula (comprising among others L-Sorbose, D-Mannose, L-Pyroglutamic Acid, Sebacic Acid, Glycerol, Amygdalin, N-Acetyl-D-Glucosamine, Turanose, D-Trehalose, L-Alanine, Sucrose, g-Amino-n-Butyric Acid) forming a separate cluster.

**Figure 4:**
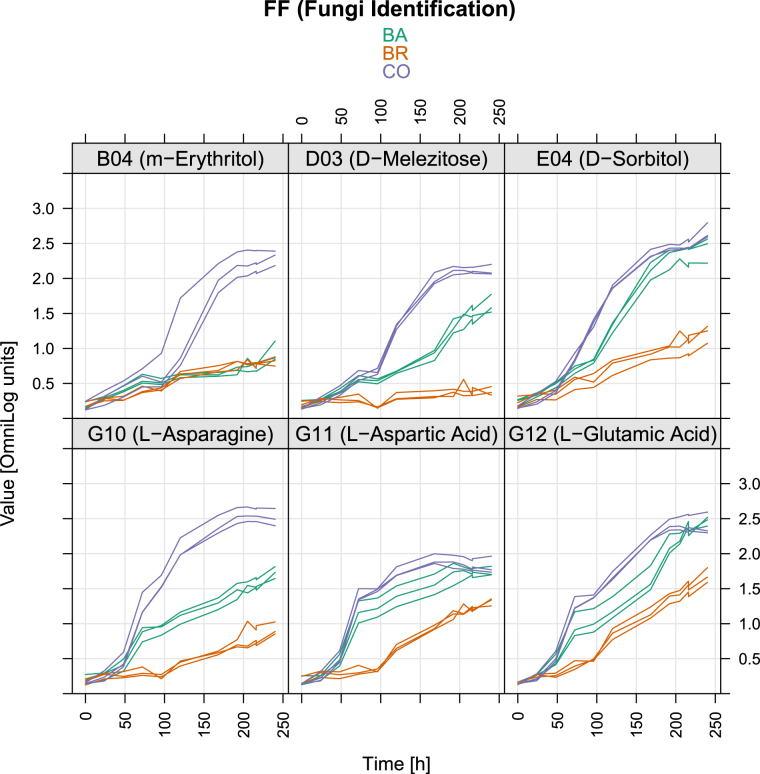
OD values of Phenotype Microarray curves of CO, BA and BR on six substrates that triggered the respiration of CO. Respiration data (OD at 490 nm). The x-axes show the measurement time in hours, the y-axes the measured colour intensities in optical density units.

The increased metabolism of CO, in comparison to each individual inoculum, was particularly evident for six C-sources: L-Asparagine, m- Erythritol, D-Melezitose, L-Aspartic acid, D-Sorbitol and L- Glutamic acid (Table 1 and Supplementary Table S1). The analysis of the respiration kinetic curves of the three inoculums indicated that the increased respiration for CO induced by L-Asparagine, m- Erythritol and D-Melezitose followed a sigmoid curve, while for BA and BR the increase was linear (Fig. 4). The latter three substrates also presented the largest estimated differences between CO and the other two strains (Table 1 and Supplementary Table S1). On the other hand, CO and BA showed a sigmoidal respiration kinetic curve on the other three substrates (L-Aspartic acid, D-Sorbitol and L- Glutamic acid), opposite to BR which showed a linear curve.

**Table 1:**
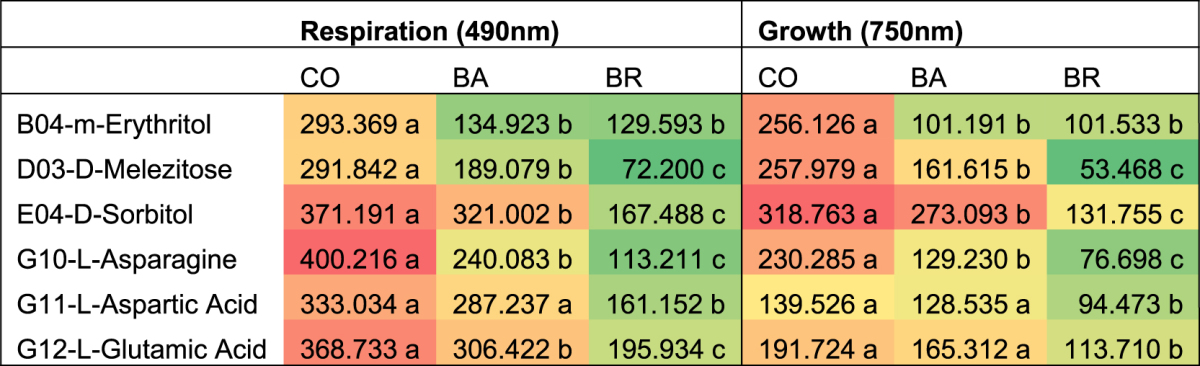
Area under the curve (AUC) of respiration and growth (means of 6 replicates). Summary of one-sided test hypothesis: CO metabolism larger than BA or BR, simultaneous tests for general linear hypotheses. See Supplementary Tables S1 and S2 for full set of statistic data. Different letters indicate significant differences between inoculums. The colour gradient is used in the table to graphically represent the degree of overall use of substrate (green = low degree, red = high degree).

Respiration of a single inoculum with respect to either CO and/or the other species was increased only by few C-sources in case of BR, unlike BA (Fig. 3, Table 2 and Supplementary Table S3). BR exhibited significantly larger respiration on 2-Keto-D-Gluconic acid than either BA or CO. BR also exhibited significantly larger respiration than BA, but not than CO, on N-Acetyl-L-Glutamic acid. On the other hand, BA presented significantly larger respiration than BR on 47 out of 96 carbon sources (49% of substrates tested), while no significant differences were found between BA and CO for the other substrates (Supplementary Table S3).

**Table 2:**
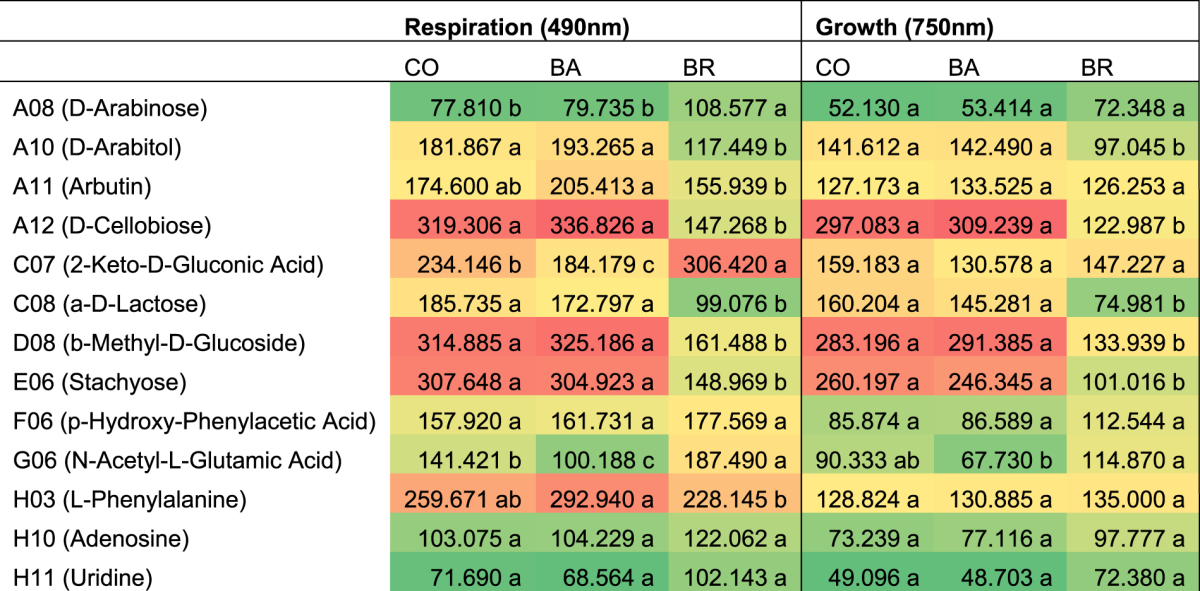
Area under the curve (AUC) for inoculum respiration and growth (means of 6 replicates). Summary of Two-sided Test Hypothesis: CO≠BA≠BR. Simultaneous Tests for General Linear Hypotheses. See Supplementary Tables S3 and S4 for full set of statistic data. Different letters indicate significant differences between inoculums. The colour gradient is used in the table to graphically represent the degree of overall use of substrate (green = low degree, red = high degree).

### Growth differences between the two fungal isolates and their co-inoculum

The mean growth curves for each substrate and inoculum, obtained plotting optical density readings at 750 over time are reported as supplementary materials (Supplementary Fig. S1). CO growth appeared more similar to that of BA than BR, reflecting clustering results obtained with AUC estimates for all substrates (Fig. 3).

**Figure 5:**
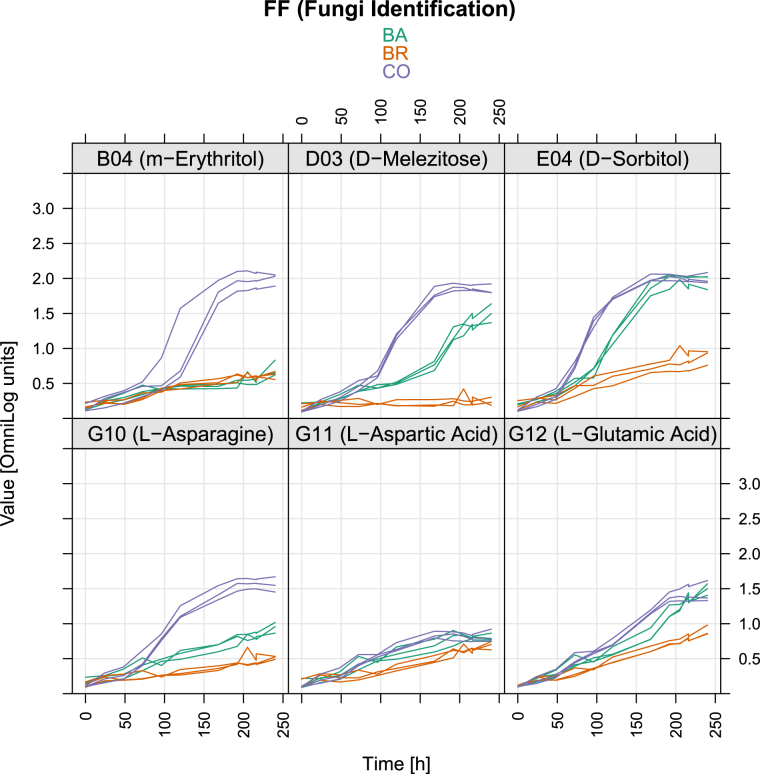
OD values of Phenotype Microarray curves of CO, BA and BR on six substrates that triggered the growth of CO. Growth data (OD at 750 nm). The x-axes show the measurement time in hours, the y-axes the measured colour intensities in optical density units.

The fungal growth kinetics, although comparable, were not fully matching respiration kinetics for several substrates (Supplementary Fig. S1a,b). Differences in coupling fungal growth and respiration for the different C-sources were confirmed by ordinal association with Kendall’s tau (Supplementary Table S5). This was particularly evident with sucrose, where correlation between growth and respiration was low in CO and BA (tau = 0.527 and 0.575 respectively) and very high in BR (tau = 0.968), or on L-Phenylalanine and N-Acetyl-D-Galactosamine where conversely the correlation was lower in BR (Supplementary Table S5). These discrepancies among inoculums and C-sources were further evidenced by the different shape of non-parametric regression curves obtained considering growth and respiration over time (Figs 4, 5 and Supplementary Fig. S2).

**Figure 6:**
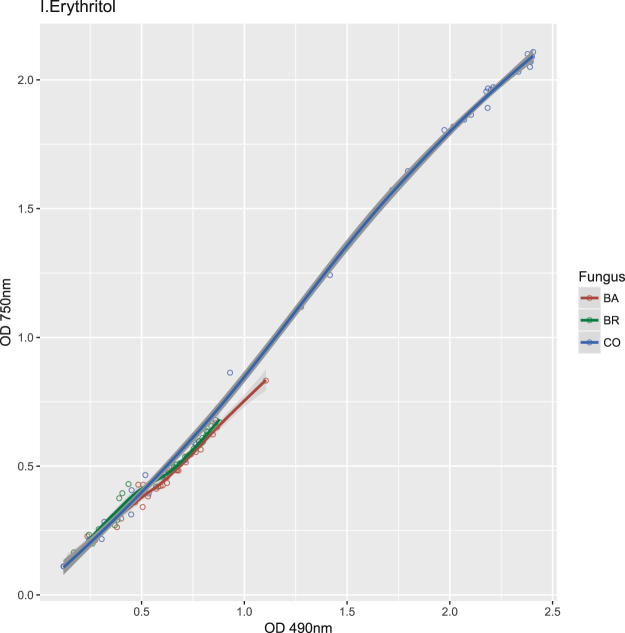
Locally weighted regression (LOESS) of growth (OD 750 nm) on respiration (OD 490 nm) values in time. The LOESS curves indicate the presence or absence of linearity between respiration-growth for BA (red), BR (green) and CO (blue). Here are shown the regression curves obtained for m-Erythritol where the CO showed both a higher growth and respiration than both BA and BR. The scatter plots obtained for the other substrates are shown in Supplementary materials (Figures in S2).

**Figure 7:**
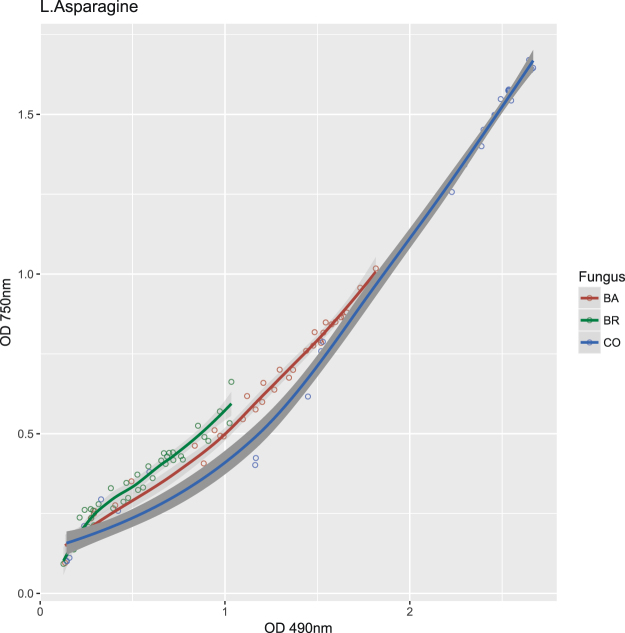
Locally weighted regression (LOESS) of growth (OD 750 nm) on respiration (OD 490 nm) values in time. The LOESS curves indicate the presence or absence of linearity between respiration-growth for BA (red), BR (green) and CO (blue). Here are shown the regression curves obtained for L-Asparagine where the CO showed both a higher growth and respiration than both BA and BR. The scatter plots obtained for the other substrates are shown in Supplementary materials (Figures in S2).

Three distinct clusters of substrates emerged from the hierarchical analysis of growth data (Fig. 3), with the central one forming a block of compounds on which the inoculums exhibited the lowest AUC values (i.e. Quinic Acid, L-Rhamnose, Adenosine-5′-Monophosphate, D-Saccharic Acid, etc.). This group comprises most of the compounds that also showed low AUC respiration values (Fig. 2). The substrates where the CO (and partly also BA) grew more were about twenty, including Sucrose, D-Trehalose, Turanose, D-Mannose, D-Sorbitol, Glycerol, D-Mannitol, Adonitol, D-Glucose, D-Maltose, Amygdalin, D-Cellobiose, g-Amino-n-Butyric Acid, Sebacic Acid, Maltotriose, b-Gentiobiose, Stachyose, N-Acetyl-D-Glucosamine, D-Melezitose, Dextrin. Some of these compounds paralleled high respiration with large growth (e.g. D-Melezitose, D-Sorbitol) (Supplementary Table S5). For the six substrates inducing a significantly higher respiration of the CO compared to the single inoculum BA and BR, only four (m-Erythritol, D-Melezitose, L-Asparagine and D-Sorbitol) were found to provoke a parallel significantly higher growth of CO, unlike Aspartic acid and Glutamic acid (Table 1, Figs 4, 5). Although for these four substrates growth curves were also sigmoidal, the absolute values of the OD was comparable only among three of them, with L-Asparagine inducing instead a lower growth. Furthermore, the regression curves obtained plotting respiration and growth values for -m- Erythritol (Fig. 6) and L-Asparagine (Fig. 7) were different in the CO inoculum with respect to BA and BR.

### Evidence of differential growth of *B. bassiana* and *B. brongniartii* on selected C-sources during co-inoculation

The measurement of the growth of each fungus when co-inoculated on some selected substrates was assessed by the combination of SSR markers and qPCR Real Time. The substrates that resulted particularly significant in stimulating or reducing the fungal respiration or growth in the CO were selected for this analysis intended to determine whether the increased activity of the co-inoculum was due to the growth of only one species or both (Table 3).

**Table 3:** Average gene copy number per gram (c.n.∙g^−1^) of *Beauveria bassiana* (BA) and *Beauveria brongniartii* (BR) in the co-inoculum (CO) grown on selected substrates (n = 4).

Carbon source	Means	
	c.n.g^−1^ of BA gene	c.n.g^−1^ BR gene
2-Keto-D-Gluconic Acid	2,753 b	628,275 a
D-Mannose	28,100 a	0,930 a
L-Asparagine	3,267 a	16,432 a
L-Aspartic Acid	3,823 a	3,269 a
L-Glutamic Acid	7,599 a	8,556 a
L-Phenylalanine	182,480 a	132,101 a
L-Pyroglutamic Acid	25,310 a	34,567 a
m- Erythritol	84,405 a	16,599 b
N-Acetyl-L-Glutamic Acid	4,448 b	134,054 a

Different letters in the row indicate statistically significant differences for p < 0.05.

A significantly different gene copy number of the two fungal species in CO biomass was determined on three substrates (m-Erythritol, N-Acetyl-L-Glutamic acid and 2-Keto-D-Gluconic acid). In particular, Erythritol stimulated significantly the growth of BA over BR, while N-Acetyl-L-Glutamic Acid and 2-Keto-D-Gluconic Acid promoted the development of BR biomass respect to BA in the co-inoculum.

## Discussion

### Differences between BA and BR metabolic behaviour

The isolates of the two *Beauveria* species showed a very different metabolic profile displaying very little overlap in carbon source use when grown *in vitro*, with the mean metabolic AUC estimates of BA significantly different to that of BR for 49 of the 96 substrates in the FF plates, 47 of which inducing a faster or/and greater respiration and growth. These included L-Aspartic acid, Arbutin, D-Arabitol, D-Cellobiose, L-Phenylalanine, Stachyose, that together indicated a low level of competition with BR. Clustering of both respiration and growth data allowed to discriminate between BA and BR, similarly to what reported after testing 130 carbon sources using Biolog SF-P2 and Biolog SF-N2 microtiter plate systems^25^.

The high versatility of BA in the different use of substrates, unlike the limited, targeted metabolism of BR, is in line with the different living behaviour of the two species. BA, in fact, is capable of living free in soil as saprophytic species and has a wide host range of nearly 750 insect species^26^. BR, on the contrary, shows a narrower host specificity, being a selective pathogen of *Melolontha melolontha* ^27^, and is scarcely capable of a saprophytic life-style^28^.

The role of some carbon sources in stimulating *Beauveria* virulence against insects was evaluated by some authors^29^. Six carbon sources (out of more than 160 different compounds) resulted to be potential virulence indicators for a *B. brongniartii* strain (Pyruvic Acid, Maltose, Glycyl-L-Glutamic Acid, Malonic Acid, Glucuronamide and Phenylethylamine)^28^. Our results showed that few substrates, other than these, enhanced growth and respiration of BR, allowing to speculate their possible role in fungal virulence. From an evolutionary standpoint the production of spores (conidia) is the most important feature for a pathogen fitness, while hyphal stages are vegetative only and usually don’t infect hosts^30^. We did not measure the number of conidia produced by the fungi when growing on the 96 substrates alone and in the co-inoculum, however the knowledge of carbon source use and preferences provides tools for understanding and control the process of spores outgrow to mycelia^31^. Considering that commercial production of biocontrol fungi is based on artificial substrates and that they can affect fungal virulence, the influence of substrate composition, particularly of carbohydrates, on biocontrol efficacy for specific strains is noteworthy^29,32,33^.

The differential use of substrates could also be associated to the different stages needed for the development of the infection in the host insect, that presumably require different metabolic abilities and the use of different carbon sources^34^. The interactions of entomopathogenic fungi, when playing the role of insect parasites, plant endophytes, rhizospheric colonizers, or simple soil inhabitants, as well as their change in trophic behaviour, require that their biosynthetic machinery undergo differential metabolic states of hyphae^35^, to fit their ecological niches^36^.

### Metabolic features of the co-inoculum

When spores of more than one species are inoculated together, competition or cooperation processes can lead to differential use of the resources^37^. In our experiment BA and BR were inoculated together on single carbon sources in a confined space (the single well). There are several possible mechanisms that may lead to the cooperation between the fungi. Species can interact during the mycelial growth phase that follows inoculation, either chemically or physically. This could lead to the partition of the resource unit into micro-territories, each territory being occupied by one species where the substrate is used independently in each territory, or the mycelia of the different species can invade the whole unit by intermingling and then either compete for the substrate, or cooperate at a metabolic level, for example producing metabolites that may be a source of carbon for the other species, otherwise unable to grow. Intermingled mycelia can transform the carbon source according to their joint enzymatic abilities with a simple additive effect (i.e. a larger or more rapid use of the carbon source), a synergic activity (i.e. use of carbon sources that single species are unable to exploit) or in a competitive effort resulting in a minor or slower use of the carbon source^38^.

The metabolic pattern of the co-inoculum (CO) showed a partial overlap with that of BA alone, thus indicating the prevalence of the latter on BR when the two strains grew together. However, significant differences were observed for some substrates: L-Asparagine, L-Aspartic Acid, m- Erythritol, D-Melezitose, D-Sorbitol and L- Glutamic Acid induced a greater metabolic activity of the co-inoculum with respect to each single inoculum. Competition theory predicts that community structure is shaped by resource partitioning between co-occurring species^39^, and that niche overlap is a key factor in community structure and species coexistence^40^. The higher performance, i.e. higher growth and metabolic activity, of the co-inoculum, with respect to each single inoculum, on some carbon sources suggests a form of interaction between the two fungal species in relation to their different ecological niches^26,28^. It could be speculated that, when forced to grow together *in vitro* (and presumably in the field when artificially co-inoculated), the two fungal species can interact and exhibit both a cooperative behaviour or a mutual repression, which could explain some inconsistent results on biocontrol of soil pests from field trials^41,42^. Co-inoculation of biocontrol microbial agents can also lead to a reciprocal inhibition^5^. The role of some carbon sources in facilitating either these behaviours could be very interesting in the perspective of new formulations of biopesticides^43^, also considering that the co-cultivation of two or more species of microorganisms apparently does not trigger a general increase in protein synthesis, but rather the induction of specific enzymes^15^, effects likely also dependent on the carbon source used and affecting the strain virulence.

### Inoculums behaviour on specific substrates

The difference in metabolic response between CO, BA and BR was found to be most pronounced with Erythritol. This compound enhanced notably the growth and respiration of the co-inoculum. It could be hypothesized that it helped the two strains to coexist in the same environment and triggered their development. However, qPCR Real Time data showed that BA mycelium dominated in the CO wells containing Erythritol. Erythritol is a sugar alcohol (or polyol) which accumulates in fungal cells under osmotic stress supporting a more rapid germination and growth at reduced water activities^44^. Conidia of *B. bassiana* are capable of storing up to 30 mg∙g^−1^ Erythritol and Trehalose, a similar polyol, whose accumulation in the fungal cells play a role in membrane and protein protection, resistance to environmental extremes, accelerates germination, enhances pathogenicity, and improves storage life of fungal propagules^45,46^. D-Trehalose is among the substrates where both the CO and BA grew more than BR and in general showed a high metabolism and growth in our study.

Among the sugars, also D-Melezitose, a non-reducing trisaccharide that is produced by many plant sap-eating insects^47^ and is itself an attractant for grub larvae^48^, stimulated growth and metabolism of CO more than BA and BR. Fungal growth and respiration on D-Melezitose showed very close kinetics, with similar shapes of the curves obtained at both 750 nm and 490 nm, resulting in very high rank correlation. However, the kinetic showed by CO was different from that of BA, the latter being characterized by a logarithmic phase less pronounced. It has been proved that this sugar can act as attractant to insect larvae in soil, and thus could have a role in the activation of virulence in entomopathogenic fungi^49–51^. *B. bassiana*, after invading its hosts via the epicuticle, infects the haemolymph and digestive tract of the host^52^, thus further suggesting a possible role of this sugar in the natural mechanism of infection.

Another polyol that triggered the co-inoculum respiration and growth was D-Sorbitol. Polyol/monosaccharide transporters are involved in plant–fungal interactions during pathogenesis^53,54^. Interestingly, substitution of glucose with sorbitol in the culturing media of *B. bassiana* decreased the yield of submerged conidia and/or increased the proportion of blastospores (hyphal bodies) in the resultant cultures^30^.

In the co-inoculum BR grew more than BA (significantly higher gene copy number in the qPCR essay) on N-Acetyl-L-Glutamic Acid and 2-Keto-D-Gluconic Acid. N-Acetyl-L-Glutamic acid is known to be metabolized by fungi^55^ and in most eukaryotic organisms the urea cycle is dependent on the presence of this compound. However, the meaning of its stimulating effect on the growth of BR in the co-inoculum (or, alternatively, the depressing effect on the development of BA) is not evident. 2-Keto-D-Gluconic Acid can be decarboxylated by fungi and produce a pentose compound^56^. The pentose pathway is of great importance for the biosynthesis of nucleic acids and amino acids. Trejo-Hernández *et al*.^57^ suggested that the activation of the pentose pathway and its upregulation during interspecific competition contributes to maintaining the metabolic activity and redox equilibrium of *Candida albicans* when growing together with bacteria and forming a biofilm.

When considering the group of substrates belonging to aminoacids, a significant difference in the metabolic response between CO, BA and BR was found with only few of them. L-Asparagine triggered significantly the growth and respiration of the co-inoculum in comparison to the single strains. Interestingly, in this case, the growth of BR prevailed on that of BA, as measured by gene copy number. L-Asparagine is a storage form of nitrogen in plants and a favourable source of organic nitrogen for fungal growth. Its release, as a consequence of *Melolontha* larvae foraging behaviour, could represent a form of attraction towards host-specific semiochemicals^58^, since the presence of asparagine was consistently determined in *Melolontha* haemolymph^59^. Other proteic compounds that triggered the metabolic activity of the co-inoculum were L-Aspartic acid and L- Glutamic acid. These amino acids, as well as others that have a role in stimulating the production of proteases, showed to play a role in the infection process of the insect host^60^.

Triggering the growth and respiration of the two fungi in the co-inoculum by several amino acids recalls the up-regulation of the asparagine, glutamine and transglutaminase activities in fungal infected arthropods, which corresponds to an enhanced humoral immune response^35^. Graham *et al*.^61^ observed that the insects feeding on carbohydrate survived a fungal infection more effectively than those fed upon protein diets, thus suggesting that the entomopathogenic fungi can more efficiently metabolise the protein contents from the insect hemocoel than the host themselves. A better performance of the two species, but particularly of *B. brongniartii*, on amino acids when co-inoculated could suggest a common activation pathway of their infective biosynthetic machinery^62^.

The evidence that, at least on some substrates, there is a stimulus to the development of the two fungi together when co-inoculated indicates the existence of some forms of interaction between the two strains of *Beauveria*. Our results, obtained *in vitro*, with controlled initial inoculum density, and in the absence of other stimuli beside the nutrient source, provided an interesting view in relation to results obtained from field trials to control *M. melolontha* larvae, where the co-inoculum was more efficient, in respect to the single strains, in reducing the number of damaged plants^63^.

The substrates inducing a higher metabolic activity in the co-inoculum with respect to single strains could be considered as promoting the coexistence of the two species. A mechanism of up-regulation of the enzymes necessary to metabolise the carbon sources induced by the fungal coexistence, linked to the ability to modify the metabolism according to the available substrate, could thus be hypothesized^64^. Another possible explanation could derive from potential interspecific hybridization of the two strains^65^.

The metabolic profile between the two *Beauveria* species singly inoculated was different from that obtained in the co-inoculated microarrays. Indeed, some carbon sources that were scarcely catabolised by the single strains, were instead metabolized effectively when there was a competition between the two species, prompting the assumption that some catabolic strategies in the fungi are expressed only when absolutely necessary, triggering the activation of “less used” metabolic pathways. Losada *et al*.^66^ performed co-cultivation competition assays among different species of *Aspergillus* showing that co-cultivation stimulated the production of novel antifungal compounds and that, in general, production of secondary metabolites by fungi is modified due to the presence of competitors.

Simple sequence repeats (SSRs) allowed the detection of very low DNA amounts. However, the number of repeats of multicopy loci can differ between strains and even within a single individual strain^67^. This variability was afforded preparing a calibration curve for each of the fungal species, thus measuring the degree of correlation between the number of spores and the copy number of SSRs^68^, which was in all cases highly significant. This procedure could not account of two other sources of variability: the presence of dikaryotic cells in the mycelia (dikaryotic hyphae, occurring after sexual reproduction, contains two nuclei, one from each parent) or the occurrence of parasexual recombination which involves heterokaryon formation and the fusion of two unlike haploid nuclei to give a diploid heterozygous nucleus. These sources of variability in the amount of DNA, and thus in the SSRs sequences in each fungal cell, makes the quantification of fungal biomass using SSRs gene copy number less strong. Further experiments should be addressed to evaluate if some carbon sources are capable of stimulating in the co-inoculum hyphal fusion more quickly than in the single inoculum.

## Conclusions

The formulation of inoculums with the combination of more than one species of biocontrol fungi implicates possible interactions, either synergic or inhibitory, between the strains/species that can affect the production phase and the biocontrol activity. The *in vitro* evaluation of the interaction between *B. bassiana* and *B. brongniartii* on a range of different carbon sources revealed that L-Asparagine, L-Aspartic Acid, m- Erythritol, L- Glutamic Acid, D-Melezitose, and D-Sorbitol triggered the metabolism and growth of the co-inoculum. The two *Beauveria* species, when tested alone, showed different behaviour in carbon source use. *B. bassiana* showed a higher metabolism than *B. brongniartii* on a wide range of substrates, paralleled by higher biomass production. The comparable metabolic and growth patterns of the co-inoculum to those of *B. bassiana* single inoculum suggests that this species would dominate in the co-inoculum. Such hypothesis was confirmed for Erythritol by means of gene copy number determination. On the other hand, few C-sources, mainly amino acids, promoted the growth of *B. brongniartii* over *B. bassiana* in the co-inoculum. These results suggest the hypothesis that the two fungi have a little niche overlap and therefore are different enough not to enter in a real competition when co-inoculated but, at the same time, at the presence of specific stimuli (i.e. competition for specific carbon sources) they can react with a higher respiration and biomass production that could possibly be accompanied by a higher virulence (yet to be verified).

## Materials and Methods

### Fungal strains

The strain of *B. brongniartii* was isolated from the soil of a potato field highly infested by *M. melolontha* in Romanów locality (Lublin voivodeship, Eastern Poland) by C. Tkaczuk and deposited in the Fungal Collection of the Department of Plant Protection and Breeding, Siedlce University of Natural Sciences and Humanities (Siedlce, Poland). Sequence has been deposited in the GenBank database and can be accessed with ID KT932309.

The strain of *B. bassiana* was selected from rhizospheric soil of an apple orchard located in Valle d’Aosta by the company CCS Aosta, (Aosta, Italy) and named BB59. Its sequence has been deposited in the GenBank database and can be accessed to ID KT932307.

### Metabolic profiling using Biolog FF microplates

The Phenotype MicroArray^TM^ system^23,69^ was used to gather information on the phenotype of *B. bassiana* (BA), *B. brongnartii* (BR) and of the co-inoculum of the two strains (CO), utilizing a method based on the FF MicroPlate^TM^ (Biolog, Inc., Hayward, California, USA), which is a commercial microarray that has 95 low-molecular weight carbon sources^23^. The inoculation procedure of pure cultures of both *Beauveria* species in the arrays was based on the original manufacturer’s supplied protocol and the protocol used by Tanzer *et al*.^70^. Briefly, conidia of the two fungal isolates were obtained by cultivation in the dark for 10 days at 25 °C on 2% malt extract agar (Oxoid Thermo Fisher Scientific Inc. Milan, Italy). Conidia were collected using a sterile cotton swab, previously moistened in Biolog FF inoculating fluid (0.25% Phytagel, 0.03% Tween 40 in distilled water) and rolled over sporulating areas of the plates. The spores were suspended in sterile Biolog FF inoculating fluid and adjusted to an optical transmission of 75% in a Biolog standard turbidimeter, calibrated with the Biolog turbidity standard for filamentous fungi in FF inoculating fluid (Code 3426 Turbidity Standard FF^TM^: 75%T, Biolog, OD 590 nm). The same suspensions were used to prepare both the single and the co-inoculum. The co-inoculum consisted in a mixture of equal volumes of the single strains spores’ suspensions (30 ml of each inoculum, at 1:1 ratio), which resulted, as well, in a final optical transmission of 75%. The initial conidial density (optical transmission of the suspension) is very important to obtain comparable results with this approach. In fact, the speed and uniformity of colour formation in each well is strongly affected by initial density of conidia; while the presence and absence of growth and colour formation is highly repeatable in each fungal species, despite the initial concentration^24^. The optical density was here used to estimate spore concentrations^71^. The cell density of spores’ suspension in FF inoculation fluid of either *B.bassiana* or *B.brongniartii* was about ~1 × 10^4^ CFU mL^−1^. They were about the same because the two fungi have conidia with approximately the same colour and dimensions. The mixture of equal volumes of each suspension showed the same cell density of about ~1 × 10^4^ CFU mL^−1^.

The FF microplates were inoculated with 100 μL of spore suspensions per well and incubated in the dark at 25 °C. Biolog plates were read immediately after inoculation, using a microplate reader (Vmax, Molecular Device), at 490 nm and 750 nm in order to zero the spectrophotometer specifically for each Biolog plate. Plates were then read at intervals of 24, 48, 72, 96, 168, 192 and 240 hours of incubation^24,69^. Three replicates were considered for each fungus and the co-inoculum. Optical density at 490 nm (OD490) was used to measure respiration. Optical density at 750 nm (OD750) was used to measure cell density.

### Quantification of fungal growth in microplate’s wells by SSR markers and qPCR Real Time

DNA was extracted from the fungal mycelium obtained from selected wells of the Biolog FF microplates after 240 h incubation of the fungal co-inoculum, using the PowerSoil DNA Isolation Kit (Mo Bio Laboratories, Carlsbad, CA) according to the manufacturer’s protocol. For each selected substrate (see Results), three distinct samples, consisting of the whole content of a well, each containing 100 microlitres of substrate with the grown fungal mycelia, were collected with a pipette from as many microplates containing the co-inoculated fungi. The concentration of DNA crude extracts, was checked by Qubit® 2.0 Fluorometer kit, following manufacturer’s instructions and stored at −20 °C before PCR analysis.

Positive controls for the quantification of BR and BA in the CO were created, for each targeted gene, using DNA from pure fungal cultures. Genomic DNA was extracted from 0.5 mg fresh mycelia of respectively *B* *. brongniartii* and *B. bassiana* as described above.

Standard curves for relating DNA concentration to fungal biomass were created quantifying DNA by qPCR Real Time from known dilutions of fungal spores’ suspensions.

The initial concentrations in the two fungal samples were: 26.5·10^5^ and 27.3·10^4^ spores ml^−1^ for BR and BA, respectively. Four sequential dilutions in sterile distilled water of the initial suspensions were then prepared.

The genetic marker used for quantifying BR with qPCR Real Time both from the microplate wells and the spores’ dilutions was the SSR marker amplified by the primers pair of the locus Bb4H9^4,26^. The marker applied to the quantification of BA was the SSR marker amplified by the primers pair of the locus Ba01^4,72^. qPCR reactions were performed in duplicate per DNA template. In order to counteract PCR inhibitory substances that might be present in fungal DNA extracts, bovine serum albumine (BSA) was added to the SYBR green mix (Qiagen). Real-time qPCR mix was then subject to the following cycling conditions: 10 min initial denaturation and 42 PCR cycles of 1 min at 95 °C, 40 s at 58 °C, followed by 30 s at 72 °C. The absence of primers dimers in amplification products, was evaluated analysing the melting curves of the products considering the fluorescence range 50–99 °C. Moreover, qPCR products were visualised on 2% agarose gel. The template quantities in FF-microplates wells was also compared with the template quantities obtained running qPCR on the spores’ suspensions. The gene copies per qPCR reaction for both fungi were calculated with respect to the fungal biomass present in each microplates’ well.

The amount of qPCR reactions products were plotted on standard curves obtained from spores’ suspensions in sterile distilled water, as described above. The gene copy number gathered for each targeted gene was calculated using the following procedure (http://www.uri.edu/research/gsc/resources/cndna.html):

$$gene\,copy\,number=(n{g}^{\ast }number/mol)/(base\,pair{s}^{\ast }ng/{g}^{\ast }g\,mol\,base\,pairs)$$

### Data analysis

Data obtained from the Phenotype MicroArray^TM^ assays were used to compare the carbon source utilisation of the two fungal species and the co-inoculum.

Analyses of 490 nm and 750 nm kinetic data was performed with the R package *opm* ^73^. Readings were normalised by the plate-specific average OD at each measurement time point, to account for varying inoculum densities, growth conditions and reading errors^74^.

Respiration (OD 490 nm) and growth (OD 750 nm) curves were subsequently modelled by cubic smoothing splines^75,76^. The descriptive curve parameters obtained with the *opm* package are respiration rate (μ), lag phase (lambda), maximum curve height (A) and Area Under the Curve (AUC)^76^. Bootstrapped (n = 10.000) estimates of the Area Under Curve (AUC) obtained for i) each substrate and plate and ii) each substrate according to inoculum type^73^ were used in the comparisons between CO, BA and BR inoculums.

The AUC provides a convenient summary of curve behaviour, accounting for changes in either lag phase, maximum rate and/or maximum curve height, together with potential secondary ‘spurs’ in respiratory or growth activity at longer incubation times, and below maximum level^77^, and was thus the parameter selected for the clustering analysis.

Fungal strains and substrates were jointly hierarchically clustered by complete linkage of aggregate AUC parameter estimates, based on Euclidean distance^24^. The results were subsequently visualised with two-dimensional heatmaps, to allow an effective identification of substrates presenting differential responses among inoculums. Differences in response were also further investigated by comparison of plate-specific (discrete) AUC bootstrap estimates and confidence intervals^73^.

We initially investigated substrates for which CO would present larger metabolic and/or growth response than either BA and BR. In so doing, we aimed to identify given carbon sources for which the co-culture of *B. bassiana* and *B. brongniartii* would result in responses larger than that of the fungal strain cultured individually. Candidate substrates were selected based on hierarchical clustering results and observed OD 490 nm plate readings. We then proceeded with the one-sided multiple comparison of CO AUC group means to the AUC group means of BA and BR; resulting in two one-sided comparisons per substrate investigated^73,78^. Family-wise error rate was accounted for by a single-step method for estimation of adjusted p-values based on the multivariate t-distribution^78^.

Similarly, we proceeded with the analysis of growth AUC (OD 750 nm) on the same selected substrates. Again, testing was one-sided and aimed at establishing whether CO was characterised by larger growth than both BA or BR. Additional two-sided multiple comparisons of AUC estimates were carried out for carbon sources for which BR, CO, BA seemed to present higher intensity of response in the aggregate OD 490 nm AUC heatmap. Results were then further compared to those obtained for fungal metabolic response, linking in so-doing growth and metabolism, to assess whether greater metabolic activity of CO also entailed stronger growth. The multiple comparisons were obtained using the *multcomp* package in R^78^ as adapted to the objects used within opm^73^.

For each inoculum (BA, BR and CO) strength of association between respiration (OD 490) and growth (OD 750 nm) was investigated by means of the non-parametric Kendall’s tau coefficient. The linearity of the association of OD 490 nm and OD 750 nm readings for BA, BR and CO was in turn assessed by locally fitted weighted regression (LOESS) of OD 750 nm values on OD 490 nm ones (i.e. growth to respiration values in time) and inspected graphically. LOESS and Kendall’s Tau allowed for better appreciation of the inoculum and substrate specific relationship between growth and metabolism.

qPCR results were analysed using one-way ANOVA (α = 0.05), and the means within each carbon sources were compared for statistical significance of the differences by using Tukey’s Honestly Significant Difference (HSD) test at a significant level for p ≤ 0.05. The data analysis was performed using XLSTAT, 2016 software (Addinsoft, Paris, France).

### Data availability statement

Most of the data generated and analysed during this study are included in this article (and its supplementary information files). The original datasets generated during the current study are available from the corresponding author on reasonable request.

## Electronic supplementary material

Supplementary Information

## References

[1] Butt, T. In *The* Mycota XI. *Agricultural applications* (ed. Kempken, F.) (Springer-Verlag Berlin Heidelberg, 2002).

[2] Faria MRde, Wraight SP (2007). Mycoinsecticides and Mycoacaricides: A comprehensive list with worldwide coverage and international classification of formulation types. Biol. Control.

[3] Berg G, Krause R, Mendes R (2015). Cross-kingdom similarities in microbiome ecology and biocontrol of pathogens. Front. Microbiol..

[4] Canfora L (2016). Development of a method for detection and quantification of B. brongniartii and B. bassiana in soil. Sci. Rep..

[5] Mishra DS, Kumar A, Prajapati CR, Singh AK, Sharma SD (2013). Identification of compatible bacterial and fungal isolate and their effectiveness against plant disease. J. Environ. Biol..

[6] Datnoff LE, Nemec S, Pernezny K (1995). Biological control of Fusarium crown and root rot of tomato in Florida using Trichoderma harzianum and Glomus intraradices. Biological Control.

[7] Núñez del Prado E, Iannacone J, Gómez H (2008). Effect of Two Entomopathogenic Fungi in Controlling Aleurodicus cocois (Curtis, 1846) (Hemiptera: Aleyrodidae). Chil. J. Agric. Res..

[8] Sicuia, O. *et al*. Pests and Diseases Management Using Compatible Biocontrol Bacteria and Entomopathogenic Fungal Strains. **XVIII** (2014).

[9] Koppenhöfer AM, Kaya HK (1997). Additive and Synergistic Interaction between Entomopathogenic Nematodes and Bacillus thuringiensis for Scarab Grub Control. Biol. Control.

[10] Hassan Dar G, Zargar M, Beigh G (1997). Biocontrol of Fusarium Root Rot in the Common Bean (Phaseolus vulgaris L.) by using Symbiotic Glomus mosseae and Rhizobium leguminosarum. Microb. Ecol..

[11] Raupach GS, Kloepper JW (1998). Mixtures of plant growth-promoting rhizobacteria enhance biological control of multiple cucumber pathogens. Phytopathology.

[12] Stockwell VO, Johnson KB, Sugar D, Loper JE (2011). Mechanistically compatible mixtures of bacterial antagonists improve biological control of fire blight of pear. Phytopathology.

[13] Inglis GD, Johnson DL, Cheng K-J, Goettel MS (1997). Use of Pathogen Combinations to Overcome the Constraints of Temperature on Entomopathogenic Hyphomycetes against Grasshoppers. Biol. Control.

[14] Fang W, Lu H-L, King GF, St. Leger RJ (2014). Construction of a hypervirulent and specific mycoinsecticide for locust control. Sci. Rep..

[15] Hu HL (2011). Improved enzyme production by co-cultivation of Aspergillus niger and Aspergillus oryzae and with other fungi. Int. Biodeterior. Biodegrad..

[16] Maheshwari DK, Gohade S, Paul J, Varma A (1994). Paper mill sludge as a potential source for cellulase production by Trichoderma reesei QM 9123 and Aspergillus niger using mixed cultivation. Carbohydr. Polym..

[17] Zhang H (2006). Efficient production of laccases by Trametes sp. AH28-2 in cocultivation with a Trichoderma strain. Appl. Microbiol. Biotechnol..

[18] Khachatourians, G. G. In (ed. Howard, D. H. & Miller, J. D.) 331–363 (Springer Berlin Heidelberg, 1996). 10.1007/978-3-662-10373-9_17.

[19] Hallsworth JE, Magan N (1994). Effect of carbohydrate type and concentration on polyhydroxy alcohol and trehalose content of conidia of three entomopathogenic fungi. Microbiology.

[20] Kamp AM, Bidochka MJ (2002). Conidium production by insect pathogenic fungi on commercially available agars. Lett. Appl. Microbiol..

[21] Cho EM, Boucias D, Keyhani NO (2006). EST analysis of cDNA libraries from the entomopathogenic fungus Beauveria (Cordyceps) bassiana. II. Fungal cells sporulating on chitin and producing oosporein. Microbiology.

[22] Thomas MB, Watson EL, Valverde-Garcia P (2003). Mixed infections and insect-pathogen interactions. Ecol. Lett..

[23] Bochner BR, Gadzinski P, Panomitros E (2001). Phenotype Microarrays for high-throughput phenotypic testing and assay of gene function. Genome Res..

[24] Pinzari F (2016). Phenotype MicroArrayTM system in the study of fungal functional diversity and catabolic versatility. Res. Microbiol..

[25] Pernfuss, B., Schweigkofler, W. & Strasser, H. Distinction of the entomopathogenic fungal species Beauveria brongniartii and Beauveria bassiana by comparing… in *IOBC/WPRS Bull*. **26**(1), 121–123 (2003).

[26] Ghikas DV (2010). Phylogenetic and biogeographic implications inferred by mitochondrial intergenic region analyses and ITS1-5.8S-ITS2 of the entomopathogenic fungi Beauveria bassiana and B. brongniartii. BMC Microbiol..

[27] Hajek AE, St. Leger RJ (1994). Interactions Between Fungal Pathogens and Insect Hosts. Annu. Rev. Entomol..

[28] Loesch A, Hutwimmer S, Strasser H (2010). Carbon utilization pattern as a potential quality control criterion for virulence of Beauveria brongniartii. J. Invertebr. Pathol..

[29] Khachatourians, G. G. & Qazi, S. S. Entomopathogenic Fungi: Biochemistry and Molecular Biology. *Hum. Anim. Relationships* (2008).

[30] Thomas KC, Khachatourians GG, Ingledew WM (1987). Production and properties of Beauveria bassiana conidia cultivated in submerged culture. Can. J. Microbiol..

[31] Liu H, Zhao X, Guo M, Liu H, Zheng Z (2015). Growth and metabolism of Beauveria bassiana spores and mycelia. BMC Microbiol..

[32] Hegedus DD, Khachatourians GG (1995). The impact of biotechnology on hyphomycetous fungal insect biocontrol agents. Biotechnology Advances.

[33] Ibrahim L, Butt TM, Jenkinson P (2002). Effect of artificial culture media on germination, growth, virulence and surface properties of the entomopathogenic hyphomycete Metarhizium anisopliae. Mycol. Res..

[34] Sánchez-pérez LDC, Barranco-florido JE, Rodríguez-navarro S, Cervantes-mayagoitia JF, Ramos-lópez MÁ (2014). Enzymes of Entomopathogenic Fungi, Advances and Insights. Adv. Enzym. ….

[35] Singh D, Son SY, Lee CH (2016). Perplexing metabolomes in fungal-insect trophic interactions: A Terra incognita of mycobiocontrol mechanisms. Front. Microbiol..

[36] St Leger RJ, Joshi L, Roberts DW (1997). Adaptation of proteases and carbohydrases of saprophytic, phytopathogenic and entomopathogenic fungi to the requirements of their ecological niches. Microbiology.

[37] Khachatourians GG (2002). Competition between unit-restricted fungi: a metapopulation model. J. Theor. Biol..

[38] Mazancourt Cde, Schwartz MW (2010). A resource ratio theory of cooperation. Ecol. Lett..

[39] Wilson M, Lindow SE (1994). Coexistence among epiphytic bacterial populations mediated through nutritional resource partitioning. Appl. Environ. Microbiol..

[40] Geange SW, Pledger S, Burns KC, Shima JS (2011). A unified analysis of niche overlap incorporating data of different types. Methods Ecol. Evol..

[41] Keller S, Schweizer C, Keller E, Brenner H (1997). Control of White Grubs (Melolontha melolontha L.) by Treating Adults with the Fungus Beauveria brongniartii. Biocontrol Sci. Technol..

[42] Jaronski, S. T. In *The Ecology of Fungal Entomopathogens* 159–185, 10.1007/978-90-481-3966-8_12 (2010).

[43] Vassilev N (2016). Potential application of glycerol in the production of plant beneficial microorganisms. J Ind Microbiol Biotechnol.

[44] Hallsworth JE, Magan N (1995). Manipulation of intracellular glycerol and erythritol enhances germination of conidia at low water availability. Microbiology.

[45] Hallsworth JE, Magan N (1996). Culture Age, temperature, and pH affect the polyol and trehalose contents of fungal propagules. Appl. Environ. Microbiol..

[46] Tarocco F, Lecuona RE, Couto AS, Arcas JA (2005). Optimization of erythritol and glycerol accumulation in conidia of Beauveria bassiana by solid-state fermentation, using response surface methodology. Appl. Microbiol. Biotechnol..

[47] Vilcinskas, A. *Biology and Ecology of Aphids, Nature*. (2016).

[48] Allsopp PG (1992). Sugars, Amino Acids, and Ascorbic Acid as Phagostimulants for Larvae of Antitrogus parvulus and Lepidiota negatoria (Coleoptera: Scarabaeidae). J. Econ. Entomol..

[49] Hsiao WF (1992). Effect of temperature and relative humidity on the virulence of the entomopathogenic fungus, Verticillium lecanii, toward the oat-bird berry aphid, Rhopalosiphum padi (Hom., Aphididae). J. Appl. Entomol..

[50] Hogervorst PAM, Wäckers FL, Romeis J (2007). Detecting nutritional state and food source use in field-collected insects that synthesize honeydew oligosaccharides. Funct. Ecol..

[51] Douglas AE (2006). Sweet problems: insect traits defining the limits to dietary sugar utilisation by the pea aphid, Acyrthosiphon pisum. J. Exp. Biol..

[52] Steinhaus, E. A. *Principles of insect pathology*. (Hafner Publishing Company, New York, 1967).

[53] Meena, M., Prasad, V., Zehra, A., Gupta, V. K. & Upadhyay, R. S. Mannitol metabolism during pathogenic fungal-host interactions under stressed conditions. *Frontiers in Microbiology* **6** (2015). 10.3389/fmicb.2015.01019 PMC4585237 26441941

[54] Bianco RL, Rieger M, Sung S (2000). Effect of drought on sorbitol and sucrose metabolism in sinks and sources of peach. Physiol. Plant..

[55] Hoare DS, Hoare SL, Brame J (1967). Deacetylation of N-acetyl-L-glutamic acid by Neurospora crassa. J. Bacteriol..

[56] Katznelson H, Tanenbaum SW, Tatum EL (1953). Glucose, gluconate and 2-Ketogluconate oxidation by Acetobacter melanogenum. J. Biol. Chem..

[57] Trejo-Hernández A, Andrade-Domínguez A, Hernández M, Encarnación S (2014). Interspecies competition triggers virulence and mutability in Candida albicans-Pseudomonas aeruginosa mixed biofilms. ISME J..

[58] de Bruyne M, Baker TC (2008). Odor detection in insects: Volatile codes. Journal of Chemical Ecology.

[59] Ussing HH (1946). Amino Acids and Related Compounds in the Haemolymph of Oryctes Nasicornis and Melolontha Vulgaris. Acta Physiol. Scand..

[60] Xu J, Baldwin D, Kindrachuk C, Hegedus DD (2006). Serine proteases and metalloproteases associated with pathogenesis but not host specificity in the Entomophthoralean fungus Zoophthora radicans. Can. J. Microbiol..

[61] Graham RI (2014). Locusts increase carbohydrate consumption to protect against a fungal biopesticide. J. Insect Physiol..

[62] Mondal S, Baksi S, Koris A, Vatai G (2016). Journey of enzymes in entomopathogenic fungi. Pacific Sci. Rev. A Nat. Sci. Eng..

[63] Tartanus, M., Łabanowska, B. H., Malusá, E., Tkaczuk, C. & Chałanska, A. Holistic approach for an effective control of white grub of European cockchafer (Melolontha melolontha) in organic strawberry plantations in Poland. in *Proceedings of XVII International Conference on Organic Fruit Growing, Hohenheim, Germany* 293–294 (2016).

[64] Xiao, G. *et al*. Genomic perspectives on the evolution of fungal entomopathogenicity in Beauveria bassiana. *Sci. Rep*. **2**, (2012). 10.1038/srep00483 PMC3387728 22761991

[65] Schardl CL, Craven KD (2003). Interspecific hybridization in plant-associated fungi and oomycetes: A review. Molecular Ecology.

[66] Losada L, Ajayi O, Frisvad JC, Yu J, Nierman WC (2009). Effect of competition on the production and activity of secondary metabolites in Aspergillus species. Med. Mycol..

[67] Li Y, Korol AB, Fahima T, Beiles A, Nevo E (2002). Microsatellites: genomic distribution, putativa functions, and mutational mechanism: a review. Mol. Ecol..

[68] Tellenbach C, Grünig CR, Sieber TN (2010). Suitability of quantitative real-time PCR to estimate the biomass of fungal root endophytes. Appl. Environ. Microbiol..

[69] Atanasova L, Druzhinina IS (2010). Review: Global nutrient profiling by Phenotype MicroArrays: a tool complementing genomic and proteomic studies in conidial fungi. J. Zhejiang Univ. Sci. B.

[70] Tanzer M (2003). Global nutritional profiling for mutant and chemical mode-of-action analysis in filamentous fungi. Funct. Integr. Genomics.

[71] Morris SC (1978). An Evaluation of Optical Density to Estimate Fungal Spore Concentrations in Water Suspensions. Phytopathology.

[72] Rehner SA, Buckley EA (2005). Beauveria phylogeny inferred from nuclear ITS and EF1-alpha sequences: evidence for cryptic diversification and links to Cordyceps teleomorphs. Mycologia.

[73] Vaas LAI (2013). opm: an R package for analysing OmniLog(R) phenotype microarray data. Bioinformatics.

[74] Garland JL, Mills AL, Young JS (2001). Relative effectiveness of kinetic analysis vs single point readings for classifying environmental samples based on community-level physiological profiles (CLPP). Soil Biol. Biochem..

[75] Wood, S. N. *Generalized additive models: an introduction with R. Chapman & Hall/CRC Texts in Statistical Science* (2017).

[76] Vaas, L. A. I., Sikorski, J., Michael, V., Göker, M. & Klenk, H. P. Visualization and curve-parameter estimation strategies for efficient exploration of phenotype microarray kinetics. *PLoS One* **7** (2012). 10.1371/journal.pone.0034846 PMC3334903 22536335

[77] Guckert JB (1996). Community analysis by Biolog: Curve integration for statistical analysis of activated sludge microbial habitats. J. Microbiol. Methods.

[78] Hothorn T, Bretz F, Westfall P (2008). Simultaneous inference in general parametric models. Biometrical Journal.

