# Supplementary material for: Co-inoculum of Beauveria brongniartii and B. bassiana shows in vitro different metabolic behaviour in comparison to single inoculums
Source: Sci Rep. 2017 Oct 12;7:13102. doi: 10.1038/s41598-017-12700-0 (PMC5638874; doi:10.1038/s41598-017-12700-0)
Supplement: Supplementary file 1 — Supplementary Information [file 41598_2017_12700_MOESM1_ESM.pdf]

Co-inoculum of Beauveria brongniartii and B. bassiana shows in vitro different metabolic behaviour in comparison to single inoculums

Canfora L.[1], Abu Samra N.[1§], Tartanus M.[2], Łabanowska B.H.[2], Benedetti A.[1], Pinzari F.[1,3*] and Malusà E. [2,4]

[1]Council for Agricultural Research and Economics, Research Centre for Agriculture and Environment, Via della Navicella 2-4, 00184 Rome, Italy

[2]Research Institute of Horticulture, Konstytucji 3 Maja 1, 96-100 Skierniewice, Poland

[3]Department of Life Sciences, Natural History Museum, Cromwell Road, SW7 5BD London, UK

[4]Council for Agricultural Research and Economics, Research Centre for Engineering and Agro-Food Processing, Strada delle Cacce 73, 10135 Torino, Italy

§ current address: Cirad - av. Agropolis - Génétique et innovation variétale (GIV). TA A-108/01 - 34398 Montpellier Cedex 5 – France

*Corresponding author

Supplementary Information:

Tables: S1, S2, S3, S4, S5

Figures: S1, S2, S3 (=a series of 96 plots)

Table S1. One-sided test Hypothesis: CO metabolism larger than BA or BR. Simultaneous Tests for General Linear Hypotheses. OD 490nm.

	**Linear**	**Hypotheses:**									
						Estimate	Std. Error	t value		Pr(>t)	
B04-Erythritol	CO	-	BA	<=	0	158.45	16.3	9.719	<	1.00E-04	***
	CO	-	BR	<=	0	163.78	16.3	10.046	<	1.00E-04	***
D03-D-Melezitose	CO	-	BA	<=	0	102.76	16.3	6.303	<	1.00E-04	***
	CO	-	BR	<=	0	219.64	16.3	13.473	<	1.00E-04	***
E04-D-Sorbitol	CO	-	BA	<=	0	50.19	16.3	3.079		0.02217	*
	CO	-	BR	<=	0	203.7	16.3	12.495	<	1.00E-04	***
G10-L-Asparagine	CO	-	BA	<=	0	160.13	16.3	9.822	<	1.00E-04	***
	CO	-	BR	<=	0	287.01	16.3	17.605	<	1.00E-04	***
G11-L-Aspartic Acid	CO	-	BA	<=	0	45.8	16.3	2.809		0.04353	*
	CO	-	BR	<=	0	171.88	16.3	10.543	<	1.00E-04	***
G12-Glutamic Acid	CO	-	BA	<=	0	62.31	16.3	3.822		0.00293	**
	CO	-	BR	<=	0	172.8	16.3	10.599	<	1.00E-04	***

Signif. codes: 0 ‘***’; 0.001 ‘**’; 0.01 ‘*’; 0.05 ‘.’; 0.1 ‘ ’; 1. (Adjusted p values reported -- single-step method)

Table S2. One-sided Test. Hypothesis: CO growth larger than that of BA or BR. Simultaneous Tests for General Linear Hypotheses. OD 750nm.

	Hypotheses:										
						Estimate	Std. Error	t value		Pr(>t)	
B04-Erythritol	CO	-	BA	<=	0	154.94	12.94	11.975	<	1.00E-04	***
	CO	-	BR	<=	0	154.59	12.94	11.949	<	1.00E-04	***
D03-D-Melezitose	CO	-	BA	<=	0	96.36	12.94	7.448	<	1.00E-04	***
	CO	-	BR	<=	0	204.51	12.94	15.807	<	1.00E-04	***
E04-D-Sorbitol	CO	-	BA	<=	0	45.67	12.94	3.53		0.00665	**
	CO	-	BR	<=	0	187.01	12.94	14.454	<	1.00E-04	***
G10-L-Asparagine	CO	-	BA	<=	0	101.06	12.94	7.811	<	1.00E-04	***
	CO	-	BR	<=	0	153.59	12.94	11.871	<	1.00E-04	***
G11-L-Aspartic Acid	CO	-	BA	<=	0	10.99	12.94	0.85		0.89011	
	CO	-	BR	<=	0	45.05	12.94	3.482		0.00758	**
G12-Glutamic Acid	CO	-	BA	<=	0	26.41	12.94	2.041		0.22892	
	CO	-	BR	<=	0	78.01	12.94	6.03	<	1.00E-04	***

Signif. codes: 0 ‘***’; 0.001 ‘**’; 0.01 ‘*’; 0.05 ‘.’; 0.1 ‘ ’; 1. (Adjusted p values reported -- single-step method)

Table S3. Two-sided Test Hypothesis: CO BA BR different. Simultaneous Tests for General Linear Hypotheses. OD 490nm. Multiple Comparisons of Means: User-defined Contrasts.

		**Hypotheses:**									
						**Estimate**	**Std. Error**	**t value**		**Pr(>t)**	
A08-D-Arabinose	BR	-	BA	=	0	28.842	16.369	1.762		0.915	
	CO	-	BA	=	0	-1.925	16.369	-0.118		1.000	
	CO	-	BR	=	0	-30.767	16.369	-1.88		0.856	
A10-D-Arabitol	BR	-	BA	=	0	-75.816	16.369	-4.632		0.001	***
	CO	-	BA	=	0	-11.398	16.369	-0.696		1.000	
	CO	-	BR	=	0	64.418	16.369	3.935		0.007	**
A11-Arbutin	BR	-	BA	=	0	-49.474	16.369	-3.022		0.112	
	CO	-	BA	=	0	-30.813	16.369	-1.882		0.855	
	CO	-	BR	=	0	18.661	16.369	1.14		1.000	
A12-D-Cellobiose	BR	-	BA	=	0	-189.558	16.369	-11.581	<	0.000	***
	CO	-	BA	=	0	-17.52	16.369	-1.07		1.000	
	CO	-	BR	=	0	172.038	16.369	10.51	<	0.000	***
C07-2-Keto-D-Gluconic Acid	BR	-	BA	=	0	122.241	16.369	7.468	<	0.000	***
	CO	-	BA	=	0	49.966	16.369	3.053		0.103	
	CO	-	BR	=	0	-72.275	16.369	-4.415		0.001	**
C08-α-D-Lactose	BR	-	BA	=	0	-73.72	16.369	-4.504		0.001	***
	CO	-	BA	=	0	12.939	16.369	0.79		1.000	
	CO	-	BR	=	0	86.659	16.369	5.294	<	0.000	***
D08-β-Methyl-D-Glucoside	BR	-	BA	=	0	-163.698	16.369	-10.001	<	0.000	***
	CO	-	BA	=	0	-10.301	16.369	-0.629		1.000	
	CO	-	BR	=	0	153.397	16.369	9.371	<	0.000	***
E06-Stachynose	BR	-	BA	=	0	-155.953	16.369	-9.528	<	0.000	***
	CO	-	BA	=	0	2.725	16.369	0.166		1.000	
	CO	-	BR	=	0	158.679	16.369	9.694	<	0.000	***
F06-p-Hydroxyphenyl-acetic acid	BR	-	BA	=	0	15.838	16.369	0.968		1.000	
	CO	-	BA	=	0	-3.81	16.369	-0.233		1.000	
	CO	-	BR	=	0	-19.648	16.369	-1.2		0.999	
G06-N-Acetyly-L-Glutamic Acid	BR	-	BA	=	0	87.302	16.369	5.333	<	0.000	***
	CO	-	BA	=	0	41.233	16.369	2.519		0.364	
	CO	-	BR	=	0	-46.069	16.369	-2.814		0.190	
H03-L-Phenylalanine	BR	-	BA	=	0	-64.796	16.369	-3.959		0.006	**
	CO	-	BA	=	0	-33.269	16.369	-2.032		0.754	
	CO	-	BR	=	0	31.526	16.369	1.926		0.828	
H10-Adenosine	BR	-	BA	=	0	17.833	16.369	1.089		1.000	
	CO	-	BA	=	0	-1.154	16.369	-0.071		1.000	
	CO	-	BR	=	0	-18.988	16.369	-1.16		1.000	
H11-Uridine	BR	-	BA	=	0	33.579	16.369	2.051		0.739	
	CO	-	BA	=	0	3.126	16.369	0.191		1.000	
	CO	-	BR	=	0	-30.453	16.369	-1.86		0.867	
A08-D-Arabinose	BR	-	BA	=	0	28.842	16.369	1.762		0.915	

CO	-	BA	=	0	-1.925	16.369	-0.118	1.000
CO	-	BR	=	0	-30.767	16.369	-1.88	0.856

Signif. codes: 0 ‘***’; 0.001 ‘**’; 0.01 ‘*’; 0.05 ‘.’; 0.1 ‘ ’; 1. (Adjusted p values reported -- single-step method)

Table S4. Two-sided Test Hypothesis: CO BA BR different. Simultaneous Tests for General Linear Hypotheses. OD 750nm. Multiple Comparisons of Means: User-defined Contrasts.

	**Hypotheses:**										
						**Estimate**	**Std. Error**	**t value**		**Pr(>t)**	
A08-D-Arabinose	BR	-	BA	=	0	18.935	13.186	1.436		0.991	
	CO	-	BA	=	0	-1.284	13.186	-0.097		1.000	
	CO	-	BR	=	0	-20.218	13.186	-1.533		0.979	
A10-D-Arabitol	BR	-	BA	=	0	-45.445	13.186	-3.446		0.033	*
	CO	-	BA	=	0	-0.878	13.186	-0.067		1.000	
	CO	-	BR	=	0	44.567	13.186	3.380		0.040	*
A11-Arbutin	BR	-	BA	=	0	-7.273	13.186	-0.552		1.000	
	CO	-	BA	=	0	-6.353	13.186	-0.482		1.000	
	CO	-	BR	=	0	0.920	13.186	0.070		1.000	
A12-D-Cellobiose	BR	-	BA	=	0	-186.252	13.186	-14.125	<	0.000	***
	CO	-	BA	=	0	-12.156	13.186	-0.922		1.000	
	CO	-	BR	=	0	174.097	13.186	13.203	<	0.000	***
C07-2-Keto-D-Gluconic Acid	BR	-	BA	=	0	16.649	13.186	1.263		0.998	
	CO	-	BA	=	0	28.605	13.186	2.169		0.645	
	CO	-	BR	=	0	11.956	13.186	0.907		1.000	
C08-α-D-Lactose	BR	-	BA	=	0	-70.300	13.186	-5.331	<	0.000	***
	CO	-	BA	=	0	14.923	13.186	1.132		1.000	
	CO	-	BR	=	0	85.223	13.186	6.463	<	0.000	***
D08-β-Methyl-D-Glucoside	BR	-	BA	=	0	-157.446	13.186	-11.940	<	0.000	***
	CO	-	BA	=	0	-8.189	13.186	-0.621		1.000	
	CO	-	BR	=	0	149.257	13.186	11.319	<	0.000	***
E06-Stachynose	BR	-	BA	=	0	-145.328	13.186	-11.021	<	0.000	***
	CO	-	BA	=	0	13.852	13.186	1.051		1.000	
	CO	-	BR	=	0	159.181	13.186	12.072	<	0.000	***
F06-p-Hydroxyphenyl-acetic acid	BR	-	BA	=	0	25.955	13.186	1.968		0.800	
	CO	-	BA	=	0	-0.715	13.186	-0.054		1.000	
	CO	-	BR	=	0	-26.670	13.186	-2.023		0.761	
G06-N-Acetyly-L-Glutamic Acid	BR	-	BA	=	0	47.140	13.186	3.575		0.022	*
	CO	-	BA	=	0	22.603	13.186	1.714		0.934	
	CO	-	BR	=	0	-24.537	13.186	-1.861		0.867	
H03-L-Phenylalanine	BR	-	BA	=	0	4.115	13.186	0.312		1.000	
	CO	-	BA	=	0	-2.061	13.186	-0.156		1.000	
	CO	-	BR	=	0	-6.176	13.186	-0.468		1.000	
H10-Adenosine	BR	-	BA	=	0	20.661	13.186	1.567		0.973	
	CO	-	BA	=	0	-3.877	13.186	-0.294		1.000	
	CO	-	BR	=	0	-24.538	13.186	-1.861		0.867	
H11-Uridine	BR	-	BA	=	0	23.678	13.186	1.796		0.900	
	CO	-	BA	=	0	0.393	13.186	0.030		1.000	
	CO	-	BR	=	0	-23.285	13.186	-1.766		0.914	
A08-D-Arabinose	BR	-	BA	=	0	18.935	13.186	1.436		0.991	

CO	-	BA	=	0	-1.284	13.186	-0.097	1.000
CO	-	BR	=	0	-20.218	13.186	-1.533	0.979

Signif. codes: 0 ‘***’; 0.001 ‘**’; 0.01 ‘*’; 0.05 ‘.’; 0.1 ‘ ’; 1. (Adjusted p values reported -- single-step method)

Table S5. Kendall's tau coefficient used to measure the ordinal association between respiration (OD 490) and growth (OD 750 nm) data. The colour gradient is used in the table to graphically represent the degree of correlation between growth and respiration (green=low degree, red=high degree).

Substrate	tau CO	2-sided p-value	tau BA	2-sided p-value	tau BR	2-sided p-value
A.Cyclodextrin	0.695	<2.22e-16	0.788	<2.22e-16	0.639	<2.22e-16
a.D.Glucose	0.886	<2.22e-16	0.949	<2.22e-16	0.968	<2.22e-16
a.D.Lactose	0.98	<2.22e-16	0.976	<2.22e-16	0.896	<2.22e-16
A.Keto.glutaricAcid	0.868	<2.22e-16	0.846	<2.22e-16	0.912	<2.22e-16
a.Methyl.D.Galactoside	0.769	<2.22e-16	0.826	<2.22e-16	0.575	3.58E-07
a.Methyl.D.Glucoside	0.729	<2.22e-16	0.743	<2.22e-16	0.537	1.67E-06
Adenosine	0.681	<2.22e-16	0.741	<2.22e-16	0.293	0.0089771
Adenosine.5.Monophosphate	0.783	<2.22e-16	0.756	<2.22e-16	0.692	<2.22e-16
Adonitol	0.894	<2.22e-16	0.959	<2.22e-16	0.895	<2.22e-16
Alaninamide	0.729	<2.22e-16	0.751	<2.22e-16	0.64	<2.22e-16
Amygdalin	0.816	<2.22e-16	0.87	<2.22e-16	0.957	<2.22e-16
Arbutin	0.795	<2.22e-16	0.861	<2.22e-16	0.978	<2.22e-16
b.Cyclodextrin	0.756	<2.22e-16	0.786	<2.22e-16	0.61	<2.22e-16
B.Hydroxy.butyricAcid	0.825	<2.22e-16	0.792	<2.22e-16	0.765	<2.22e-16
b.Methyl.D.Galactoside	0.828	<2.22e-16	0.828	<2.22e-16	0.861	<2.22e-16
b.Methyl.D.Glucoside	0.884	<2.22e-16	0.93	<2.22e-16	0.978	<2.22e-16
BromosuccinicAcid	0.822	<2.22e-16	0.772	<2.22e-16	0.885	<2.22e-16
D.Arabinose	0.773	<2.22e-16	0.797	<2.22e-16	0.872	<2.22e-16
D.Arabitol	0.975	<2.22e-16	0.972	<2.22e-16	0.946	<2.22e-16
D.Cellobiose	0.874	<2.22e-16	0.87	<2.22e-16	0.968	<2.22e-16
D.Fructose	0.962	<2.22e-16	0.97	<2.22e-16	0.949	<2.22e-16
D.Galactose	0.967	<2.22e-16	0.975	<2.22e-16	0.982	<2.22e-16
D.GalacturonicAcid	0.835	<2.22e-16	0.834	<2.22e-16	0.83	<2.22e-16
D.GluconicAcid	0.837	<2.22e-16	0.862	<2.22e-16	0.866	<2.22e-16
D.Glucosamine	0.933	<2.22e-16	0.934	<2.22e-16	0.894	<2.22e-16
D.GlucuronicAcid	0.802	<2.22e-16	0.746	<2.22e-16	0.606	1.19E-07
D.LacticAcidMethylEster	0.662	<2.22e-16	0.685	<2.22e-16	0.618	<2.22e-16
D.MalicAcid	0.959	<2.22e-16	0.914	<2.22e-16	0.854	<2.22e-16
D.Mannitol	0.866	<2.22e-16	0.866	<2.22e-16	0.916	<2.22e-16
D.MannitolBA	0.916	<2.22e-16	0.973	<2.22e-16	0.973	<2.22e-16
D.Mannose	0.791	<2.22e-16	0.897	<2.22e-16	0.981	<2.22e-16
D.Melezitose	0.957	<2.22e-16	0.954	<2.22e-16	0.507	5.96E-06
D.Melibiose	0.97	<2.22e-16	0.98	<2.22e-16	0.583	2.38E-07
D.Psicose	0.816	<2.22e-16	0.816	<2.22e-16	0.723	<2.22e-16
D.Raffinose	0.727	<2.22e-16	0.894	<2.22e-16	0.968	<2.22e-16
D.Ribose	0.951	<2.22e-16	0.964	<2.22e-16	0.948	<2.22e-16
D.SaccharinAcid	0.616	<2.22e-16	0.636	<2.22e-16	0.471	2.68E-05
D.Sorbitol	0.808	<2.22e-16	0.889	<2.22e-16	0.922	<2.22e-16
D.Tagatose	0.709	<2.22e-16	0.691	<2.22e-16	0.623	<2.22e-16
D.Trehalose	0.826	<2.22e-16	0.914	<2.22e-16	0.976	<2.22e-16
D.Xylose	0.947	<2.22e-16	0.949	<2.22e-16	0.943	<2.22e-16

Dextrin	0.913	<2.22e-16	0.928	<2.22e-16	0.941	<2.22e-16
FumaricAcid	0.972	<2.22e-16	0.868	<2.22e-16	0.887	<2.22e-16
G.Amino.butyricAcid	0.811	<2.22e-16	0.826	<2.22e-16	0.897	<2.22e-16
G.Hydroxy.butyricAcid	0.773	<2.22e-16	0.773	<2.22e-16	0.654	<2.22e-16
Gentibiose	0.727	<2.22e-16	0.88	<2.22e-16	0.989	<2.22e-16
Glucose.1.Phophate	0.951	<2.22e-16	0.959	<2.22e-16	0.897	<2.22e-16
Glucuronamide	0.684	<2.22e-16	0.687	<2.22e-16	0.67	<2.22e-16
Glycerol	0.836	<2.22e-16	0.803	<2.22e-16	0.964	<2.22e-16
Glycogen	0.96	<2.22e-16	0.976	<2.22e-16	0.943	<2.22e-16
Glycyl.L.GlutamicAcid	0.892	<2.22e-16	0.93	<2.22e-16	0.927	<2.22e-16
I.Erythritol	0.968	<2.22e-16	0.921	<2.22e-16	0.943	<2.22e-16
L.Alanine	0.895	<2.22e-16	0.9	<2.22e-16	0.943	<2.22e-16
L.Alanyl.Glycine	0.957	<2.22e-16	0.959	<2.22e-16	0.935	<2.22e-16
L.Arabinose	0.984	<2.22e-16	0.981	<2.22e-16	0.911	<2.22e-16
L.Asparagine	0.938	<2.22e-16	0.943	<2.22e-16	0.875	<2.22e-16
L.AsparticAcid	0.899	<2.22e-16	0.938	<2.22e-16	0.924	<2.22e-16
L.Fucose	0.749	<2.22e-16	0.673	<2.22e-16	0.841	<2.22e-16
L.GlutamicAcid	0.96	<2.22e-16	0.959	<2.22e-16	0.953	<2.22e-16
L.LacticAcid	0.735	<2.22e-16	0.735	<2.22e-16	0.674	<2.22e-16
L.LacticAcidBA	0.674	<2.22e-16	0.613	<2.22e-16	0.613	<2.22e-16
L.MalicAcid	0.9	<2.22e-16	0.868	<2.22e-16	0.903	<2.22e-16
L.Ornithine	0.924	<2.22e-16	0.783	<2.22e-16	0.791	<2.22e-16
L.Phenylalanine	0.787	<2.22e-16	0.856	<2.22e-16	0.538	1.55E-06
L.Proline	0.935	<2.22e-16	0.907	<2.22e-16	0.926	<2.22e-16
L.PyroglutamicAcid	0.897	<2.22e-16	0.842	<2.22e-16	0.932	<2.22e-16
L.Rhamnose	0.738	<2.22e-16	0.754	<2.22e-16	0.538	1.55E-06
L.Serine	0.9	<2.22e-16	0.903	<2.22e-16	0.903	<2.22e-16
L.Sorbose	0.8	<2.22e-16	0.937	<2.22e-16	0.691	<2.22e-16
L.Threonine	0.747	<2.22e-16	0.727	<2.22e-16	0.689	<2.22e-16
Lactulose	0.914	<2.22e-16	0.9	<2.22e-16	0.808	<2.22e-16
m.Inositol	0.927	<2.22e-16	0.935	<2.22e-16	0.935	<2.22e-16
Maltilol	0.809	<2.22e-16	0.793	<2.22e-16	0.716	<2.22e-16
Maltose	0.906	<2.22e-16	0.929	<2.22e-16	0.97	<2.22e-16
Maltotriose	0.867	<2.22e-16	0.875	<2.22e-16	0.974	<2.22e-16
N.Acetyl.D.Galactosamine	0.818	<2.22e-16	0.791	<2.22e-16	0.452	5.58E-05
N.Acetyl.D.Glucosamine	0.967	<2.22e-16	0.967	<2.22e-16	0.972	<2.22e-16
N.Acetyl.D.Mannosamine	0.793	<2.22e-16	0.743	<2.22e-16	0.5	8.46E-06
N.Acetyly.L.GlutamicAcid	0.914	<2.22e-16	0.827	<2.22e-16	0.957	<2.22e-16
P.Hydroxyphenyl.aceticAcid	0.91	<2.22e-16	0.781	<2.22e-16	0.904	<2.22e-16
Palatinose	0.88	<2.22e-16	0.878	<2.22e-16	0.727	<2.22e-16
Putrescine	0.943	<2.22e-16	0.903	<2.22e-16	0.903	<2.22e-16
QuinicAcid	0.773	<2.22e-16	0.71	<2.22e-16	0.511	4.77E-06
Salicin	0.678	<2.22e-16	0.777	<2.22e-16	0.862	<2.22e-16
SebacicAcid	0.67	<2.22e-16	0.723	<2.22e-16	0.922	<2.22e-16
Sedoheptulosan	0.748	<2.22e-16	0.74	<2.22e-16	0.544	1.19E-06
Stachynose	0.891	<2.22e-16	0.87	<2.22e-16	0.883	<2.22e-16
SuccinamicAcid	0.774	<2.22e-16	0.78	<2.22e-16	0.811	<2.22e-16

SuccinicAcid	0.792	<2.22e-16	0.819	<2.22e-16	0.935	<2.22e-16
SuccinicAcidMono.MethylEster	0.838	<2.22e-16	0.636	<2.22e-16	0.738	<2.22e-16
Sucrose	0.527	2.50E-06	0.575	2.38E-07	0.968	<2.22e-16
Turanose	0.86	<2.22e-16	0.895	<2.22e-16	0.921	<2.22e-16
Tween80	0.897	<2.22e-16	0.776	<2.22e-16	0.887	<2.22e-16
Uridine	0.725	<2.22e-16	0.619	<2.22e-16	0.78	<2.22e-16
Water	0.728	<2.22e-16	0.783	<2.22e-16	0.543	1.31E-06
2.AminoEthanol	0.953	<2.22e-16	0.951	<2.22e-16	0.813	<2.22e-16
2.Keto.D.GluconicAcid	0.946	<2.22e-16	0.962	<2.22e-16	0.876	<2.22e-16
Xylitol	0.97	<2.22e-16	0.943	<2.22e-16	0.969	<2.22e-16

Figure S1a. (supplementary material) Average values (n=3) of optical density units (OD) at 490nm (respiration). Phenotype Microarray averaged curves of CO, BA and BR on all the 95 substrates + water. The x-axis shows the measurement time in hours, the y-axis the measured colour intensities in optical density units (OD).

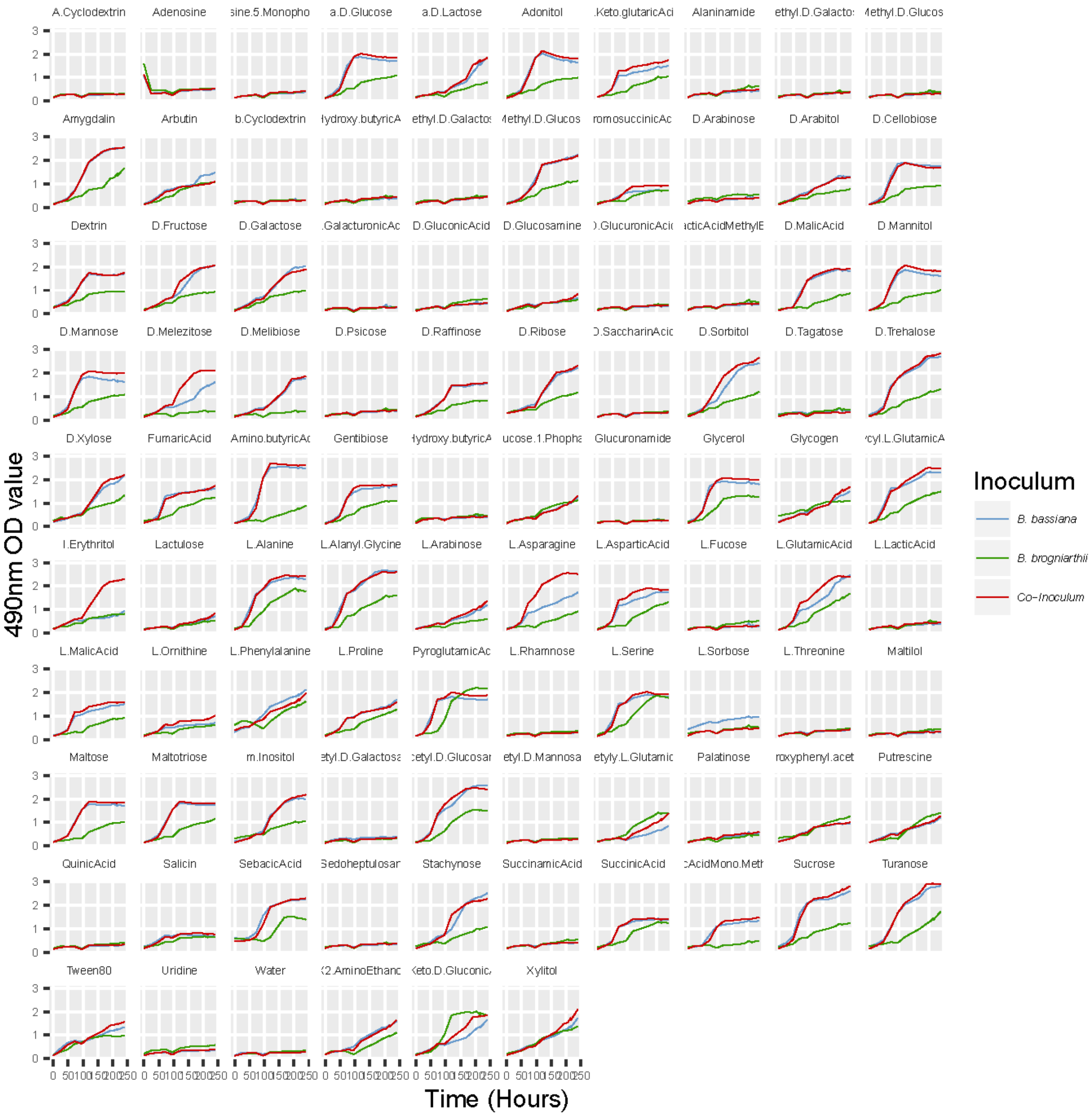

Figure S1b. (supplementary material) Average values (n=3) of optical density units (OD) at 750nm (growth/biomass). Phenotype Microarray averaged curves of CO, BA and BR on all the 95 substrates + water. The x-axis shows the measurement time in hours, the y-axis the measured colour intensities in optical density units (OD).

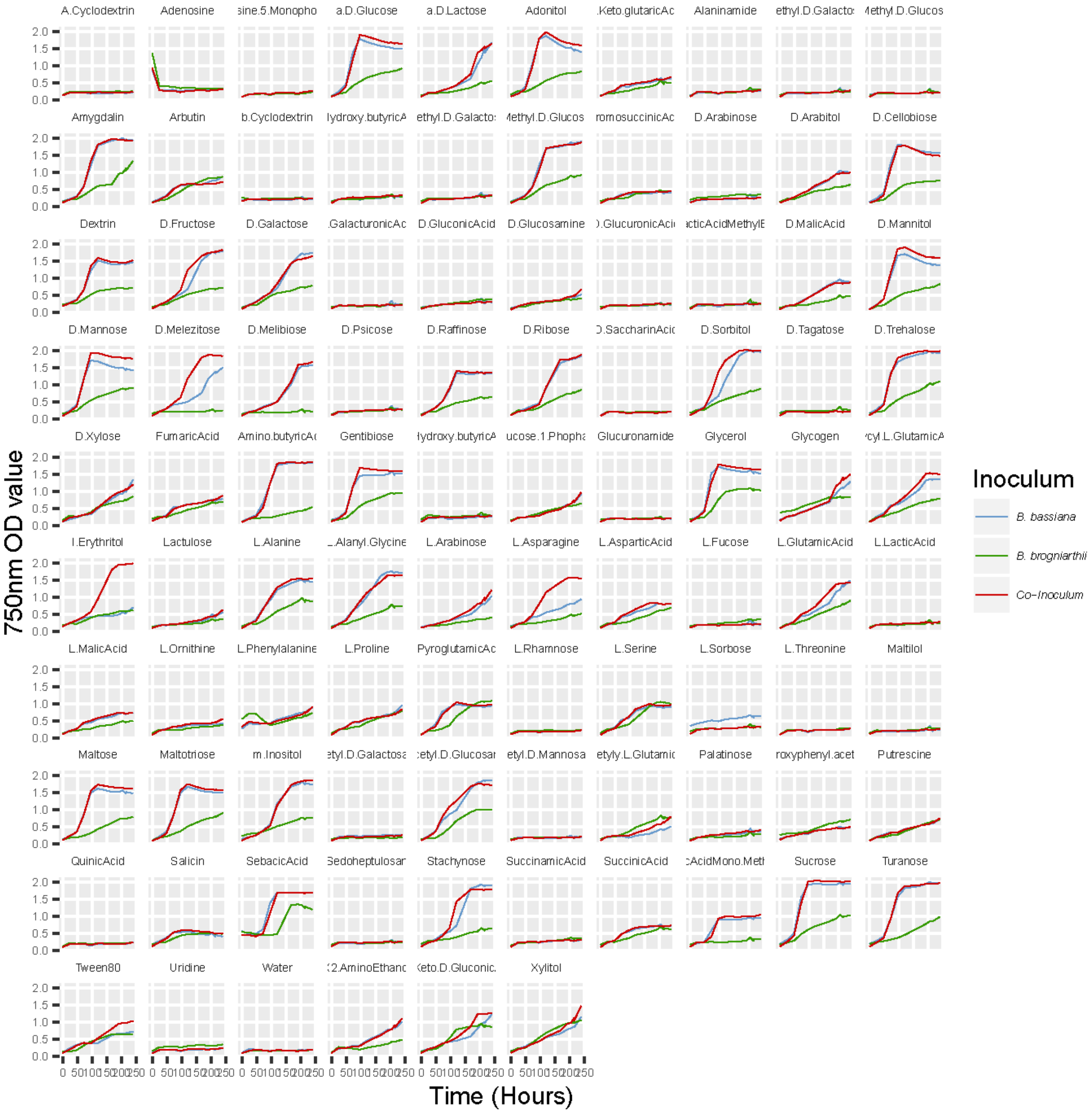

Figures S2 (1 to 96) LOESS (locally weighted scatterplot smoothing) non-parametric regression curves obtained plotting respiration to growth values in time, from all the 96 substrates by the three inoculums.

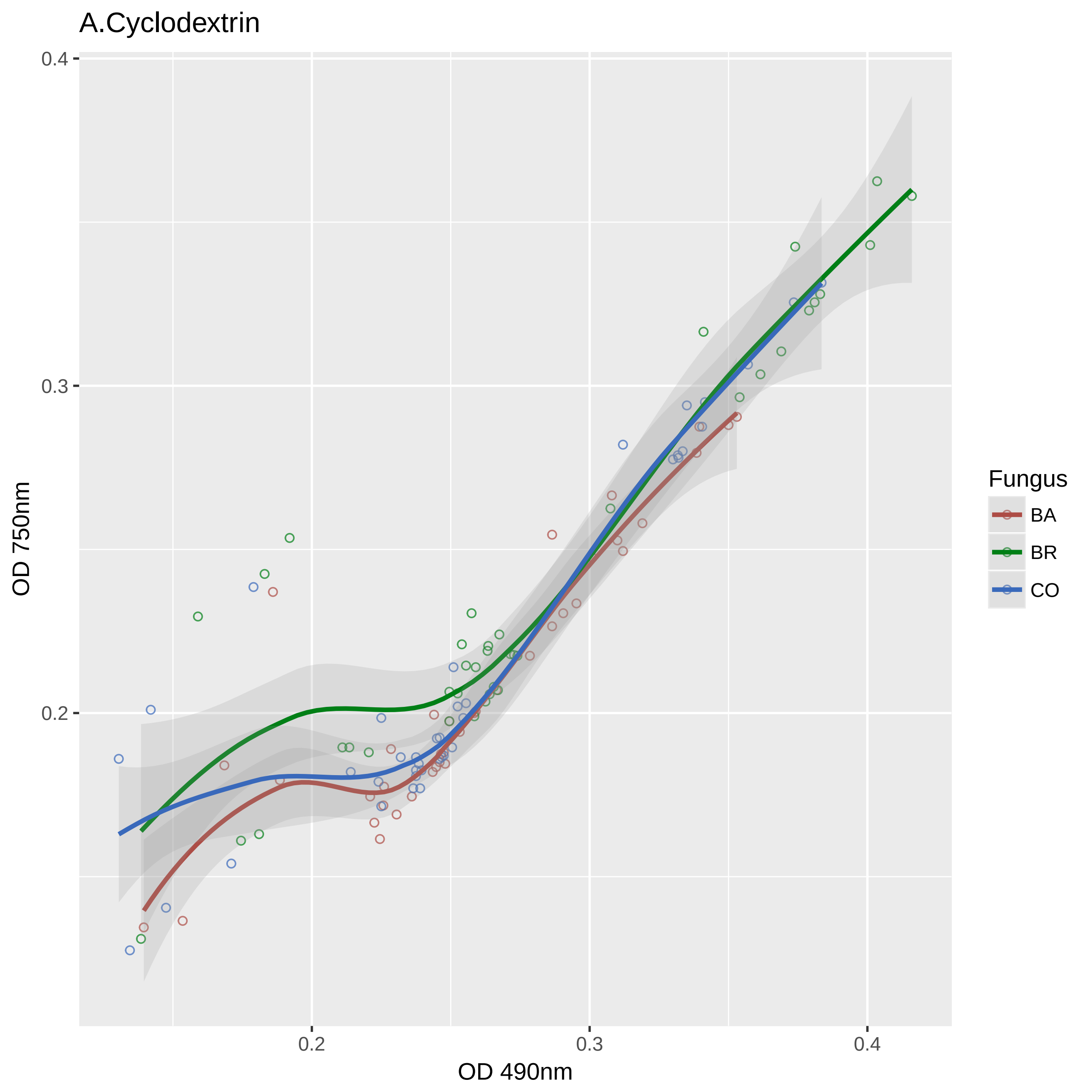

A.Cyclodextrin
0.4
0.3
0.2
0.2
0.3
0.4
OD 750nm
OD 490nm
Fungus
BA
BR
CO

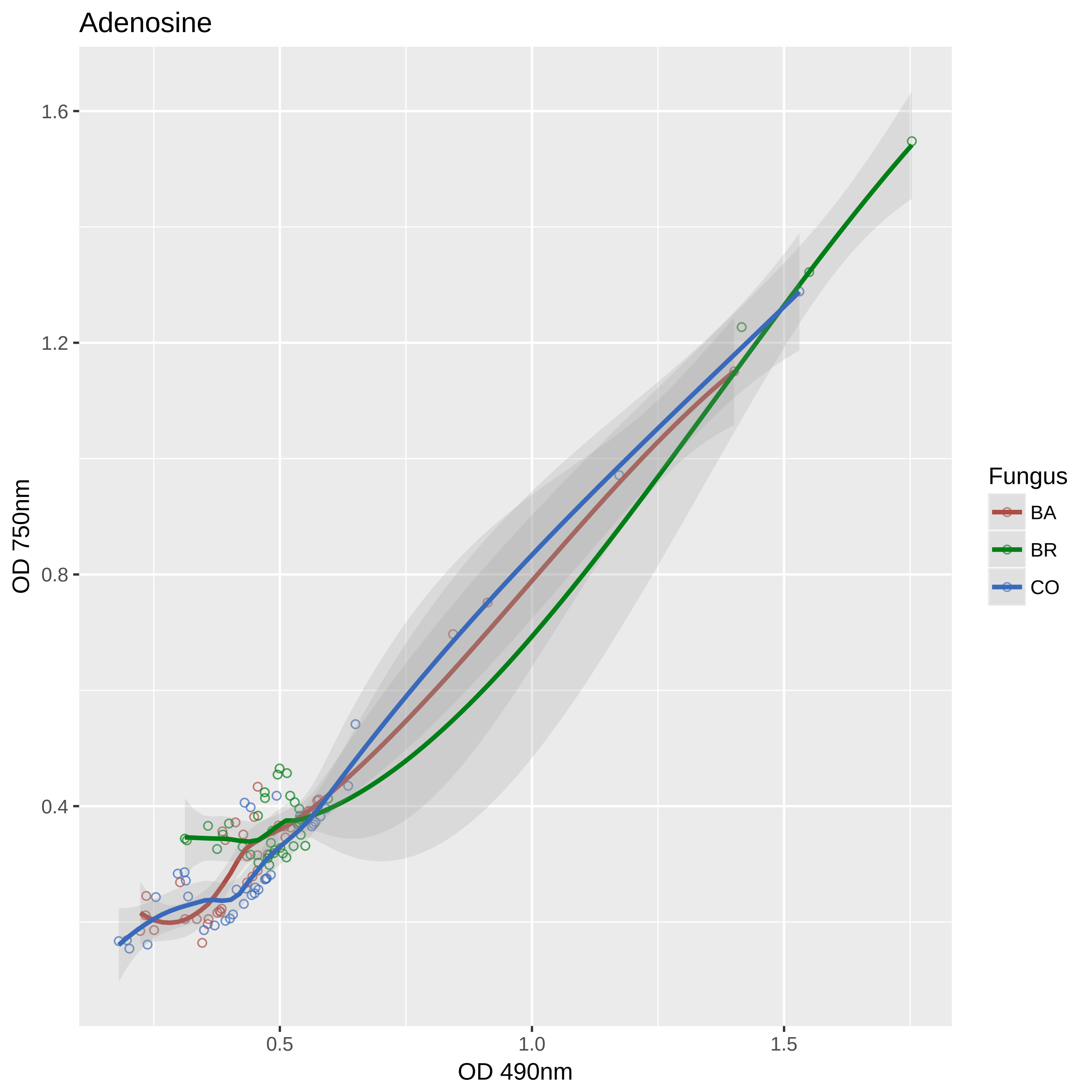
Adenosine
1.6
1.2
0.8
0.4
OD 750nm
0.5
1.0
1.5
OD 490nm
Fungus
BA
BR
CO

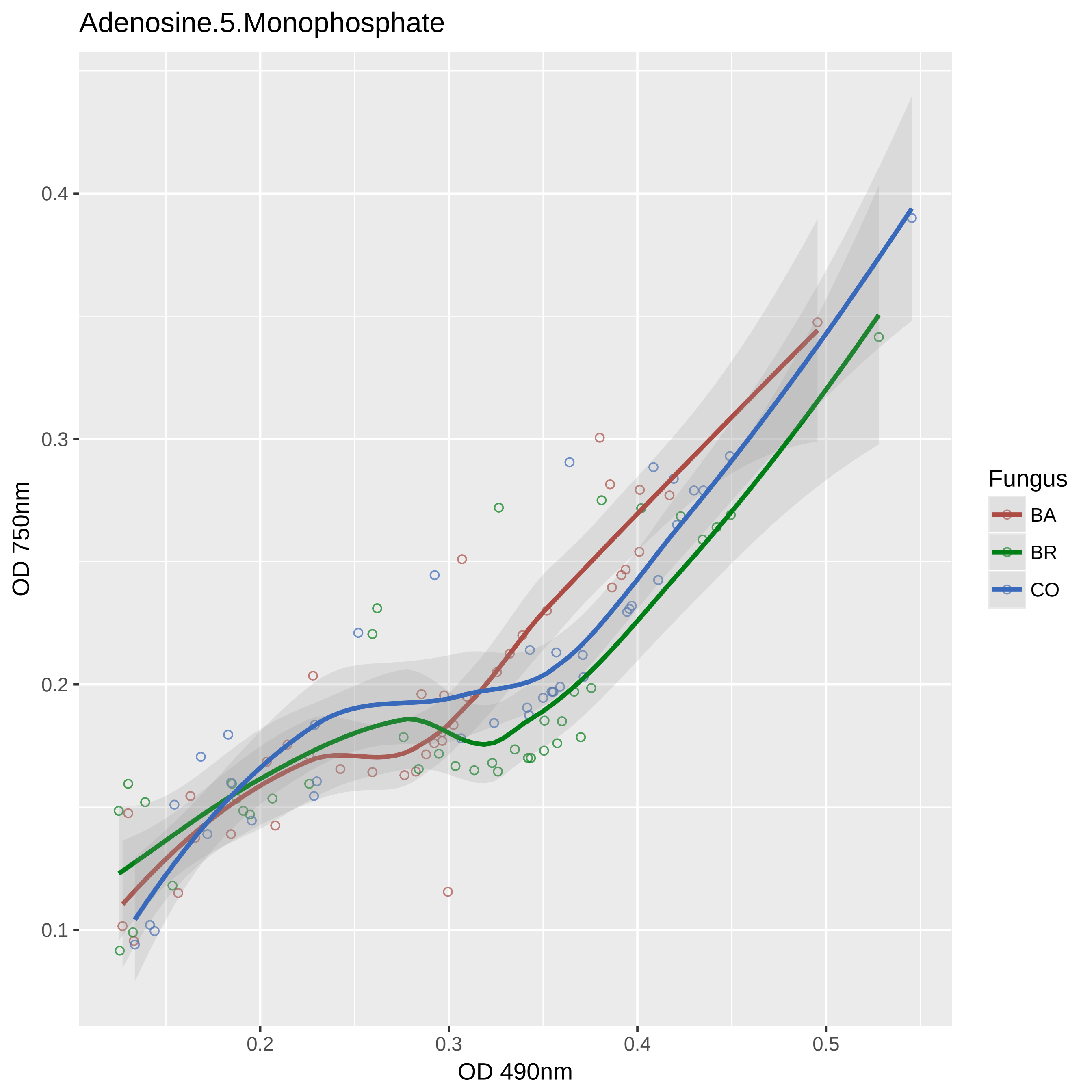

Adenosine.5.Monophosphate
OD 750nm
0.1
0.2
0.3
0.4
OD 490nm
0.2
0.3
0.4
0.5
Fungus
BA
BR
CO

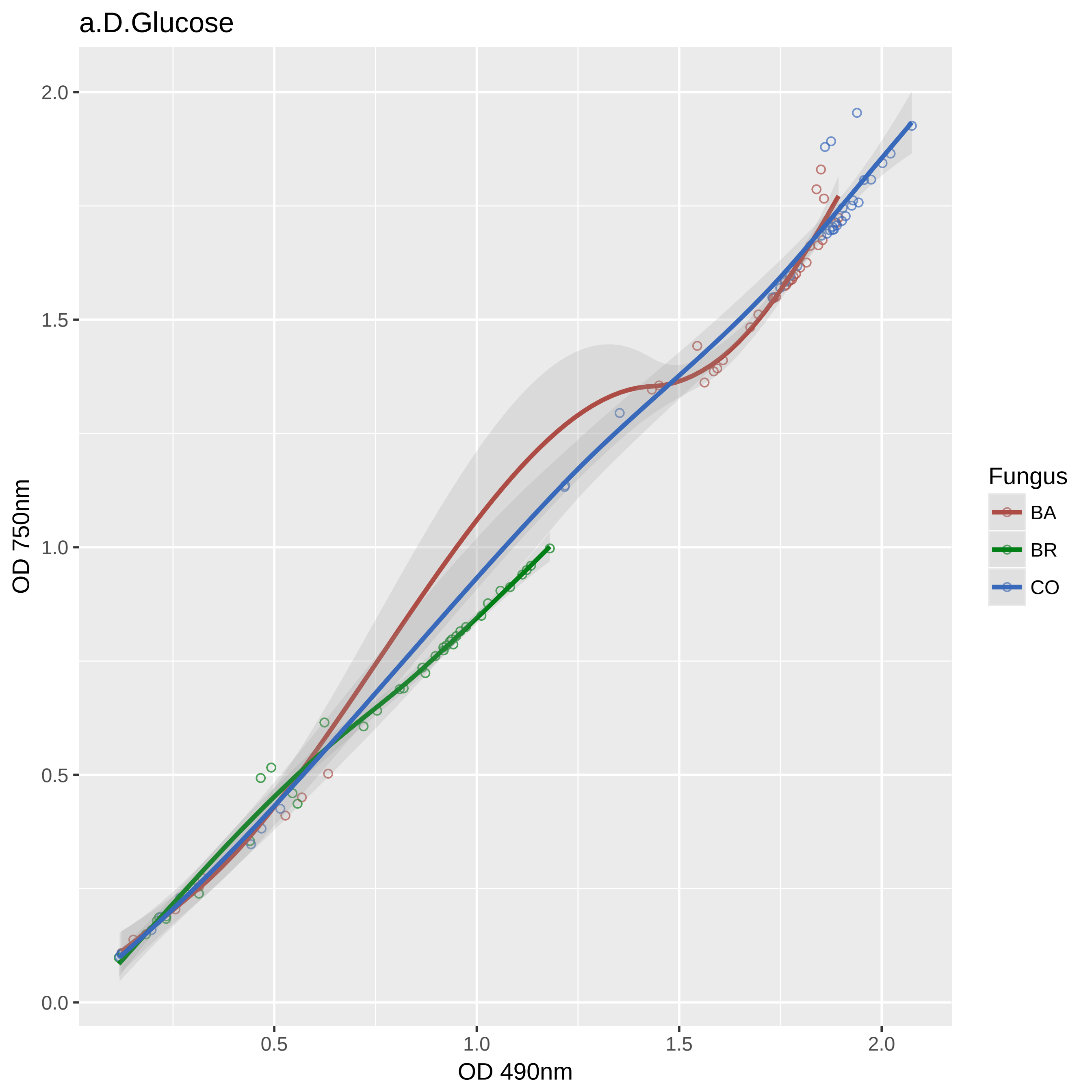
a.D.Glucose
2.0
1.5
1.0
0.5
0.0
0.5
1.0
1.5
2.0
OD 490nm
OD 750nm
Fungus
BA
BR
CO

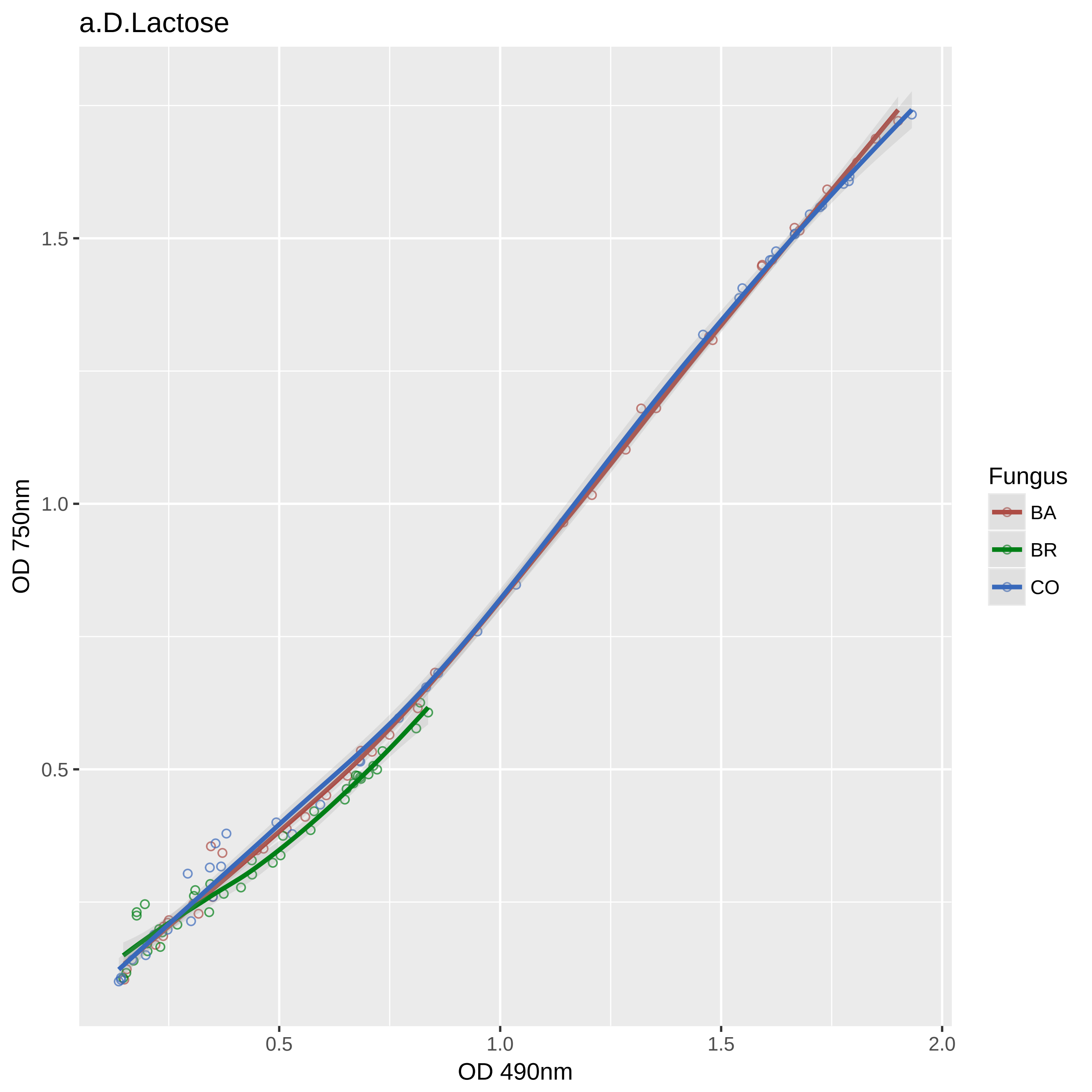

a.D.Lactose
OD 750nm
1.5
1.0
0.5
OD 490nm
0.5
1.0
1.5
2.0
Fungus
BA
BR
CO

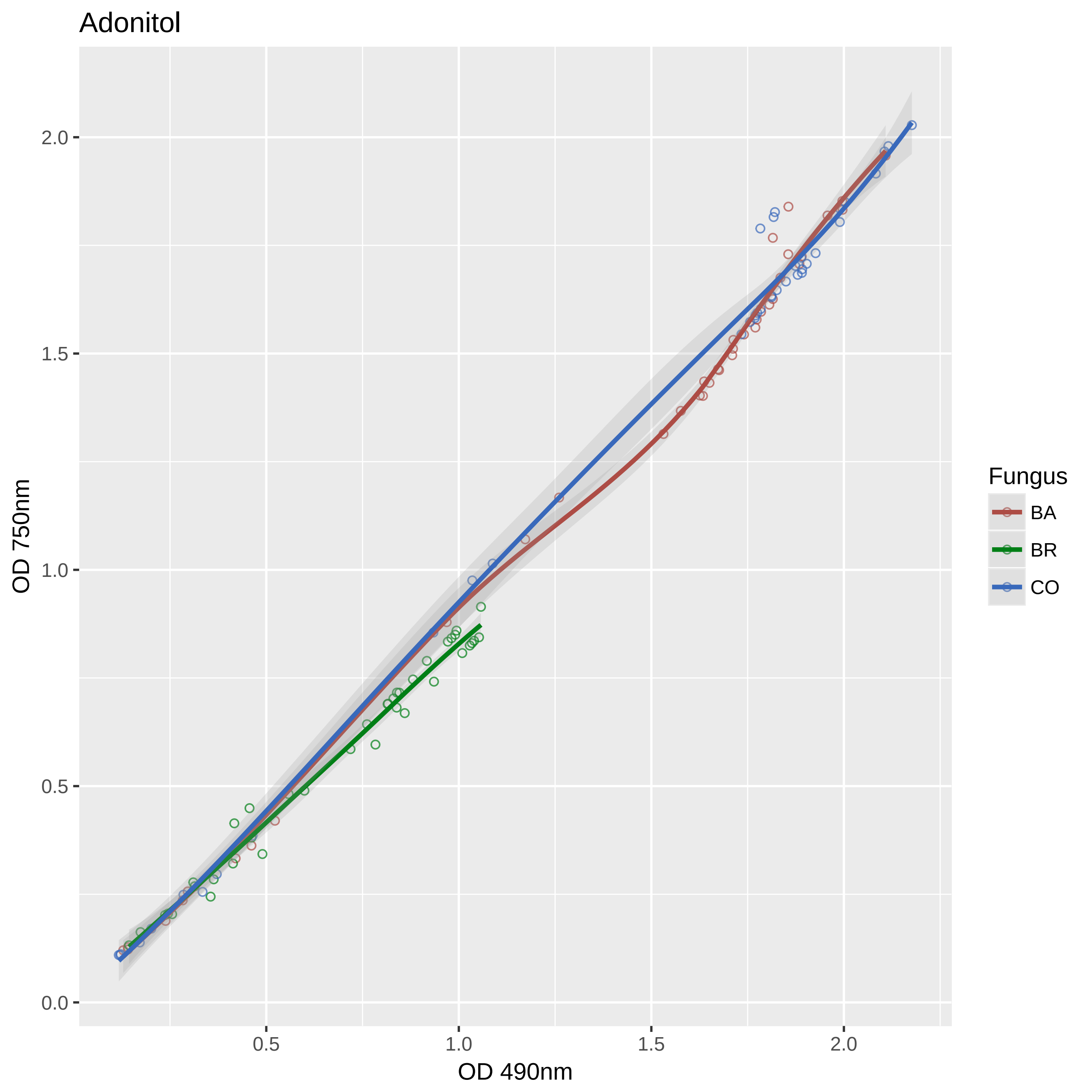

Adonitol
OD 750nm
OD 490nm
2.0
1.5
1.0
0.5
0.0
0.5
1.0
1.5
2.0
Fungus
BA
BR
CO

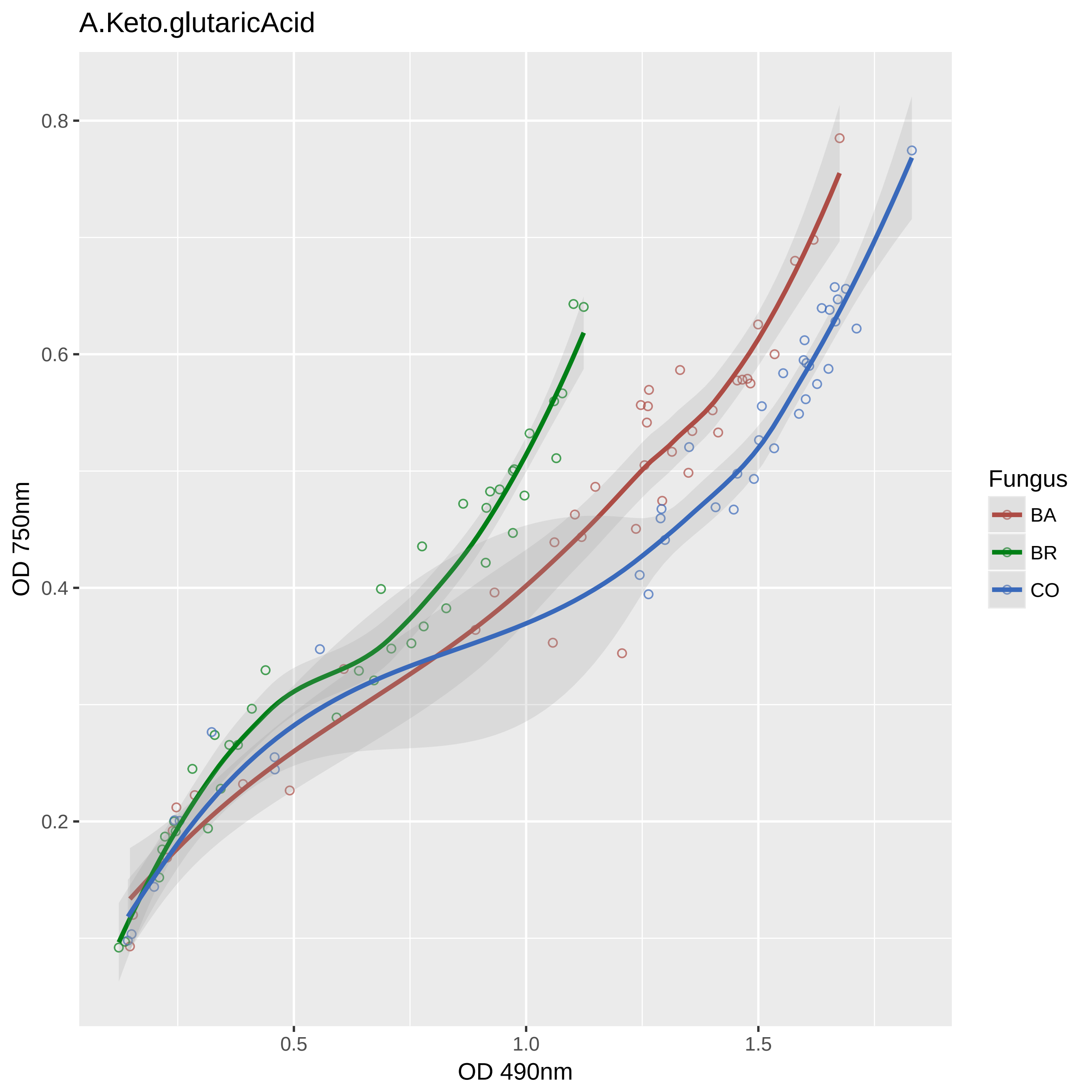
A.Keto.glutaricAcid
OD 750nm
0.8
0.6
0.4
0.2
OD 490nm
0.5
1.0
1.5
Fungus
BA
BR
CO

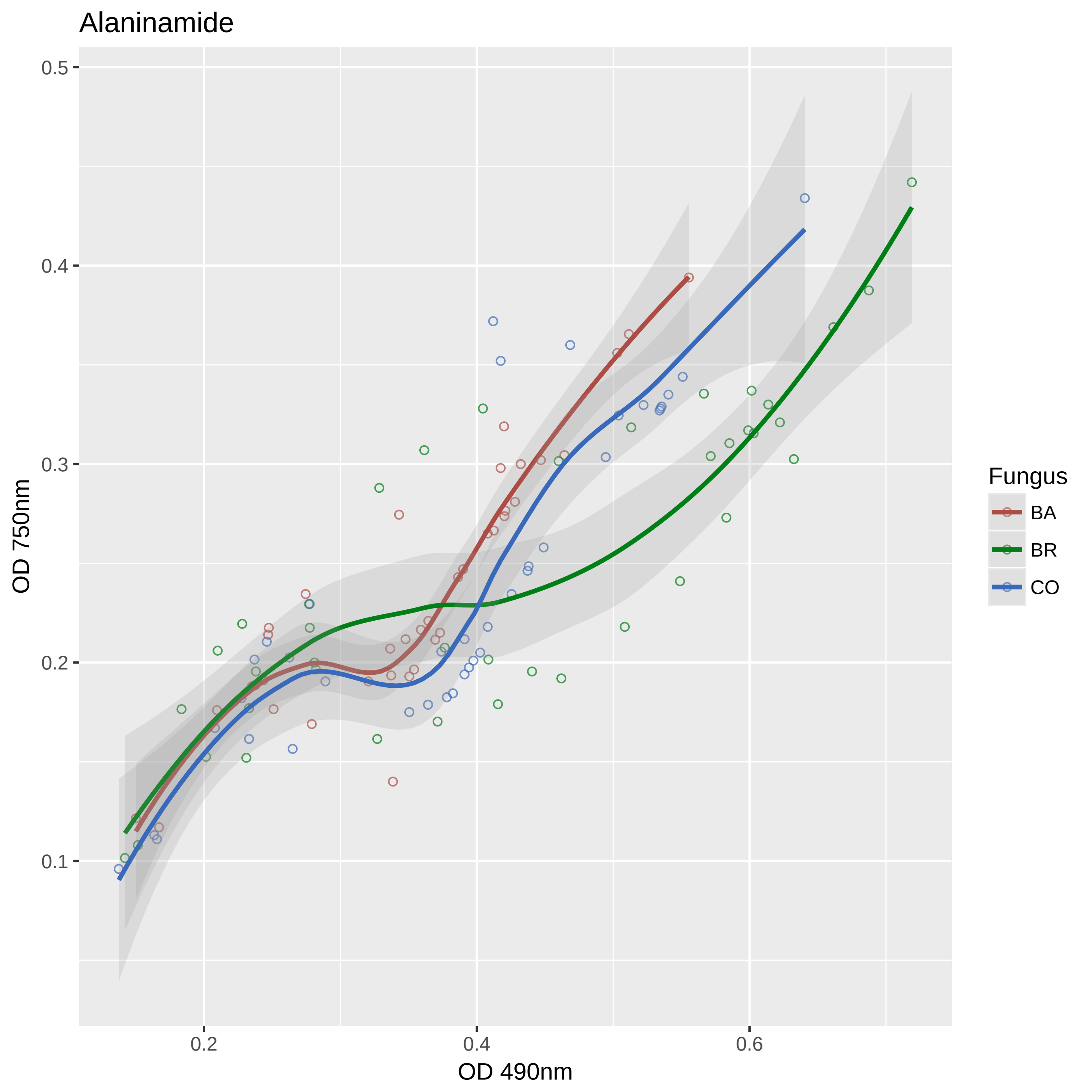

Alaninamide
0.5
0.4
0.3
0.2
0.1
OD 750nm
0.2
0.4
0.6
OD 490nm
Fungus
BA
BR
CO

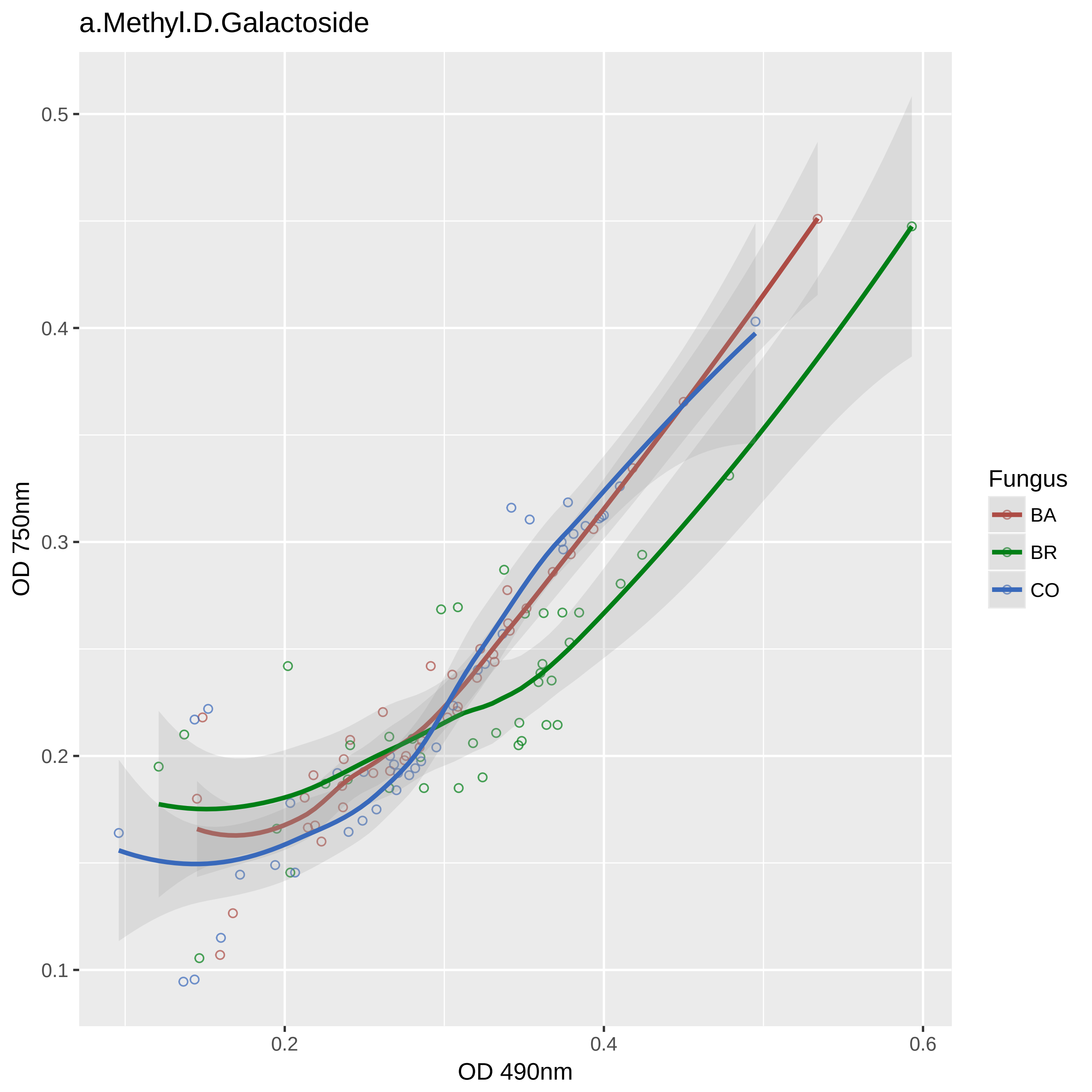

a.Methyl.D.Galactoside
OD 750nm
0.5
0.4
0.3
0.2
0.1
OD 490nm
0.2
0.4
0.6
Fungus
BA
BR
CO

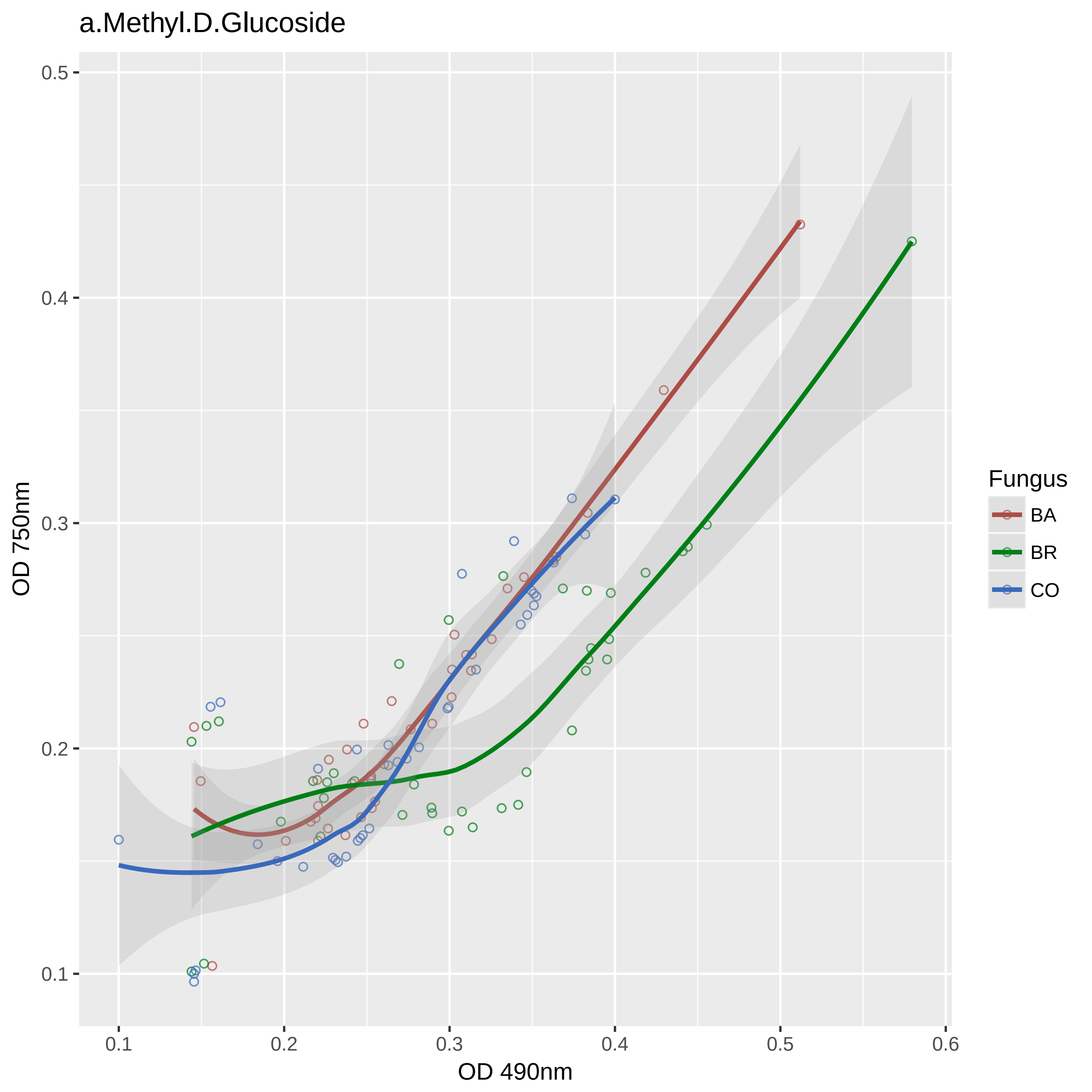

a.Methyl.D.Glucoside
OD 750nm
0.5
0.4
0.3
0.2
0.1
OD 490nm
0.1
0.2
0.3
0.4
0.5
0.6
Fungus
BA
BR
CO

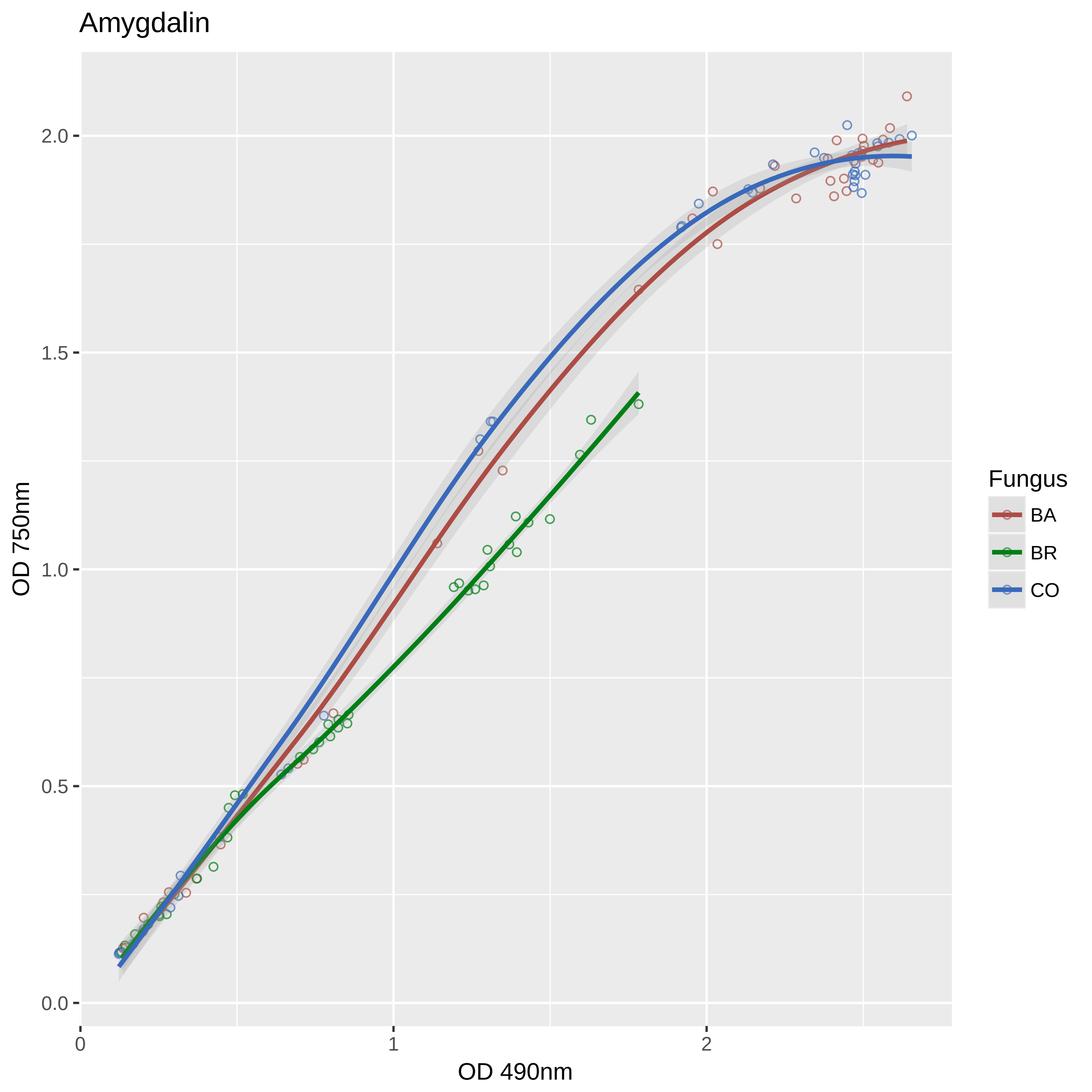

Amygdalin
OD 750nm
OD 490nm
0.0
0.5
1.0
1.5
2.0
0
1
2
Fungus
BA
BR
CO

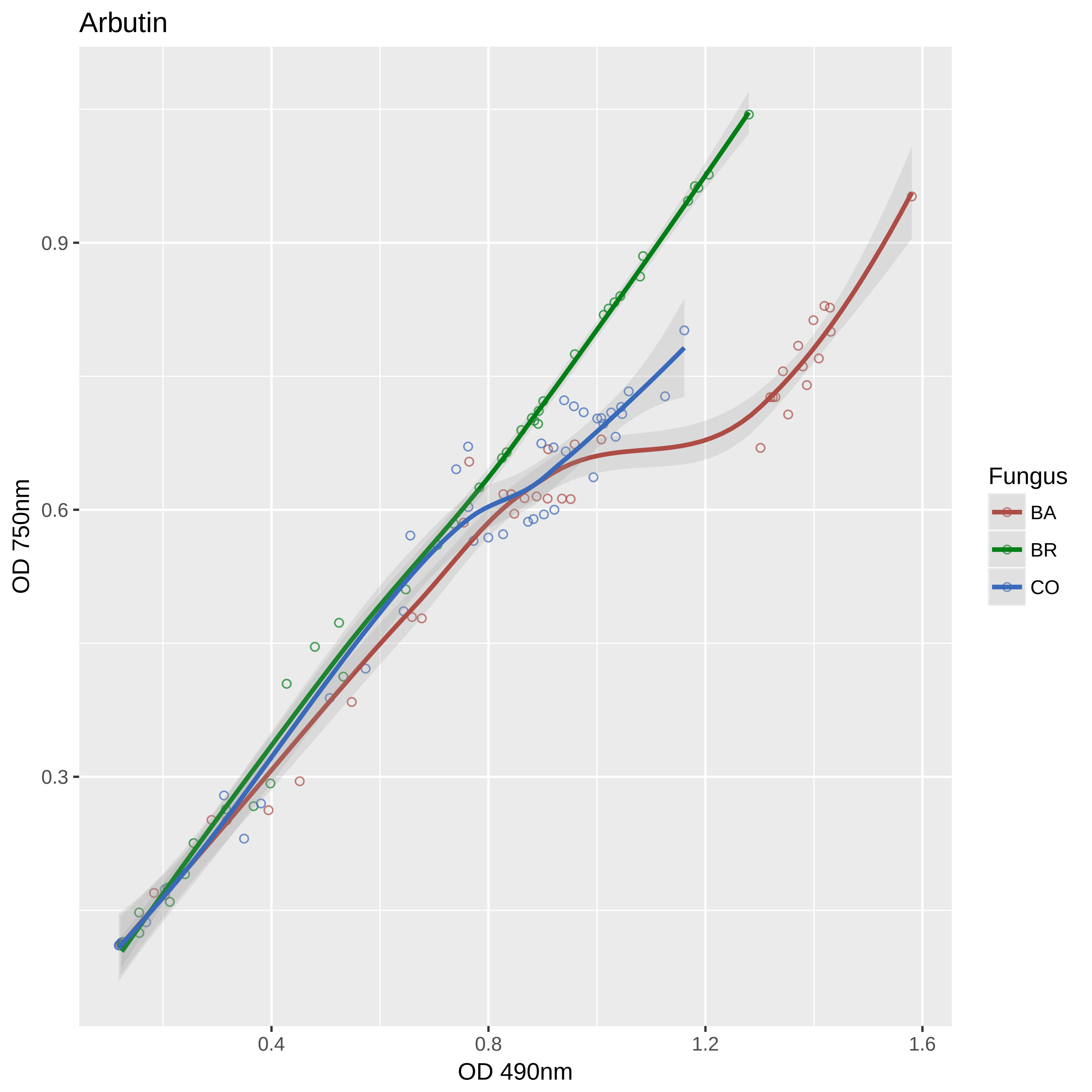

Arbutin
0.3
0.6
0.9
OD 750nm
0.4
0.8
1.2
1.6
OD 490nm
Fungus
BA
BR
CO

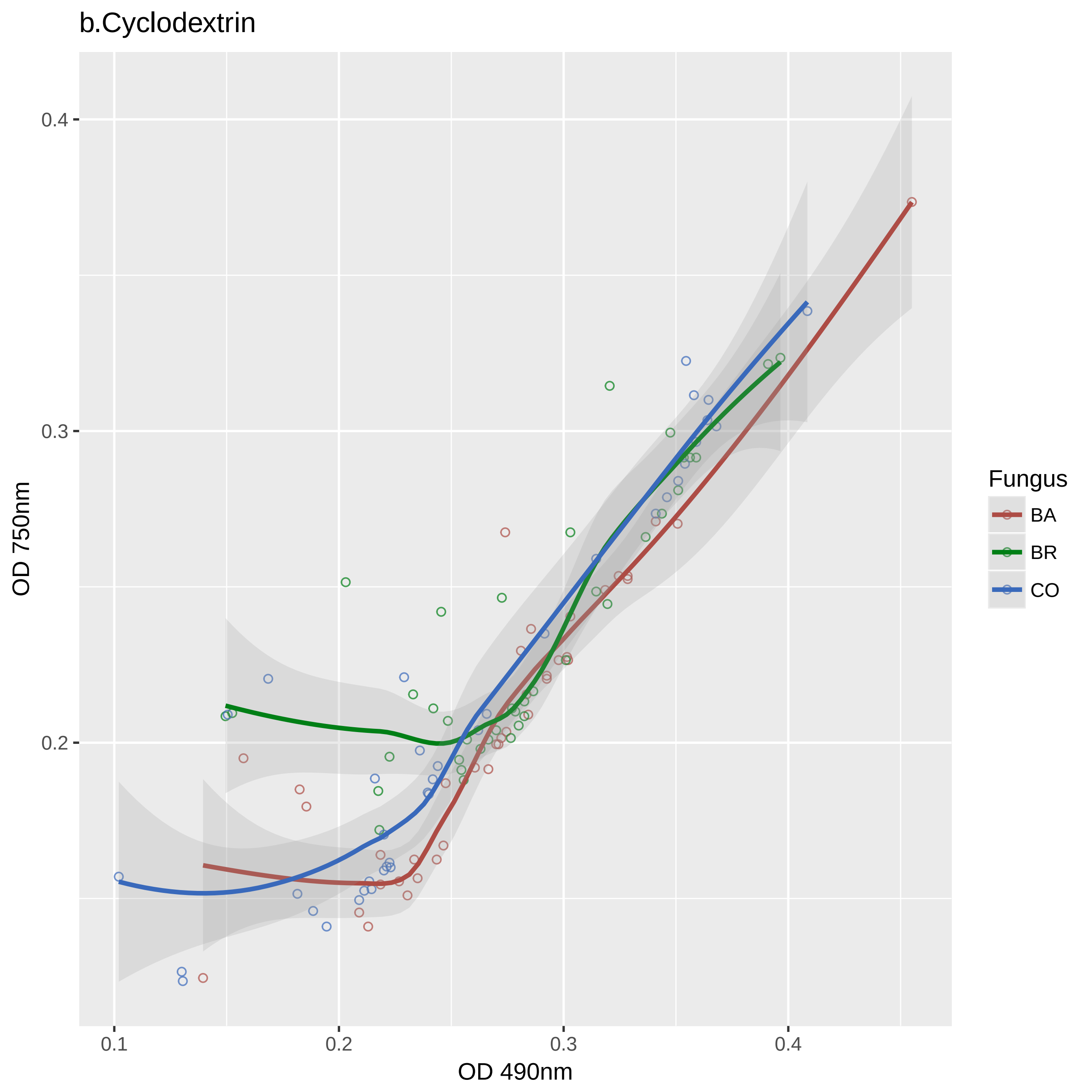

b.Cyclodextrin
OD 750nm
0.4
0.3
0.2
OD 490nm
0.1
0.2
0.3
0.4
Fungus
BA
BR
CO

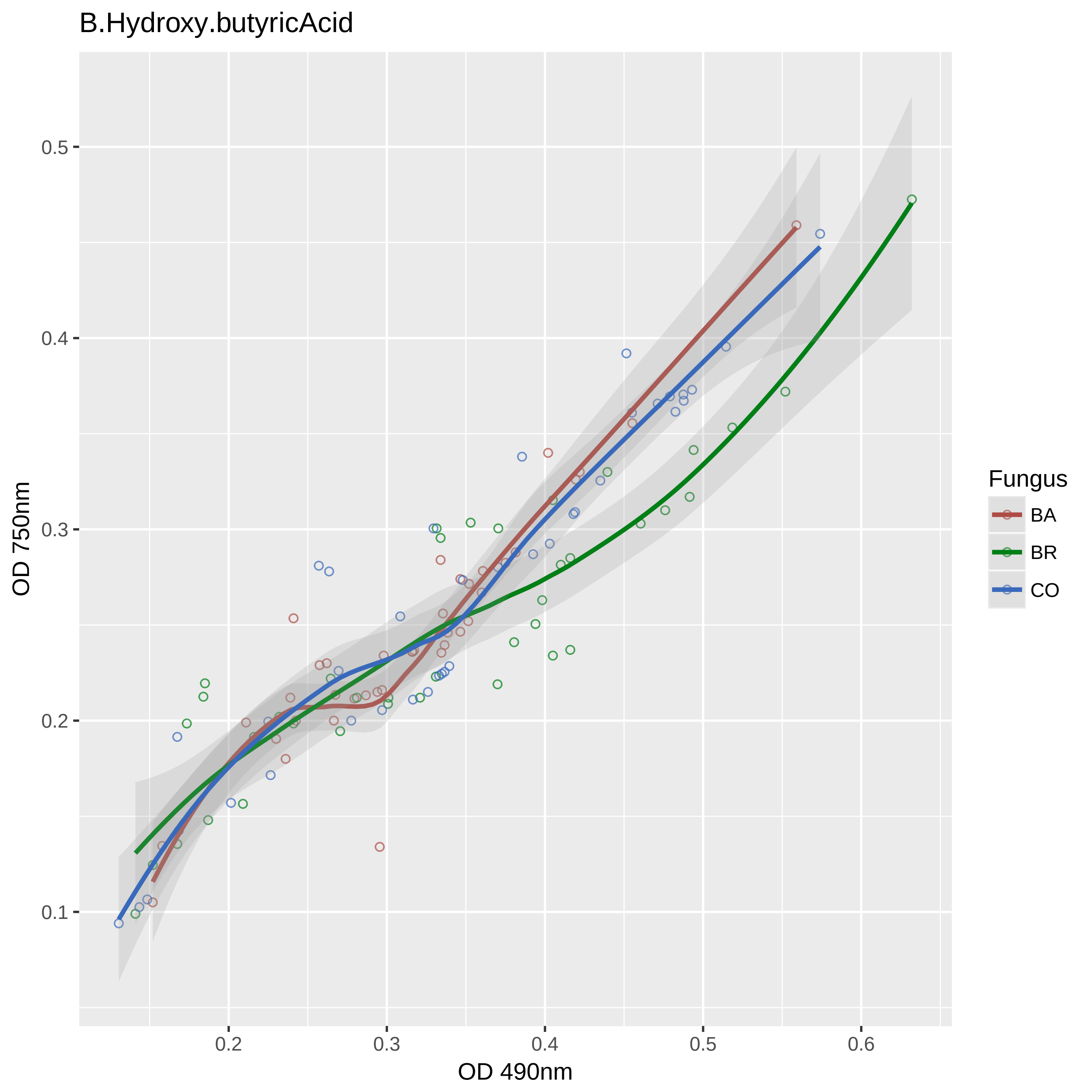
B.Hydroxy.butyricAcid
OD 750nm
OD 490nm
0.1
0.2
0.3
0.4
0.5
0.2
0.3
0.4
0.5
0.6
Fungus
BA
BR
CO

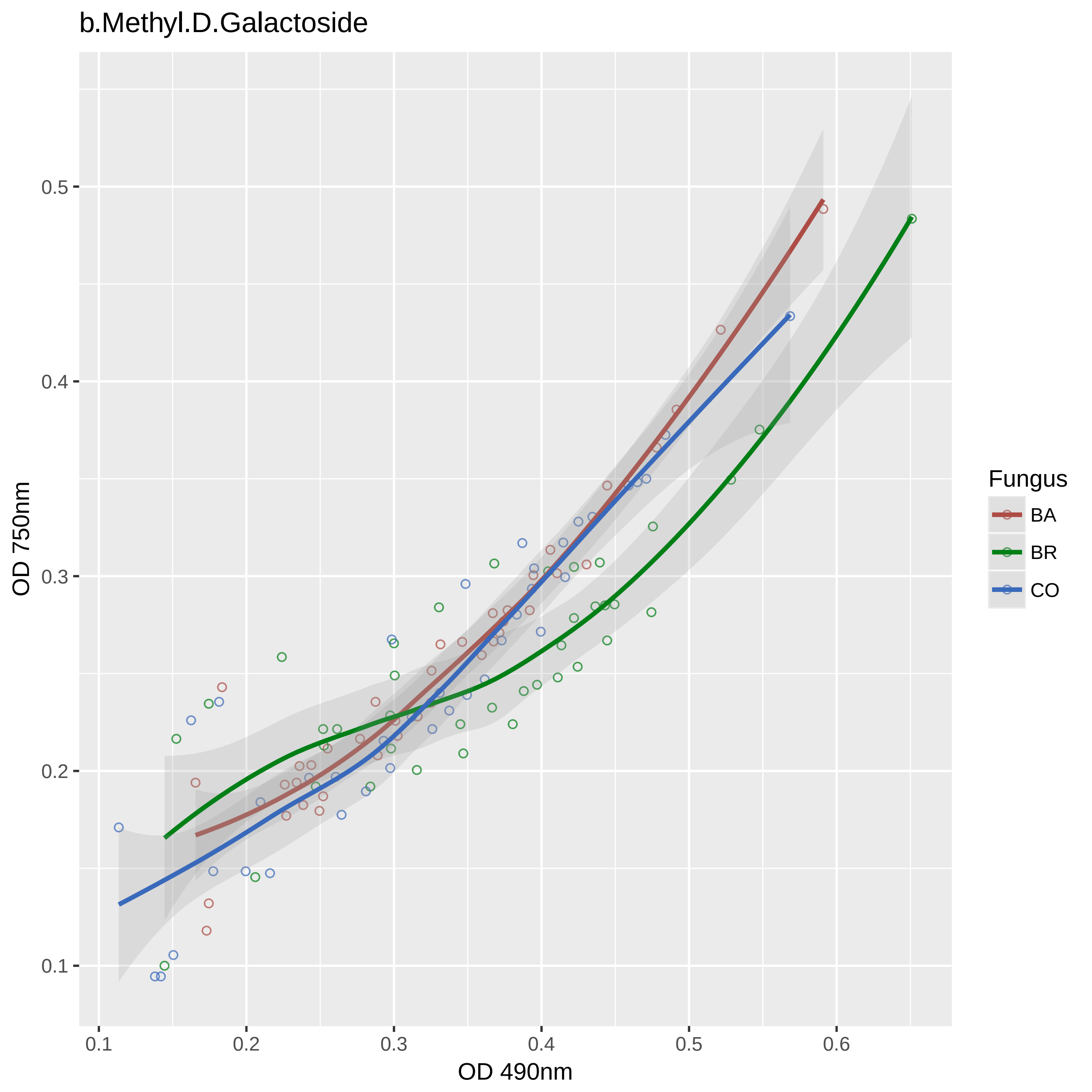

b.Methyl.D.Galactoside
OD 750nm
OD 490nm
0.5
0.4
0.3
0.2
0.1
0.1
0.2
0.3
0.4
0.5
0.6
Fungus
BA
BR
CO

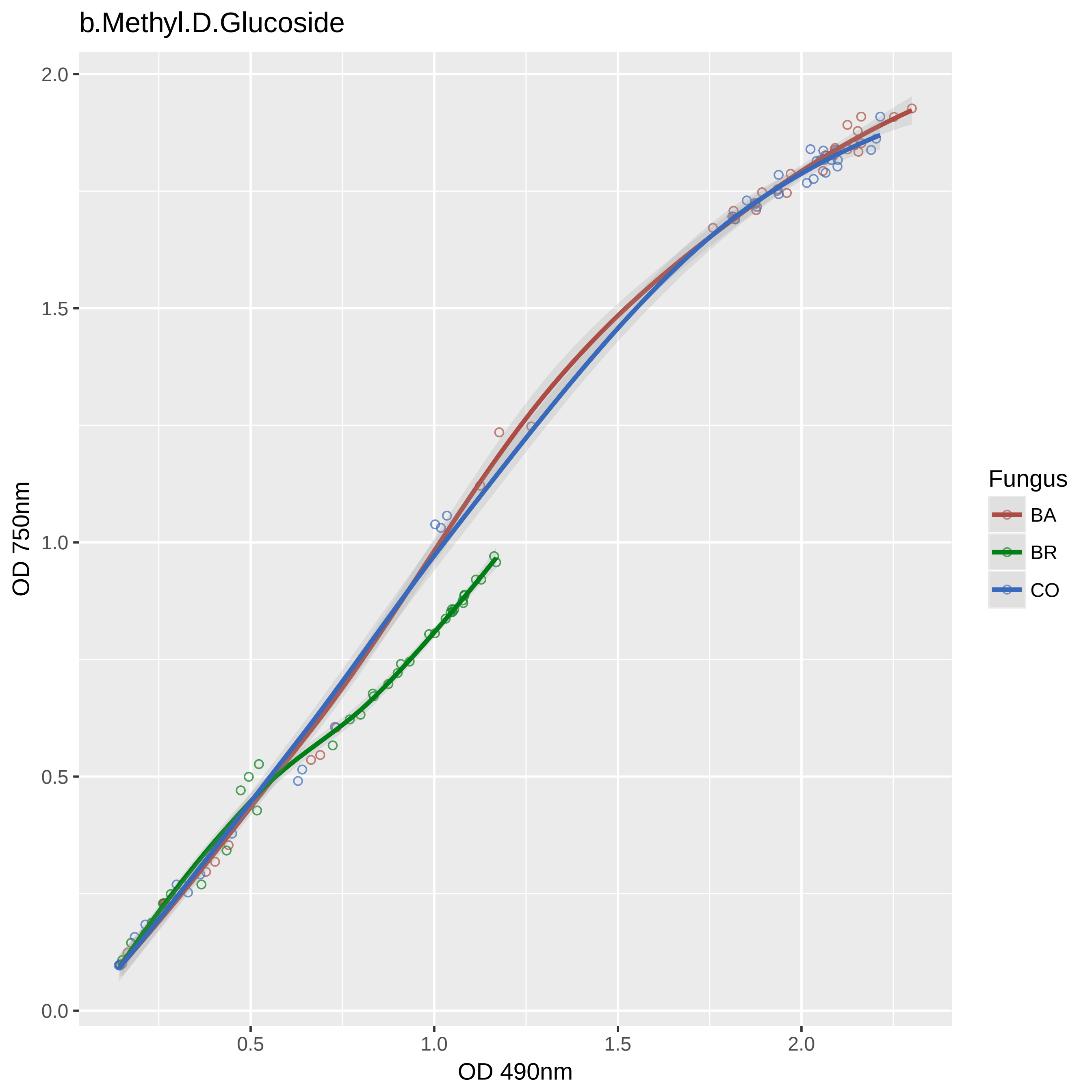

b.Methyl.D.Glucoside
OD 750nm
OD 490nm
0.0
0.5
1.0
1.5
2.0
0.5
1.0
1.5
2.0
Fungus
BA
BR
CO

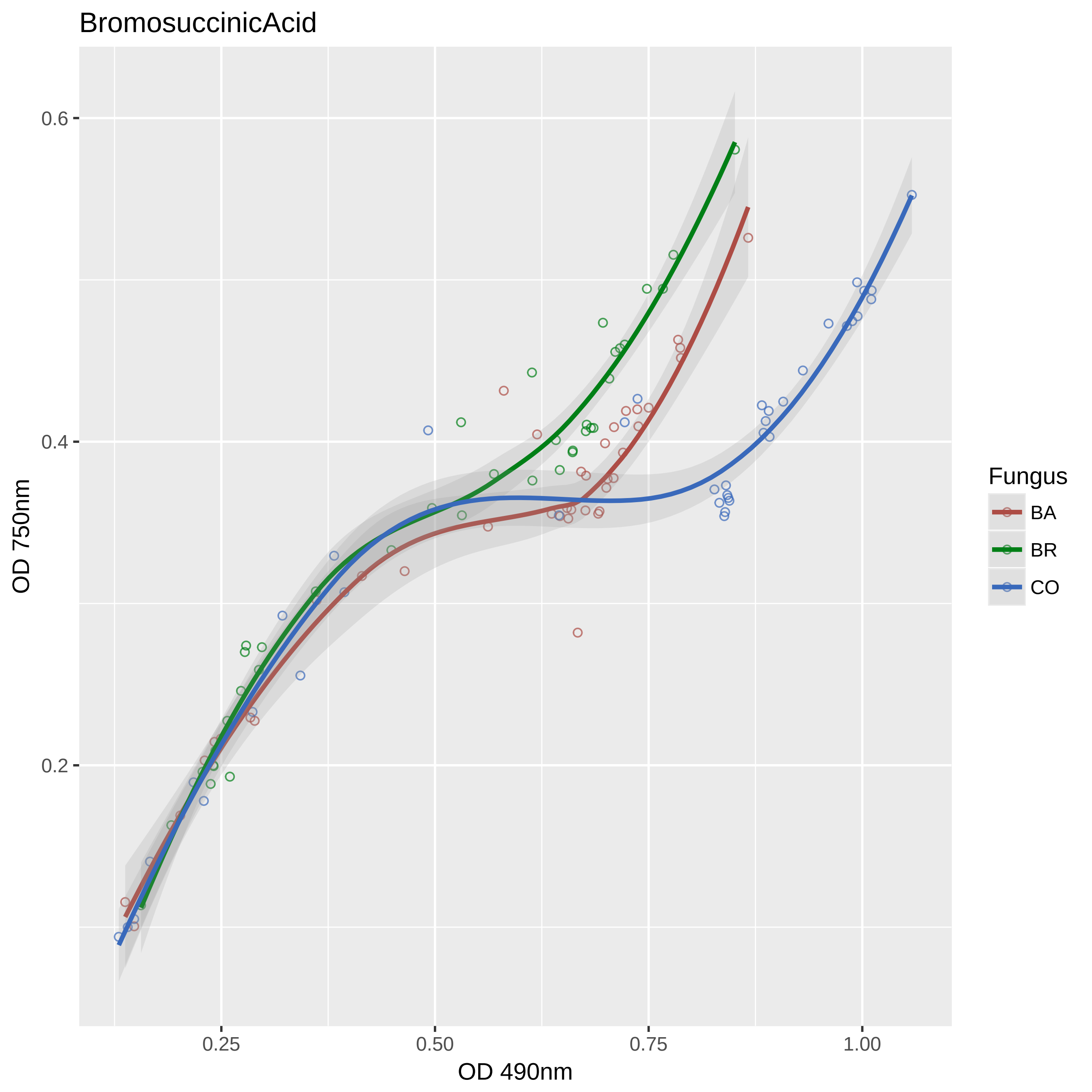

BromosuccinicAcid
0.6
0.4
0.2
0.25
0.50
0.75
1.00
OD 750nm
OD 490nm
Fungus
BA
BR
CO

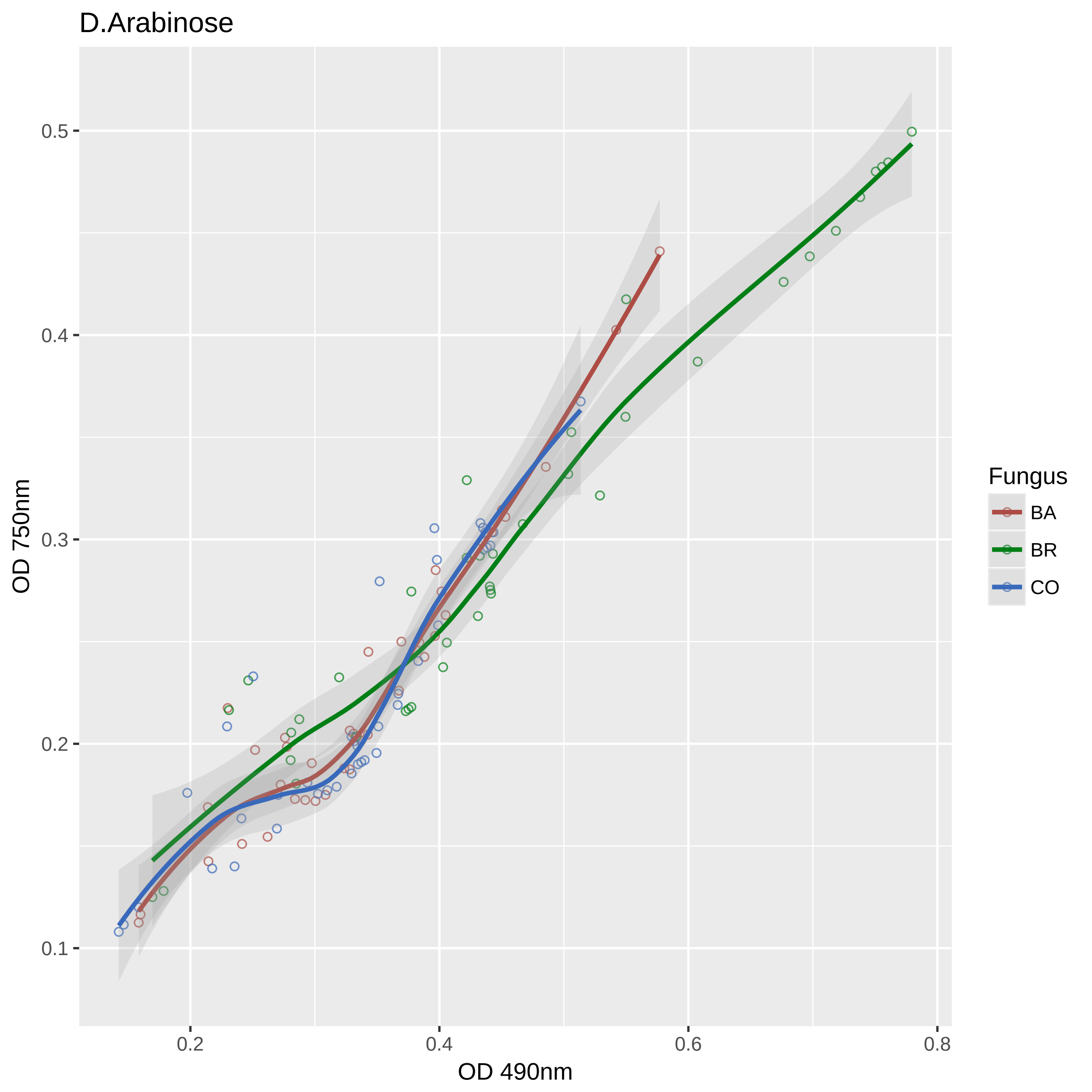
D.Arabinose
OD 750nm
0.1
0.2
0.3
0.4
0.5
OD 490nm
0.2
0.4
0.6
0.8
Fungus
BA
BR
CO

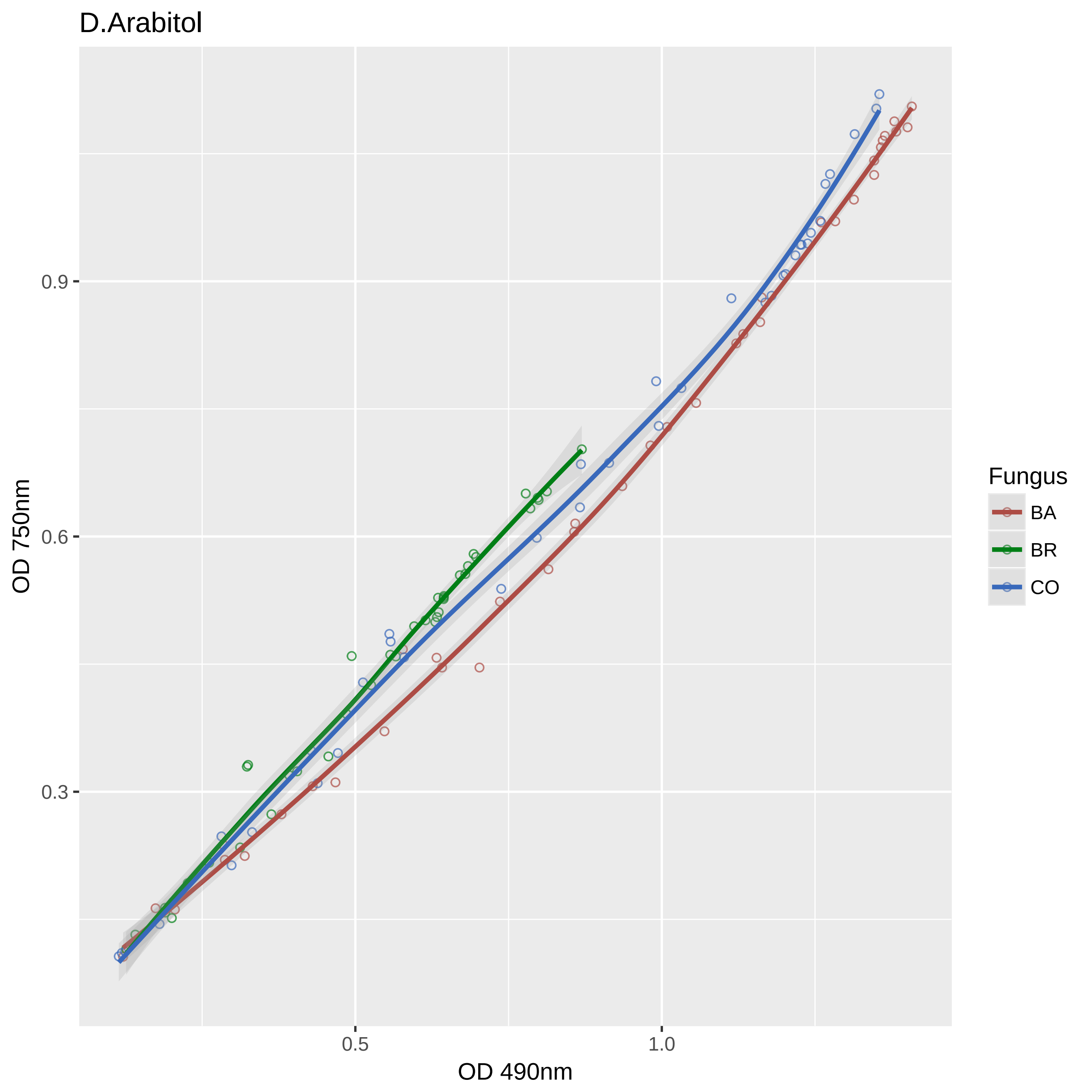

D.Arabitol
OD 750nm
0.9
0.6
0.3
0.5
1.0
OD 490nm
Fungus
BA
BR
CO

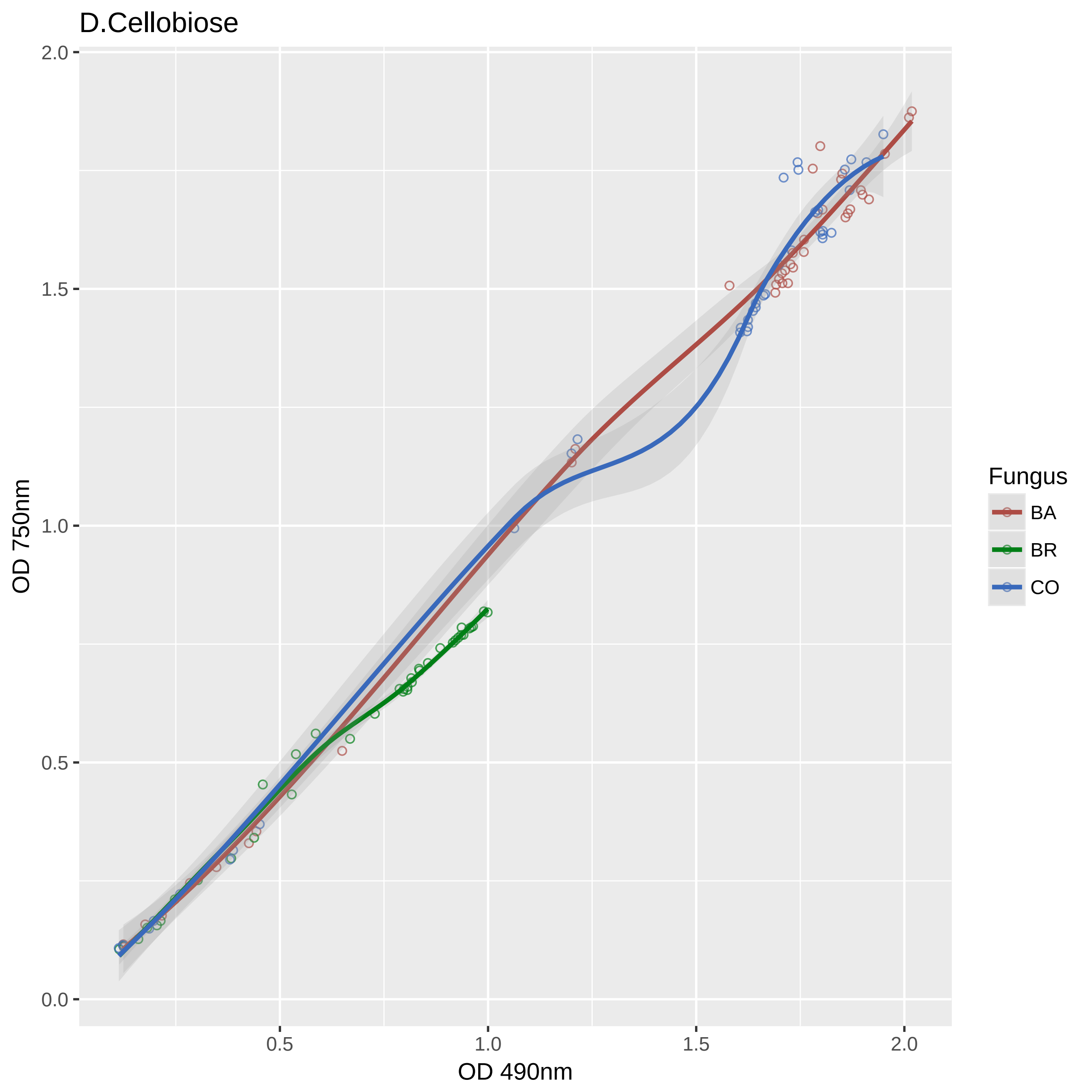

D.Cellobiose
OD 750nm
OD 490nm
2.0
1.5
1.0
0.5
0.0
0.5
1.0
1.5
2.0
Fungus
BA
BR
CO

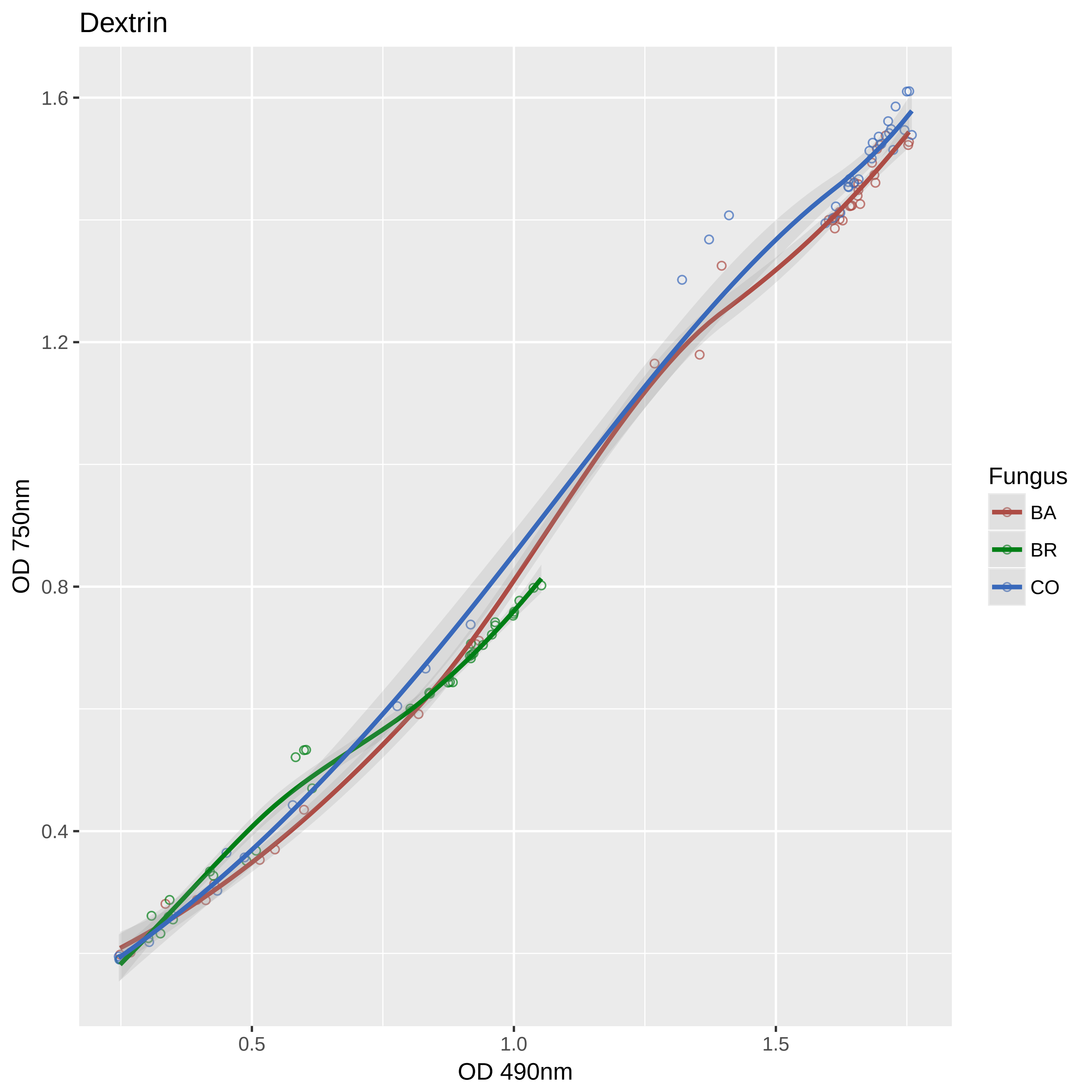

Dextrin
OD 750nm
1.6
1.2
0.8
0.4
OD 490nm
0.5
1.0
1.5
Fungus
BA
BR
CO

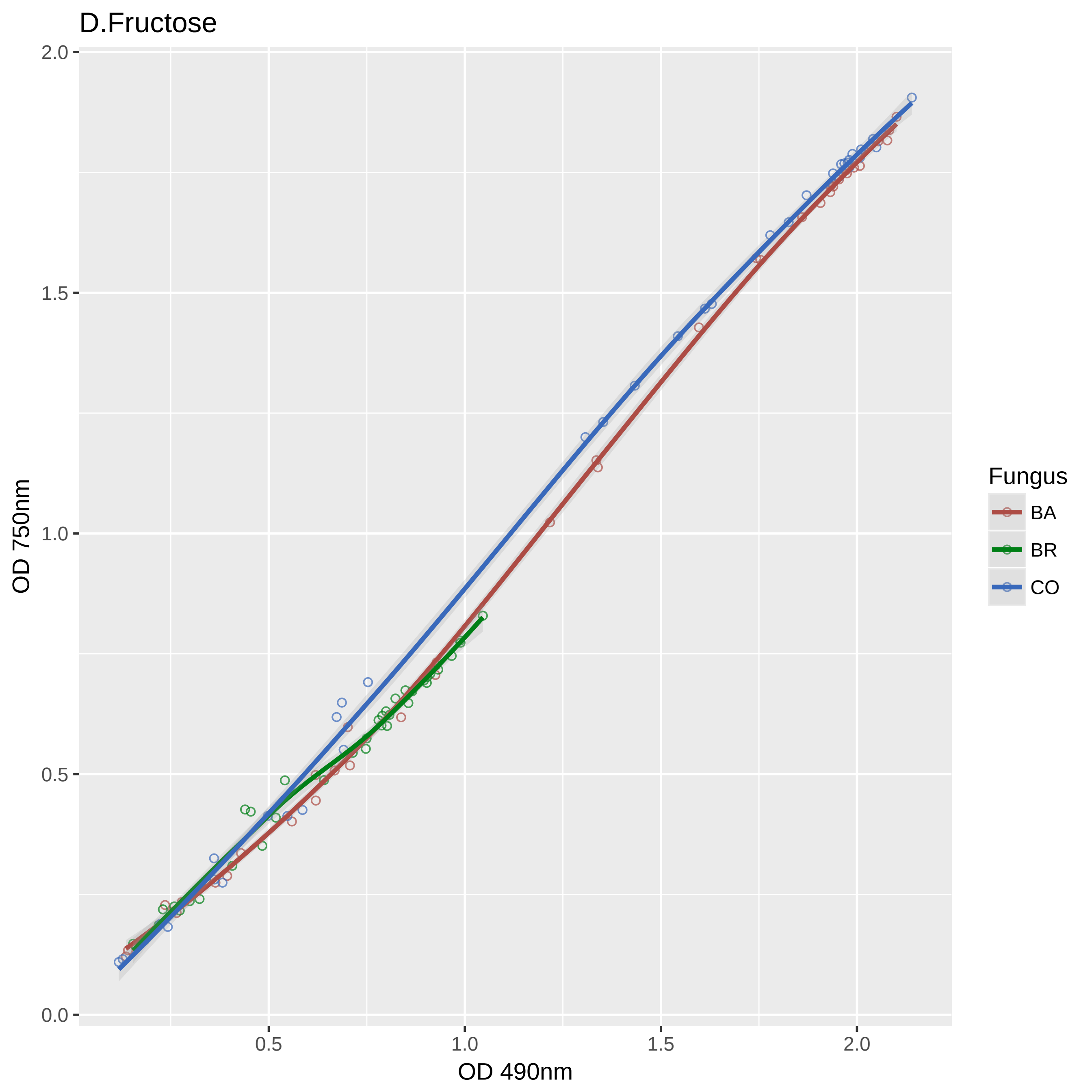

D.Fructose
OD 750nm
OD 490nm
0.0
0.5
1.0
1.5
2.0
0.5
1.0
1.5
2.0
Fungus
BA
BR
CO

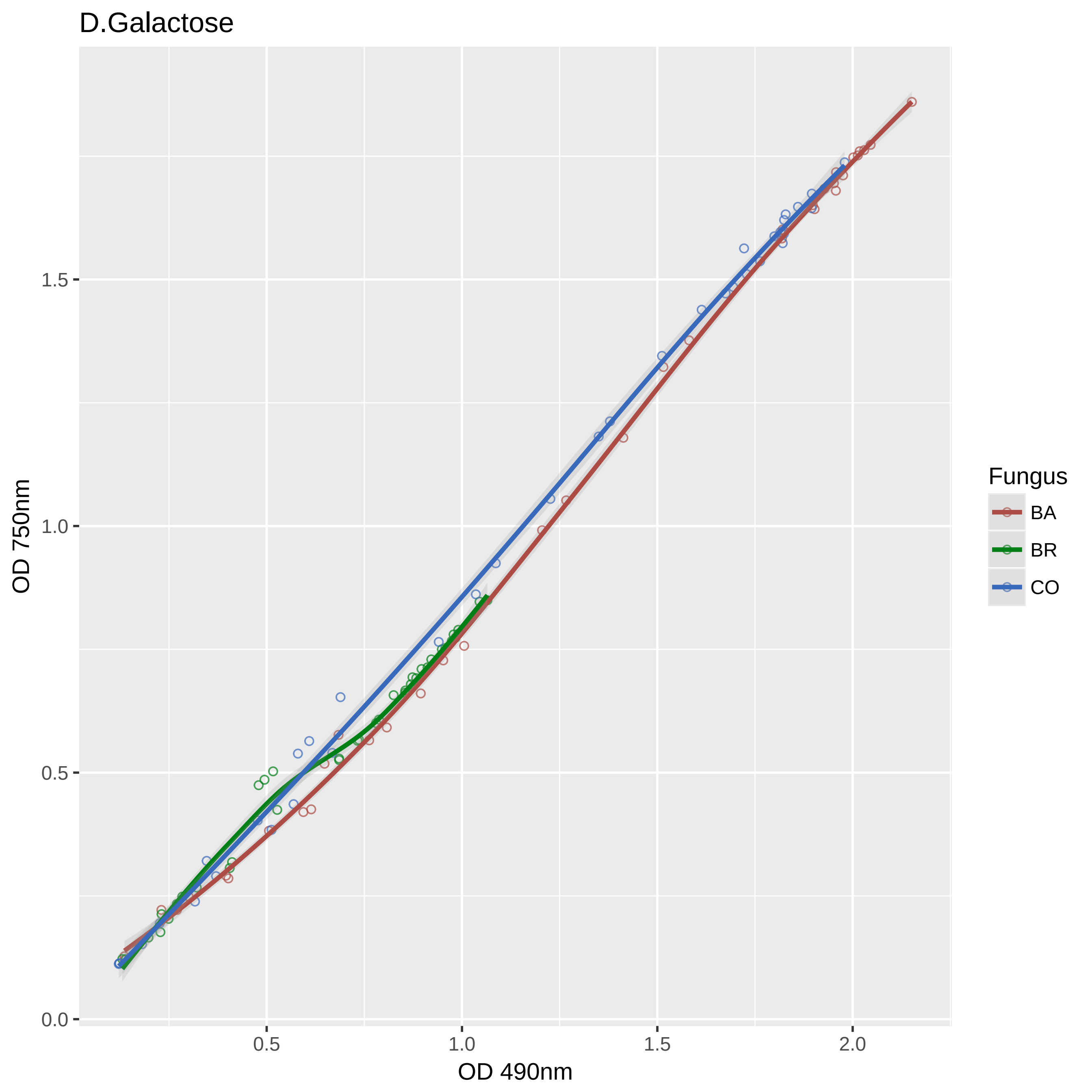

D.Galactose
OD 750nm
OD 490nm
0.0
0.5
1.0
1.5
0.5
1.0
1.5
2.0
Fungus
BA
BR
CO

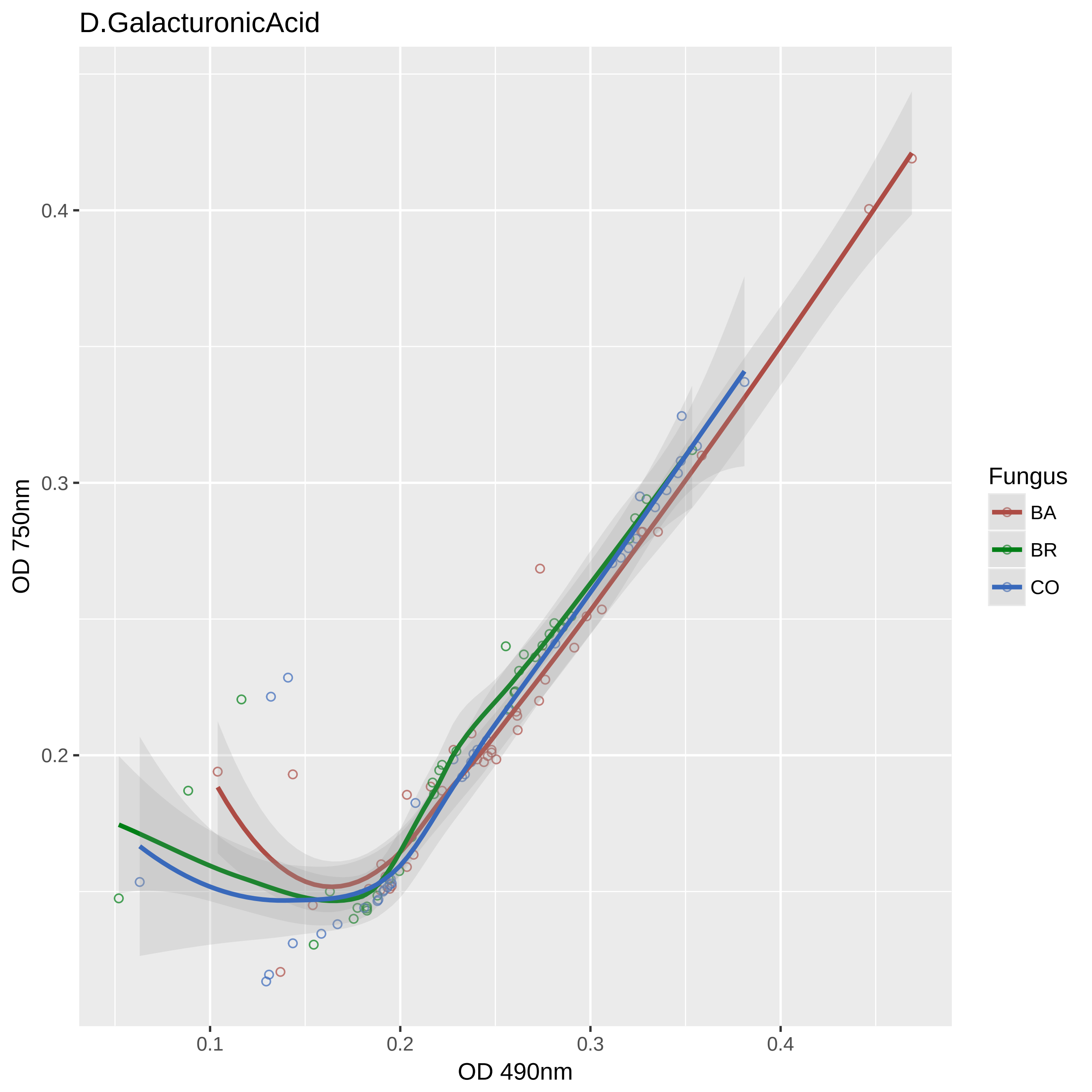

D.GalacturonicAcid
OD 750nm
0.4
0.3
0.2
OD 490nm
0.1
0.2
0.3
0.4
Fungus
BA
BR
CO

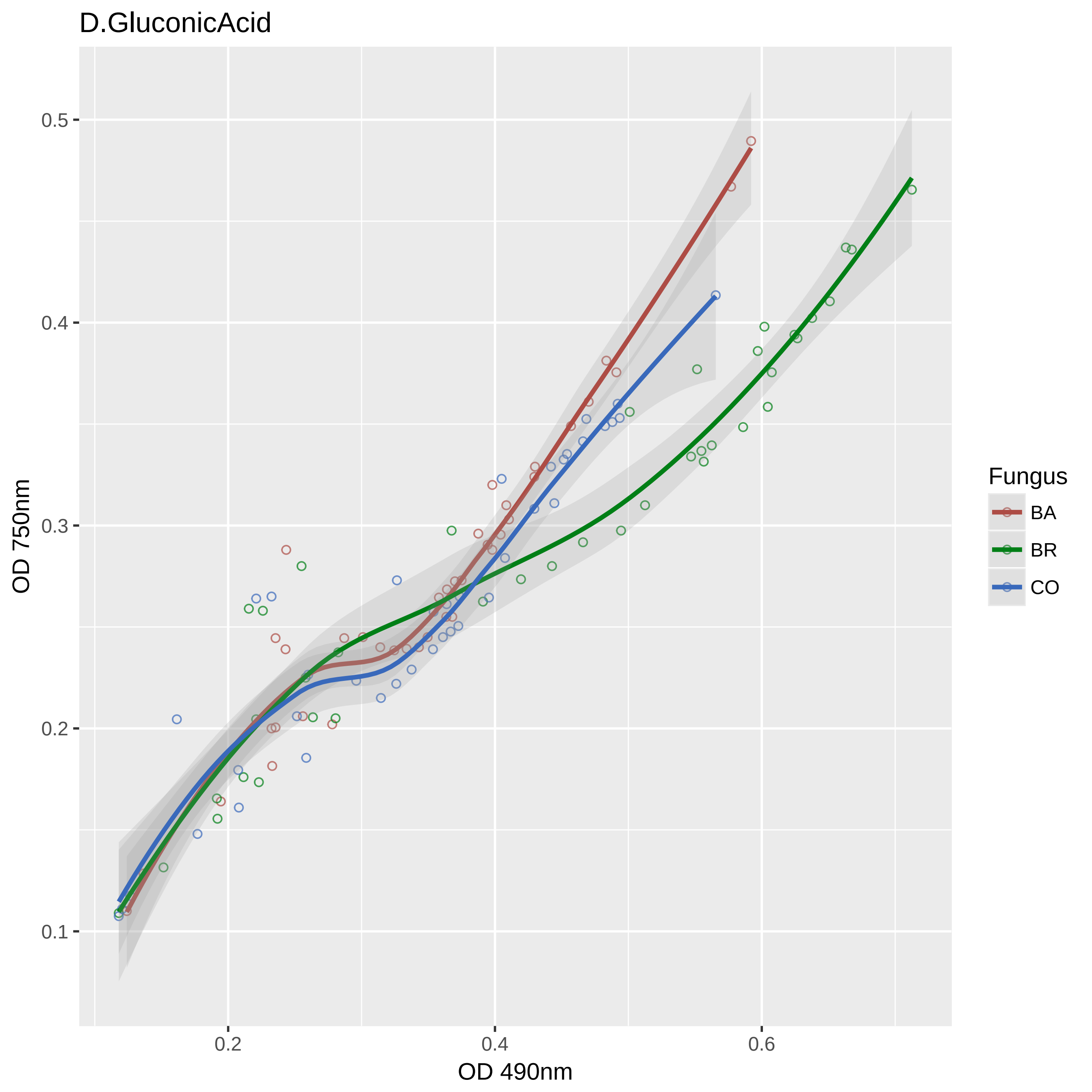

D.GluconicAcid
OD 750nm
OD 490nm
0.1
0.2
0.3
0.4
0.5
0.2
0.4
0.6
Fungus
BA
BR
CO

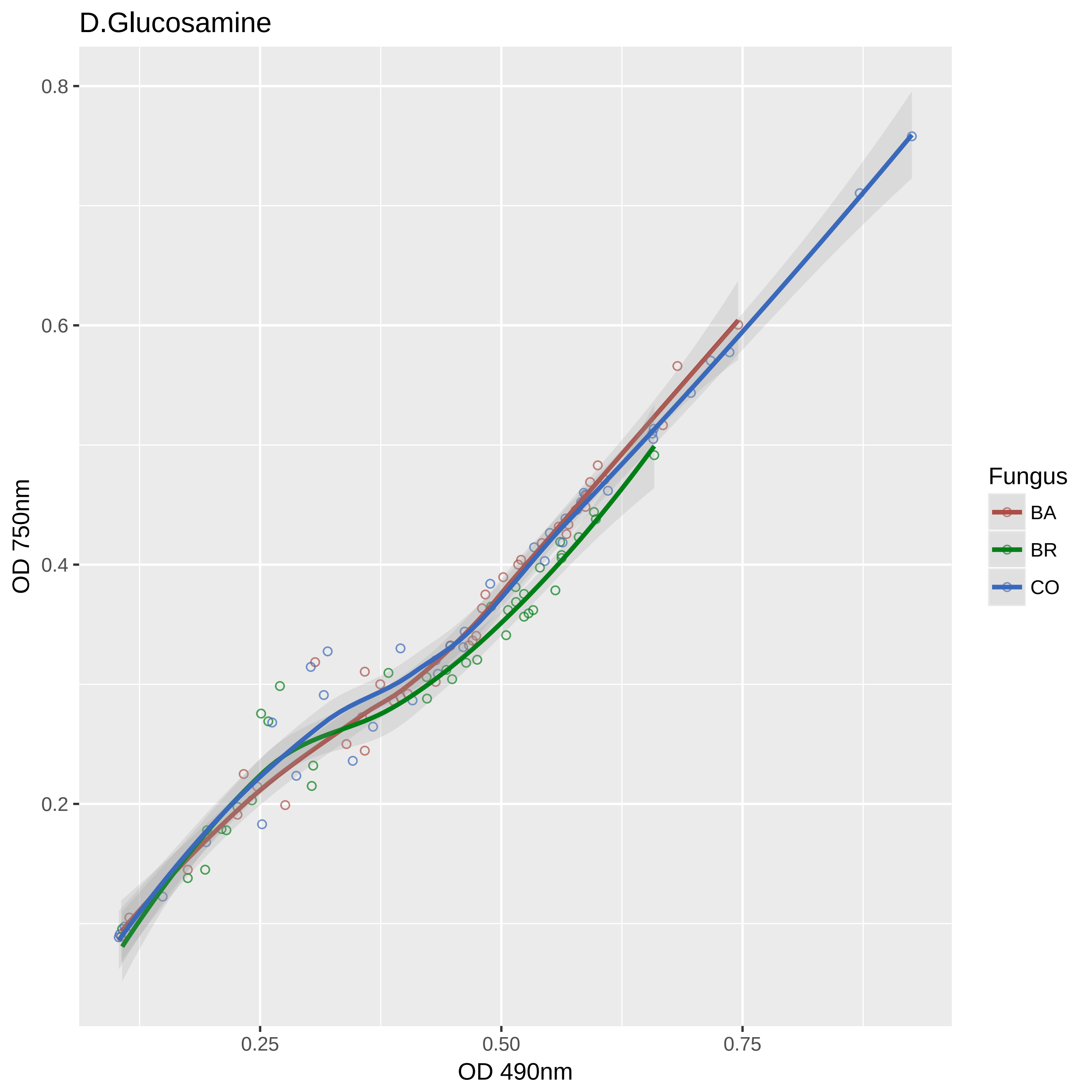
D.Glucosamine
0.8
0.6
0.4
0.2
OD 750nm
0.25
0.50
0.75
OD 490nm
Fungus
BA
BR
CO

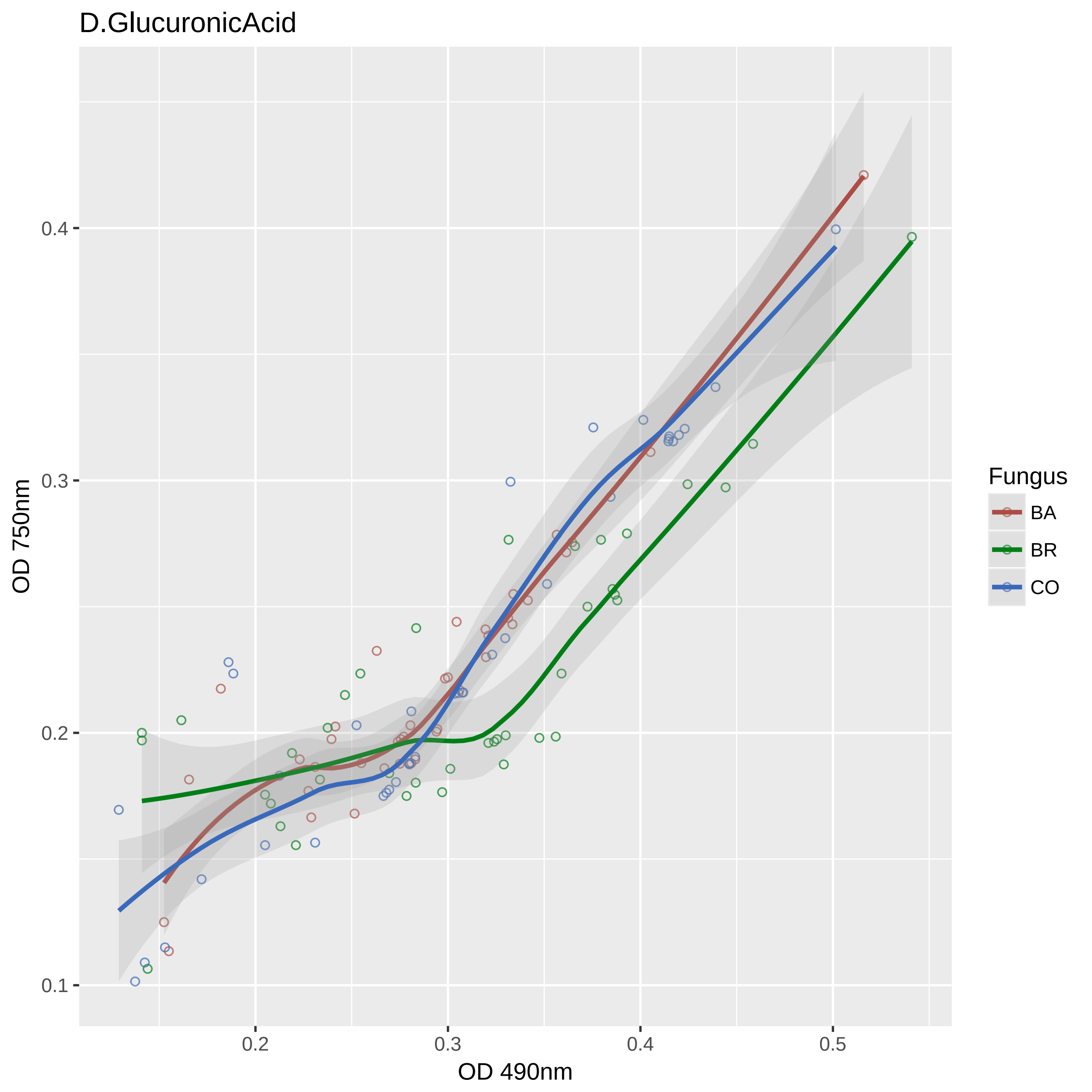

D.GlucuronicAcid
OD 750nm
0.1
0.2
0.3
0.4
0.2
0.3
0.4
0.5
OD 490nm
Fungus
BA
BR
CO

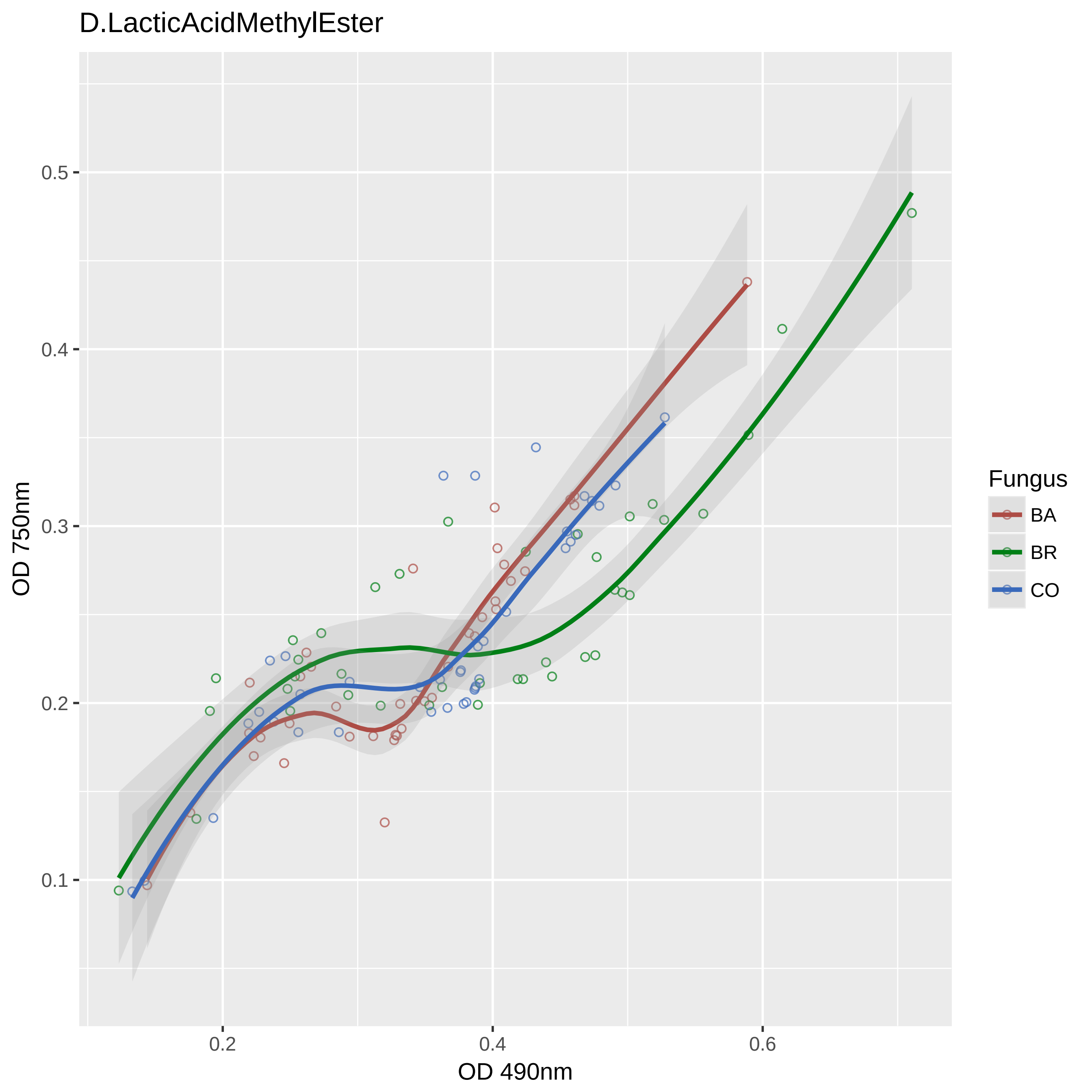

D.LacticAcidMethylEster
OD 750nm
0.5
0.4
0.3
0.2
0.1
OD 490nm
0.2
0.4
0.6
Fungus
BA
BR
CO

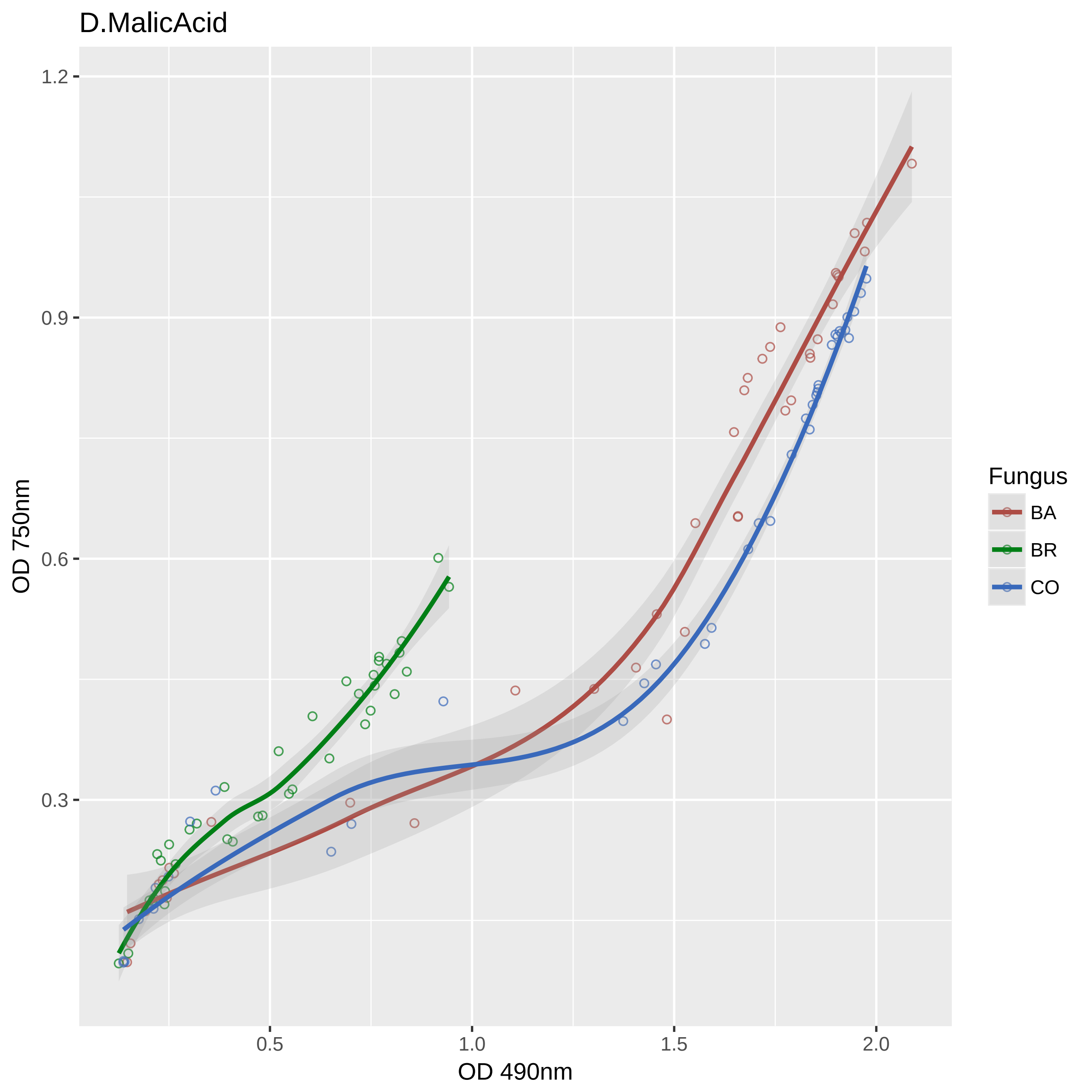

D.MalicAcid
1.2
0.9
0.6
0.3
OD 750nm
0.5
1.0
1.5
2.0
OD 490nm
Fungus
BA
BR
CO

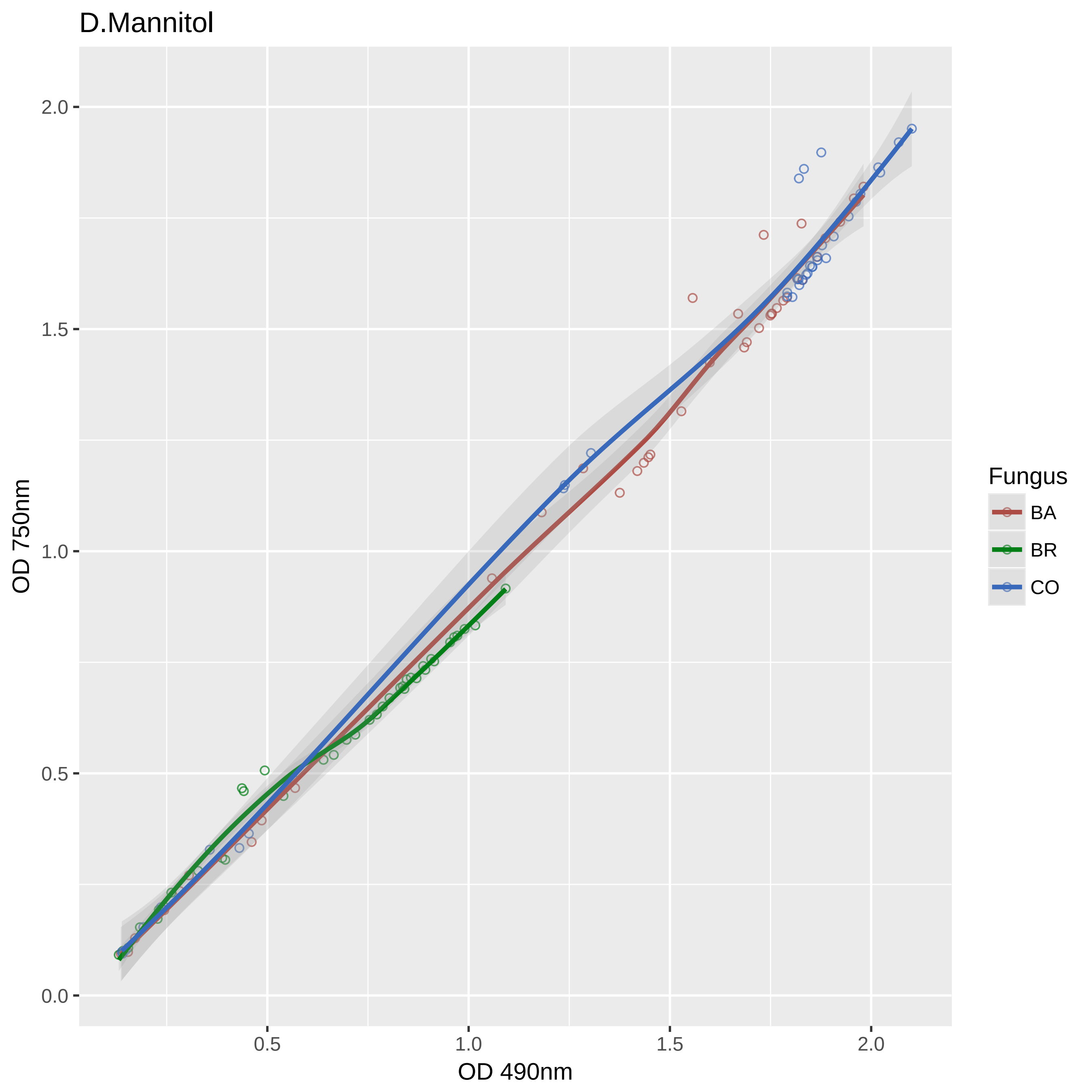

D.Mannitol
2.0
1.5
1.0
0.5
0.0
0.5
1.0
1.5
2.0
OD 490nm
OD 750nm
Fungus
BA
BR
CO

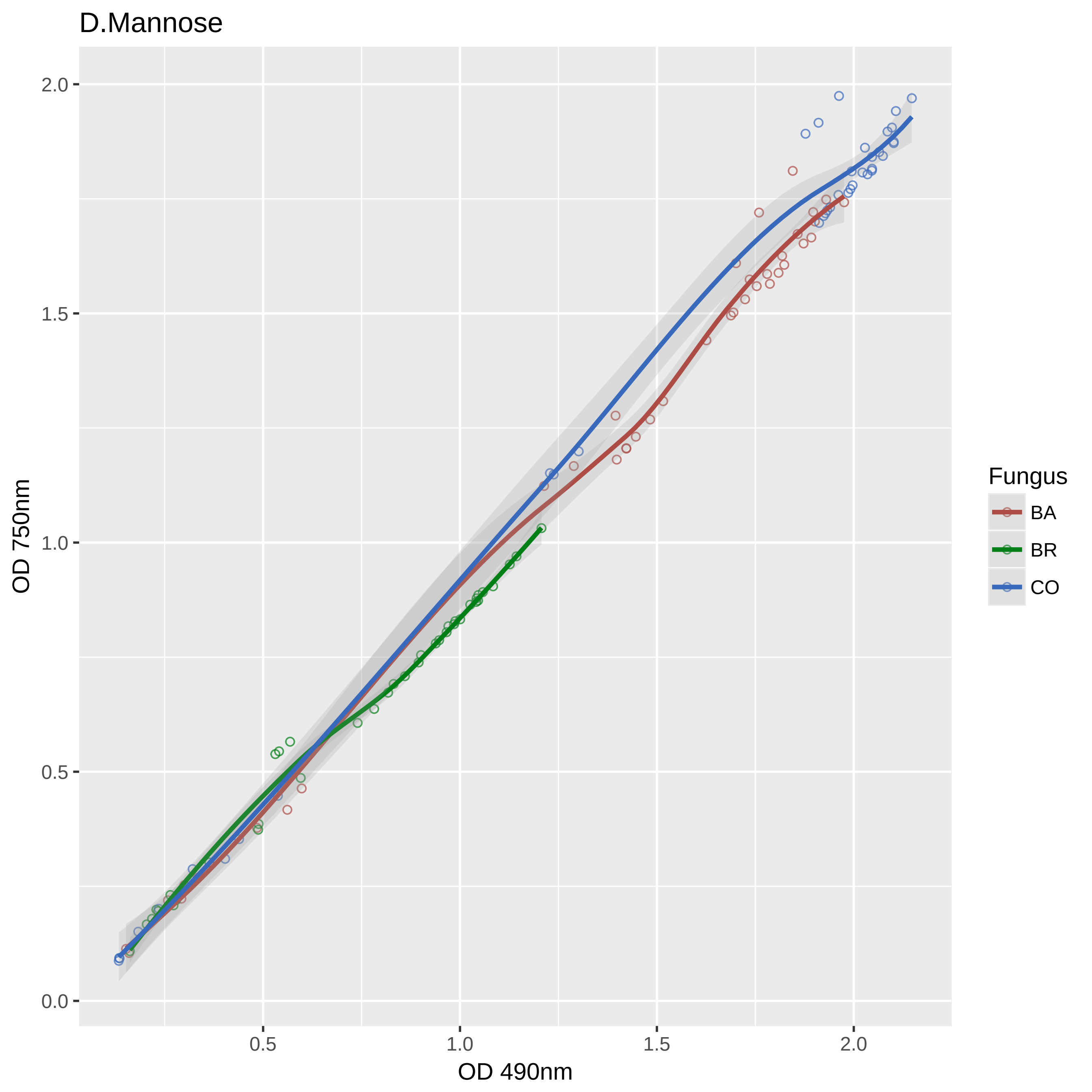
D.Mannose
OD 750nm
OD 490nm
0.0
0.5
1.0
1.5
2.0
0.5
1.0
1.5
2.0
Fungus
BA
BR
CO

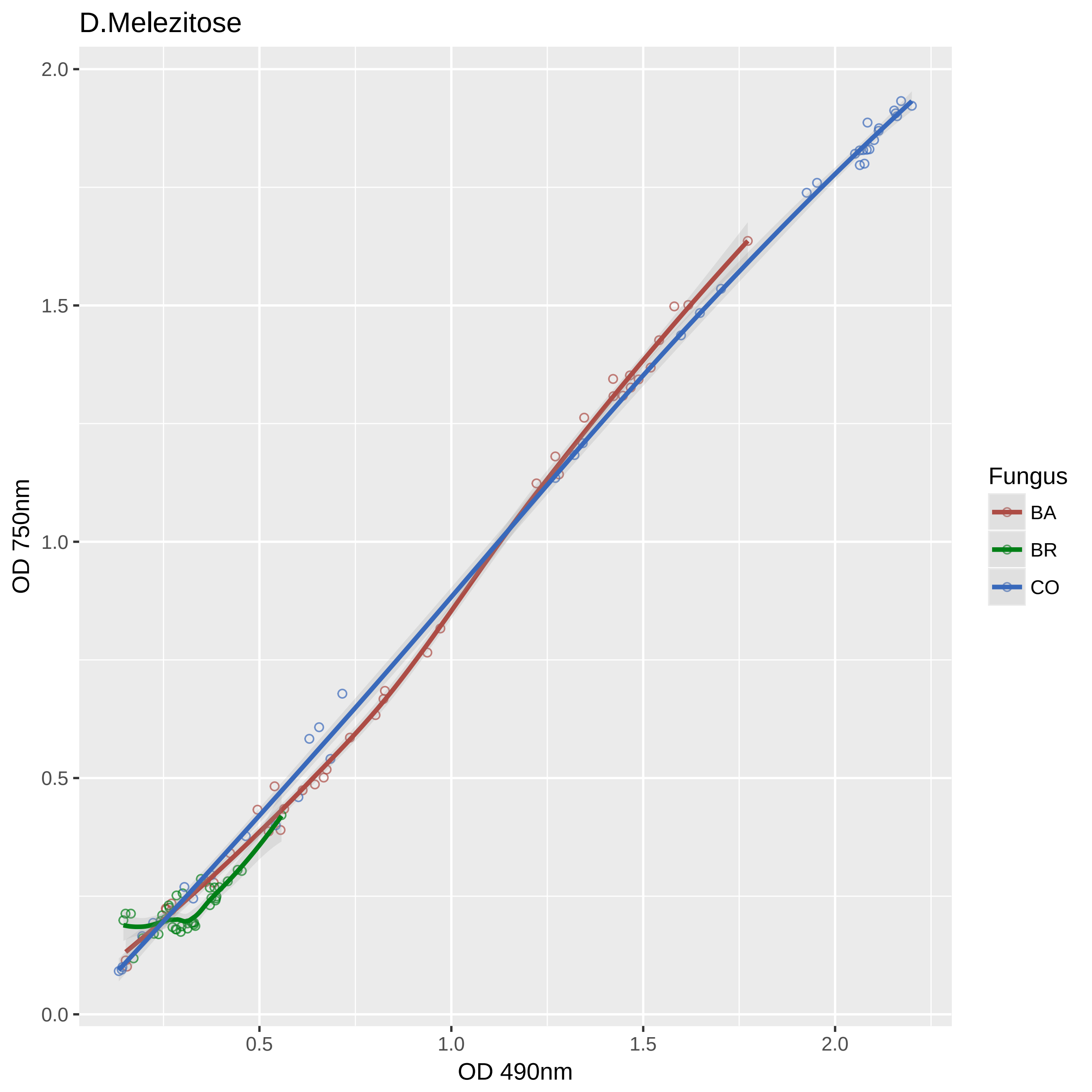
D.Melezitose
2.0
1.5
1.0
0.5
0.0
0.5
1.0
1.5
2.0
OD 490nm
OD 750nm
Fungus
BA
BR
CO

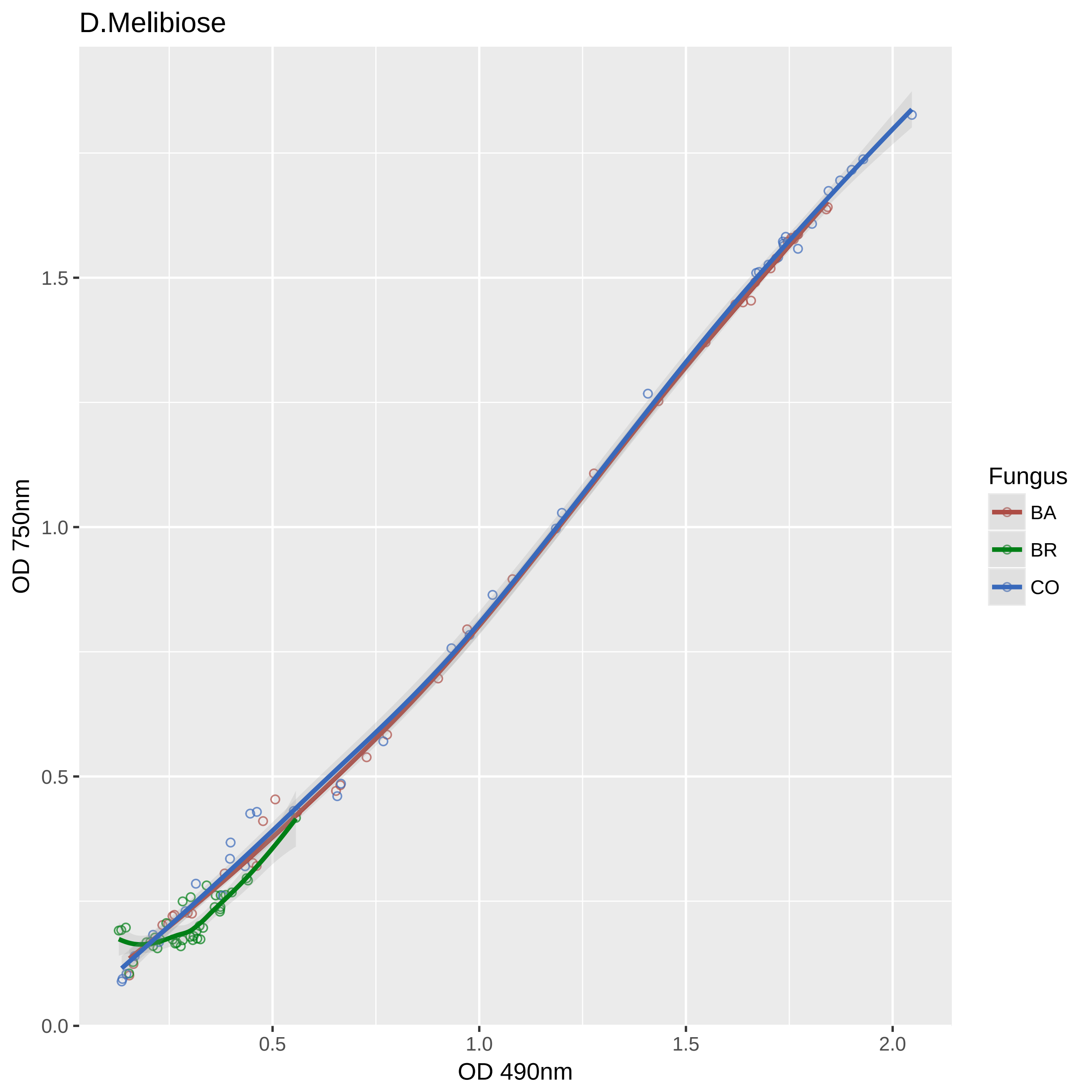

D.Melibiose
OD 750nm
OD 490nm
0.0
0.5
1.0
1.5
0.5
1.0
1.5
2.0
Fungus
BA
BR
CO

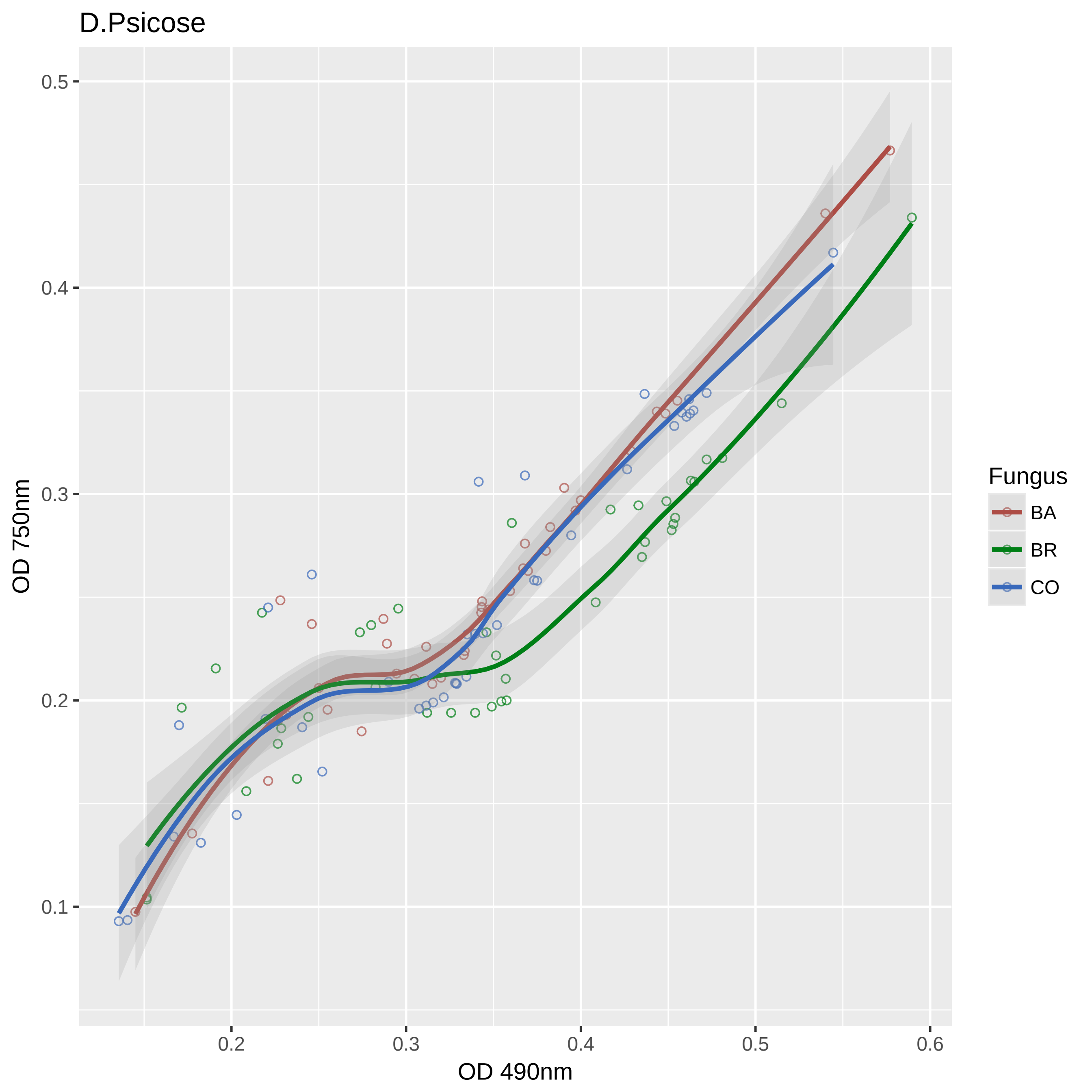

D.Psicose
OD 750nm
0.1
0.2
0.3
0.4
0.5
OD 490nm
0.2
0.3
0.4
0.5
0.6
Fungus
BA
BR
CO

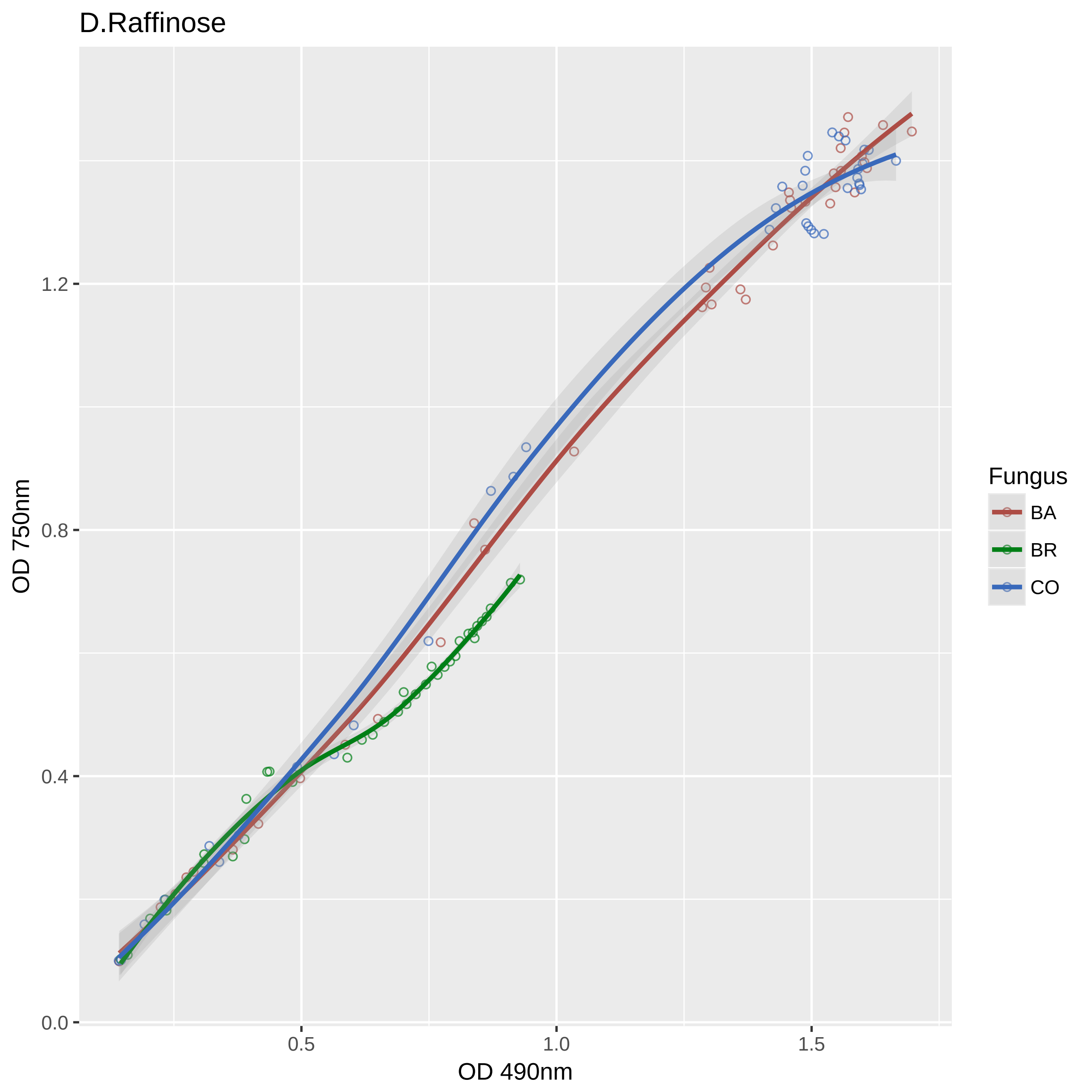

D.Raffinose
OD 750nm
OD 490nm
0.0
0.4
0.8
1.2
0.5
1.0
1.5
Fungus
BA
BR
CO

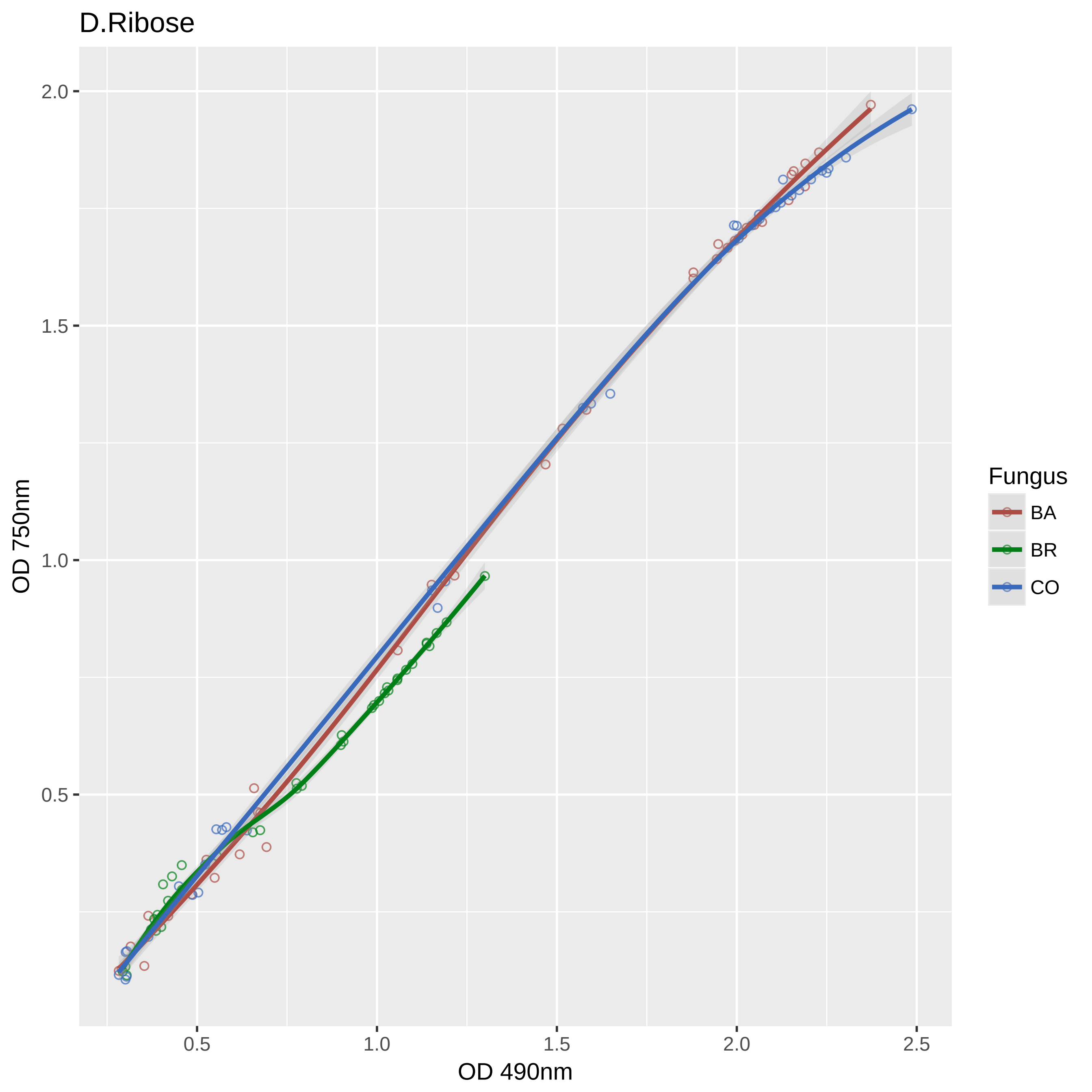

D.Ribose
OD 750nm
OD 490nm
2.0
1.5
1.0
0.5
0.5
1.0
1.5
2.0
2.5
Fungus
BA
BR
CO

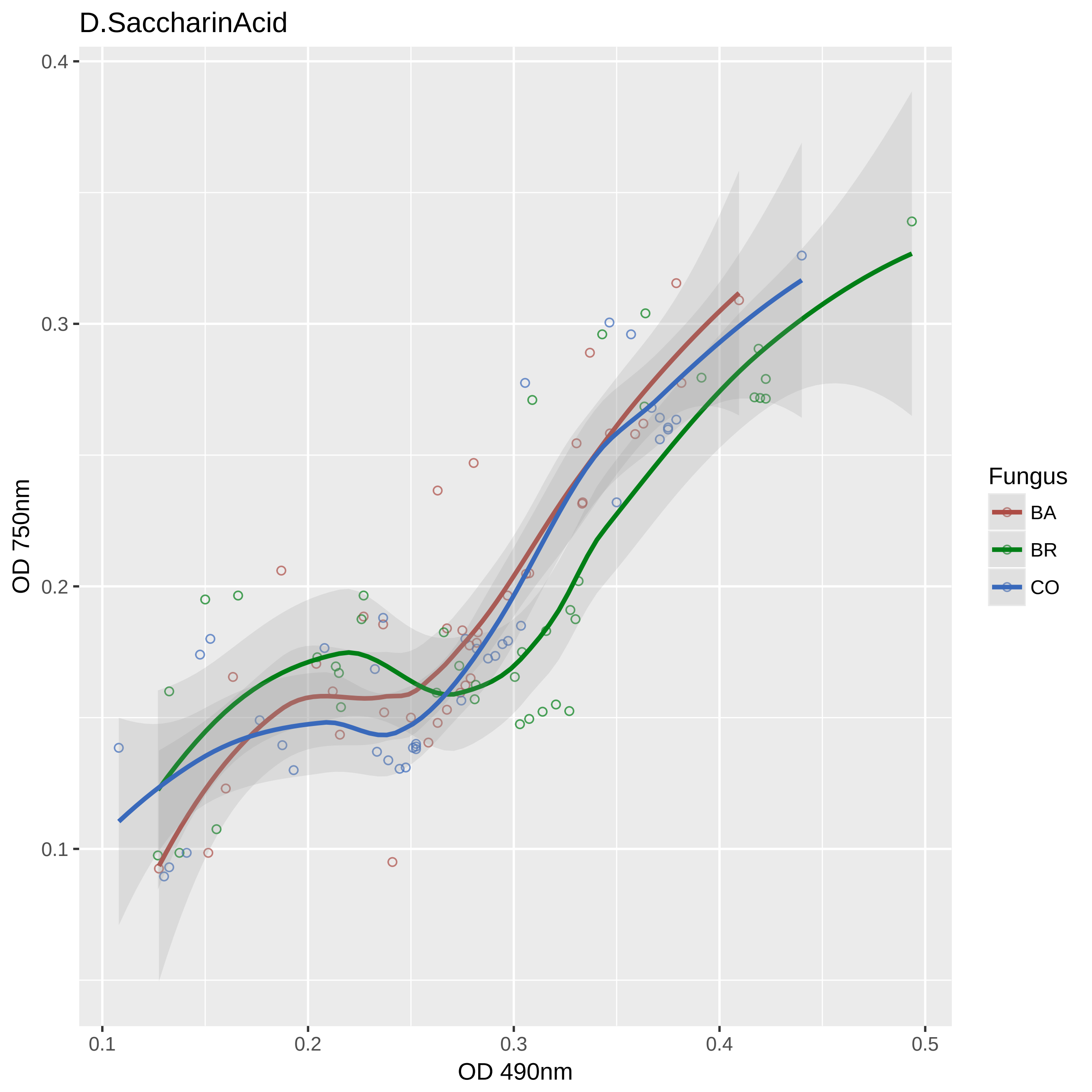
D.SaccharinAcid
OD 750nm
OD 490nm
0.1
0.2
0.3
0.4
0.5
Fungus
BA
BR
CO

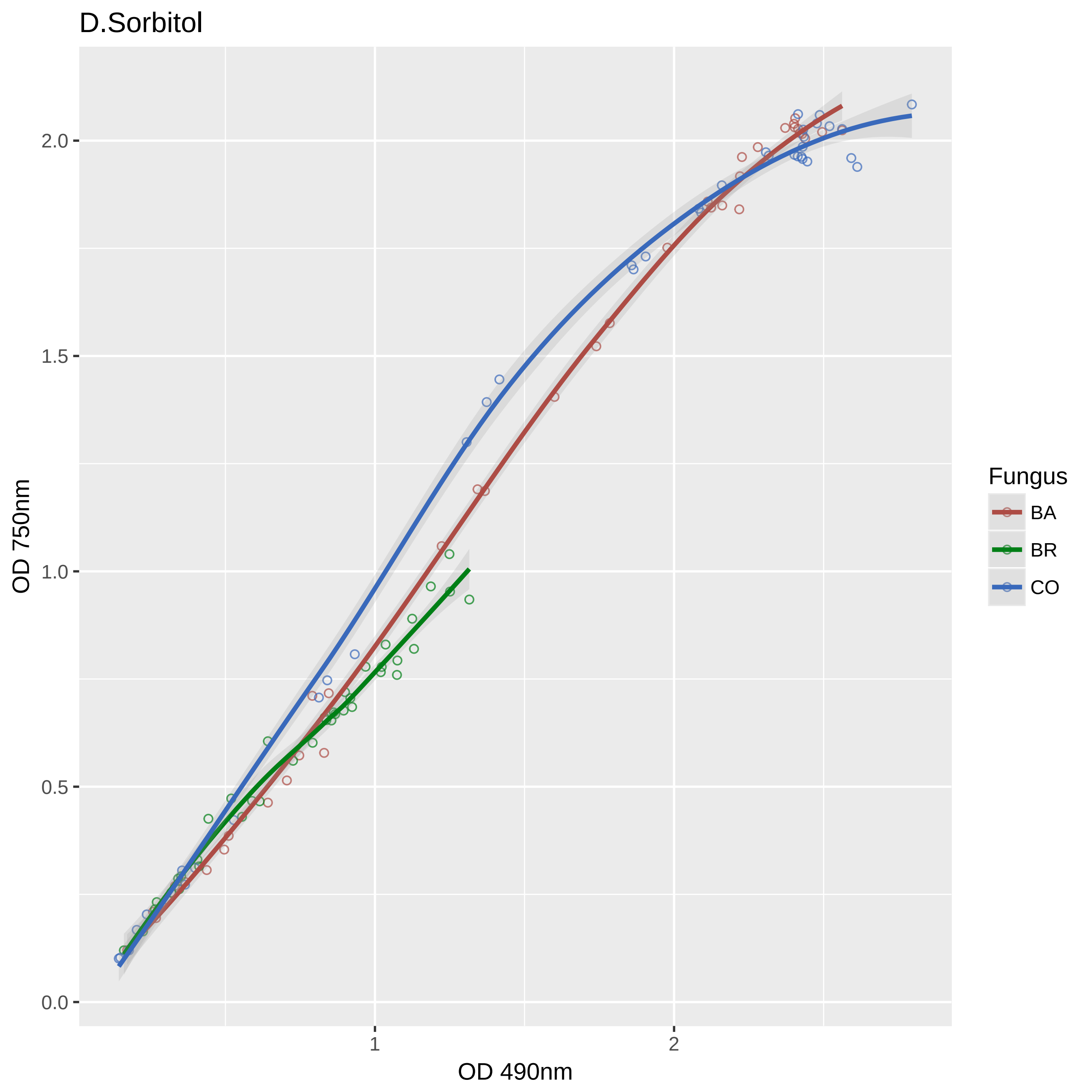

D.Sorbitol
OD 750nm
OD 490nm
0.0
0.5
1.0
1.5
2.0
1
2
Fungus
BA
BR
CO

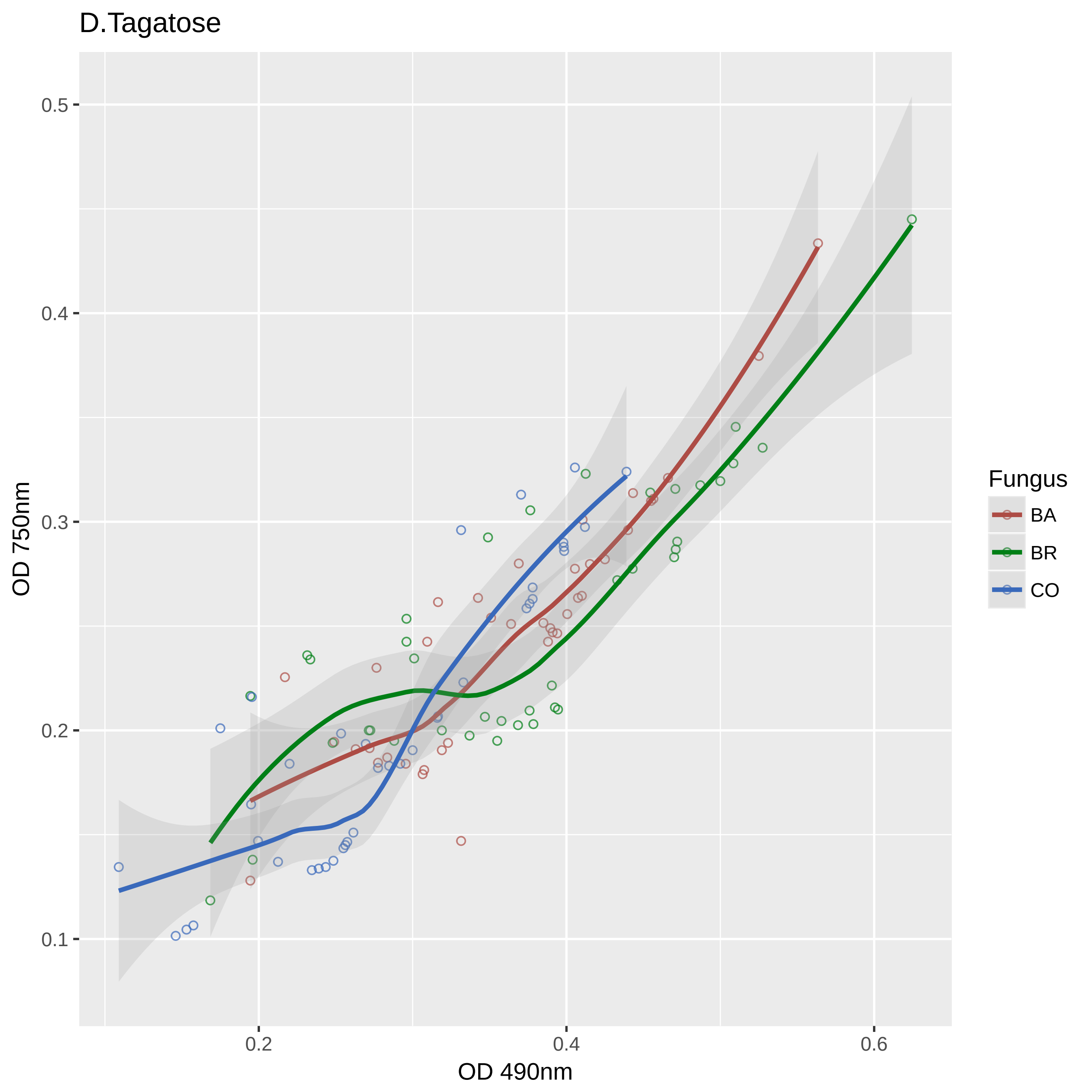

D.Tagatose
OD 750nm
OD 490nm
0.1
0.2
0.3
0.4
0.5
0.2
0.4
0.6
Fungus
BA
BR
CO

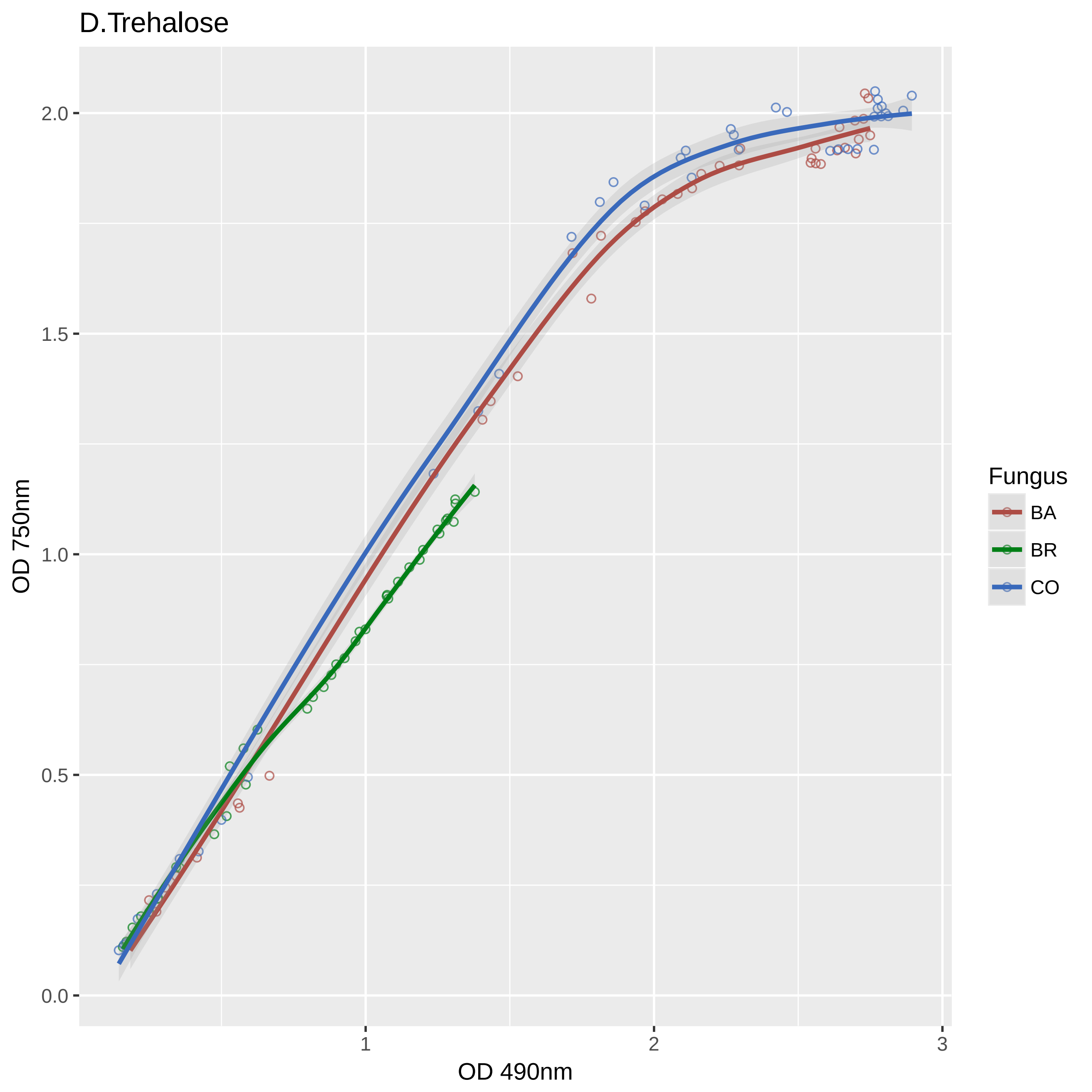

D.Trehalose
OD 750nm
OD 490nm
2.0
1.5
1.0
0.5
0.0
1
2
3
Fungus
BA
BR
CO

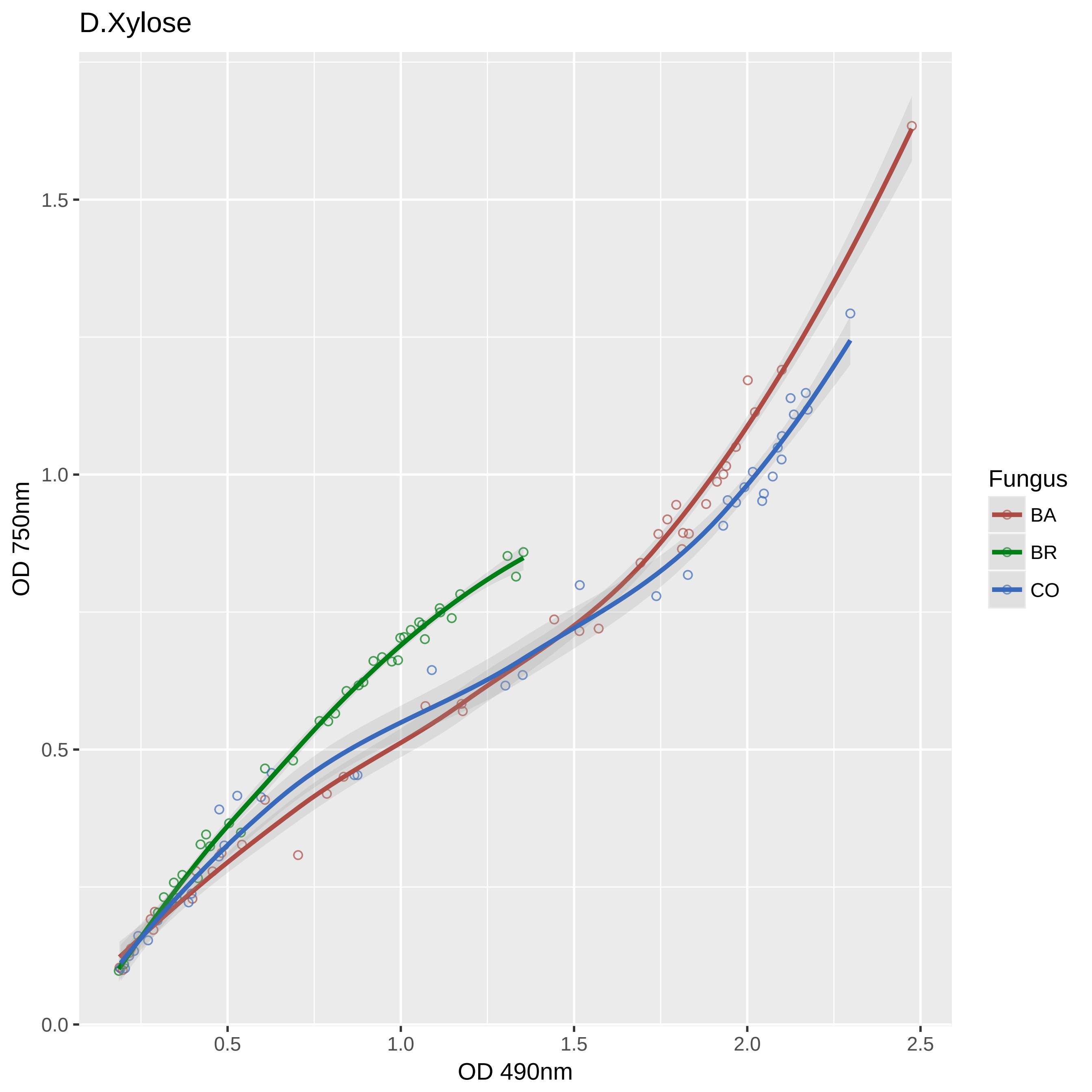
D.Xylose
OD 750nm
OD 490nm
0.0
0.5
1.0
1.5
0.5
1.0
1.5
2.0
2.5
Fungus
BA
BR
CO

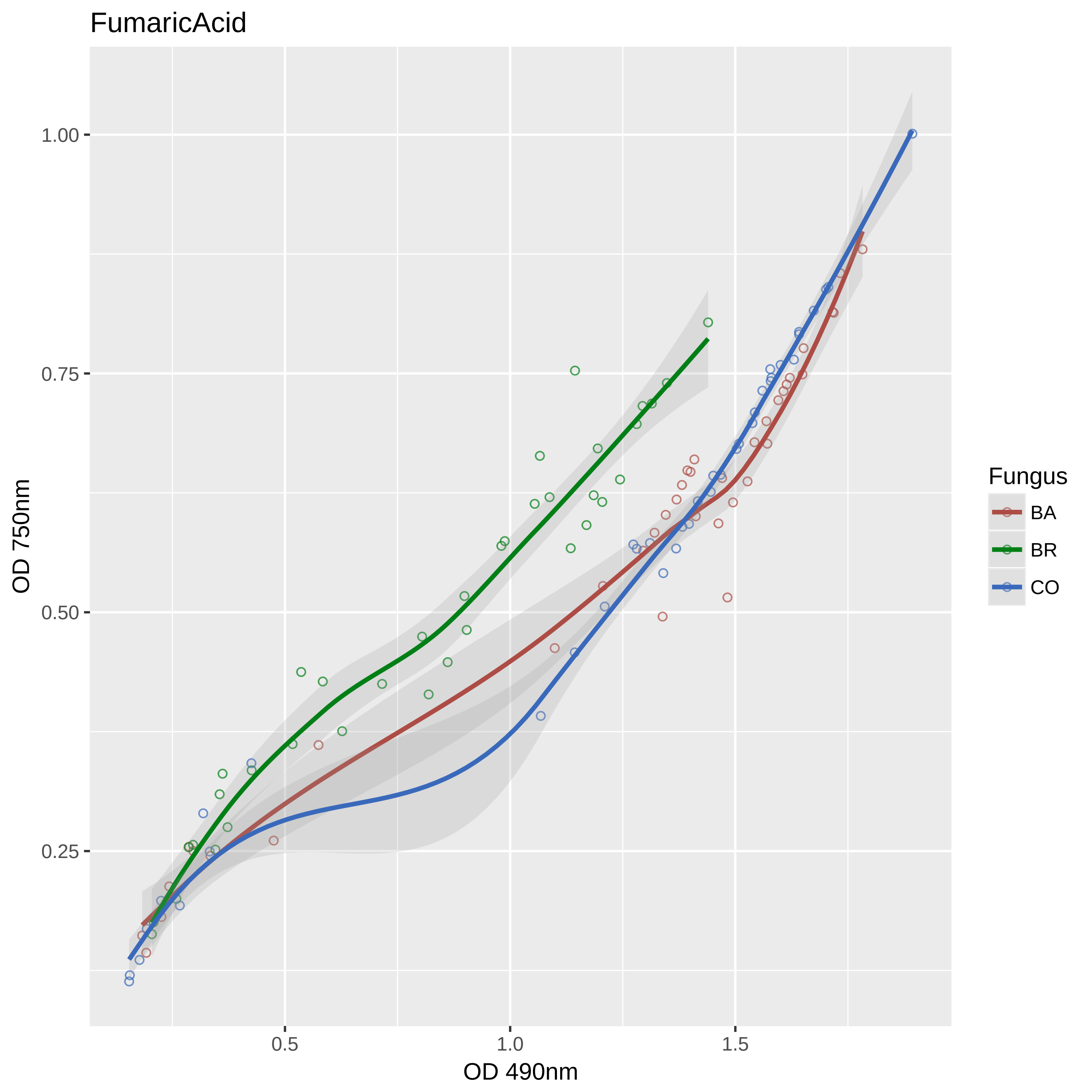
FumaricAcid
OD 750nm
1.00
0.75
0.50
0.25
0.5
1.0
1.5
OD 490nm
Fungus
BA
BR
CO

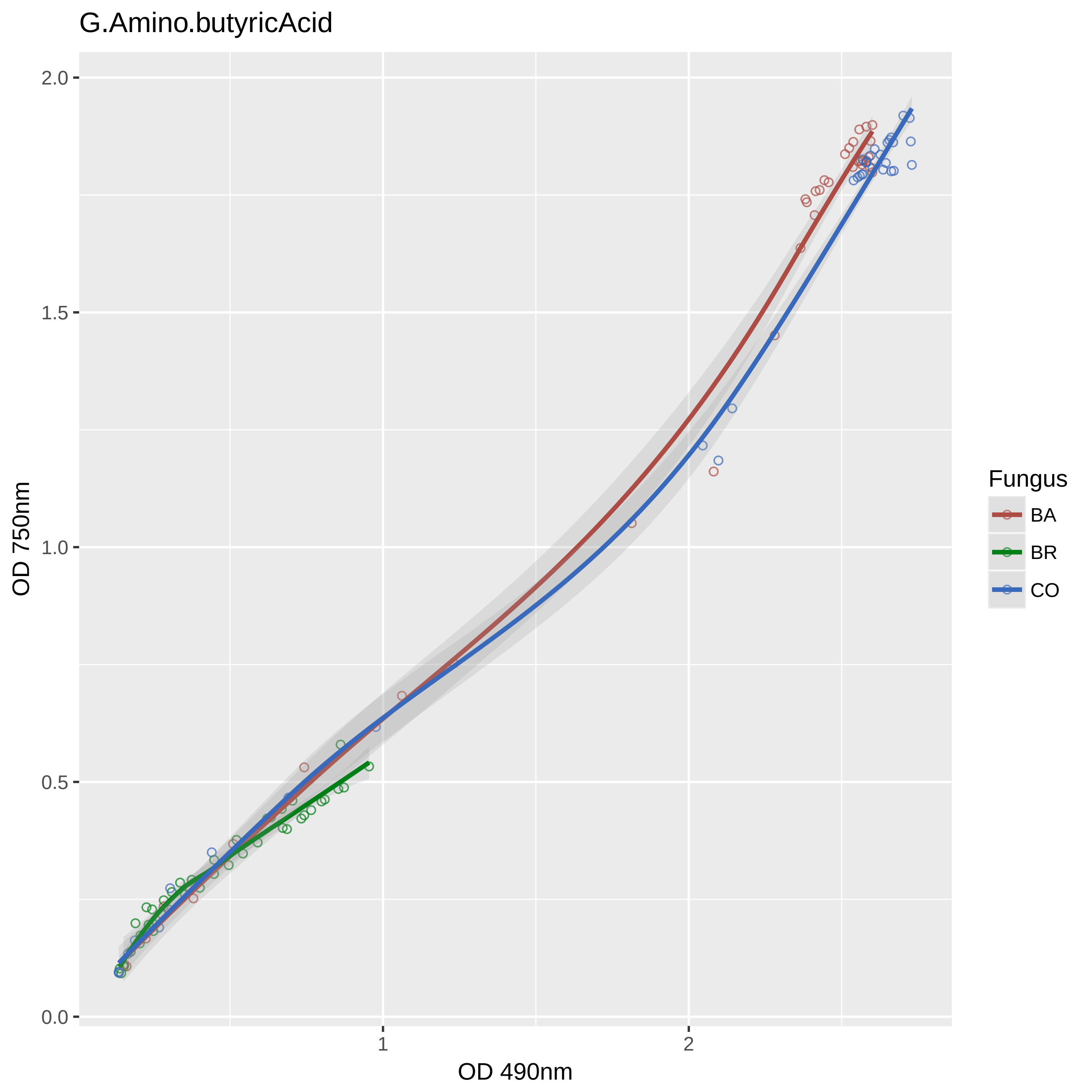

G.Amino.butyricAcid
OD 750nm
OD 490nm
2.0
1.5
1.0
0.5
0.0
1
2
Fungus
BA
BR
CO

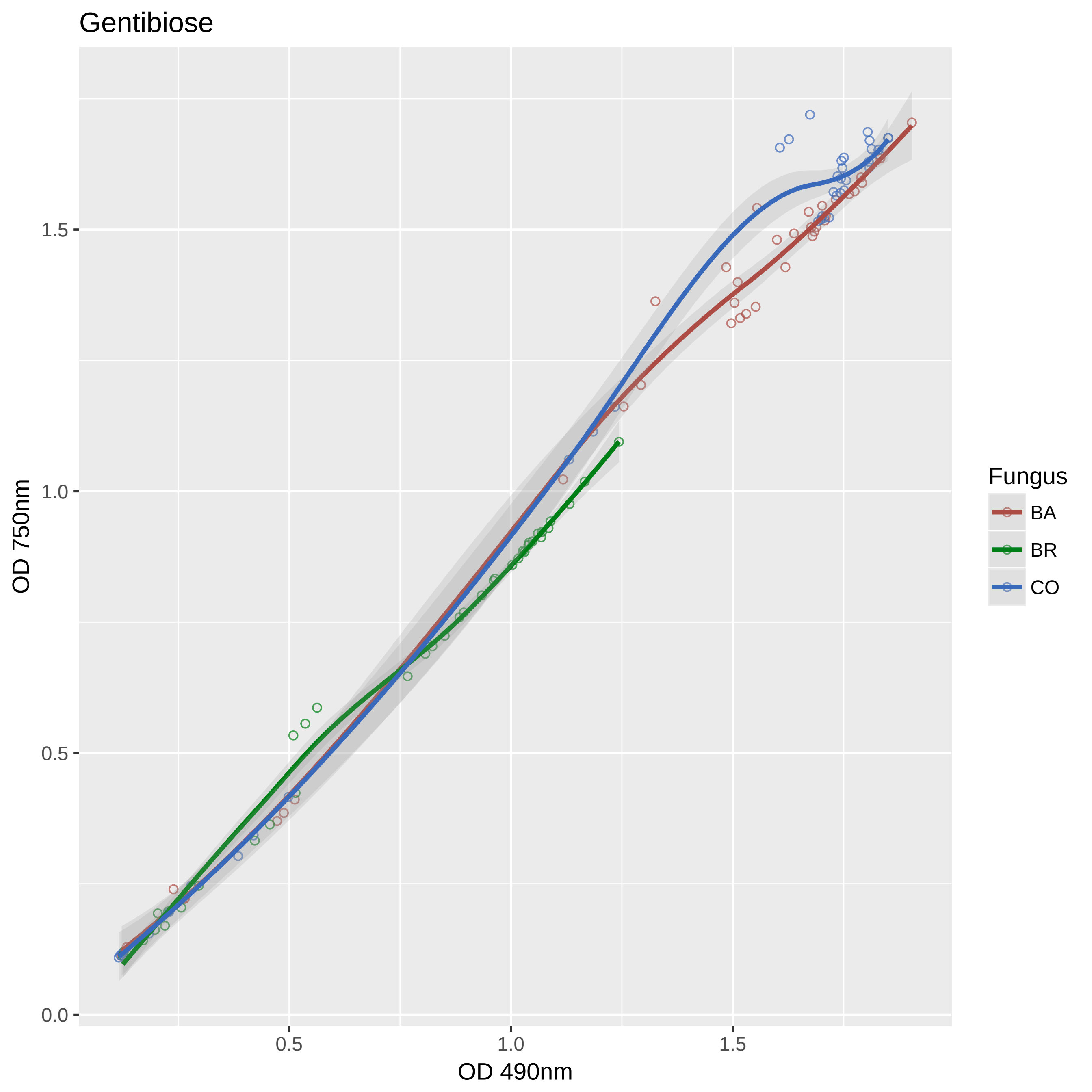
Gentibiose
OD 750nm
OD 490nm
0.0
0.5
1.0
1.5
Fungus
BA
BR
CO

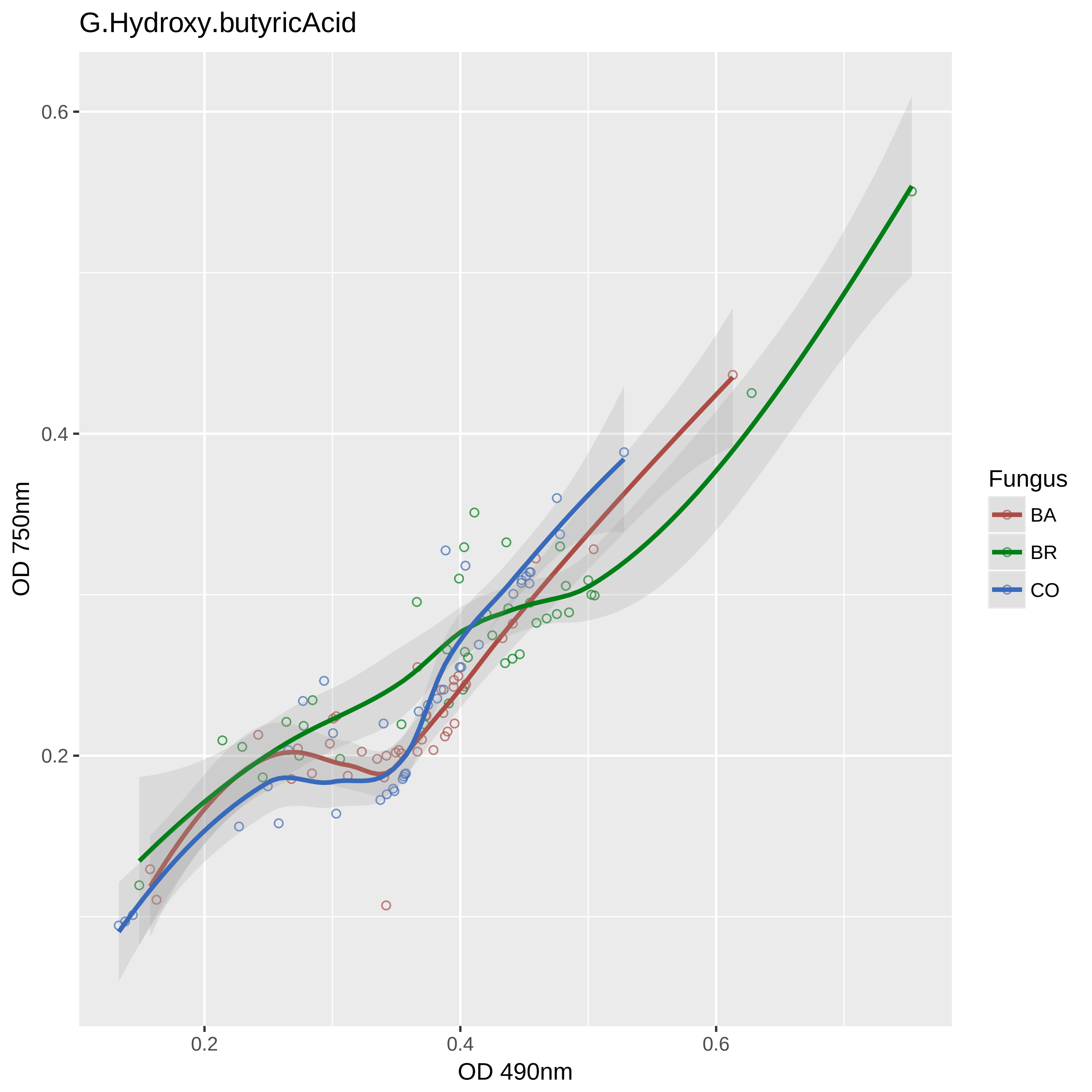

G.Hydroxy.butyricAcid
0.6
0.4
0.2
OD 750nm
0.2
0.4
0.6
OD 490nm
Fungus
BA
BR
CO

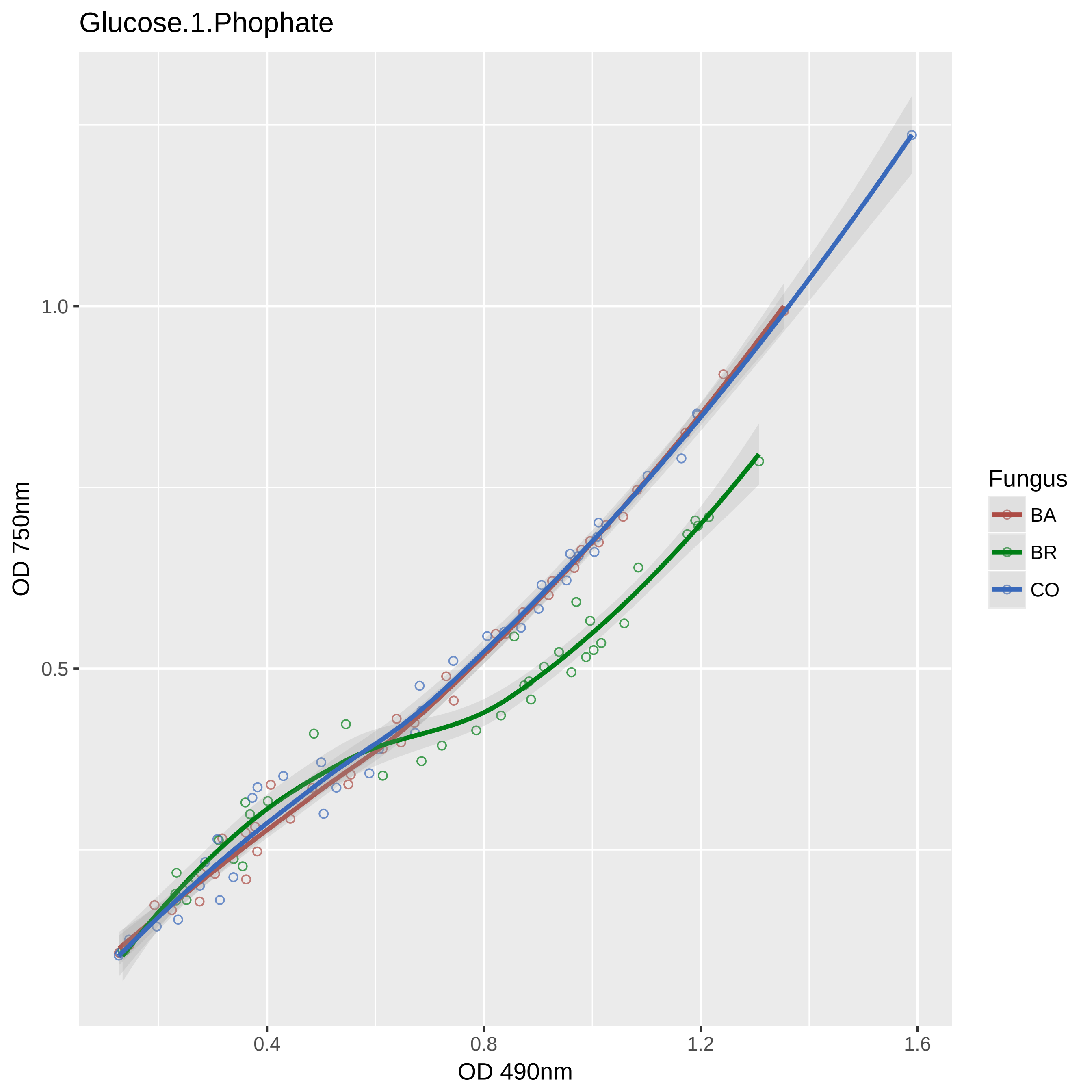

Glucose.1.Phophate
OD 750nm
1.0
0.5
0.4
0.8
1.2
1.6
OD 490nm
Fungus
BA
BR
CO

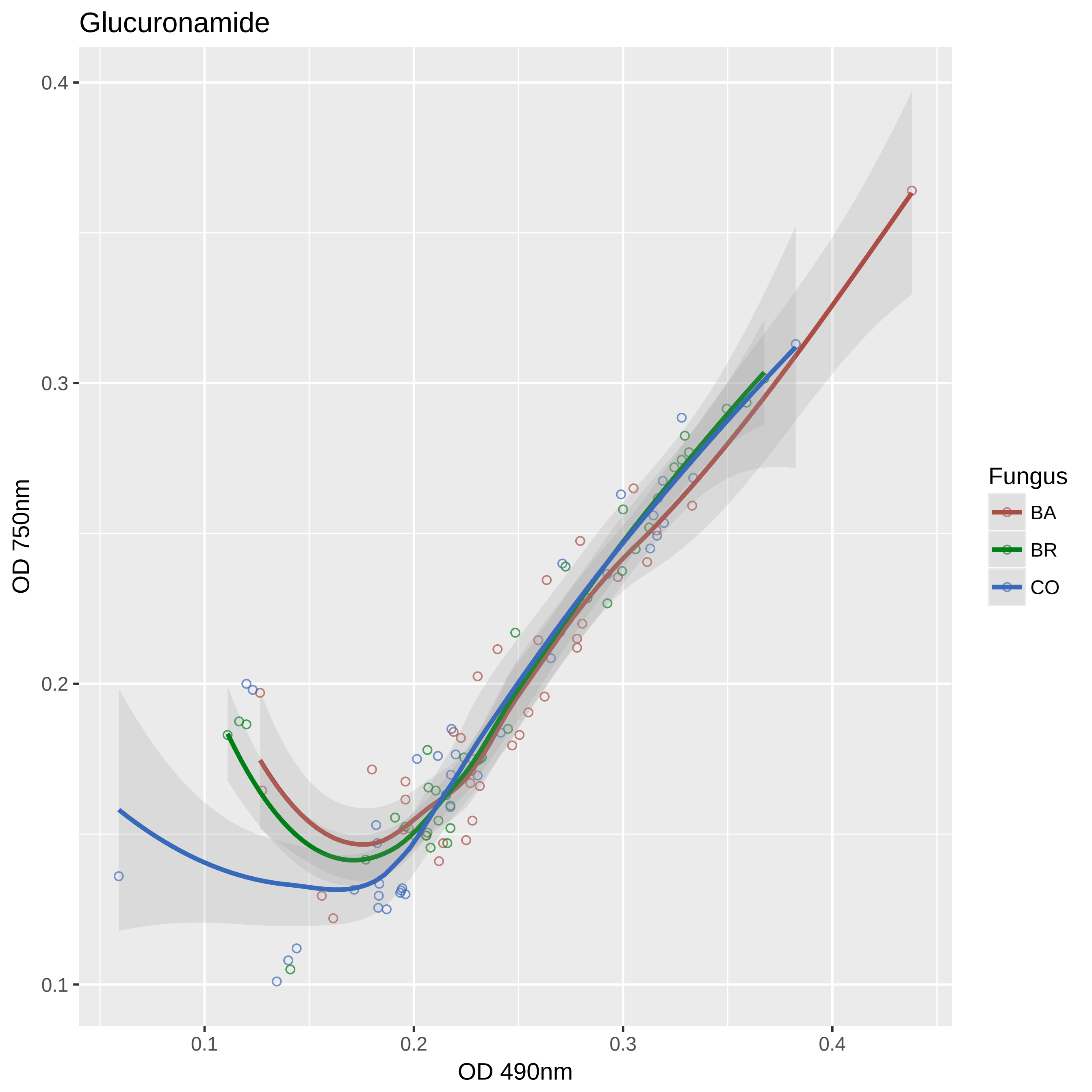

Glucuronamide
OD 750nm
OD 490nm
0.1
0.2
0.3
0.4
0.1
0.2
0.3
0.4
Fungus
BA
BR
CO

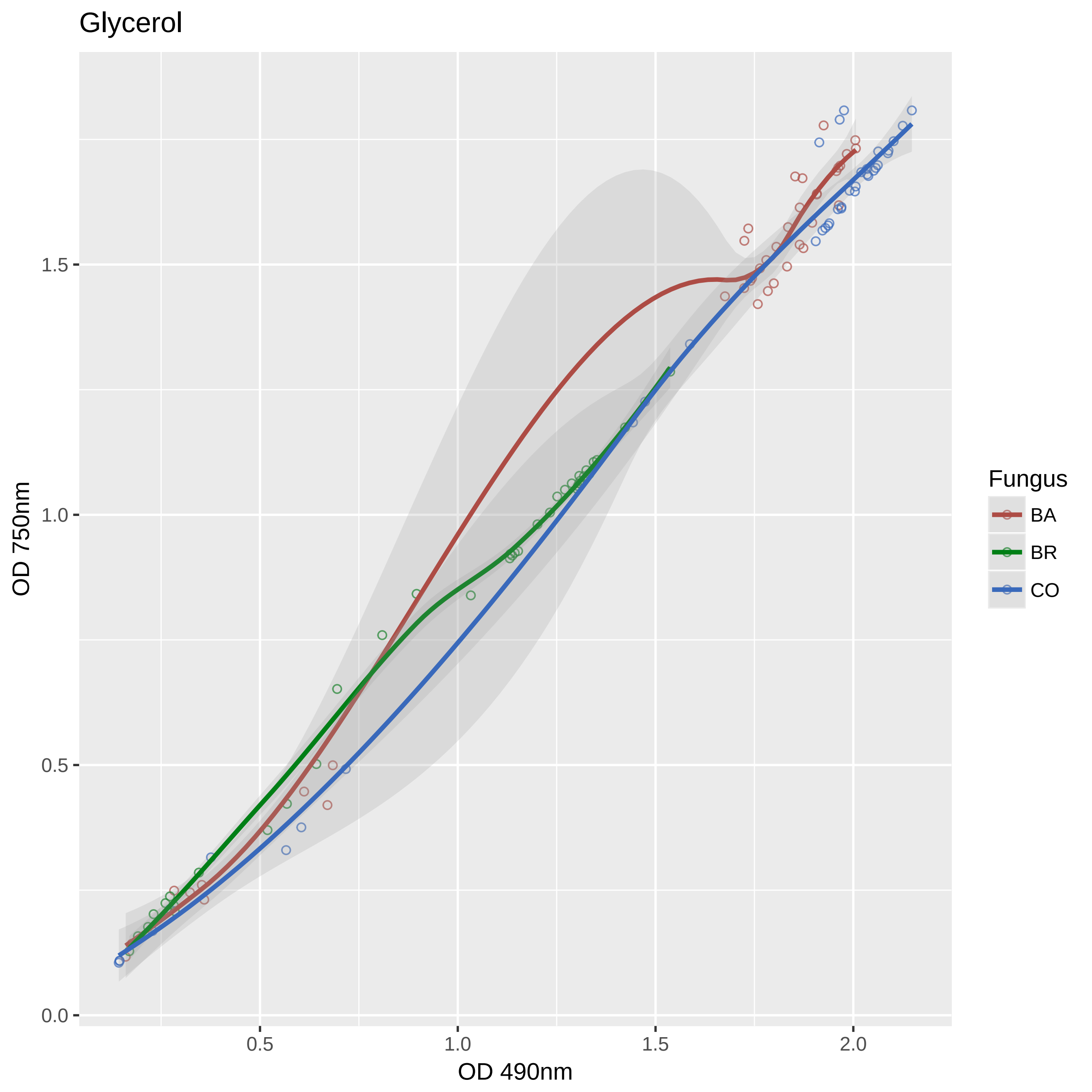
Glycerol
OD 750nm
OD 490nm
0.0
0.5
1.0
1.5
2.0
Fungus
BA
BR
CO

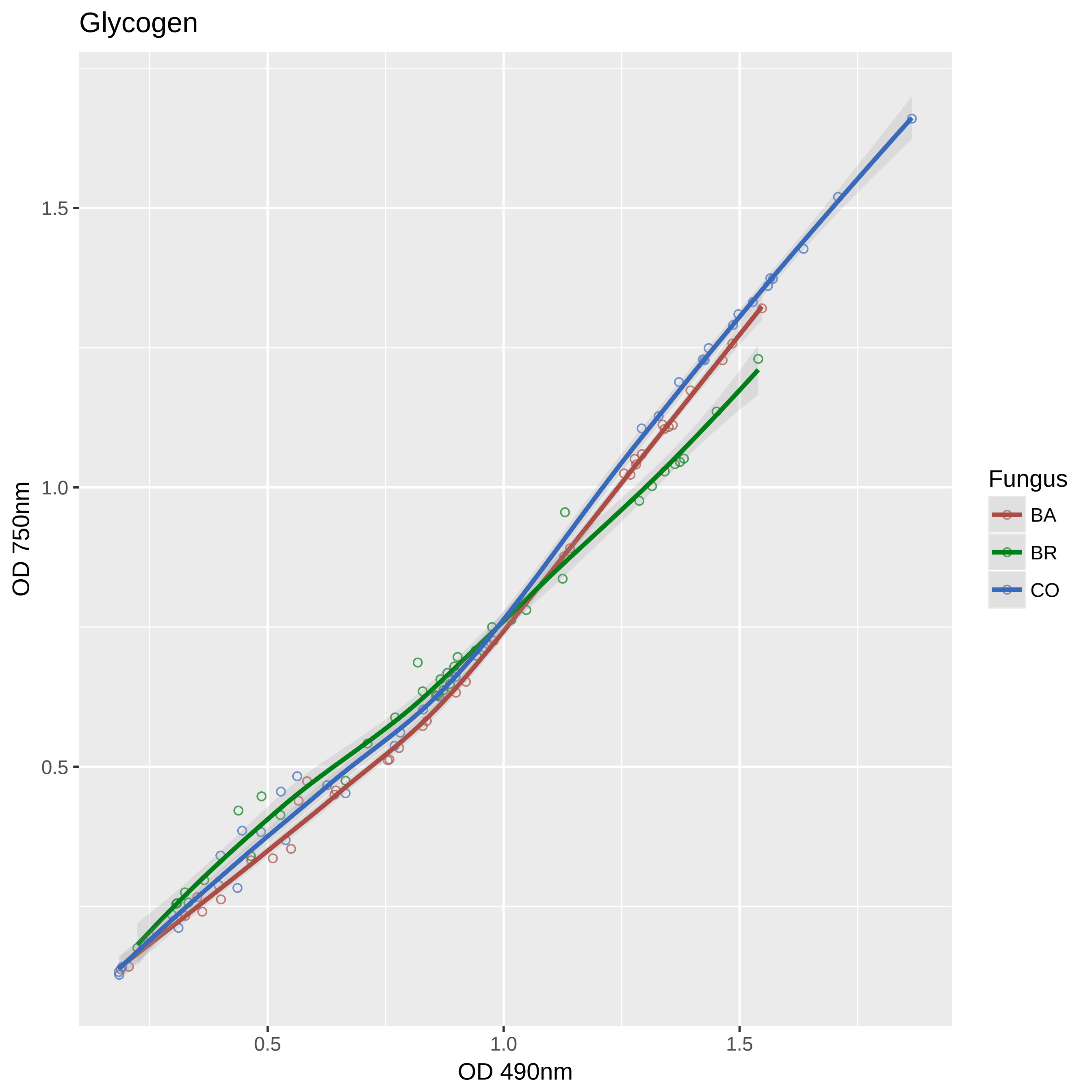

Glycogen
OD 750nm
OD 490nm
1.5
1.0
0.5
0.5
1.0
1.5
Fungus
BA
BR
CO

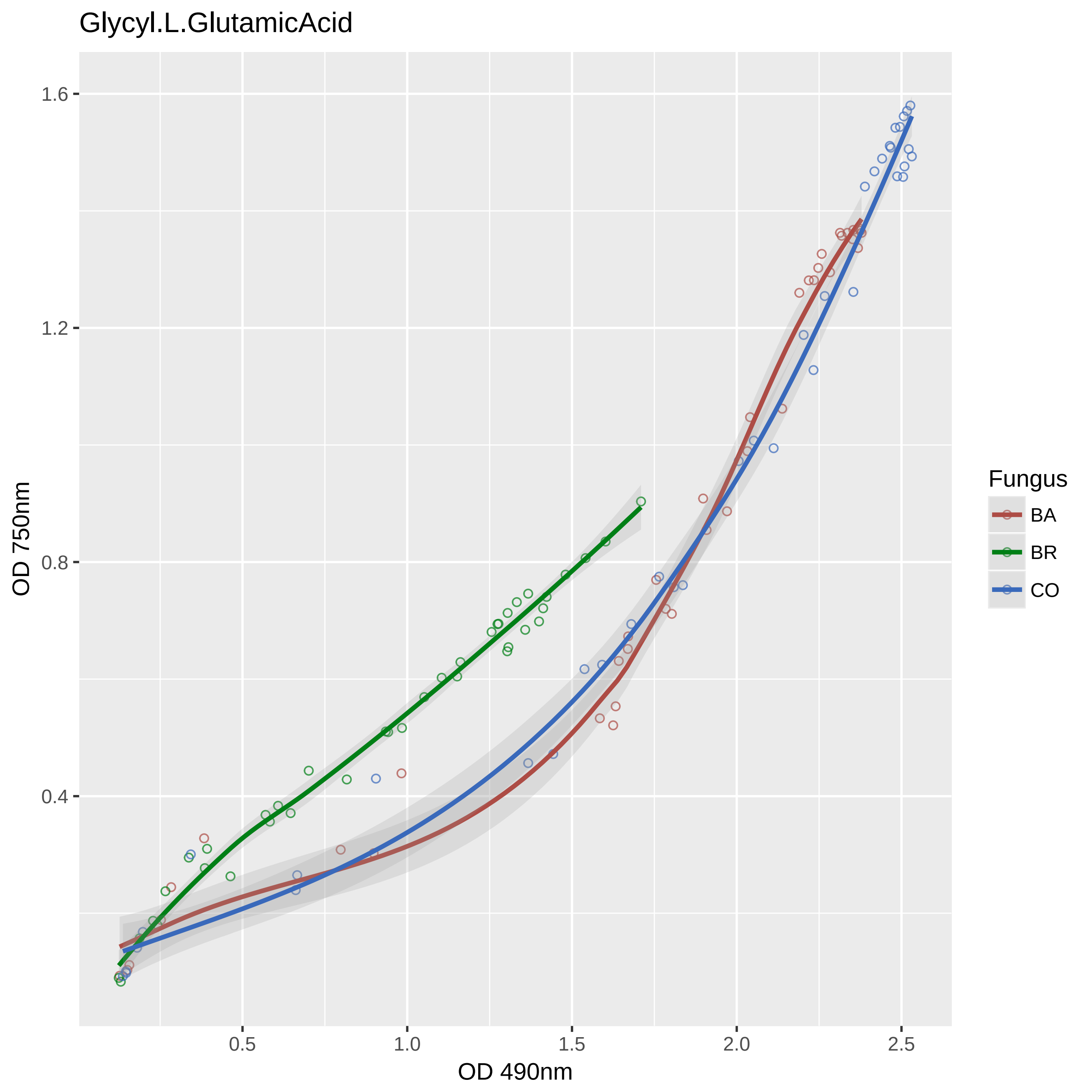
Glycyl.L.GlutamicAcid
OD 750nm
OD 490nm
0.4
0.8
1.2
1.6
0.5
1.0
1.5
2.0
2.5
Fungus
BA
BR
CO

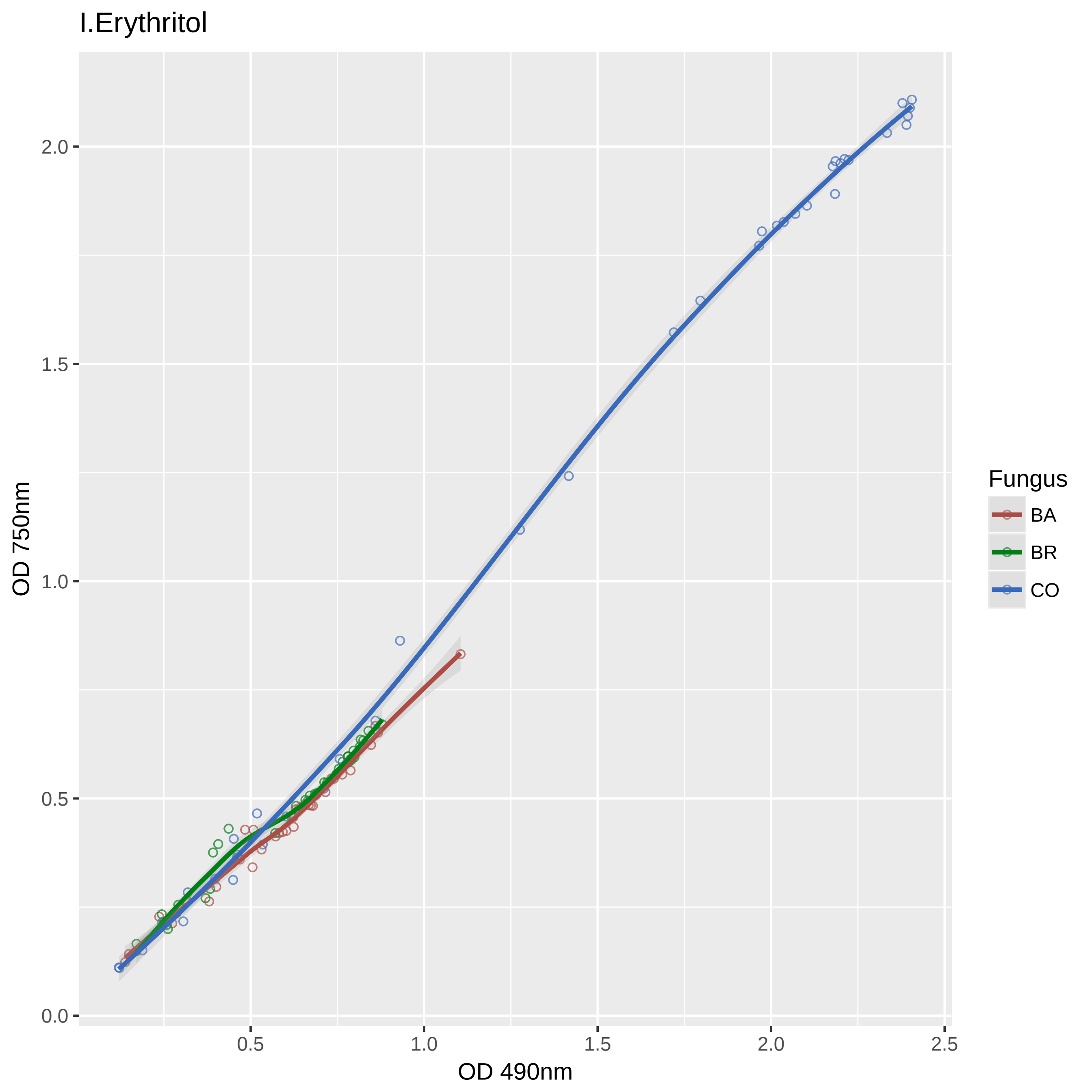
I.Erythritol
OD 750nm
OD 490nm
0.0
0.5
1.0
1.5
2.0
0.5
1.0
1.5
2.0
2.5
Fungus
BA
BR
CO

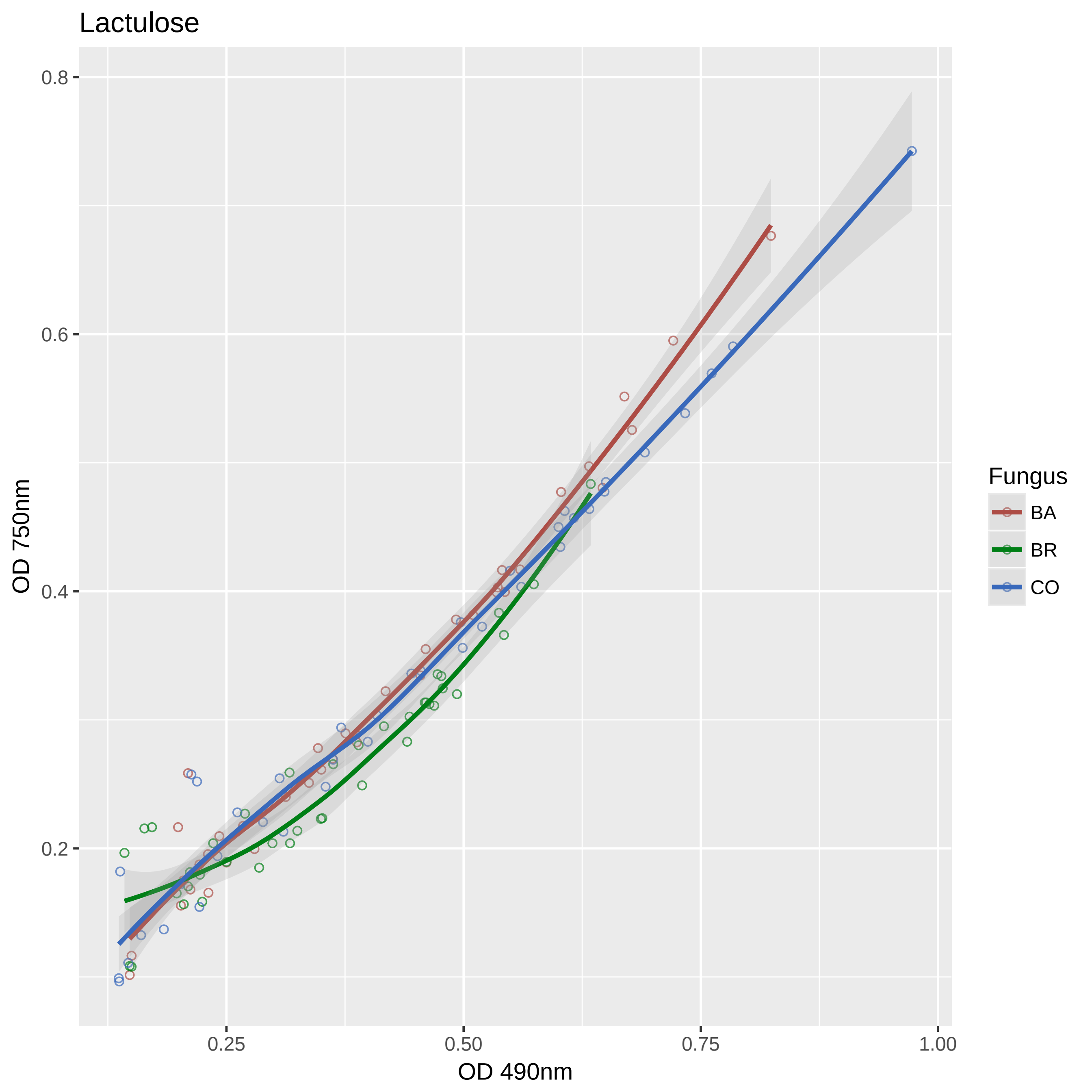
Lactulose
OD 750nm
0.8
0.6
0.4
0.2
0.25
0.50
0.75
1.00
OD 490nm
Fungus
BA
BR
CO

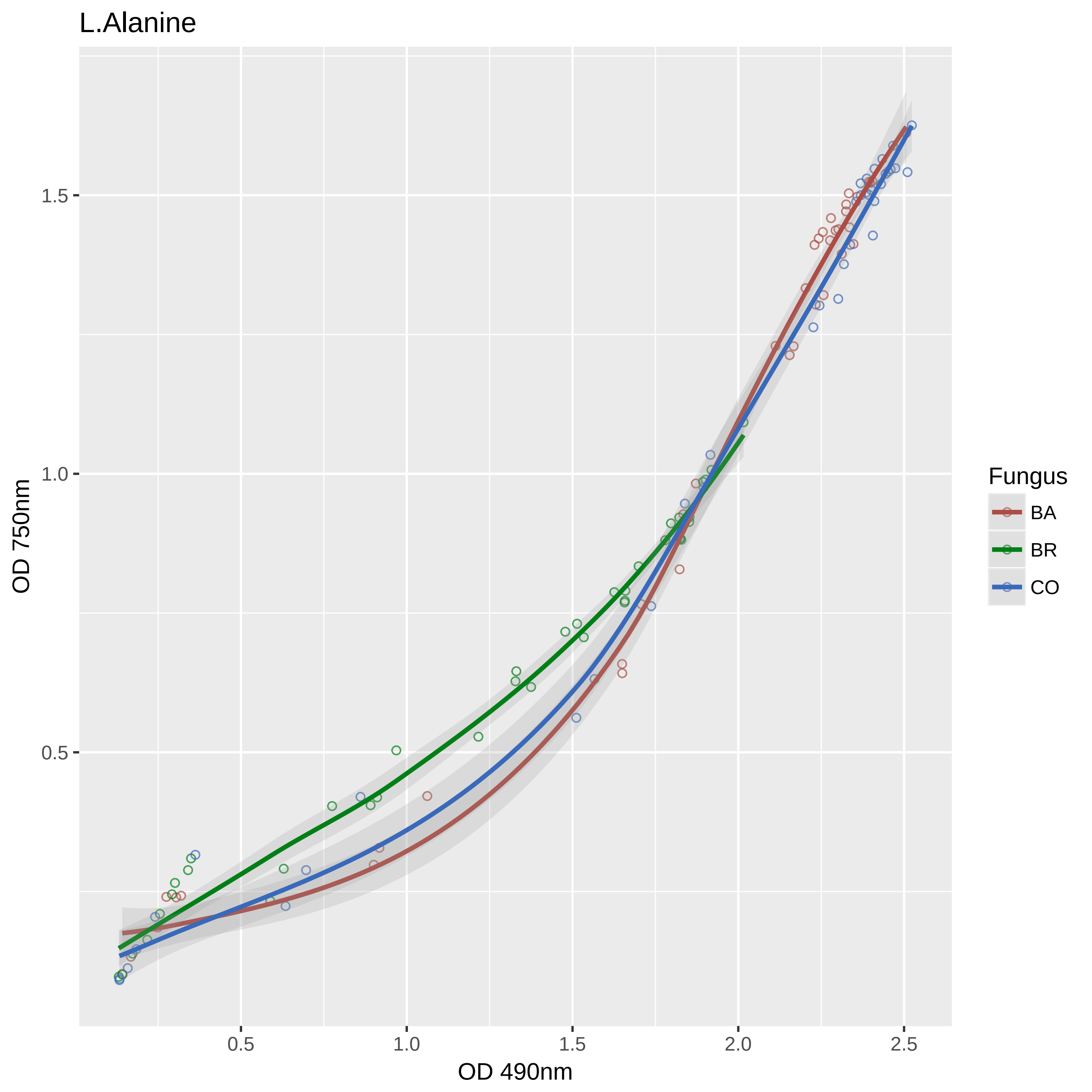

L.Alanine
OD 750nm
1.5
1.0
0.5
OD 490nm
0.5
1.0
1.5
2.0
2.5
Fungus
BA
BR
CO

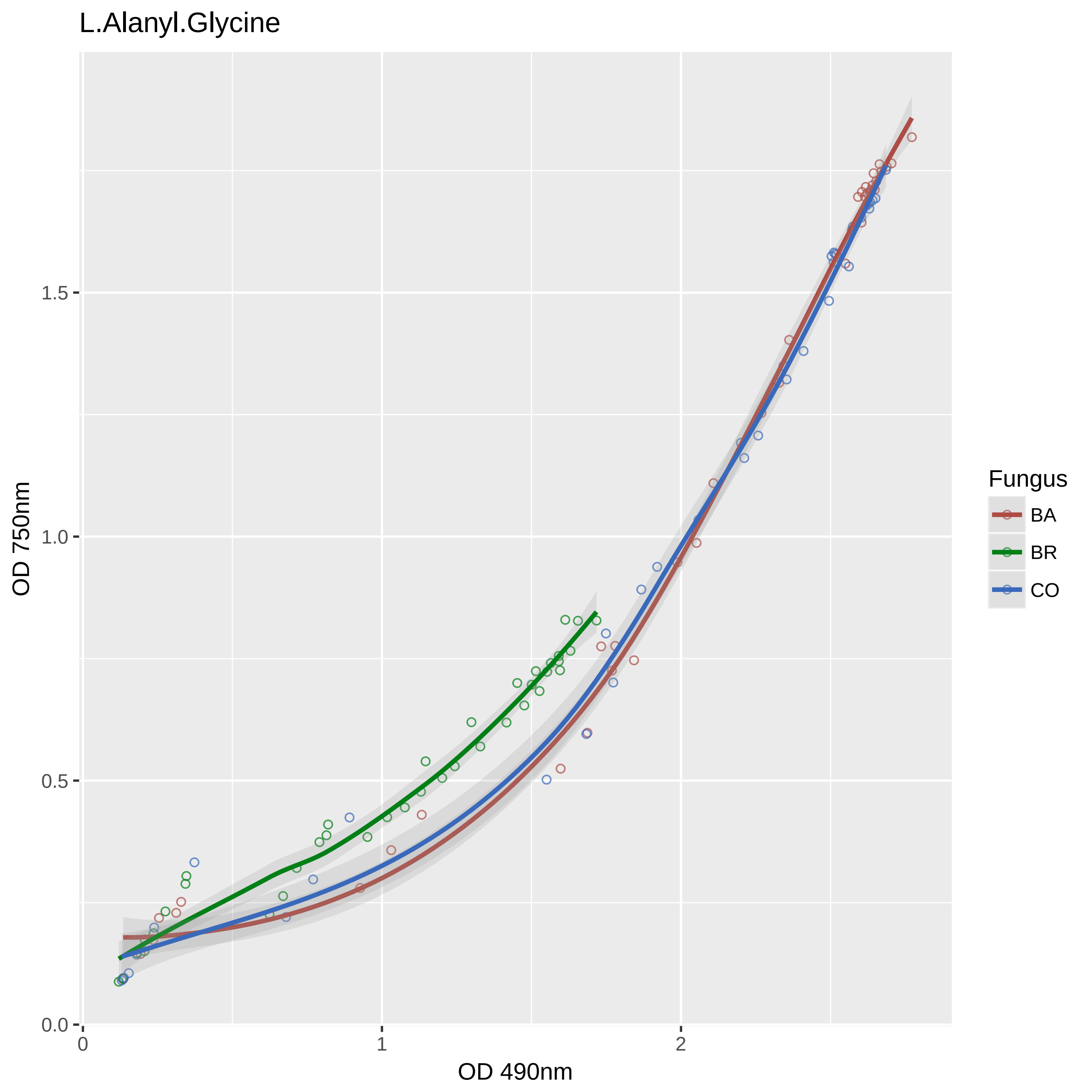

L.Alanyl.Glycine
OD 750nm
0.0
0.5
1.0
1.5
OD 490nm
0
1
2
Fungus
BA
BR
CO

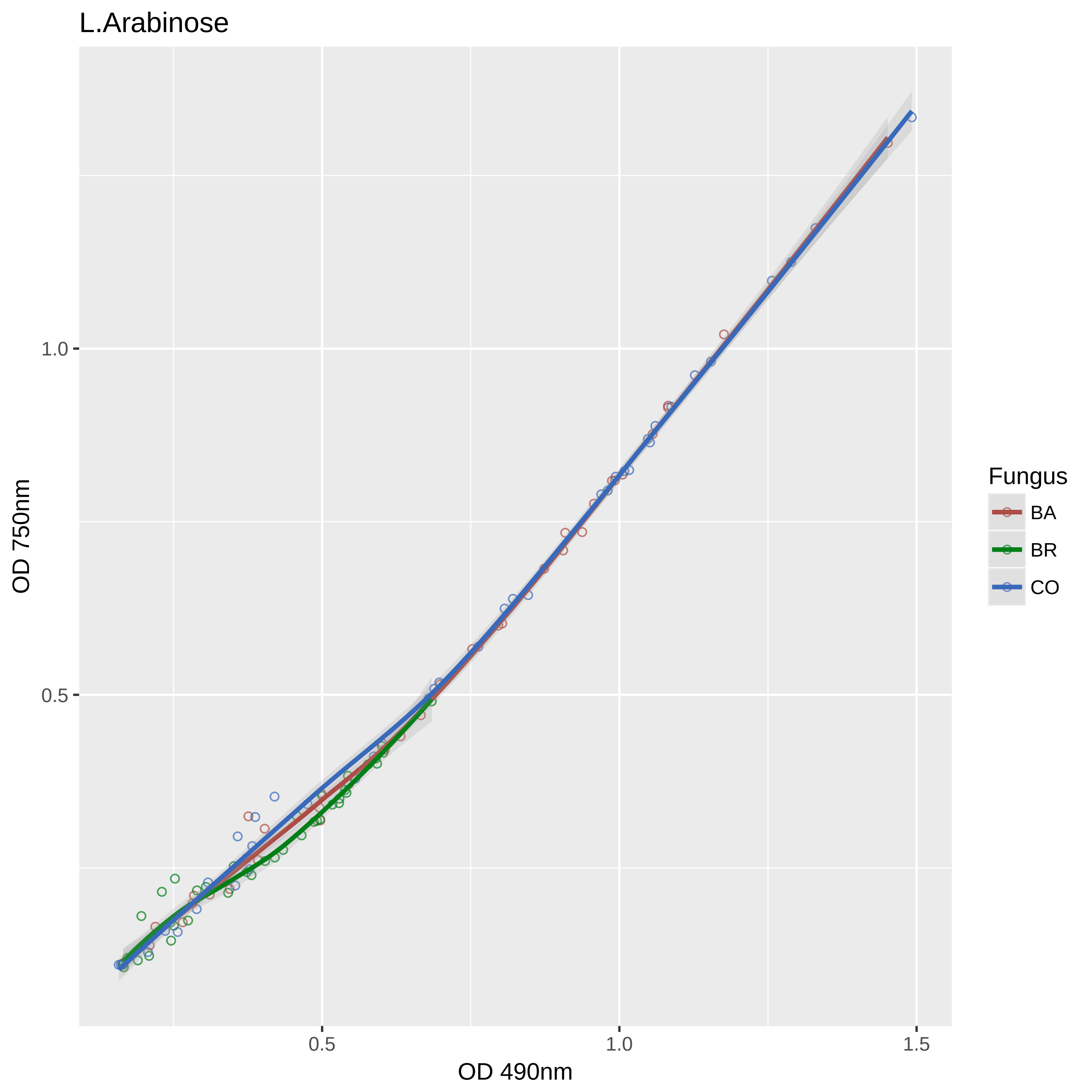
L.Arabinose
OD 750nm
OD 490nm
1.0
0.5
0.5
1.0
1.5
Fungus
BA
BR
CO

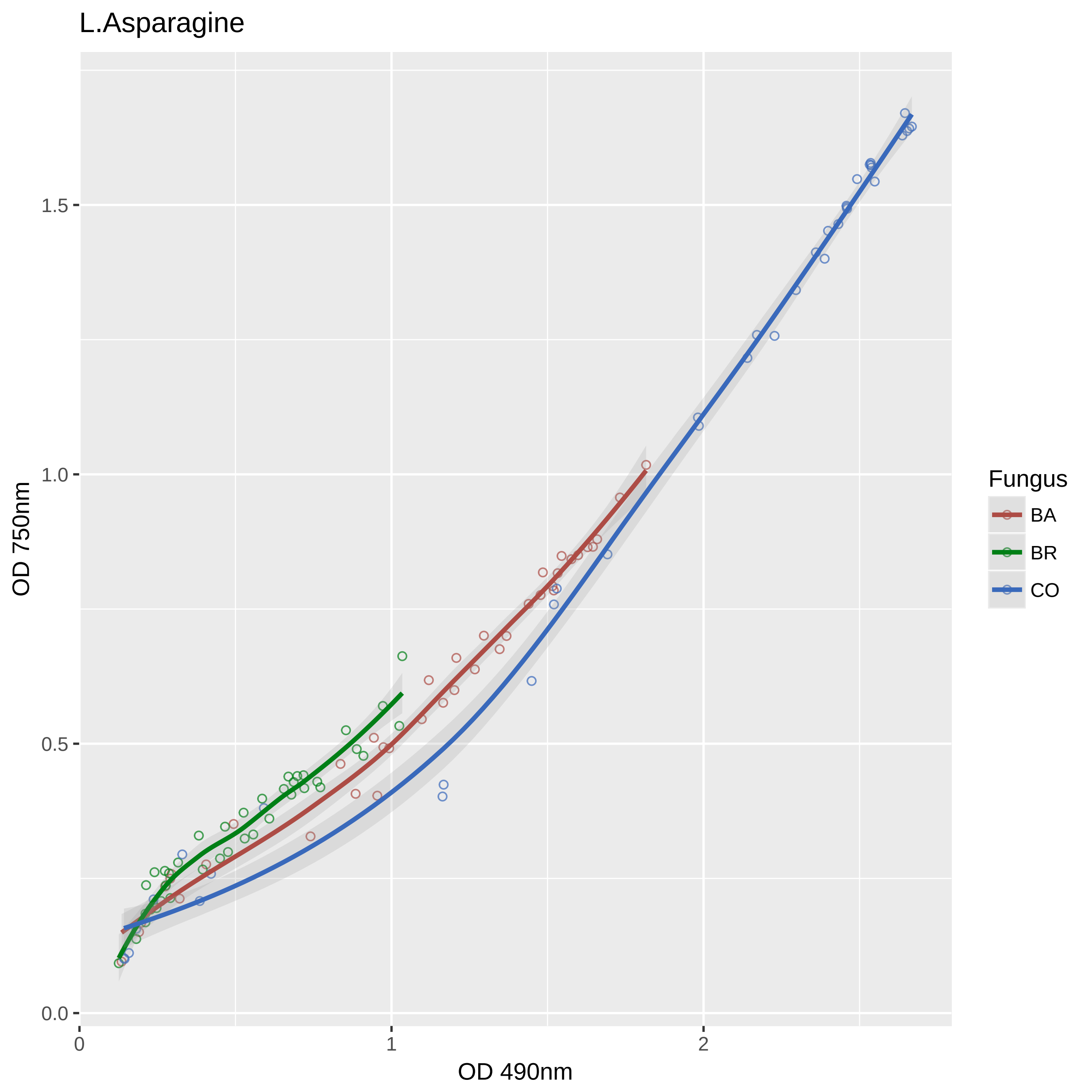
L.Asparagine
OD 750nm
OD 490nm
0.0
0.5
1.0
1.5
0
1
2
Fungus
BA
BR
CO

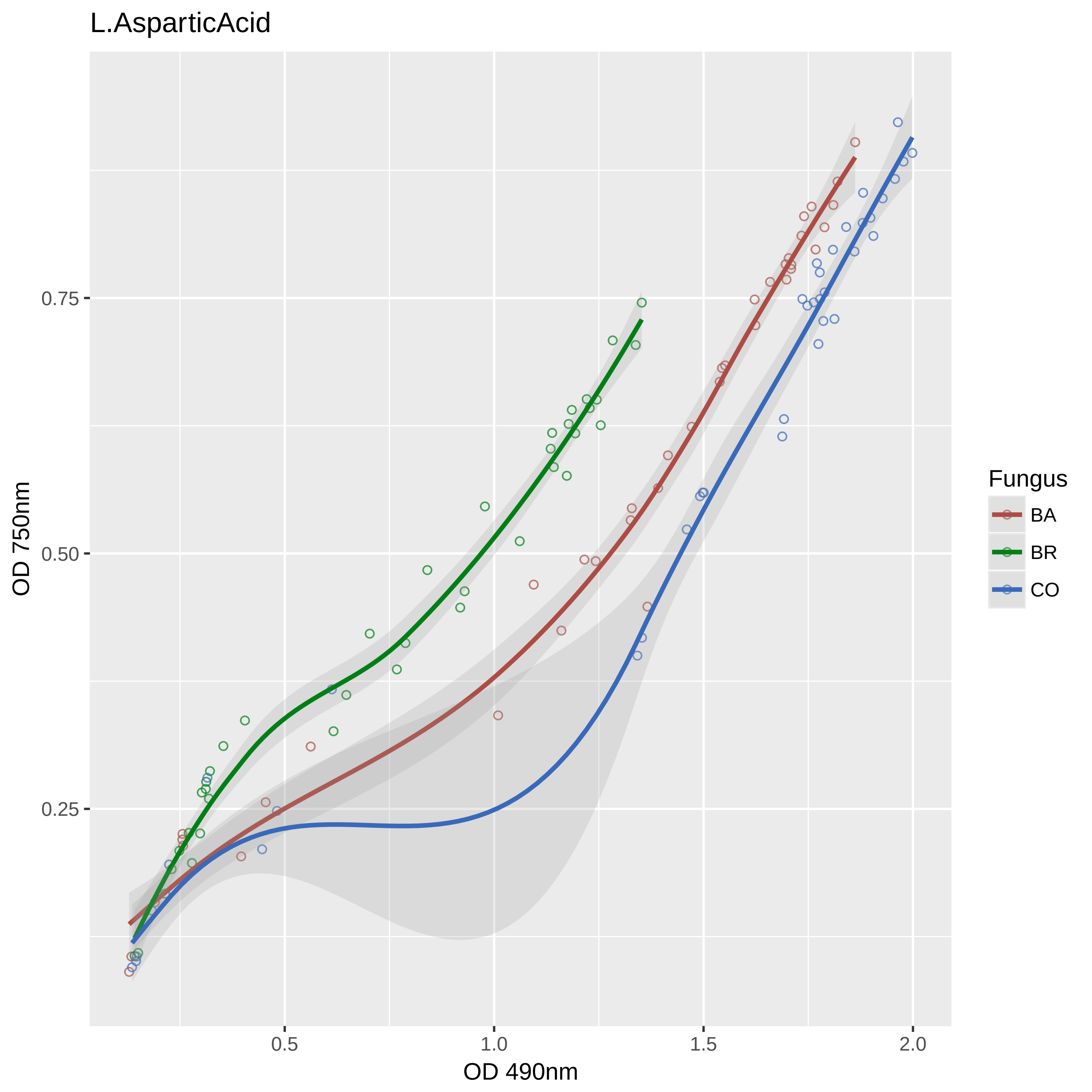

L.AsparticAcid
OD 750nm
0.75
0.50
0.25
OD 490nm
0.5
1.0
1.5
2.0
Fungus
BA
BR
CO

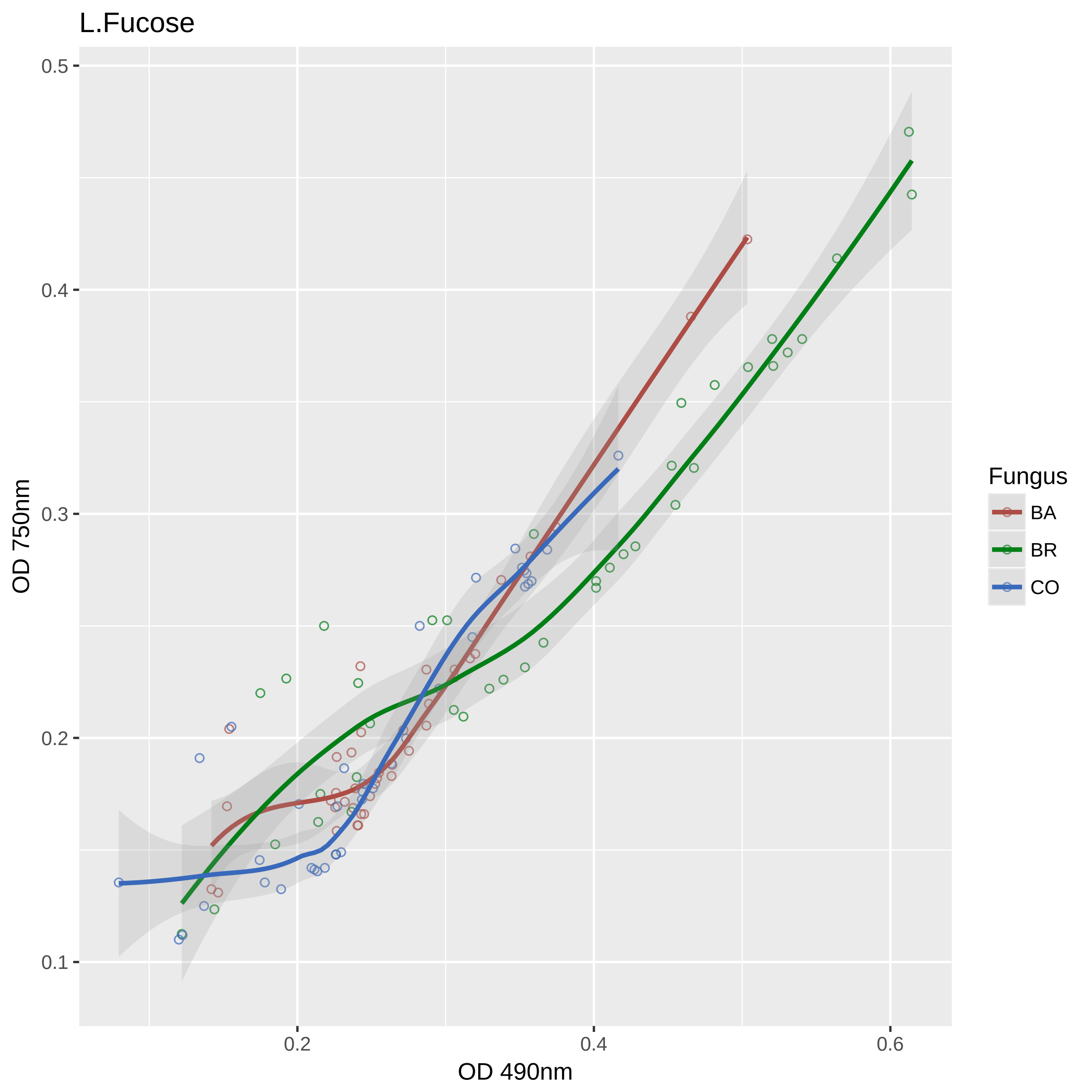

L.Fucose
OD 750nm
OD 490nm
0.1
0.2
0.3
0.4
0.5
0.2
0.4
0.6
Fungus
BA
BR
CO

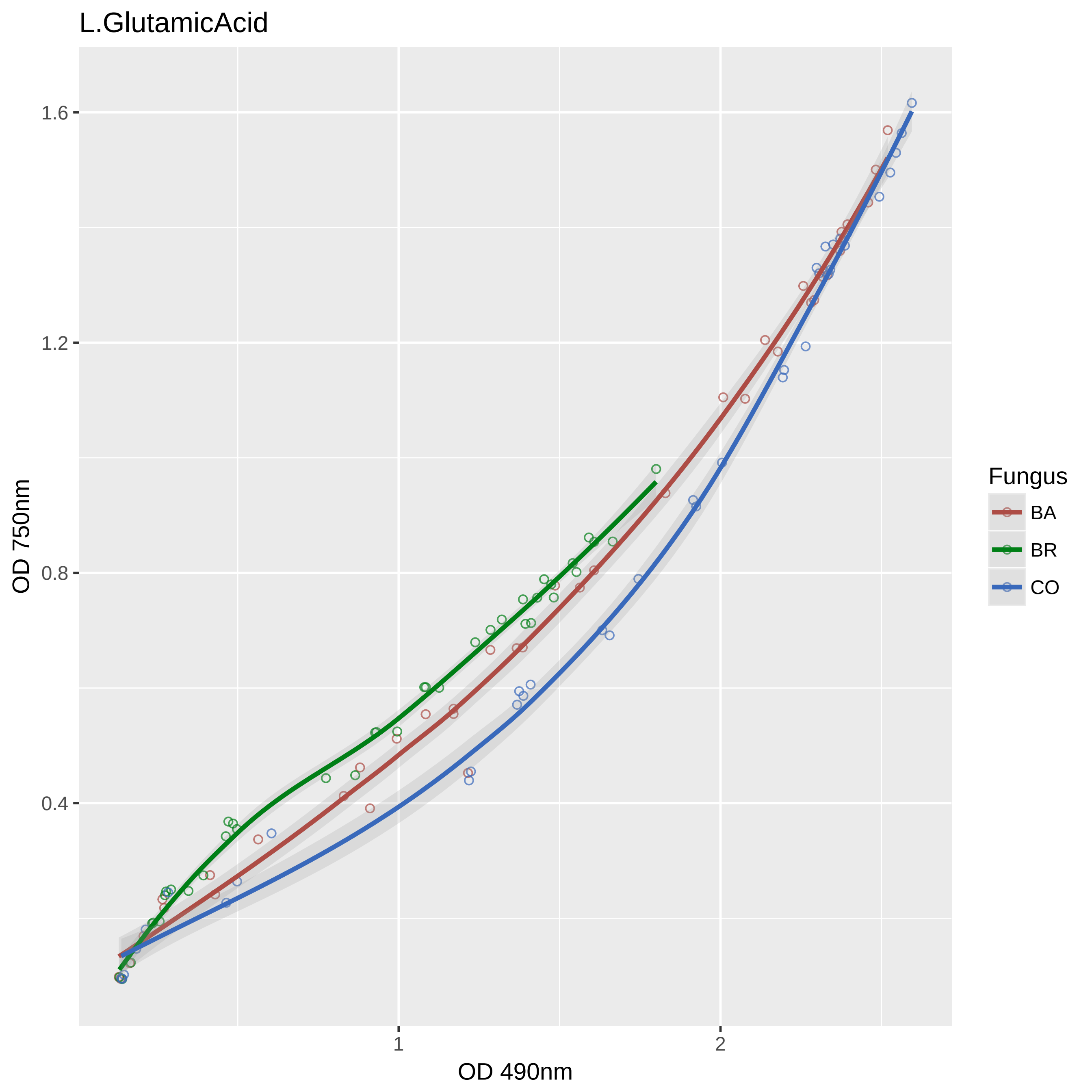
L.GlutamicAcid
1.6
1.2
0.8
0.4
OD 750nm
1
2
OD 490nm
Fungus
BA
BR
CO

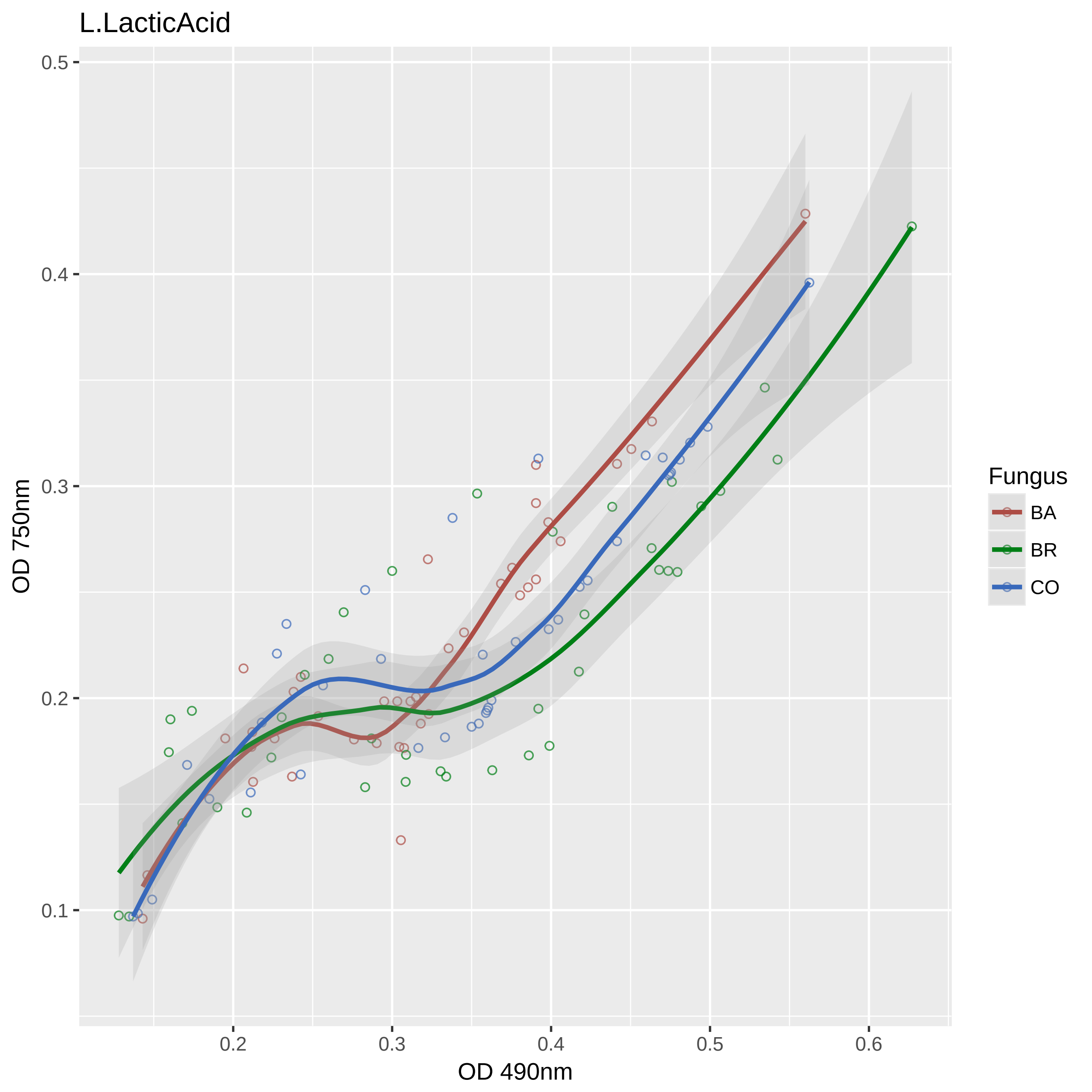

L.LacticAcid
0.5
0.4
0.3
0.2
0.1
OD 750nm
0.2
0.3
0.4
0.5
0.6
OD 490nm
Fungus
BA
BR
CO

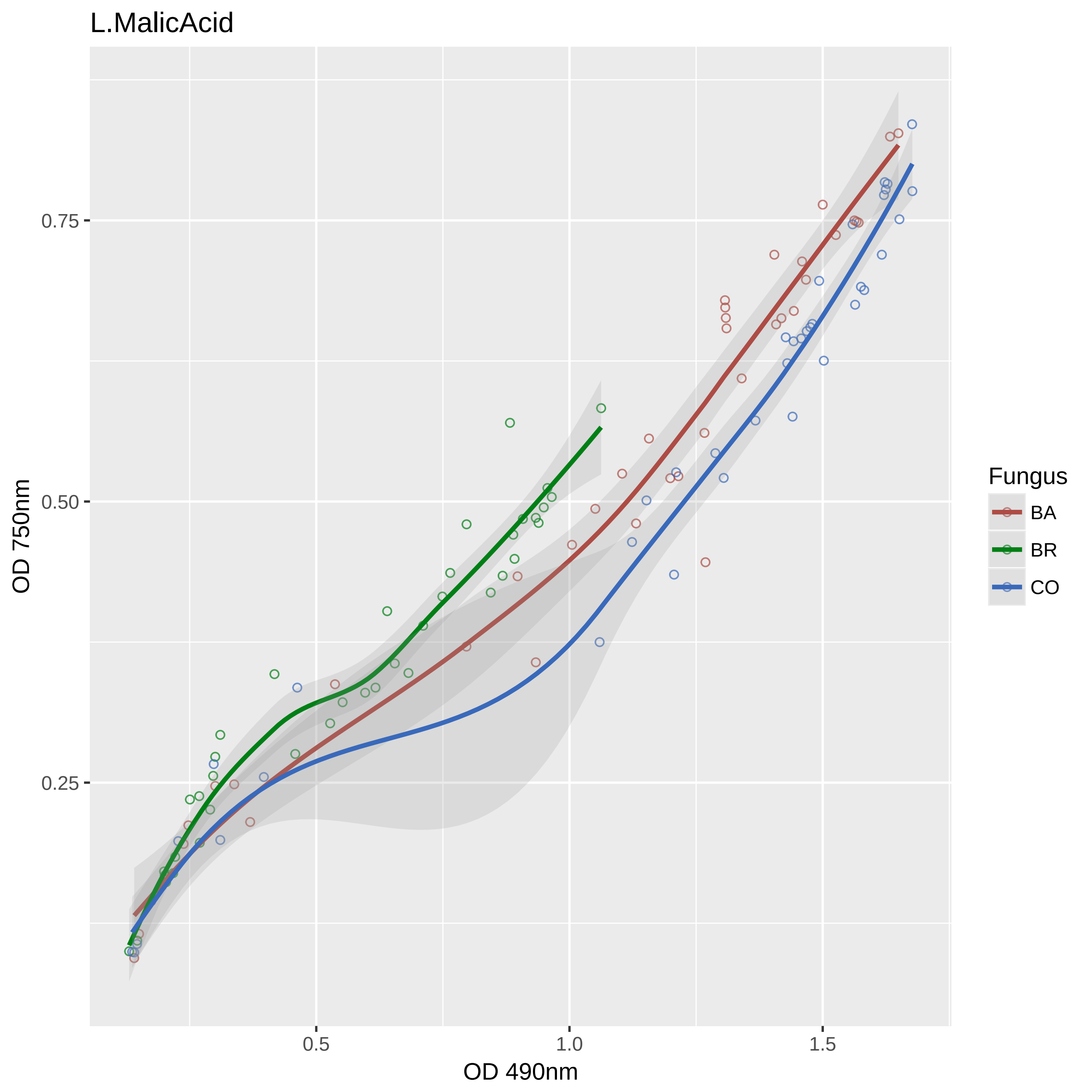
L.MalicAcid
OD 750nm
0.75
0.50
0.25
0.5
1.0
1.5
OD 490nm
Fungus
BA
BR
CO

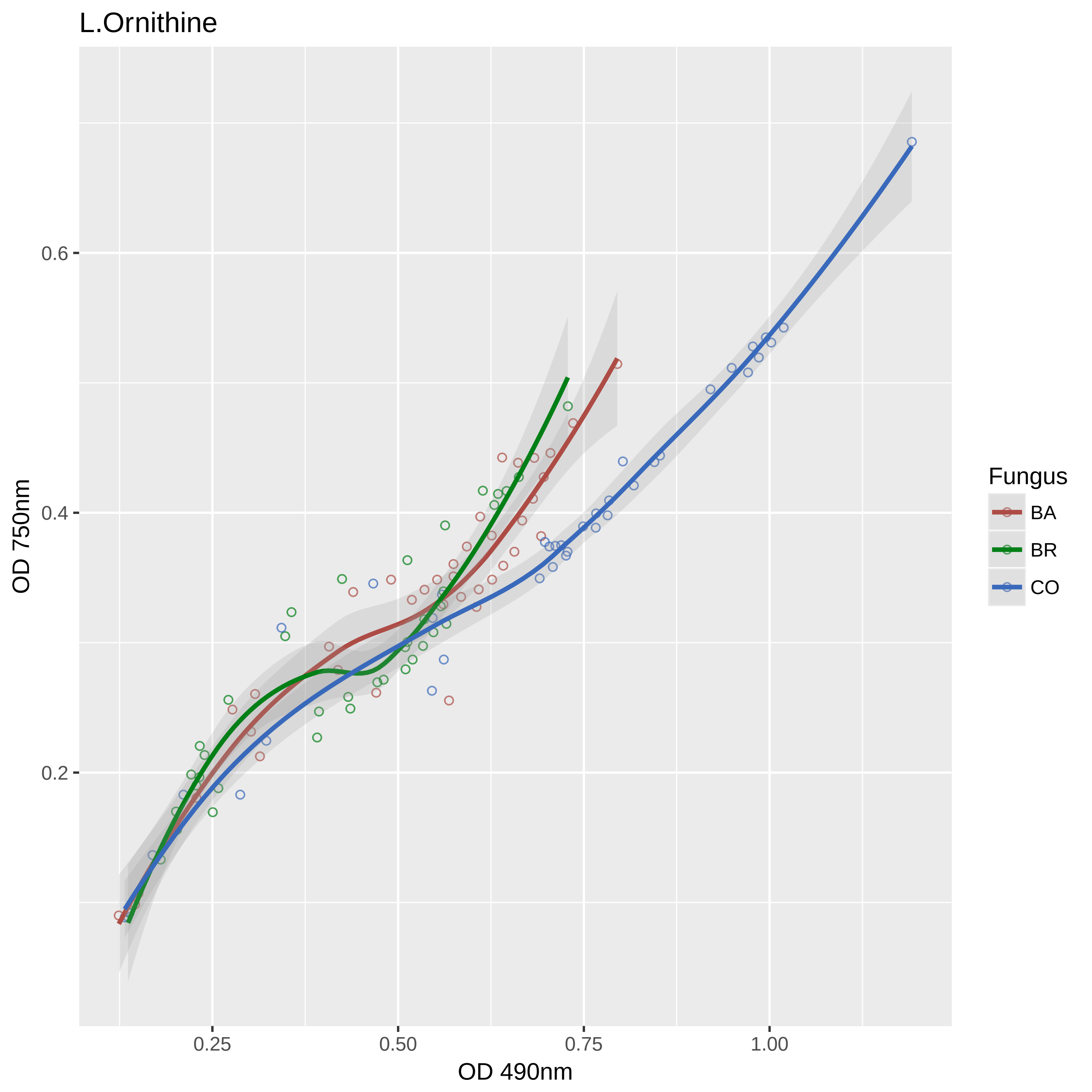

L.Ornithine
OD 750nm
OD 490nm
0.2
0.4
0.6
0.25
0.50
0.75
1.00
Fungus
BA
BR
CO

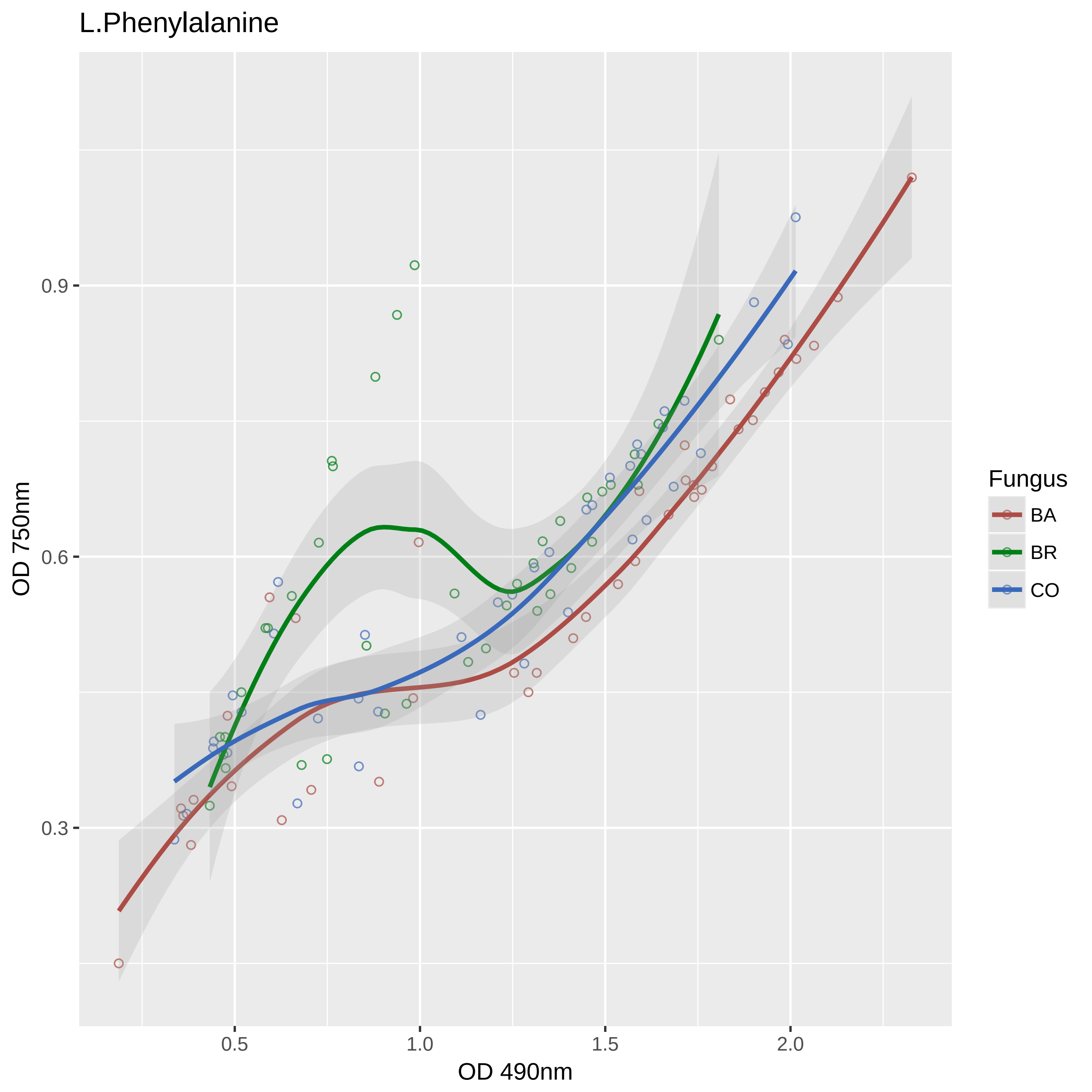

L.Phenylalanine
0.9
0.6
0.3
0.5
1.0
1.5
2.0
OD 490nm
OD 750nm
Fungus
BA
BR
CO

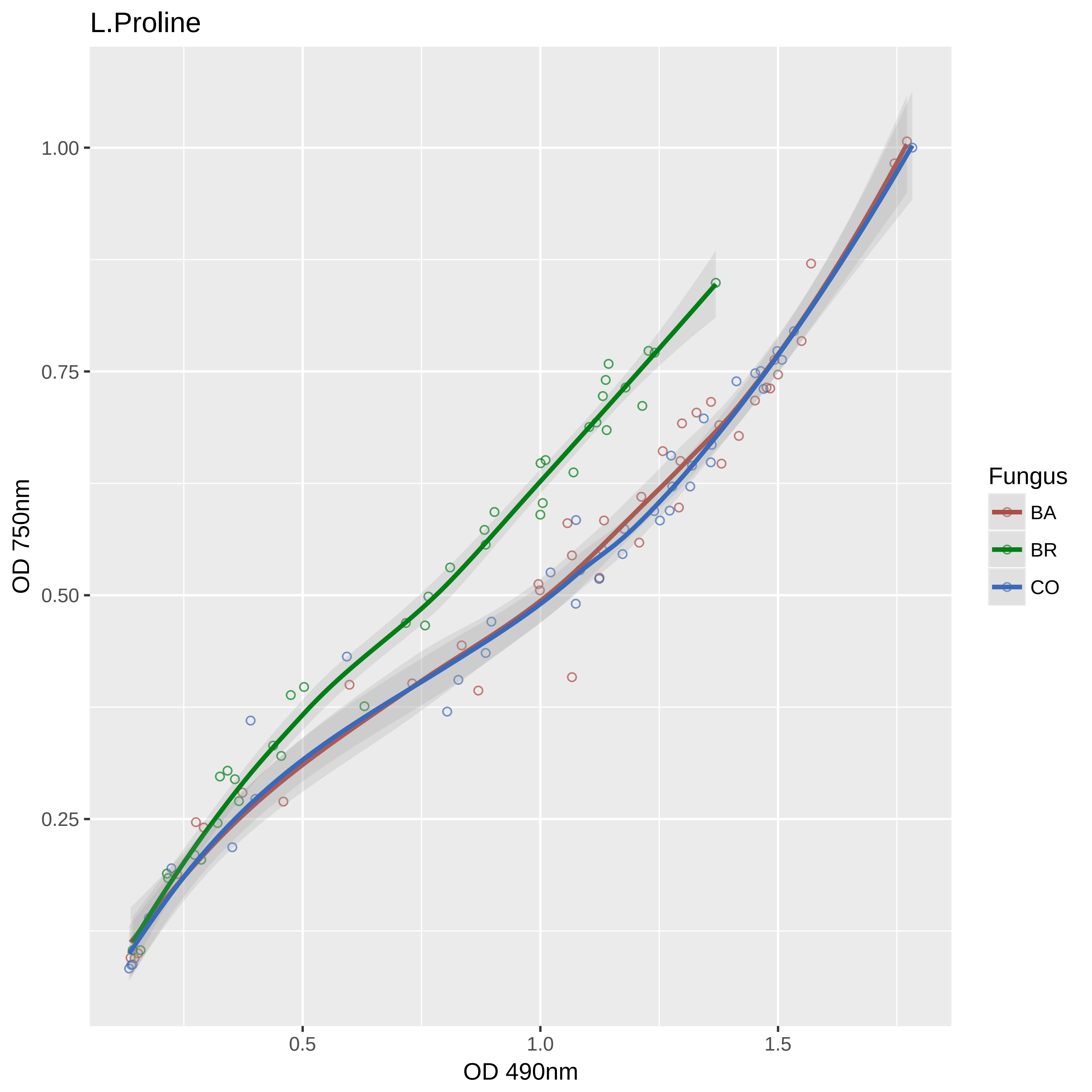

L.Proline
OD 750nm
1.00
0.75
0.50
0.25
0.5
1.0
1.5
OD 490nm
Fungus
BA
BR
CO

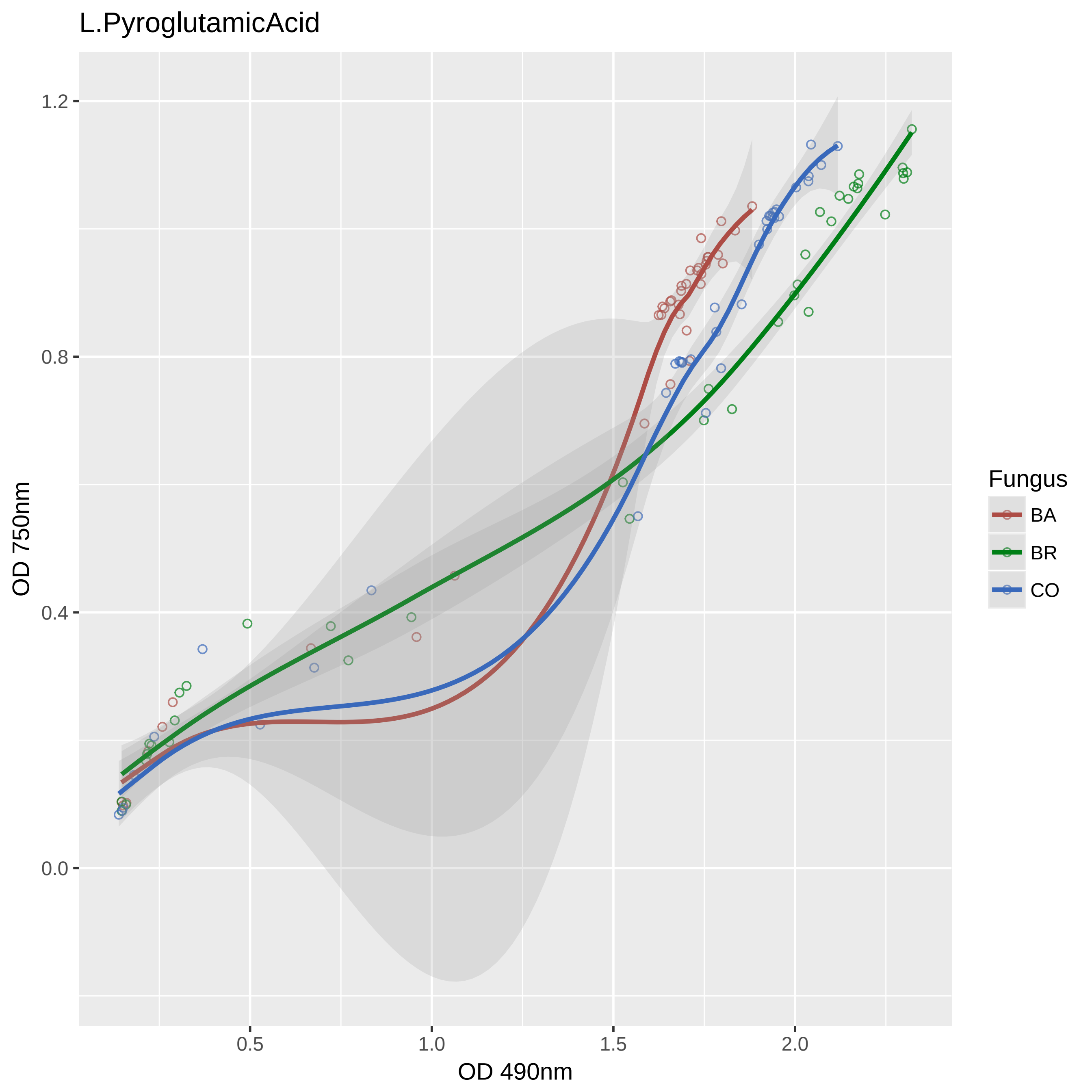

L.PyroglutamicAcid
OD 750nm
OD 490nm
1.2
0.8
0.4
0.0
0.5
1.0
1.5
2.0
Fungus
BA
BR
CO

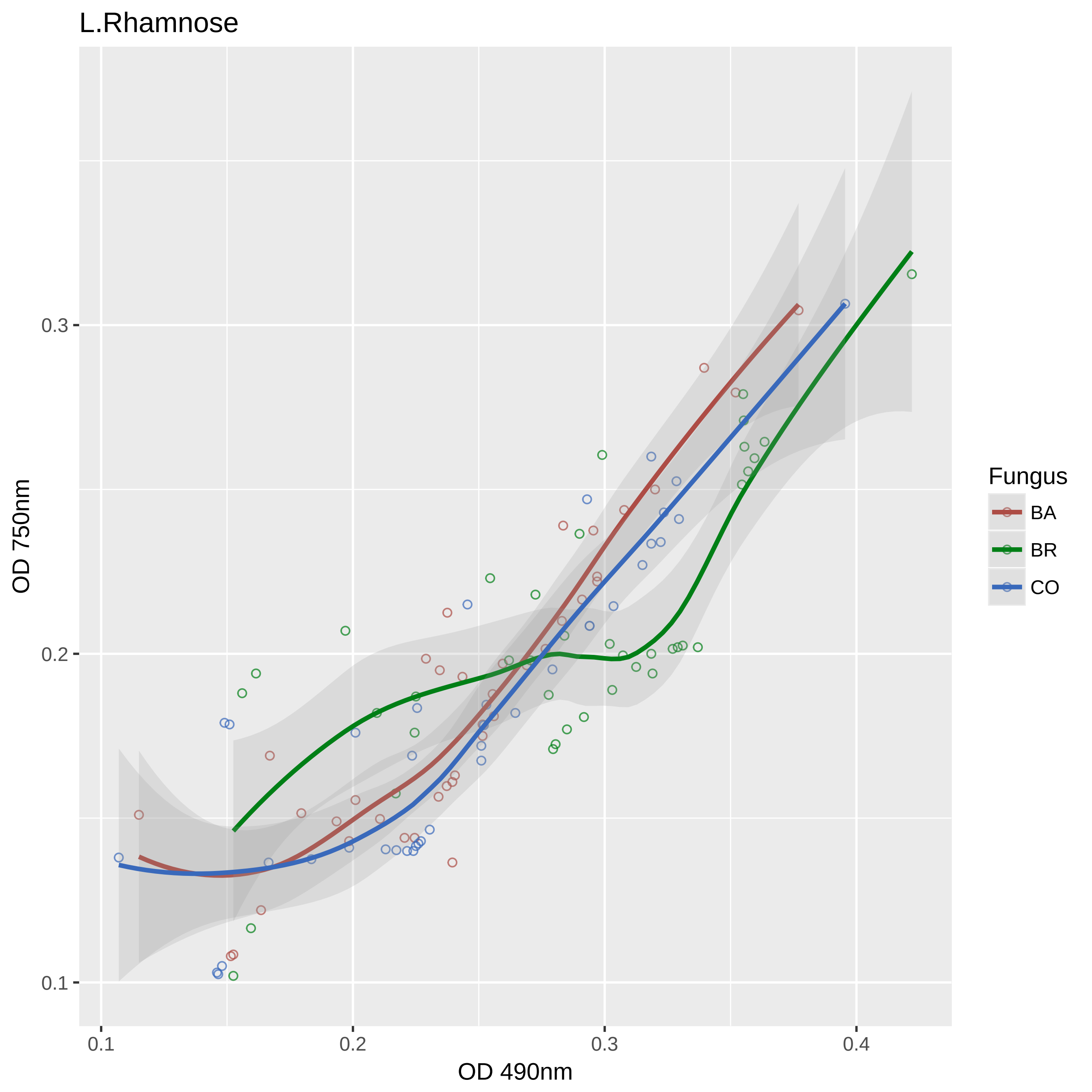

L.Rhamnose
OD 750nm
OD 490nm
0.1
0.2
0.3
0.4
0.1
0.2
0.3
Fungus
BA
BR
CO

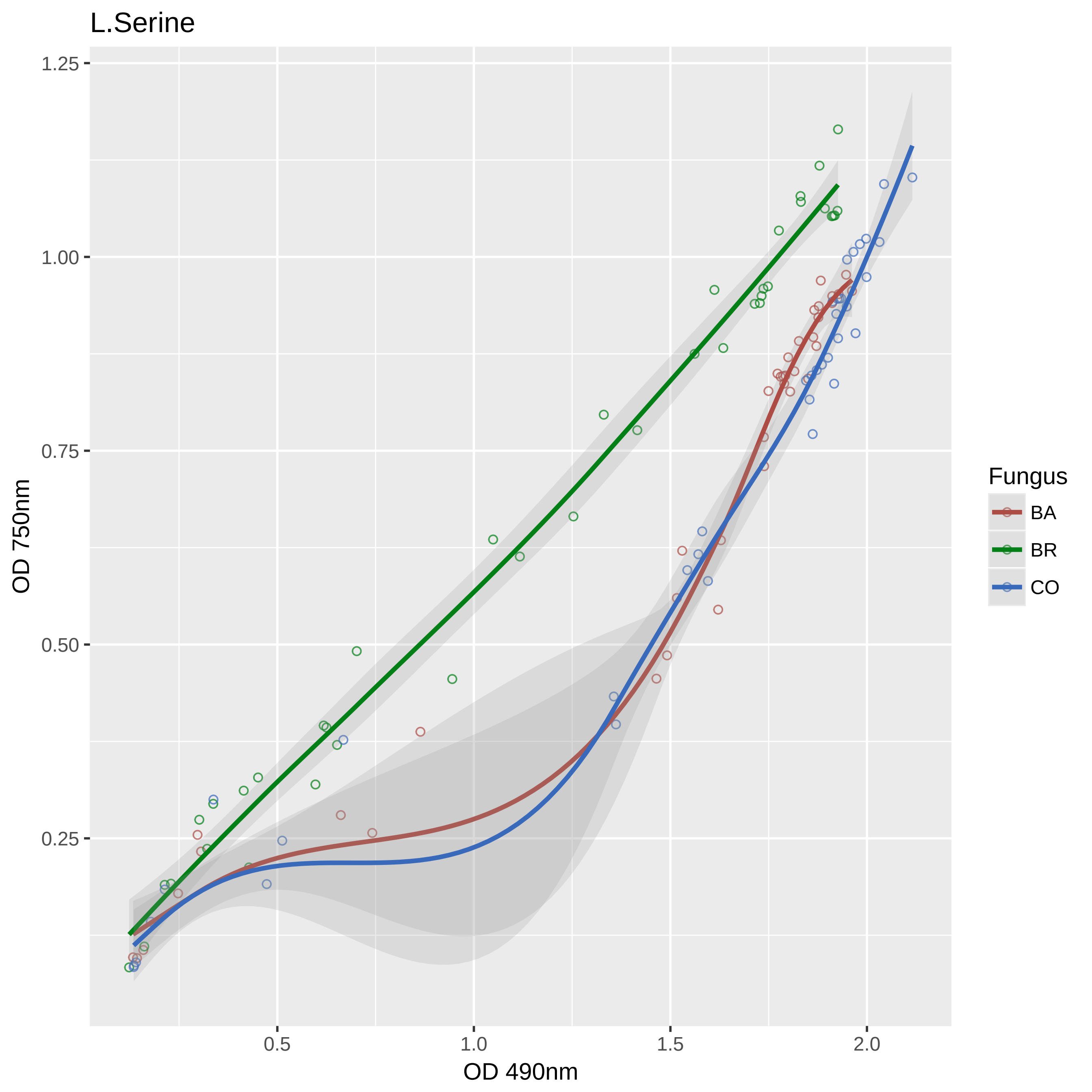

L.Serine
OD 750nm
OD 490nm
1.25
1.00
0.75
0.50
0.25
0.5
1.0
1.5
2.0
Fungus
BA
BR
CO

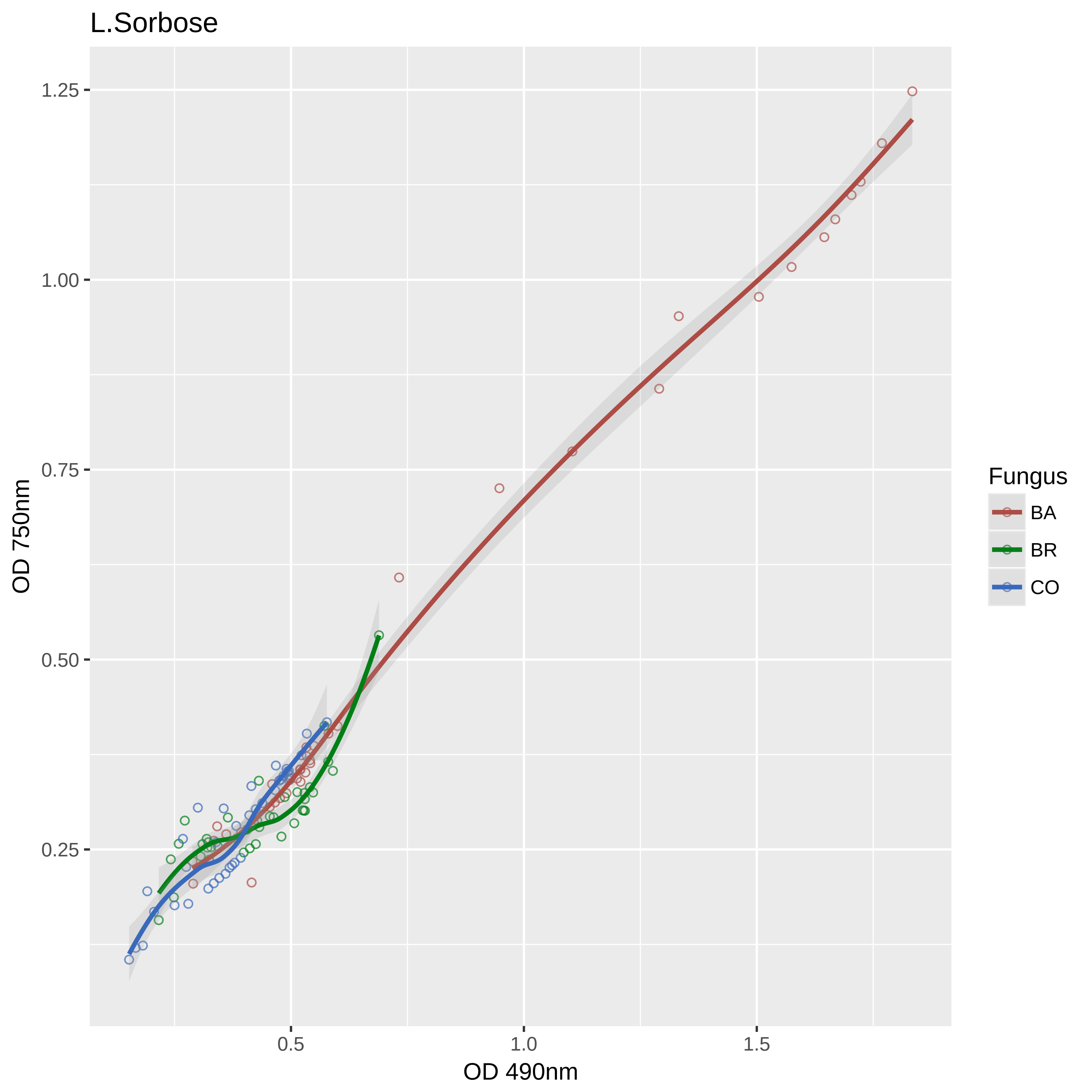

L.Sorbose
1.25
1.00
0.75
0.50
0.25
OD 750nm
0.5
1.0
1.5
OD 490nm
Fungus
BA
BR
CO

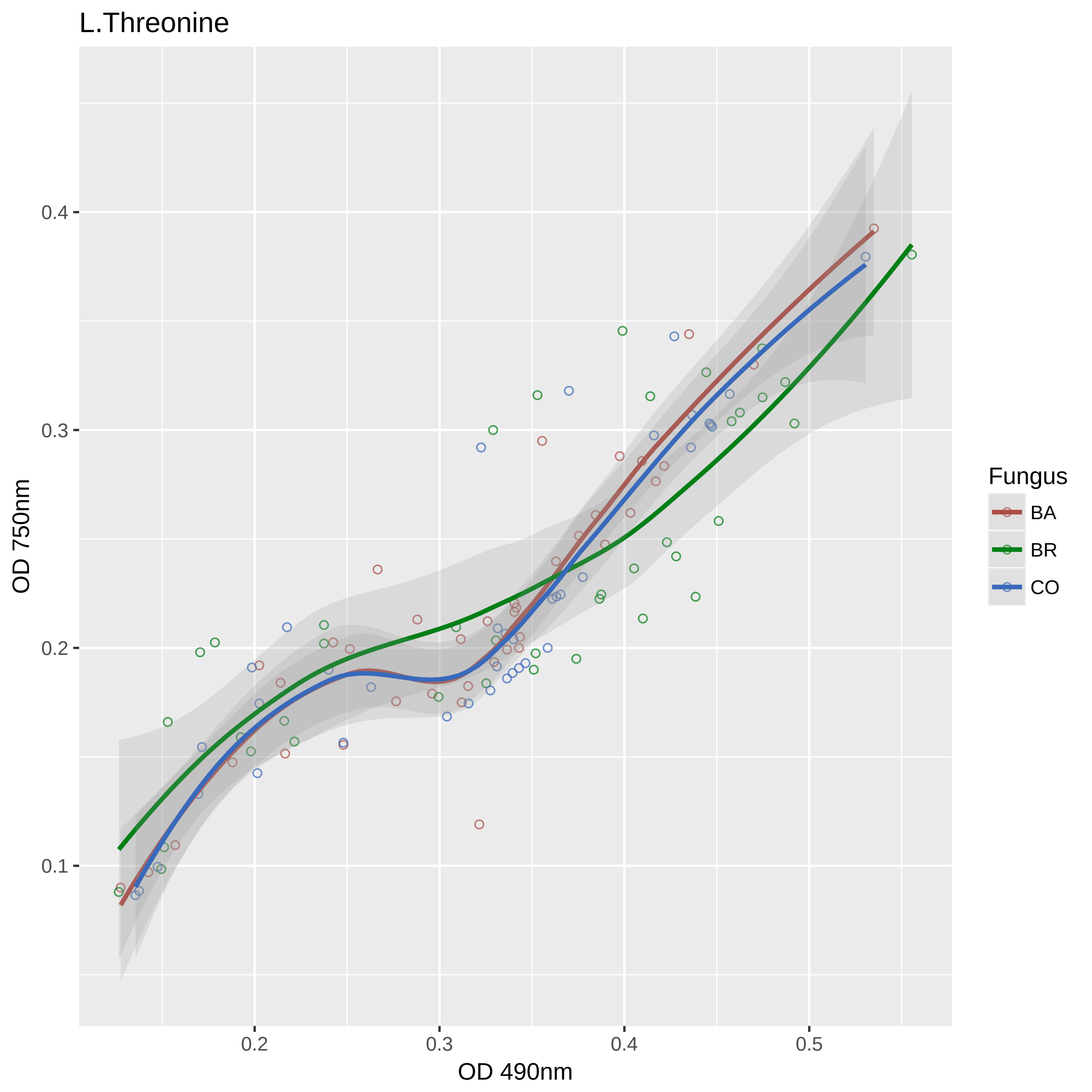

L.Threonine
OD 750nm
0.4
0.3
0.2
0.1
OD 490nm
0.2
0.3
0.4
0.5
Fungus
BA
BR
CO

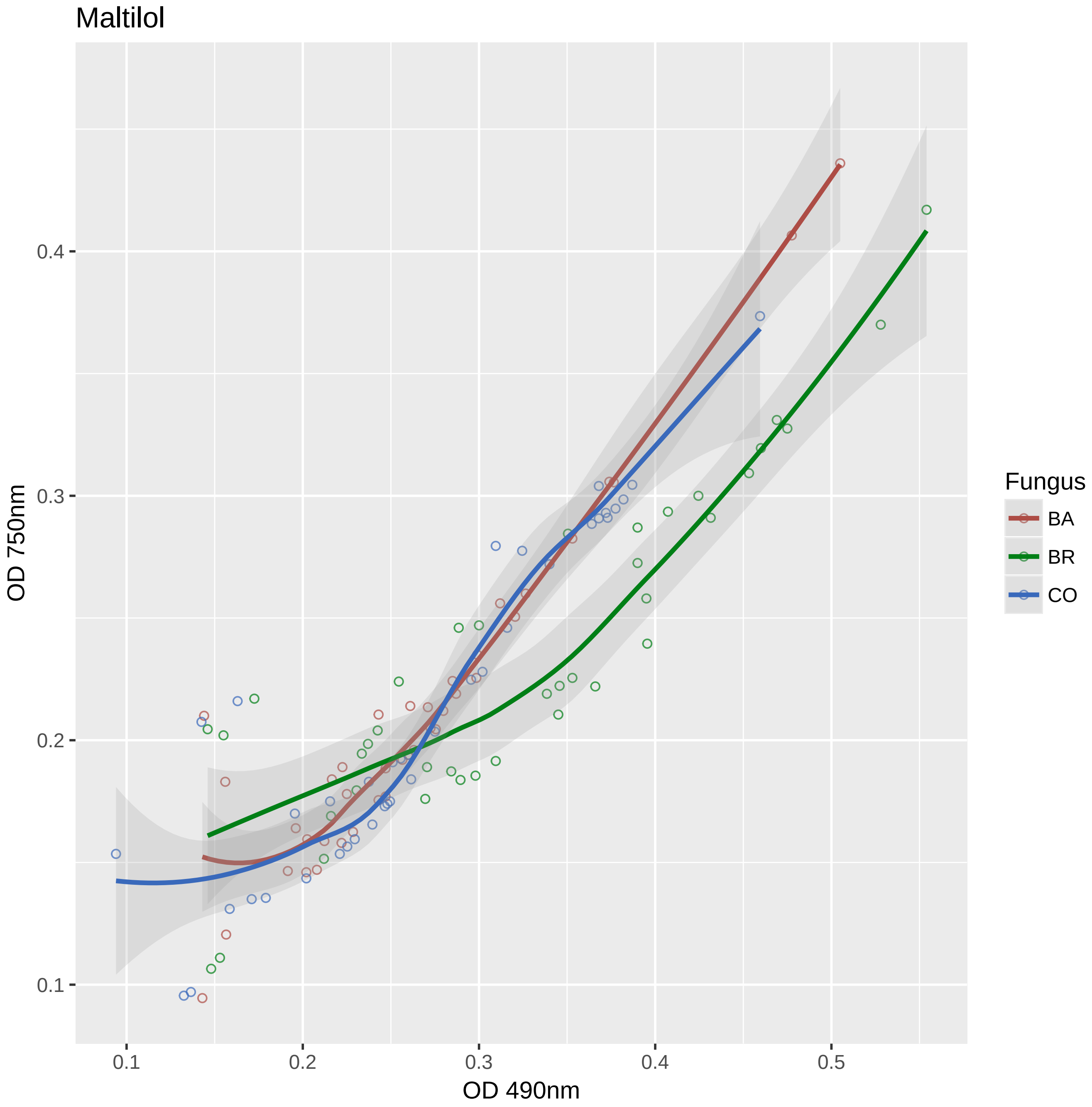

Maltilol
OD 750nm
0.4
0.3
0.2
0.1
0.1
0.2
0.3
0.4
0.5
OD 490nm
Fungus
BA
BR
CO

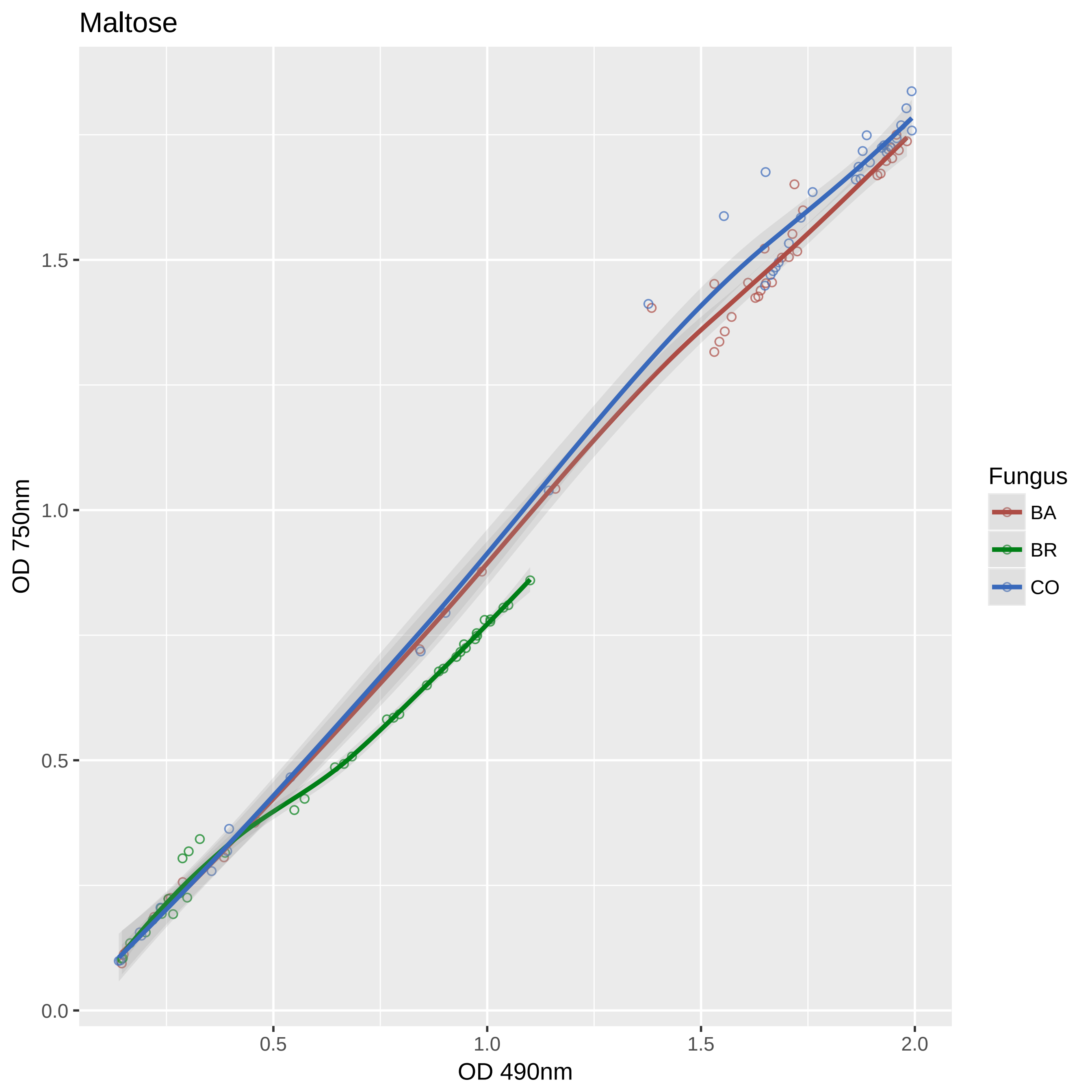

Maltose
OD 750nm
0.0
0.5
1.0
1.5
OD 490nm
0.5
1.0
1.5
2.0
Fungus
BA
BR
CO

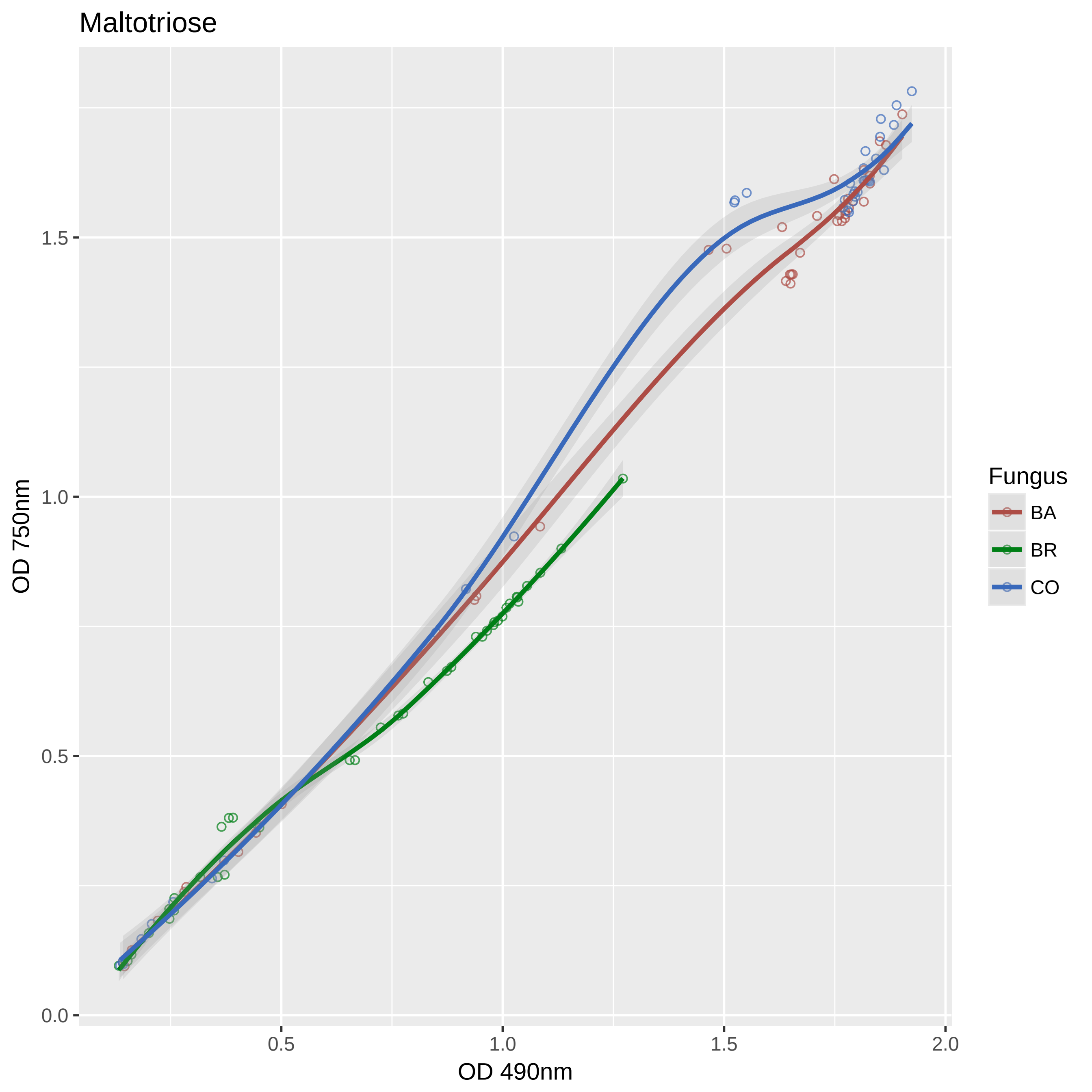

Maltotriose
OD 750nm
OD 490nm
0.0
0.5
1.0
1.5
0.5
1.0
1.5
2.0
Fungus
BA
BR
CO

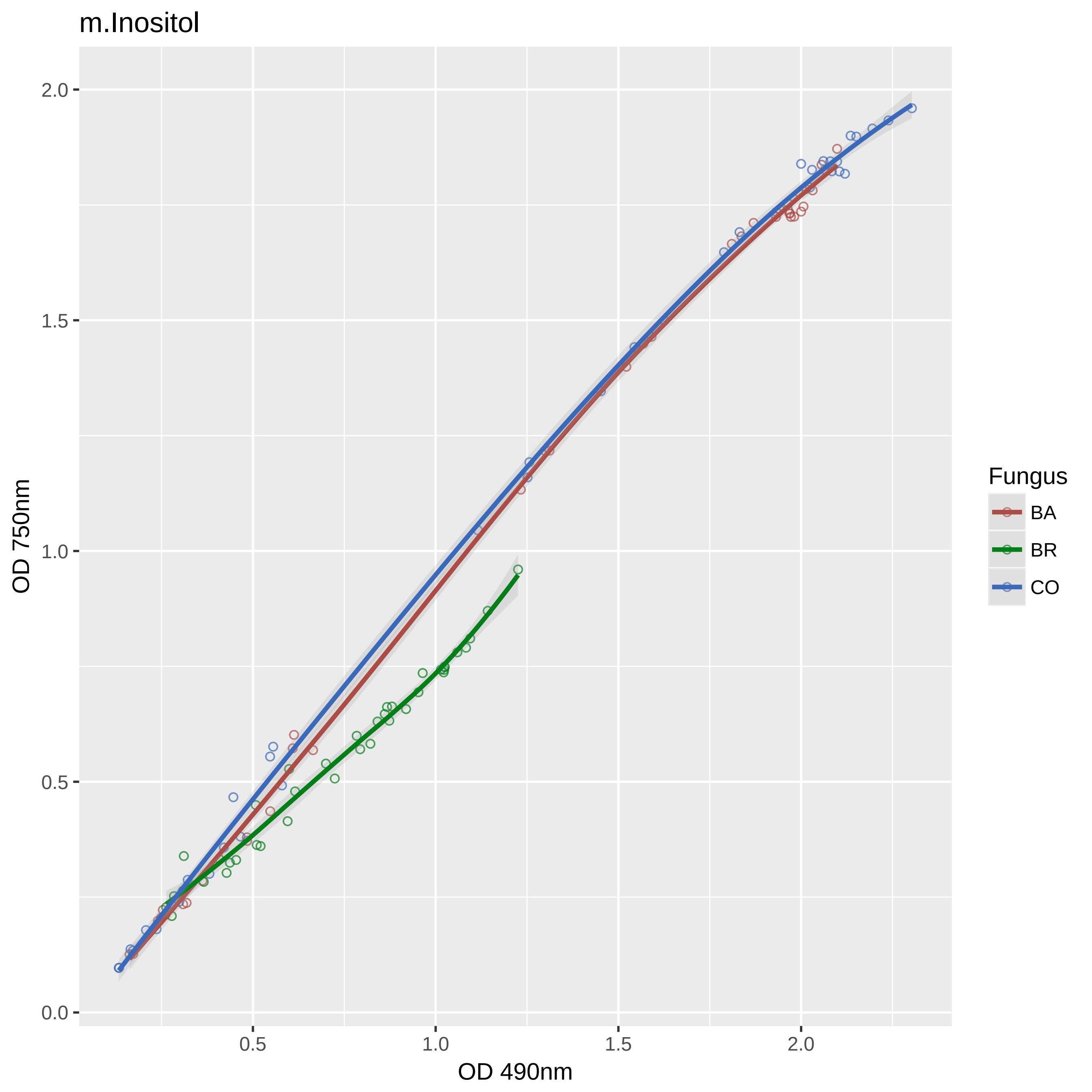

m.Inositol
OD 750nm
2.0
1.5
1.0
0.5
0.0
0.5
1.0
1.5
2.0
OD 490nm
Fungus
BA
BR
CO

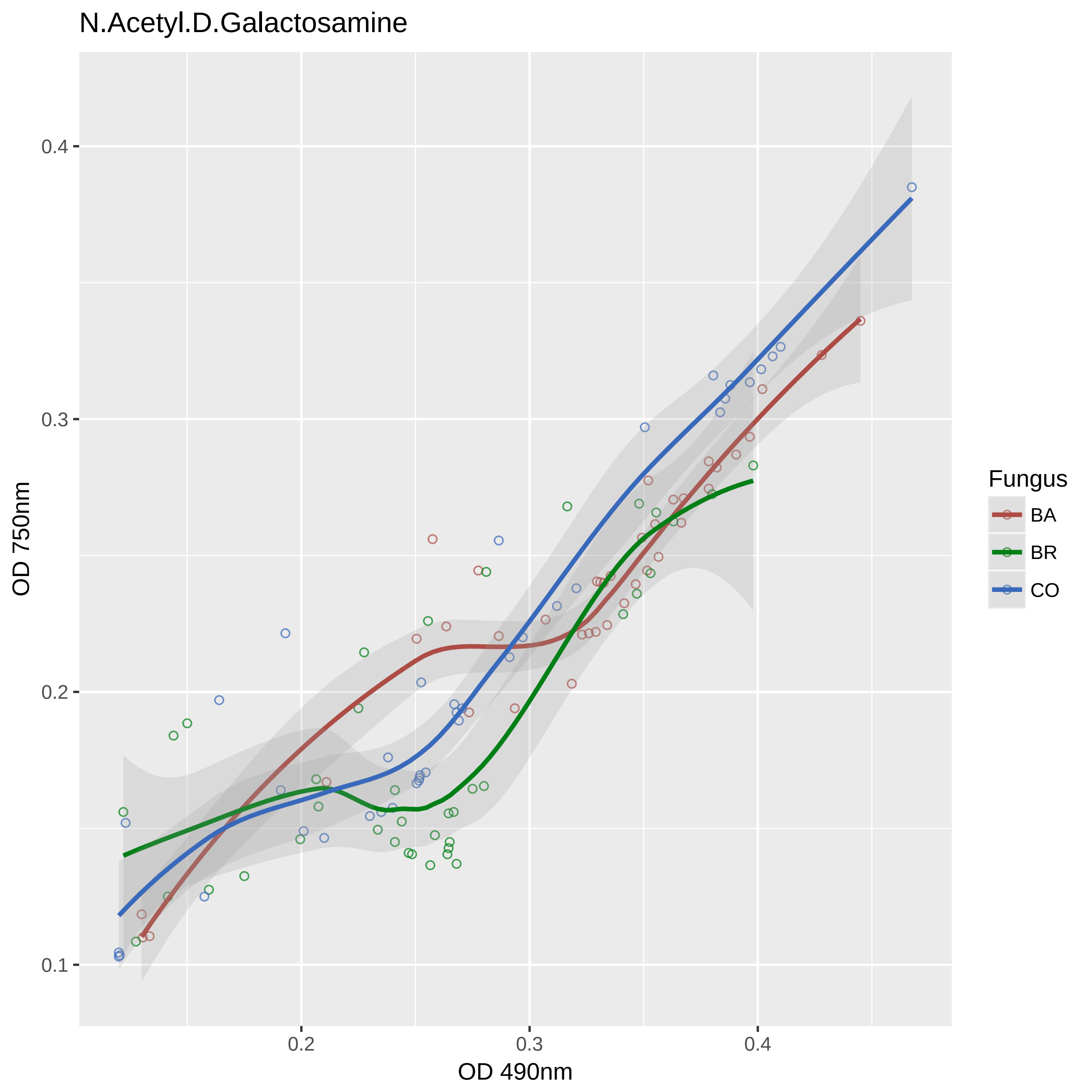

N.Acetyl.D.Galactosamine
OD 750nm
OD 490nm
0.1
0.2
0.3
0.4
0.2
0.3
0.4
Fungus
BA
BR
CO

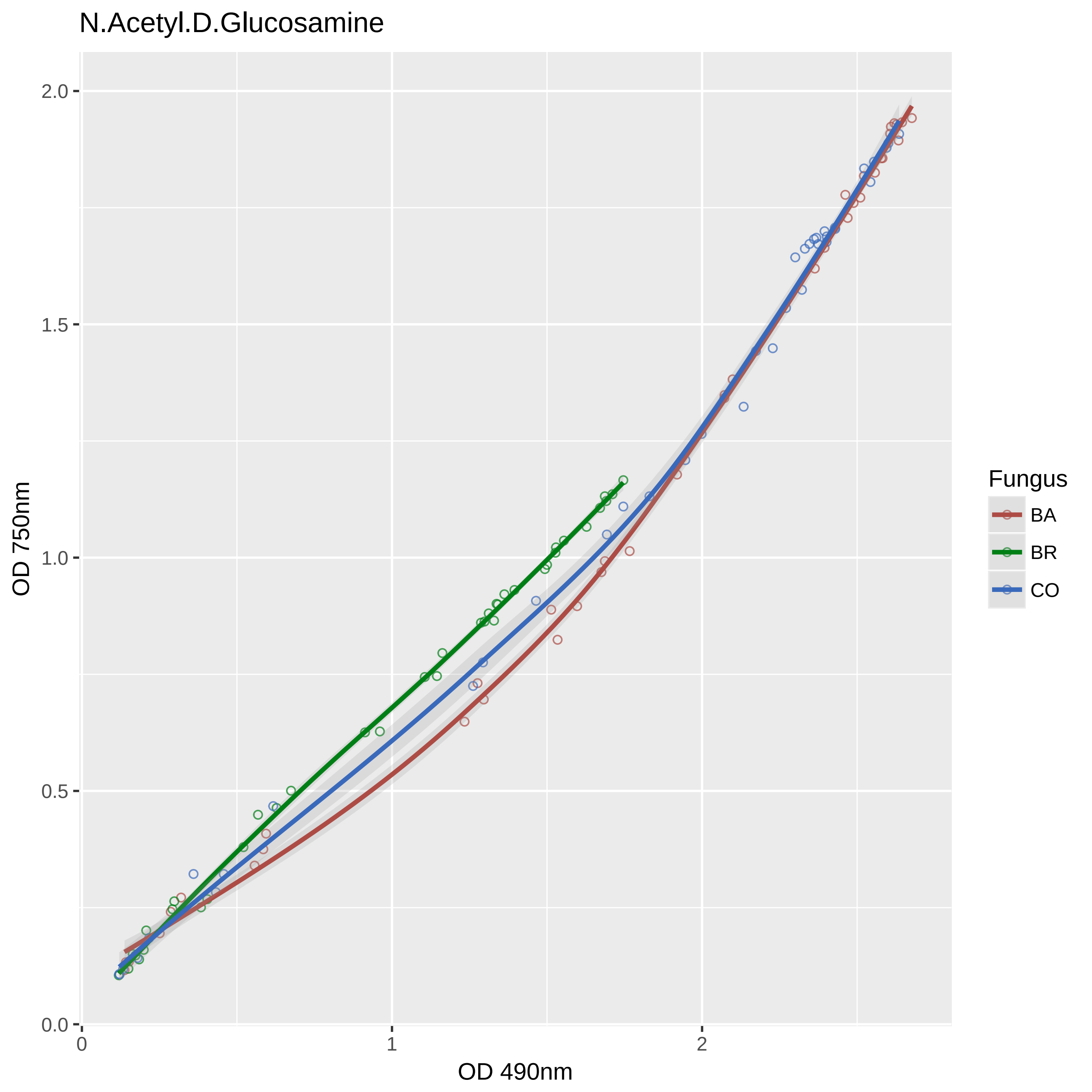

N.Acetyl.D.Glucosamine
OD 750nm
OD 490nm
0.0
0.5
1.0
1.5
2.0
0
1
2
Fungus
BA
BR
CO

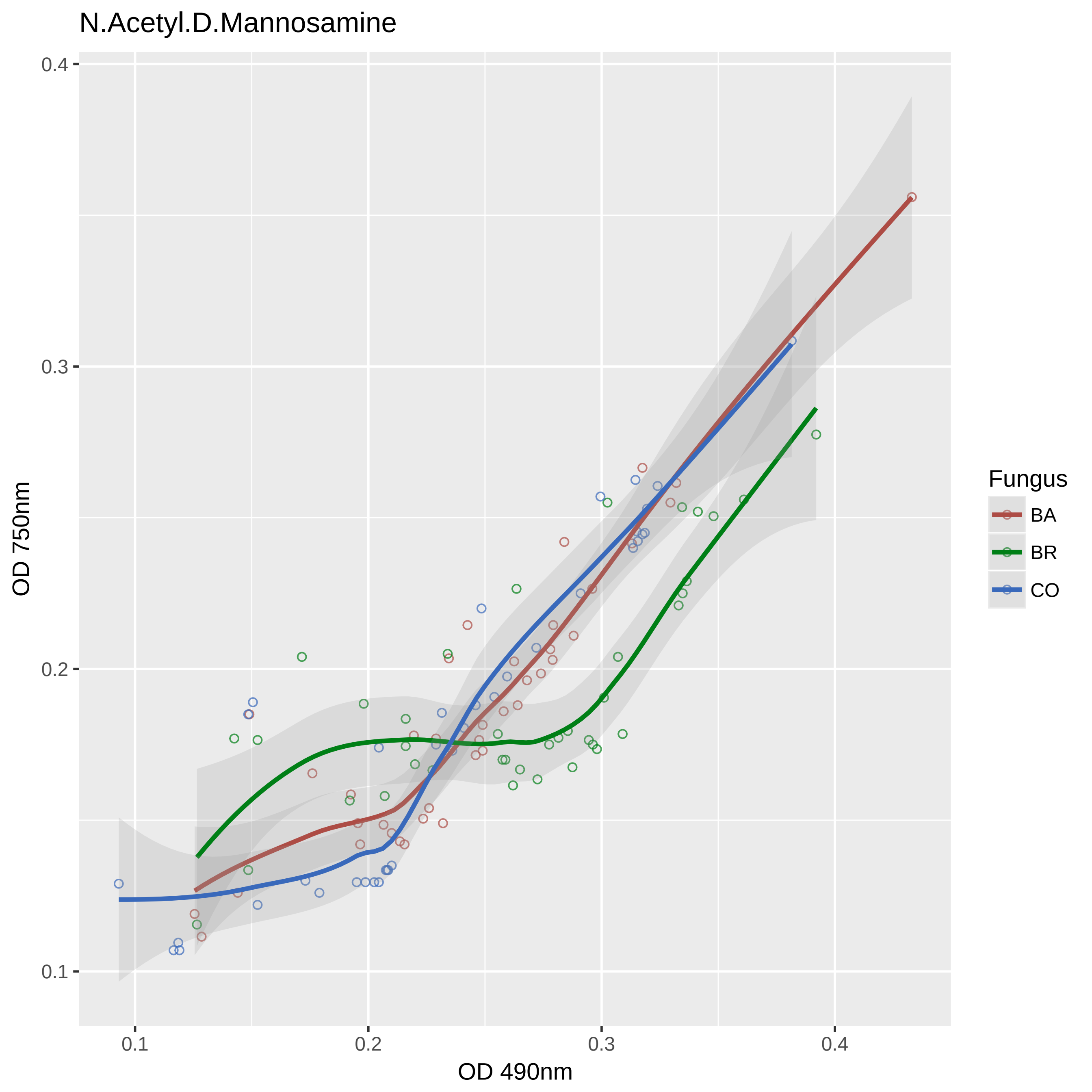
N.Acetyl.D.Mannosamine
0.4
0.3
0.2
0.1
OD 750nm
0.1
0.2
0.3
0.4
OD 490nm
Fungus
BA
BR
CO

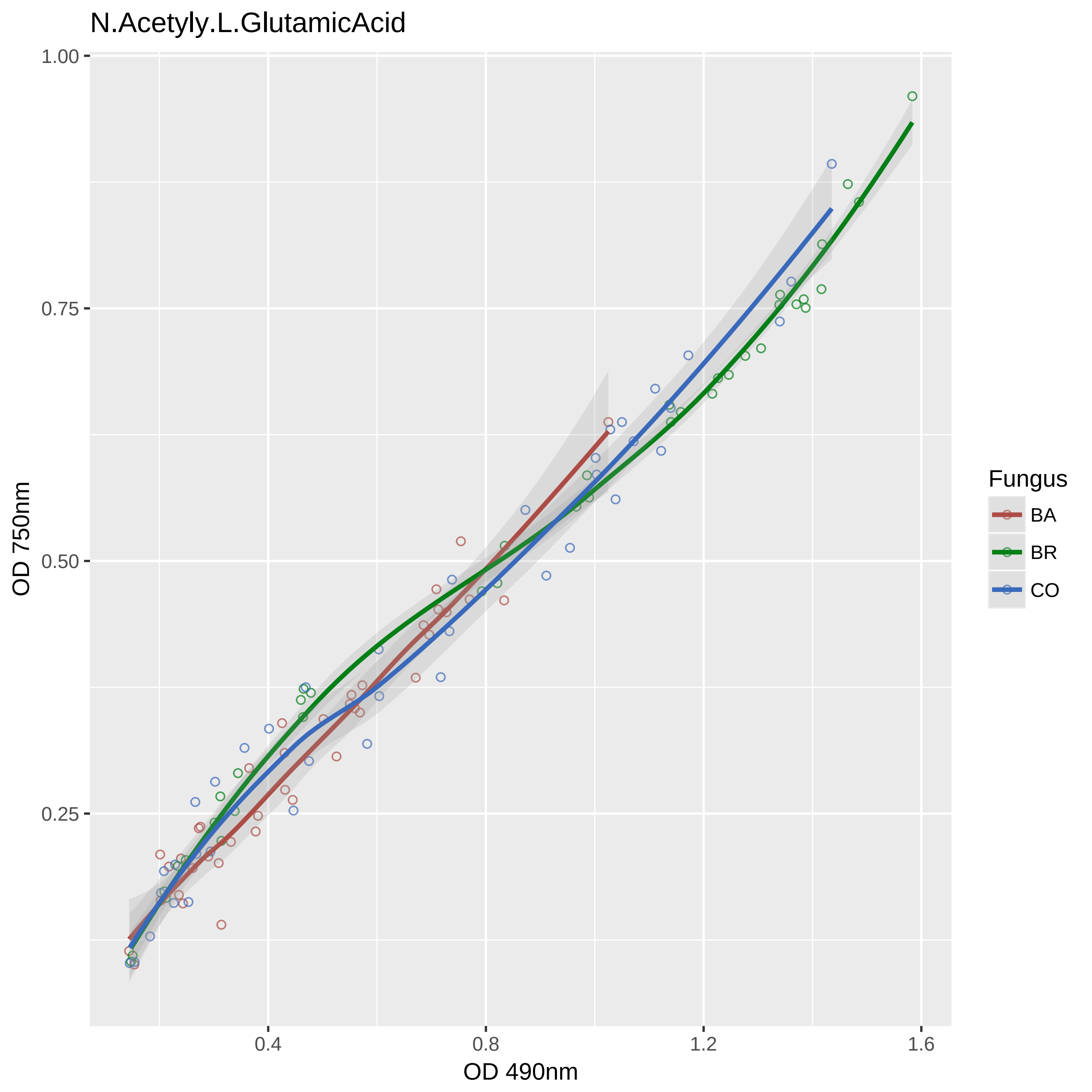

N.Acetyly.L.GlutamicAcid
1.00
0.75
0.50
0.25
OD 750nm
0.4
0.8
1.2
1.6
OD 490nm
Fungus
BA
BR
CO

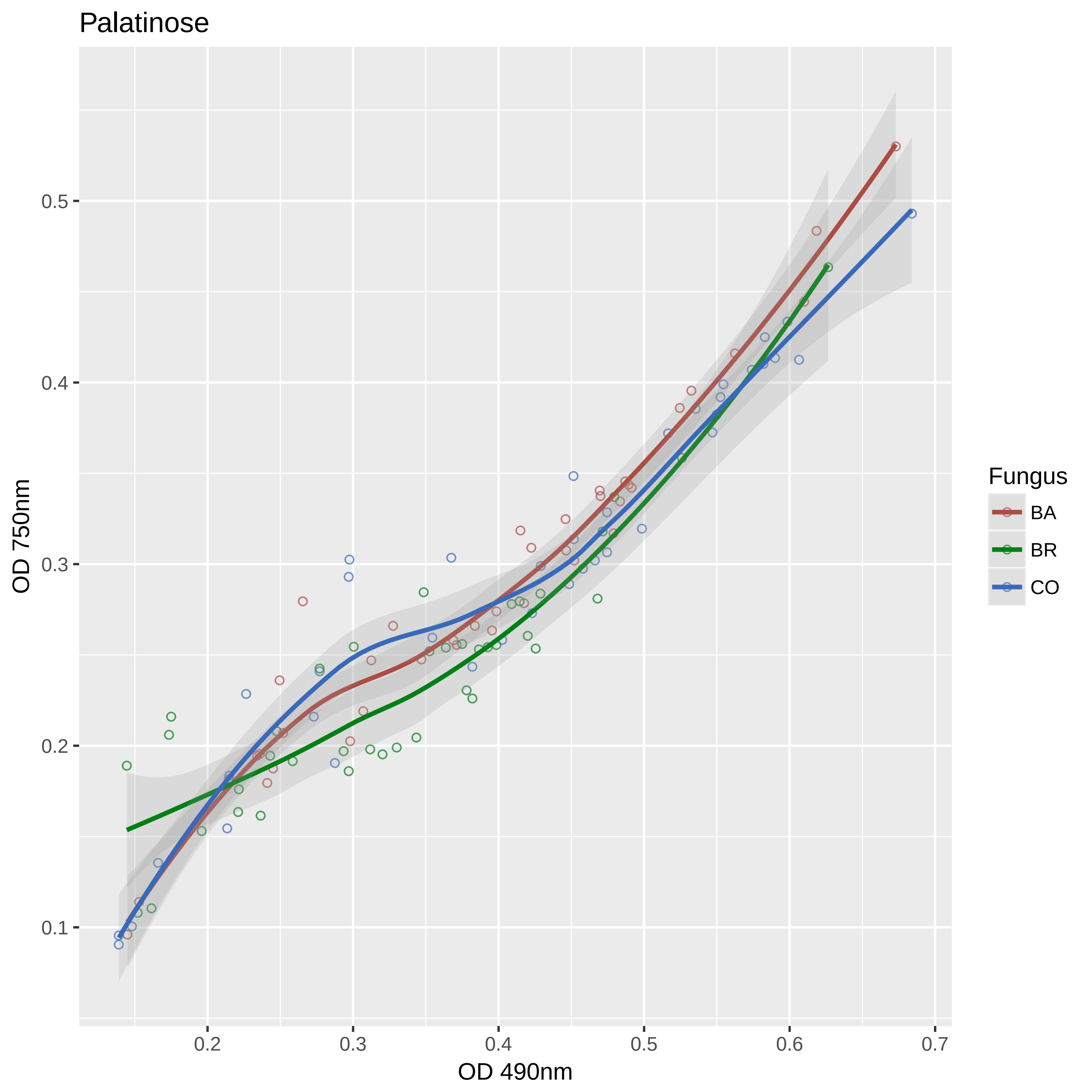

Palatinose
OD 750nm
OD 490nm
0.1
0.2
0.3
0.4
0.5
0.2
0.3
0.4
0.5
0.6
0.7
Fungus
BA
BR
CO

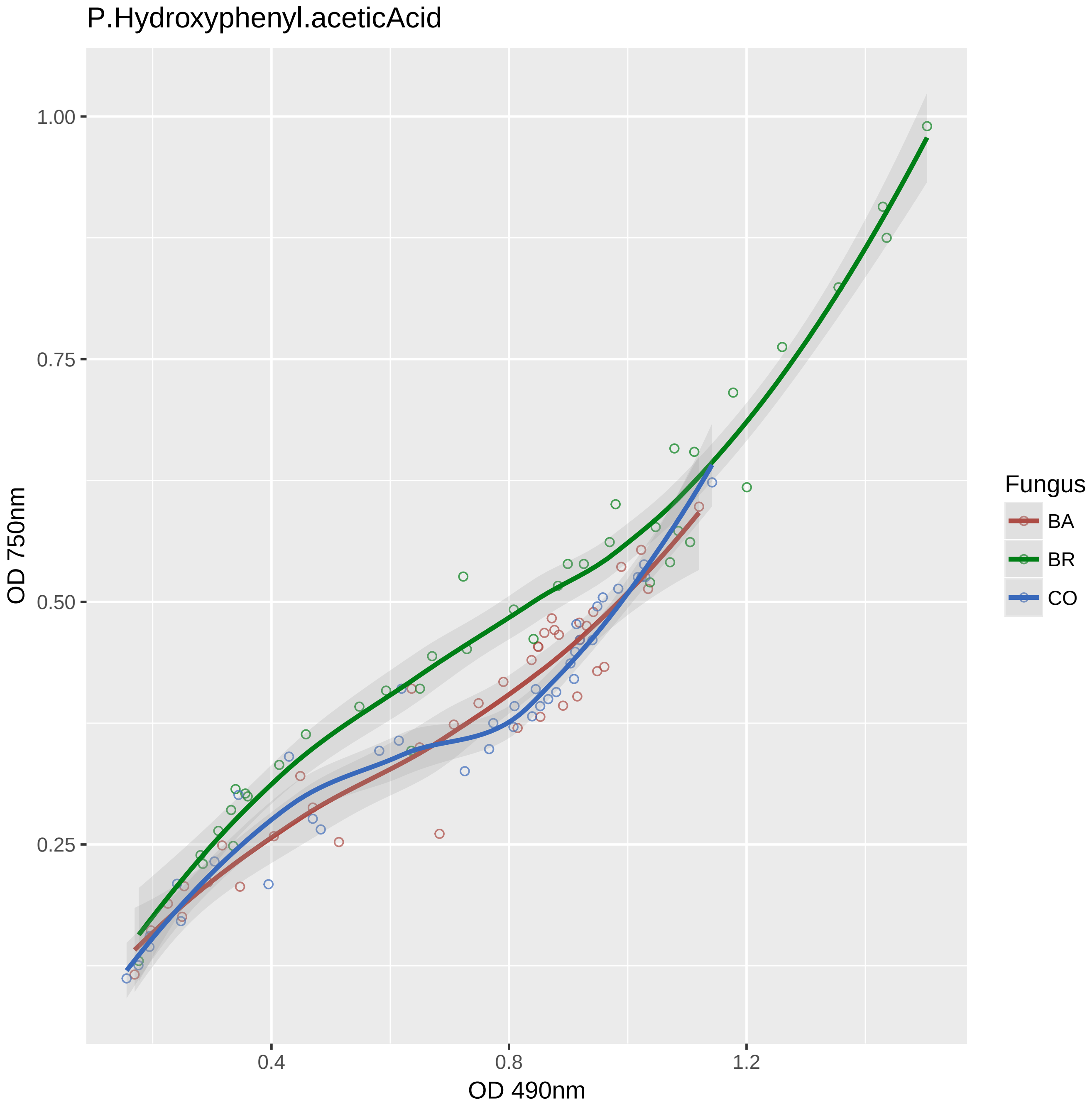
P.Hydroxyphenyl.aceticAcid
OD 750nm
1.00
0.75
0.50
0.25
OD 490nm
0.4
0.8
1.2
Fungus
BA
BR
CO

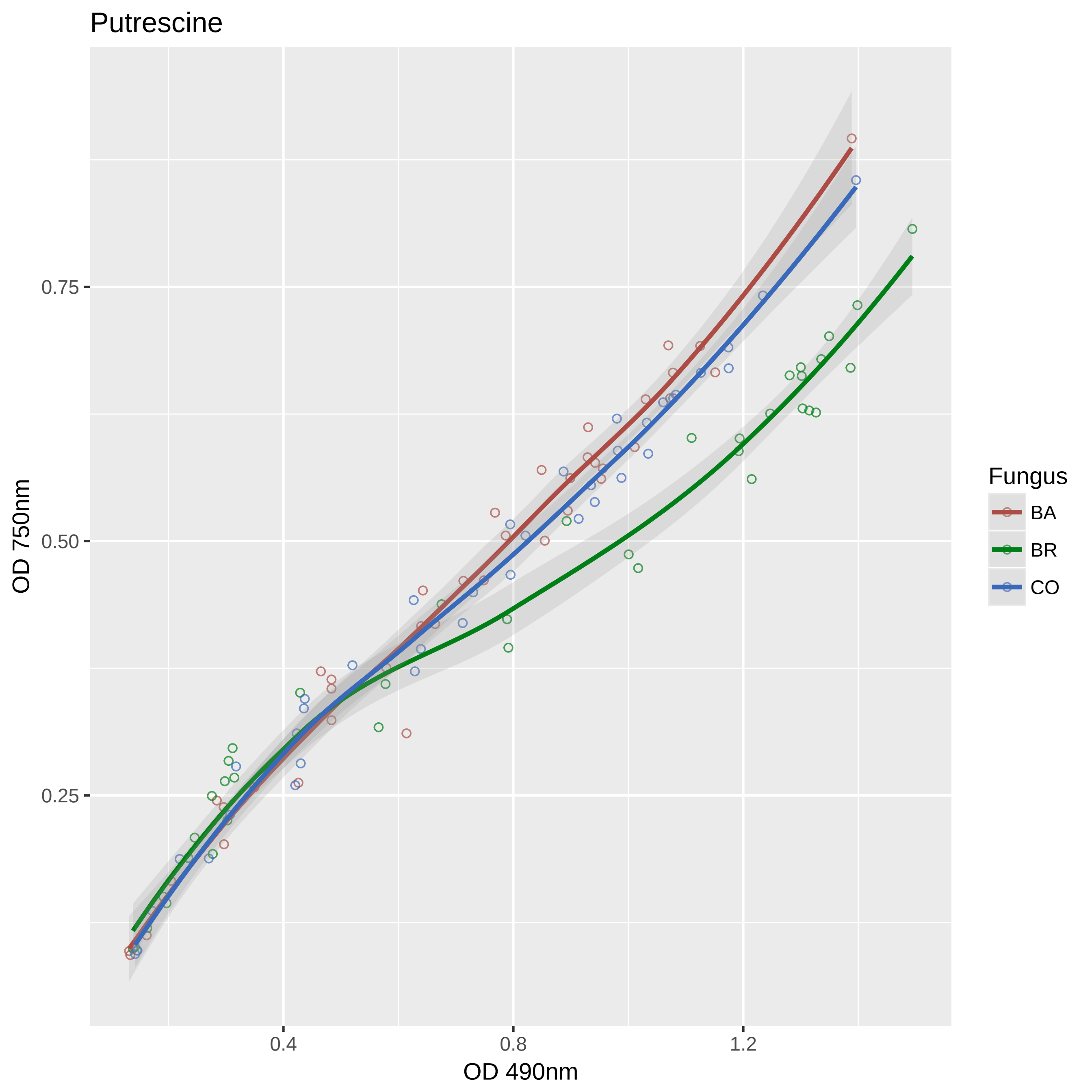

Putrescine
OD 750nm
0.25
0.50
0.75
OD 490nm
0.4
0.8
1.2
Fungus
BA
BR
CO

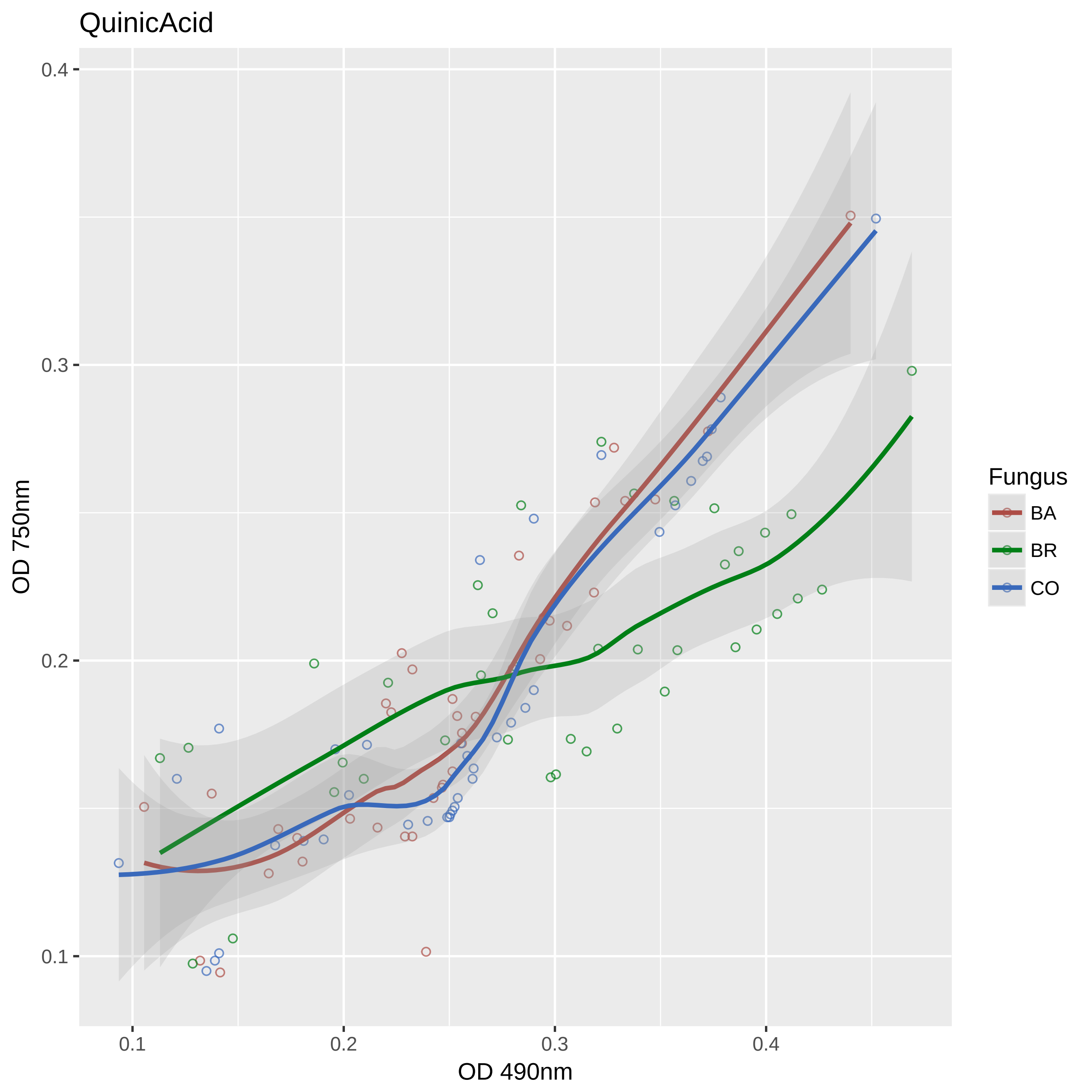
QuinicAcid
OD 750nm
OD 490nm
0.1
0.2
0.3
0.4
0.1
0.2
0.3
0.4
Fungus
BA
BR
CO

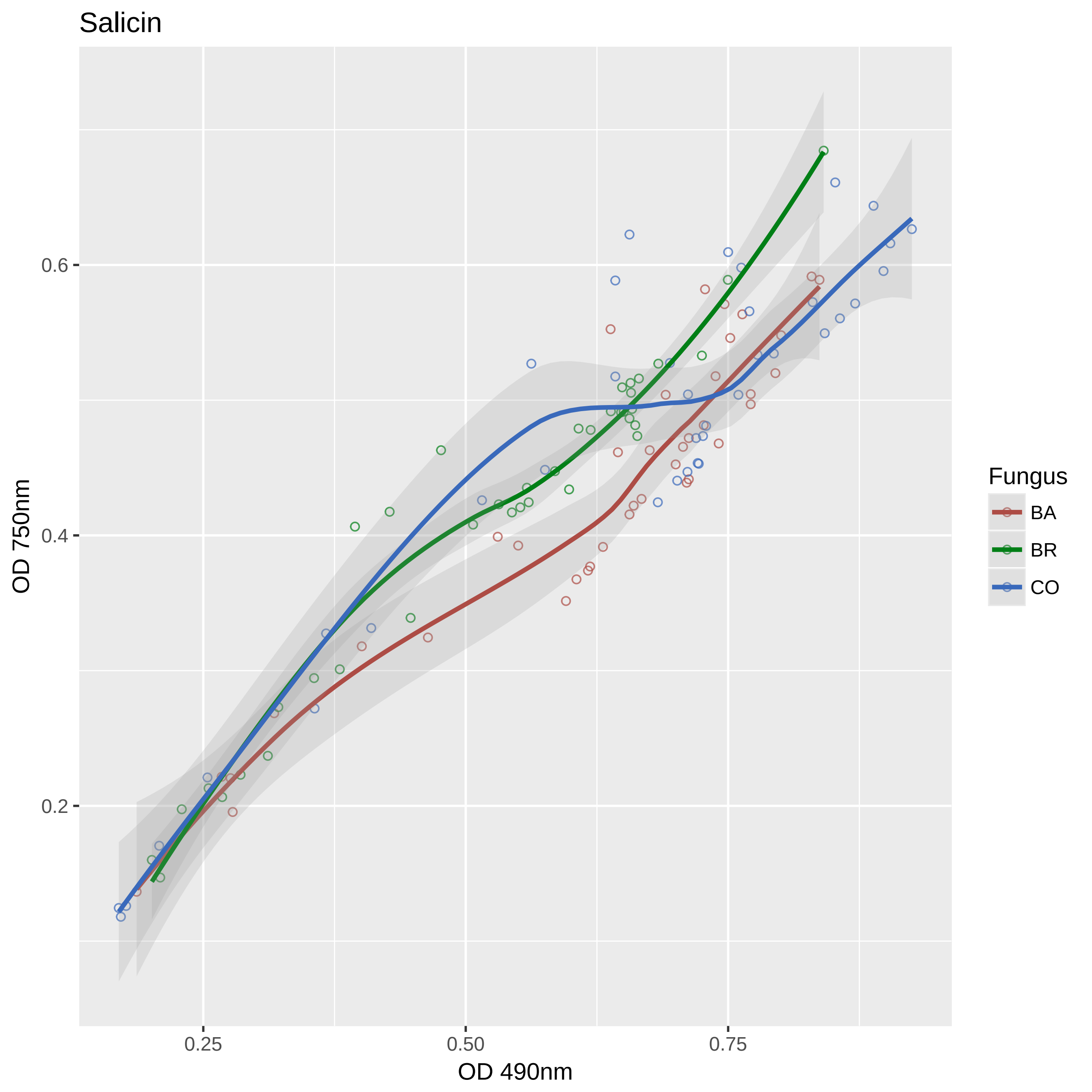
Salicin
OD 750nm
0.6
0.4
0.2
0.25
0.50
0.75
OD 490nm
Fungus
BA
BR
CO

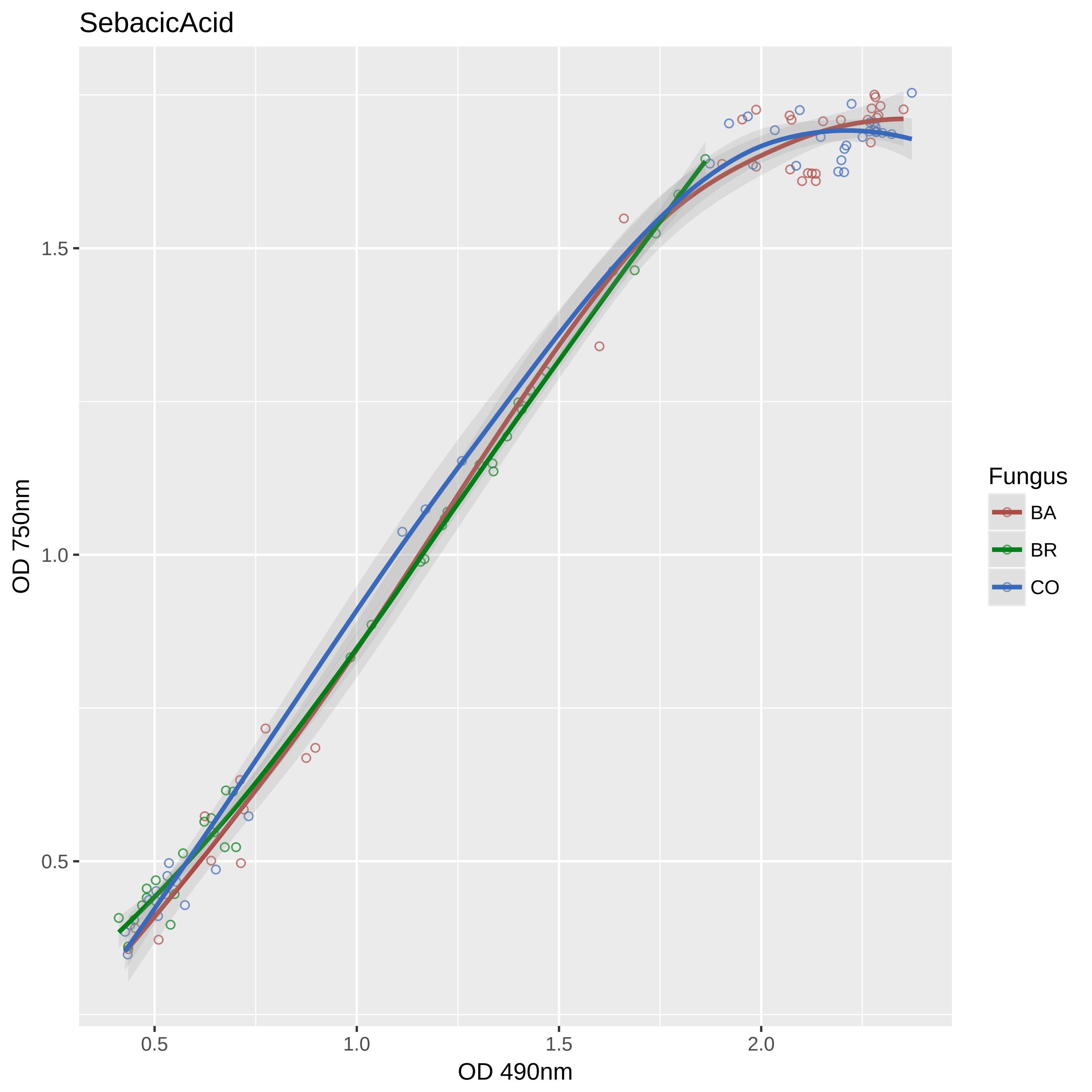

SebacicAcid
OD 750nm
1.5
1.0
0.5
0.5
1.0
1.5
2.0
OD 490nm
Fungus
BA
BR
CO

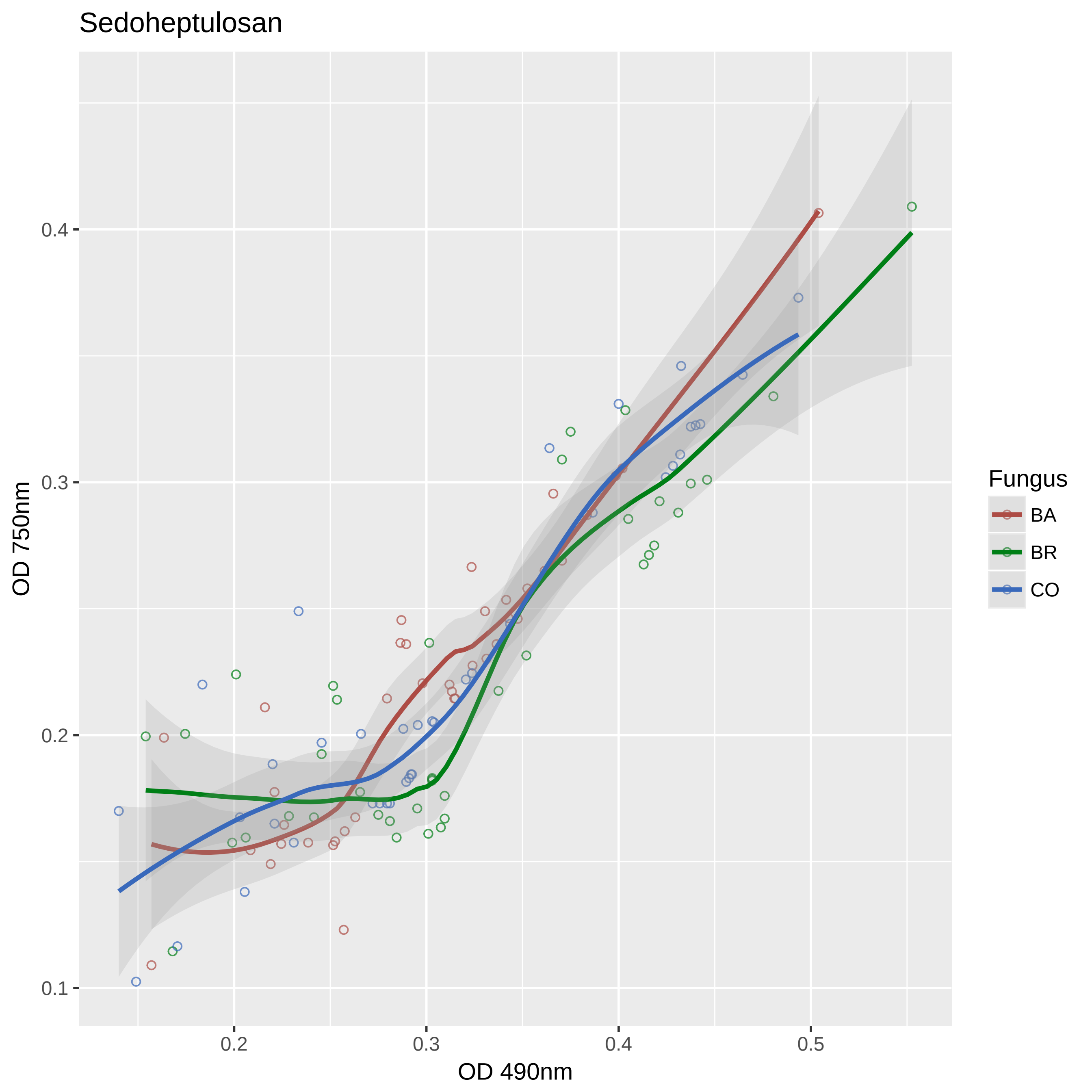

Sedoheptulosan
OD 750nm
0.1
0.2
0.3
0.4
OD 490nm
0.2
0.3
0.4
0.5
Fungus
BA
BR
CO

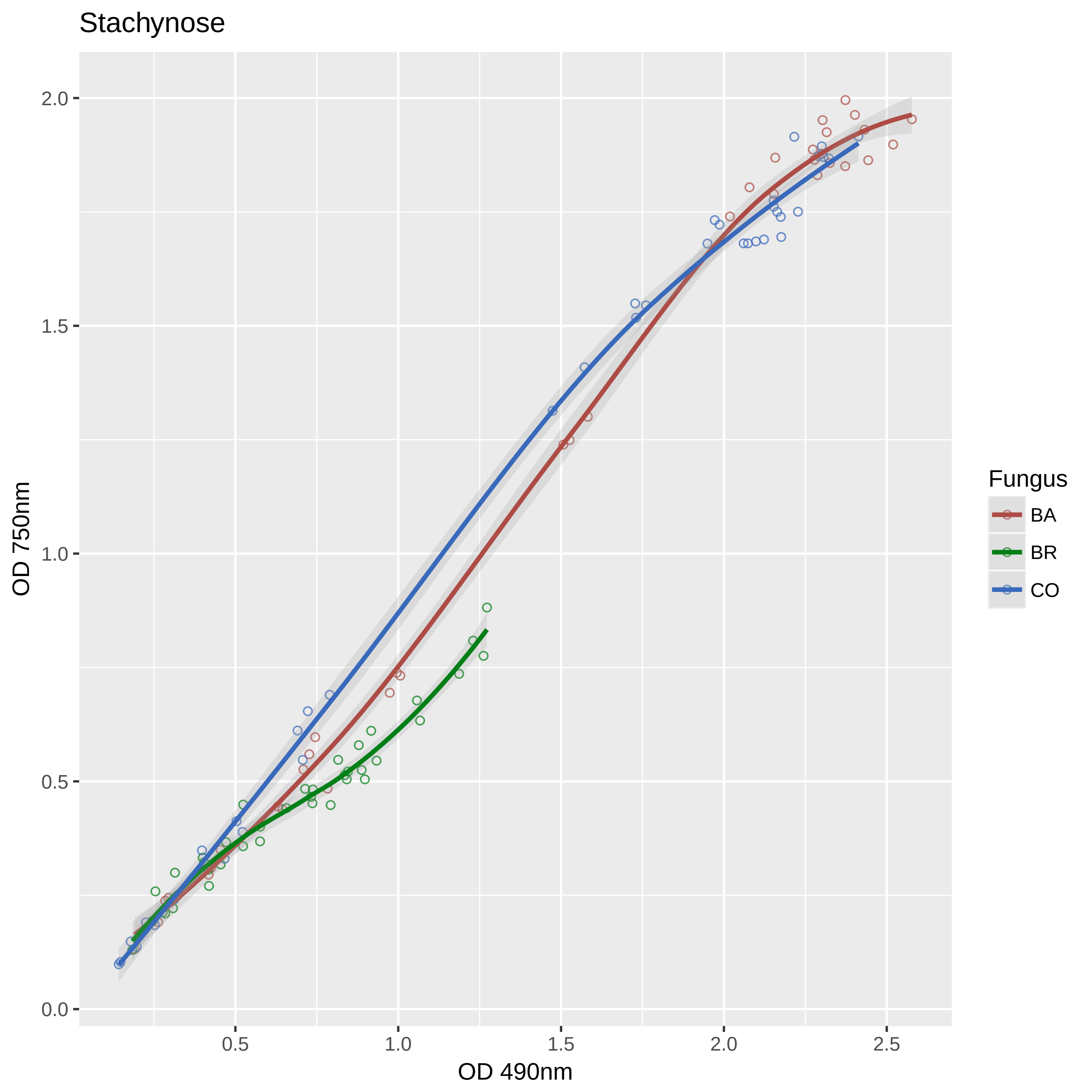

Stachynose
OD 750nm
OD 490nm
0.0
0.5
1.0
1.5
2.0
0.5
1.0
1.5
2.0
2.5
Fungus
BA
BR
CO

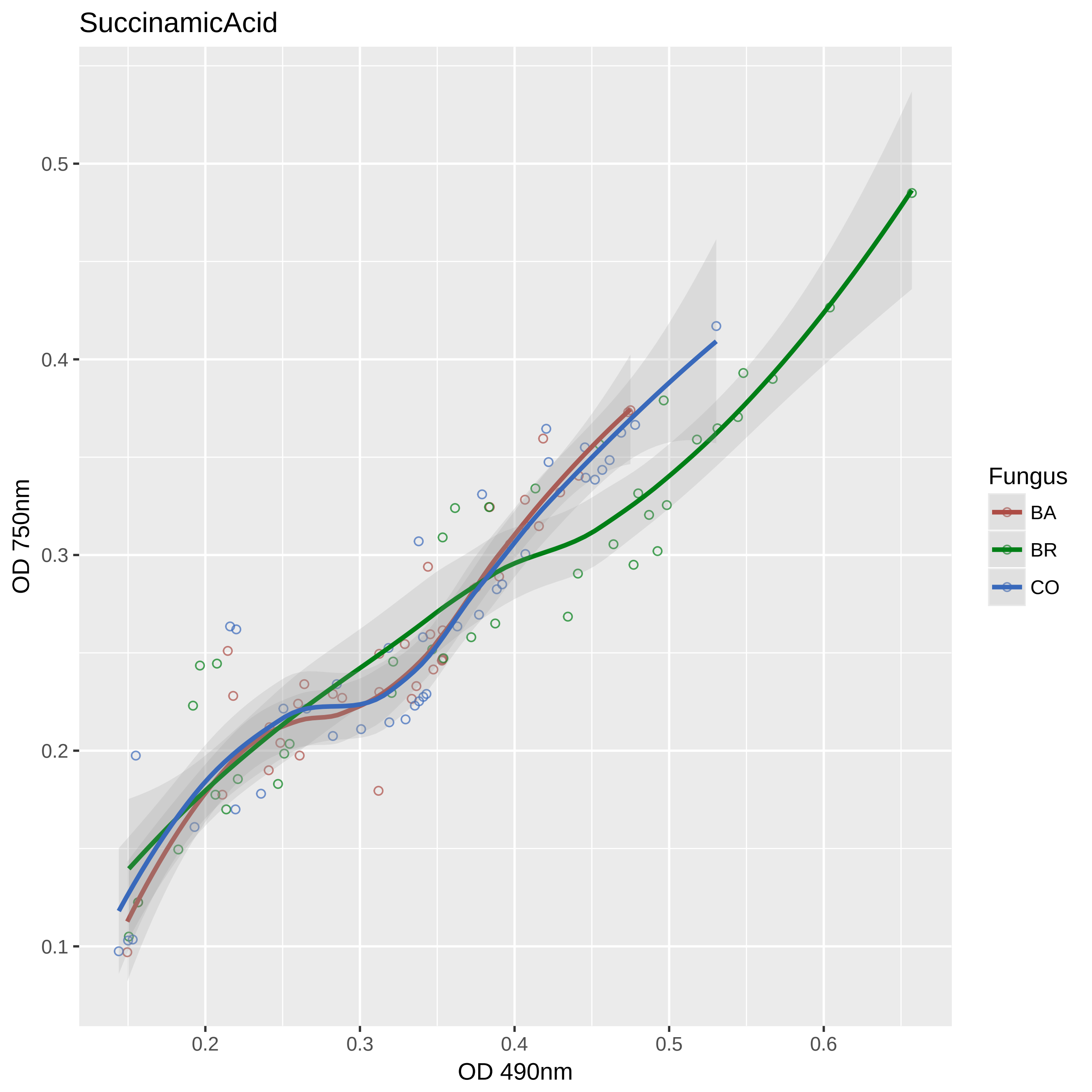

SuccinamicAcid
OD 750nm
0.1
0.2
0.3
0.4
0.5
OD 490nm
0.2
0.3
0.4
0.5
0.6
Fungus
BA
BR
CO

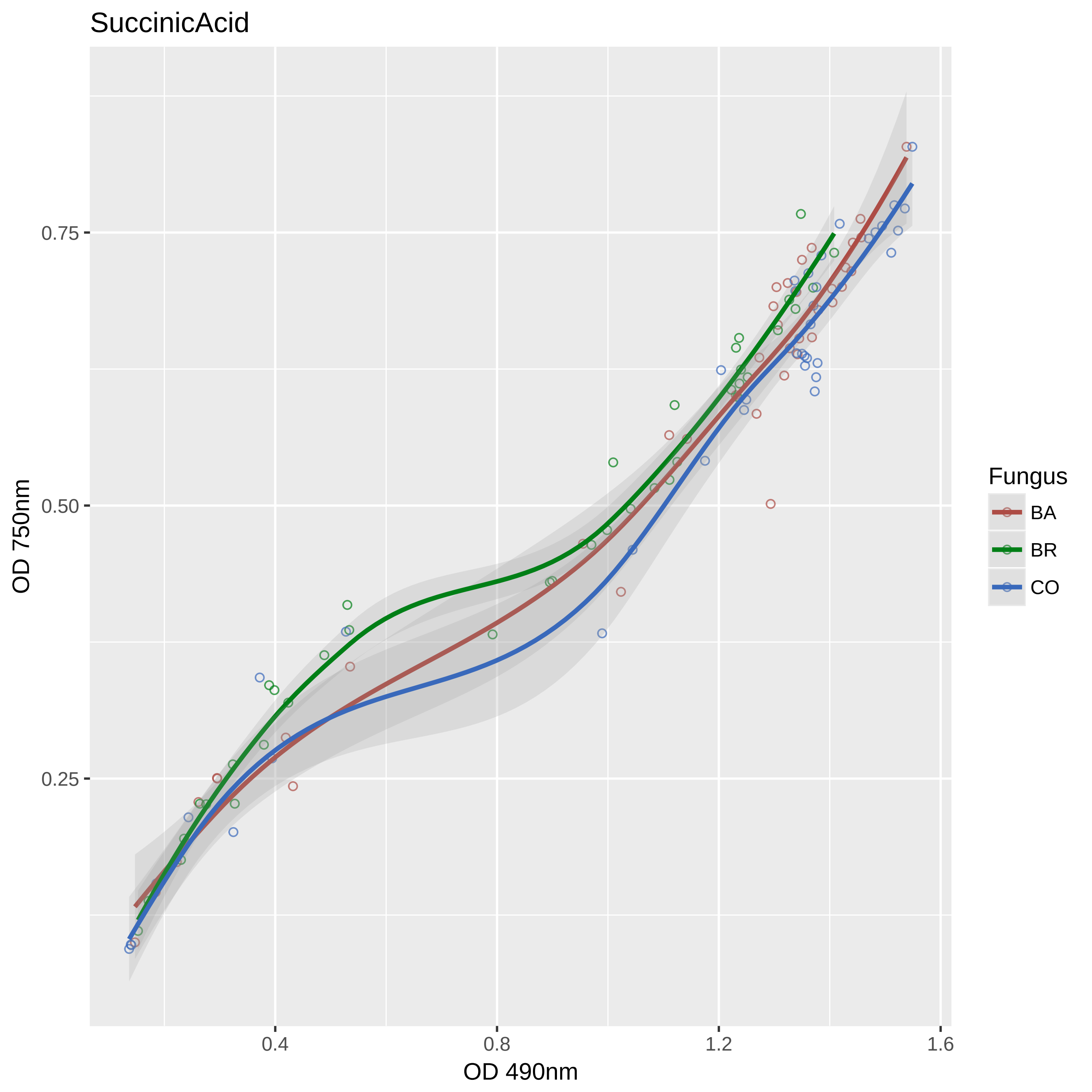

SuccinicAcid
OD 750nm
0.75
0.50
0.25
0.4
0.8
1.2
1.6
OD 490nm
Fungus
BA
BR
CO

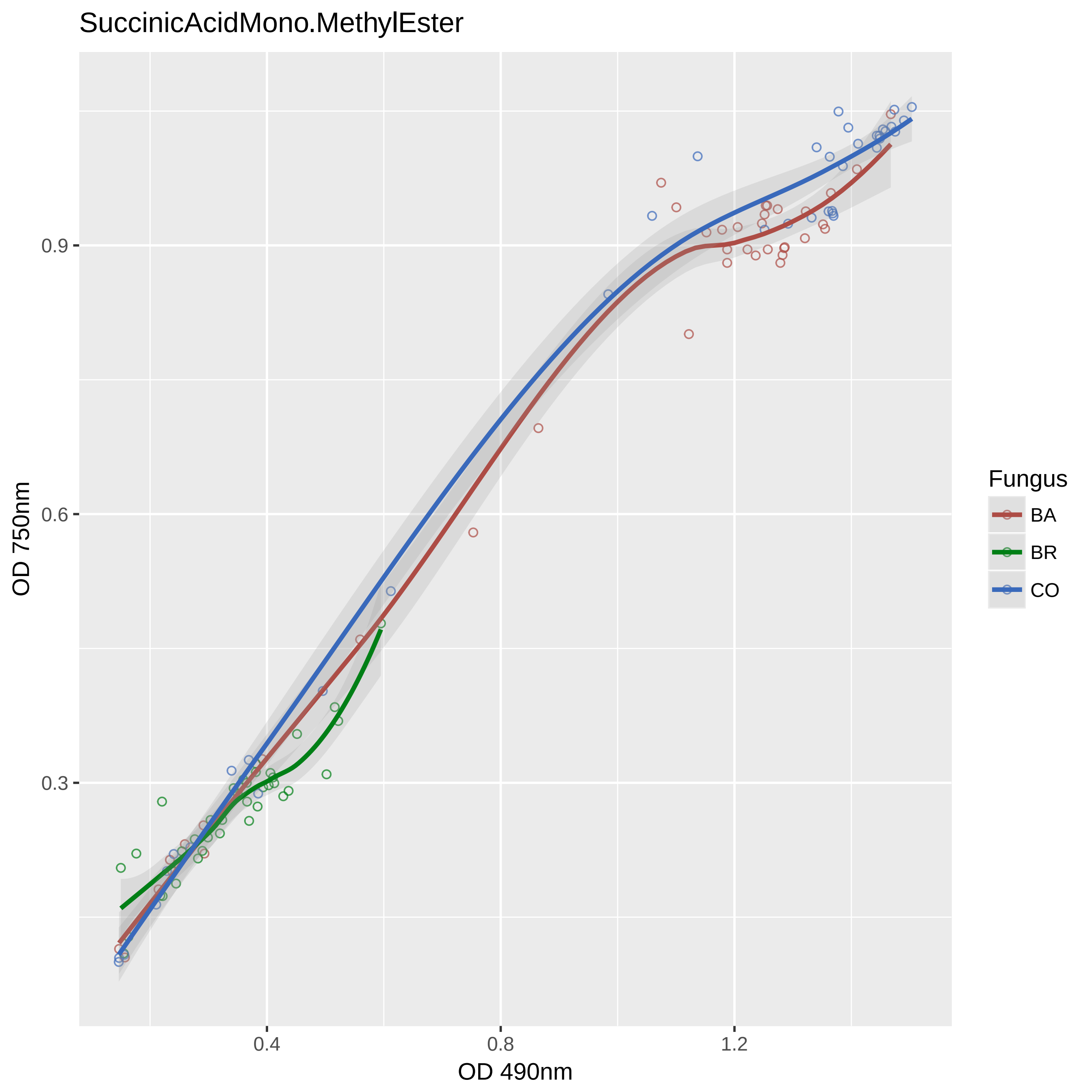

SuccinicAcidMono.MethylEster
OD 750nm
0.9
0.6
0.3
OD 490nm
0.4
0.8
1.2
Fungus
BA
BR
CO

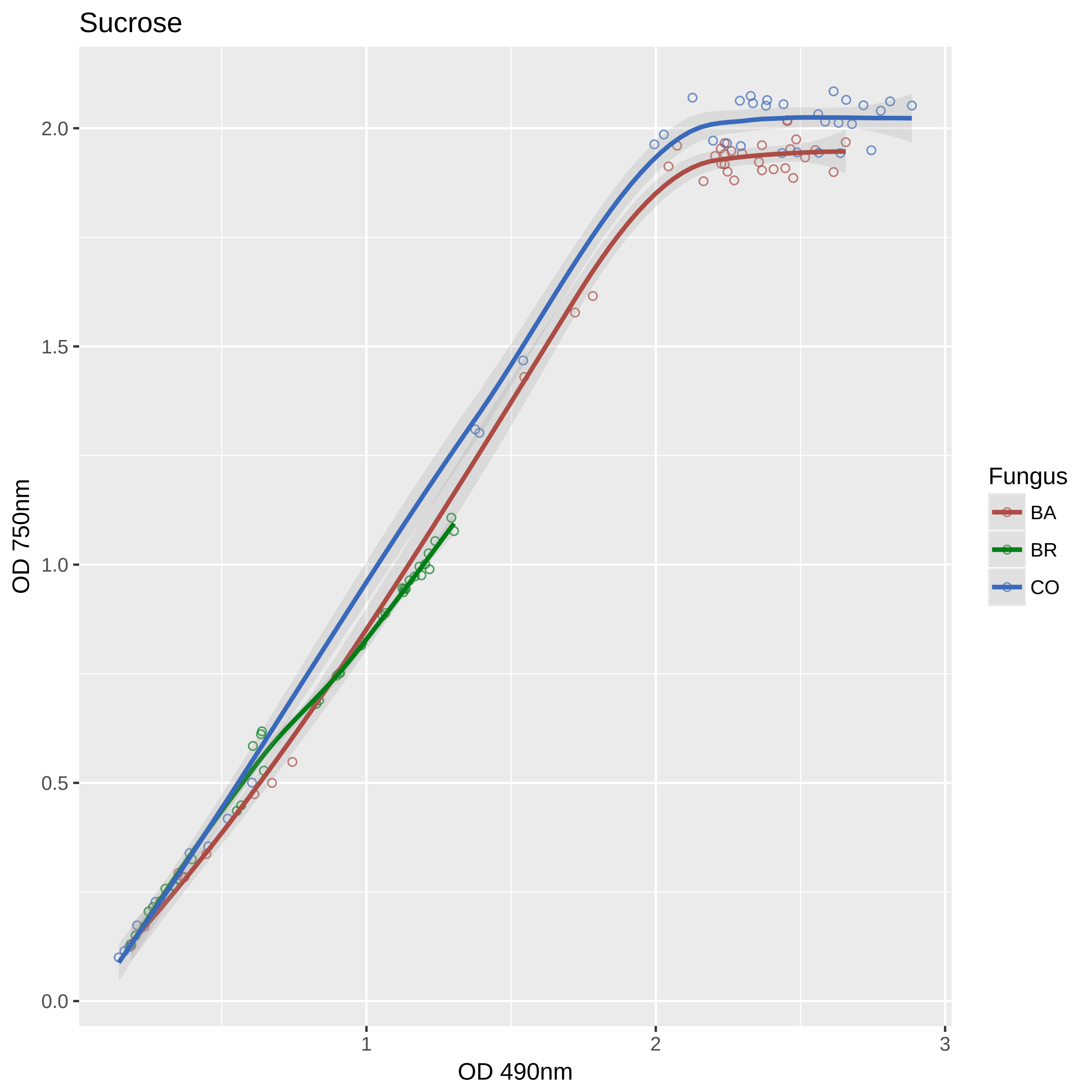

Sucrose
OD 750nm
OD 490nm
0.0
0.5
1.0
1.5
2.0
1
2
3
Fungus
BA
BR
CO

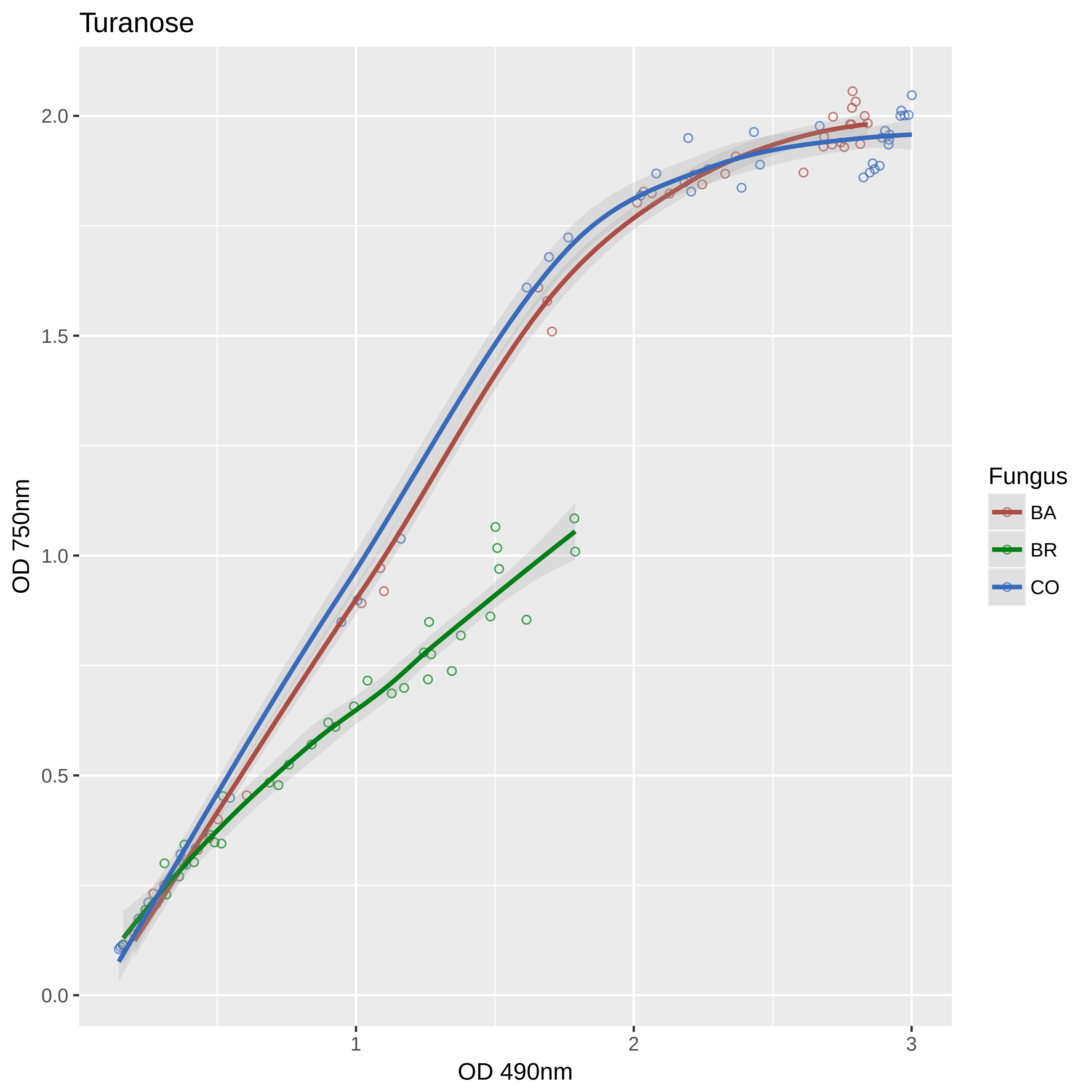

Turanose
OD 750nm
OD 490nm
0.0
0.5
1.0
1.5
2.0
1
2
3
Fungus
BA
BR
CO

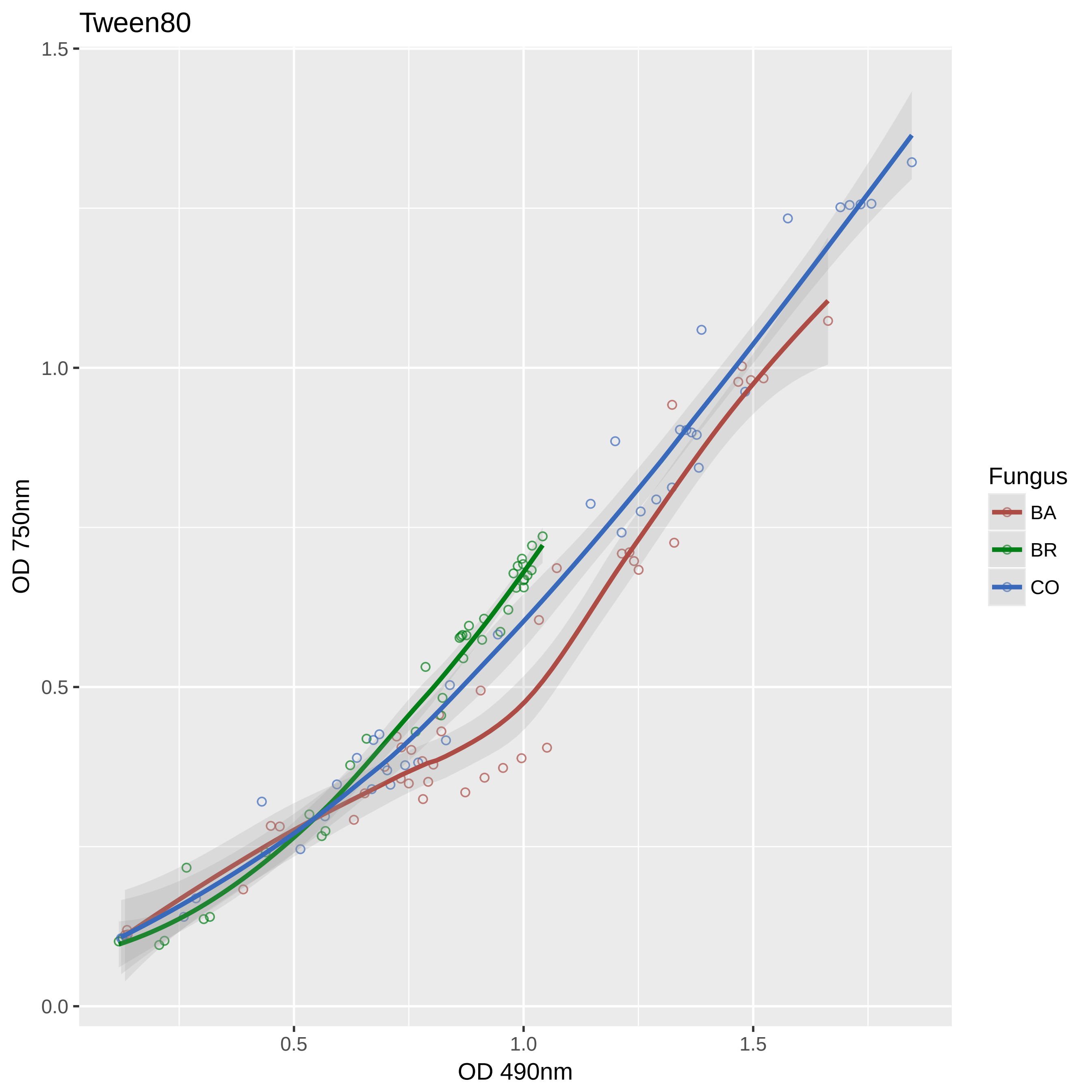

Tween80
OD 750nm
OD 490nm
0.0
0.5
1.0
1.5
0.5
1.0
1.5
Fungus
BA
BR
CO

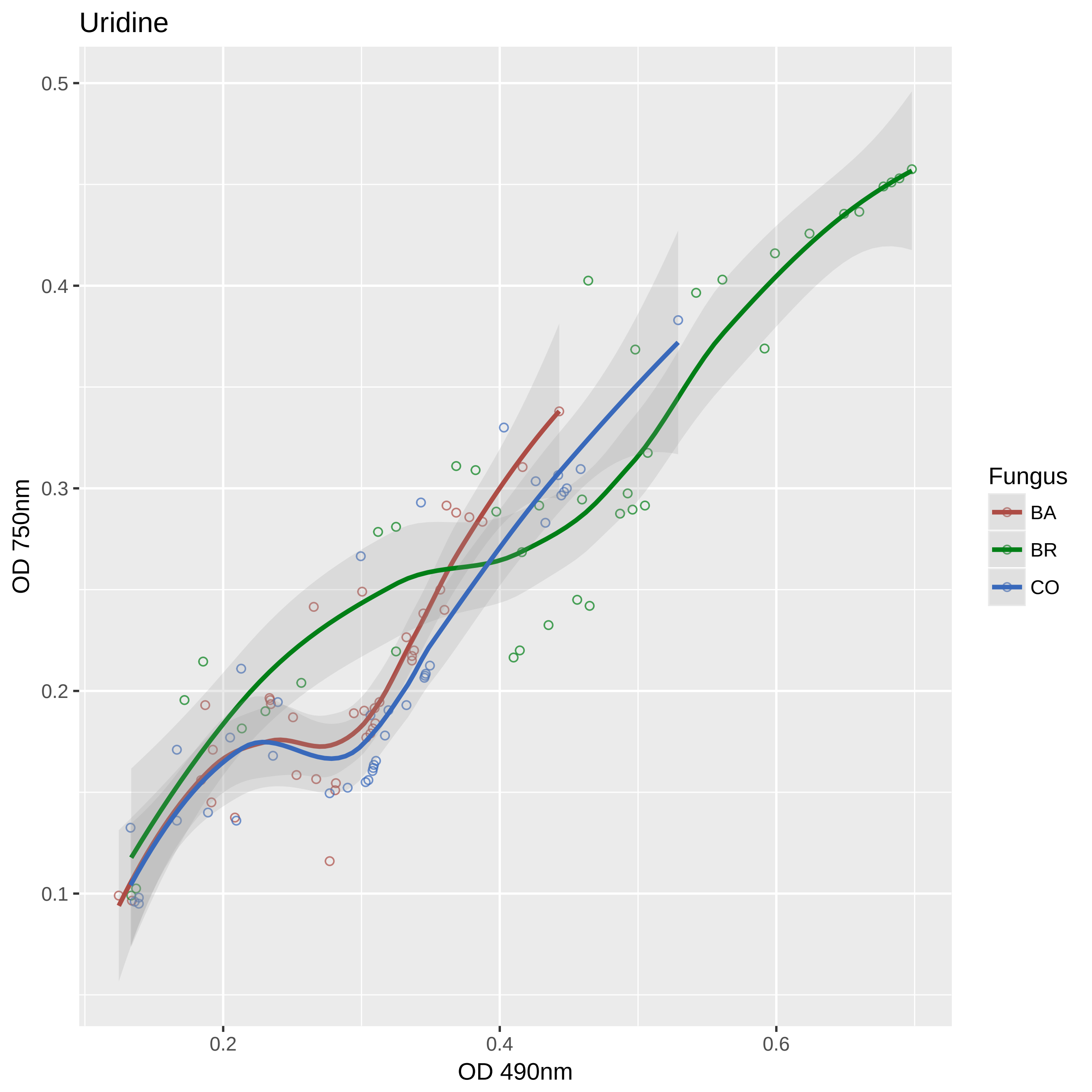

Uridine
OD 750nm
0.1
0.2
0.3
0.4
0.5
OD 490nm
0.2
0.4
0.6
Fungus
BA
BR
CO

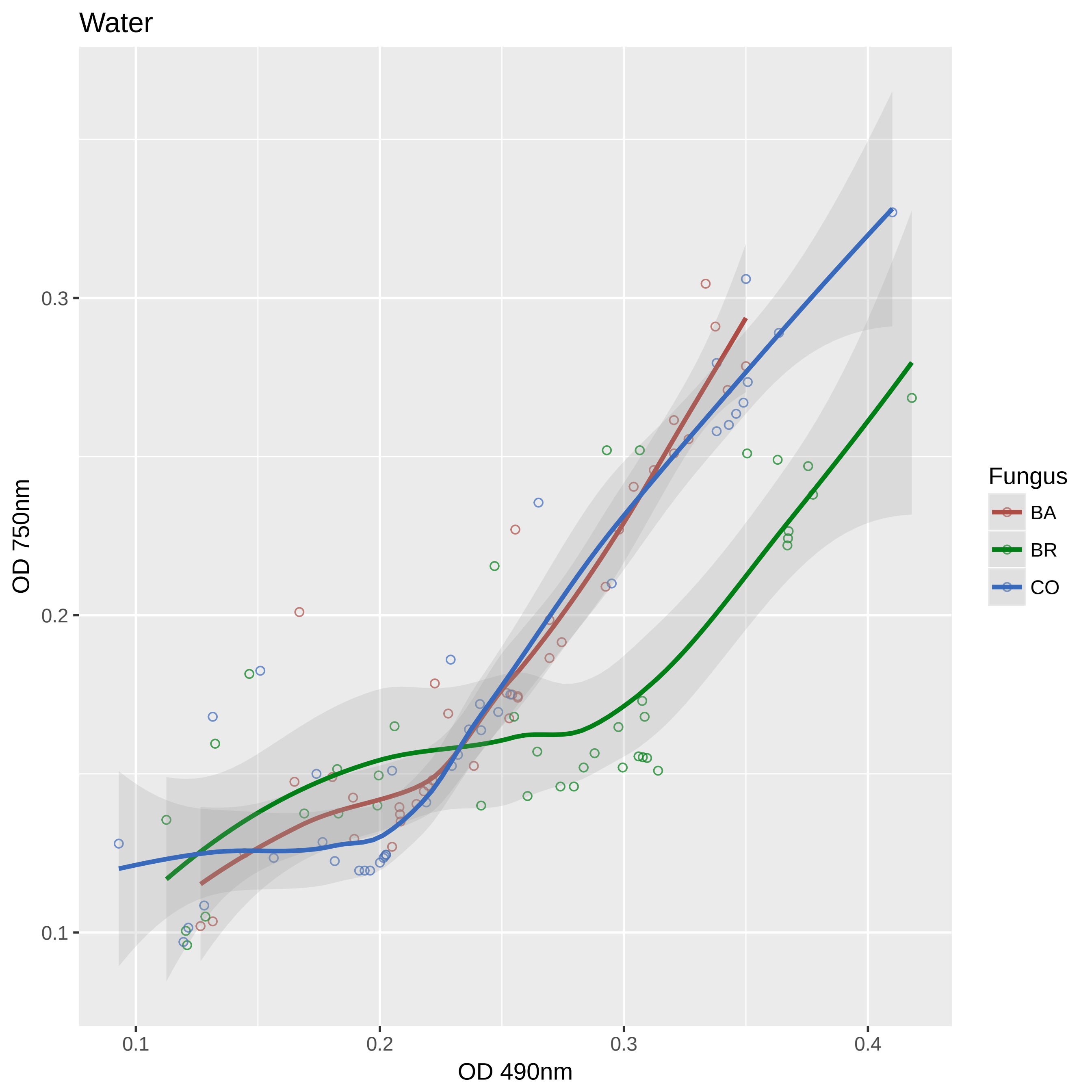

Water
OD 750nm
0.3
0.2
0.1
0.1
0.2
0.3
0.4
OD 490nm
Fungus
BA
BR
CO

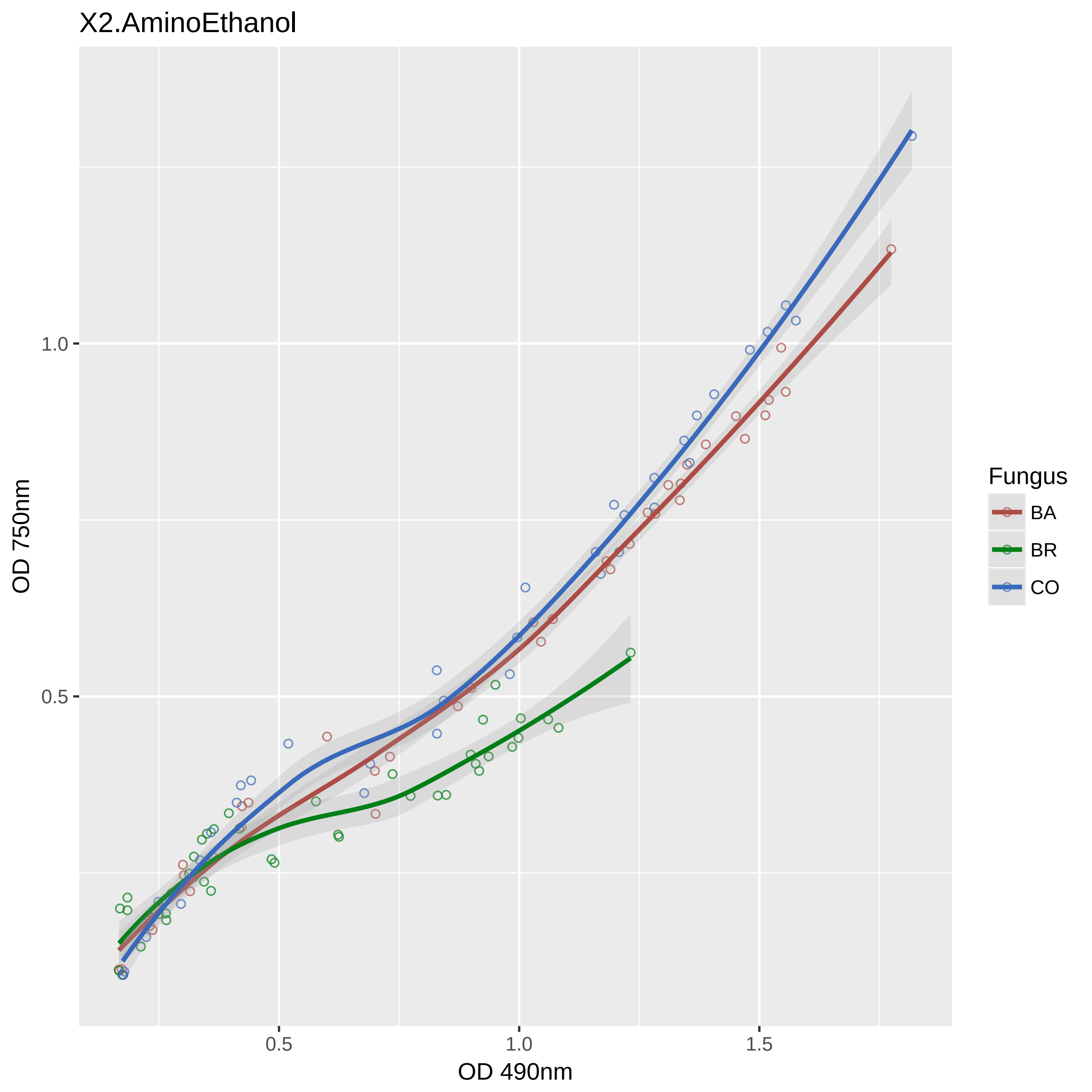
X2.AminoEthanol
OD 750nm
1.0
0.5
OD 490nm
0.5
1.0
1.5
Fungus
BA
BR
CO

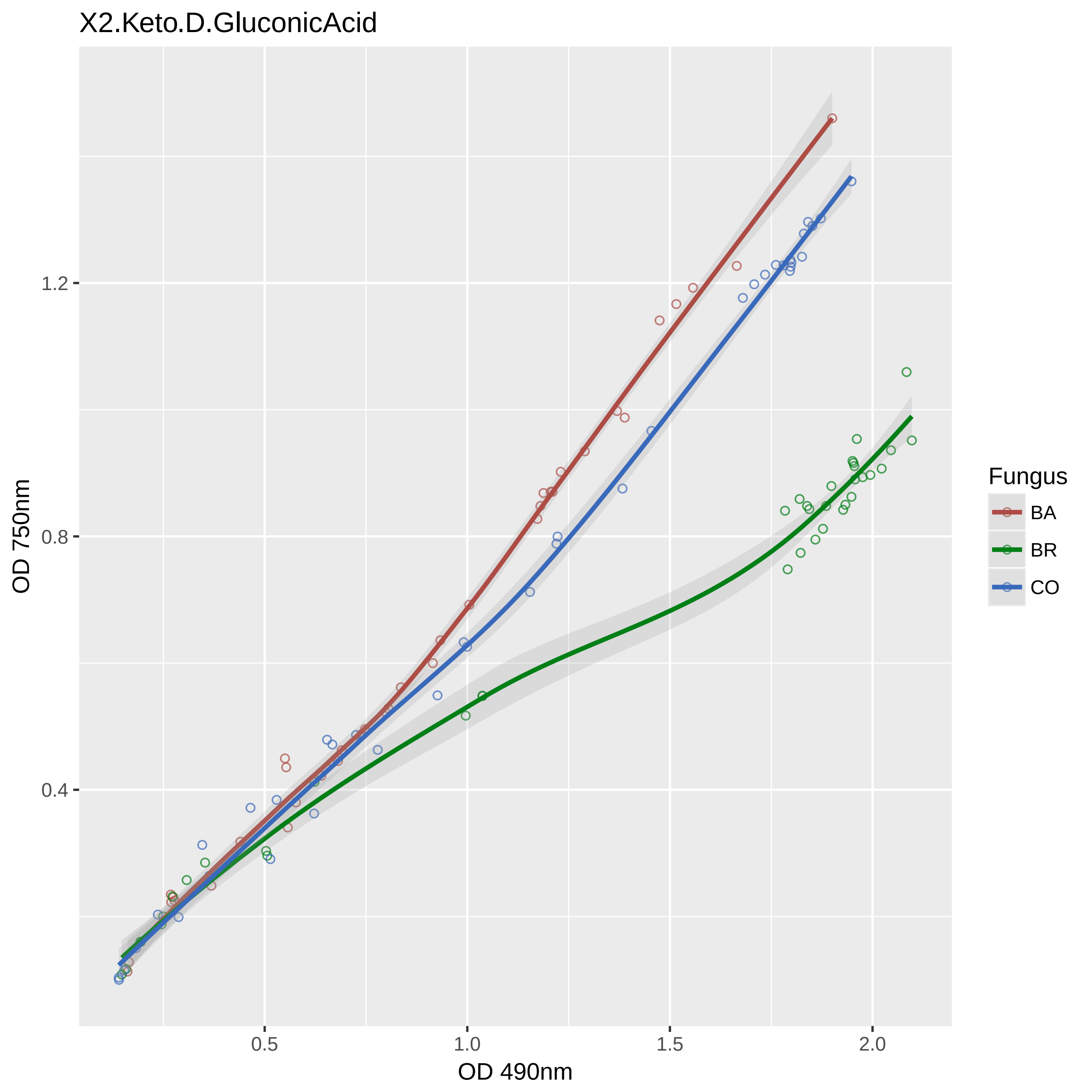
X2.Keto.D.GluconicAcid
OD 750nm
1.2
0.8
0.4
0.5
1.0
1.5
2.0
OD 490nm
Fungus
BA
BR
CO

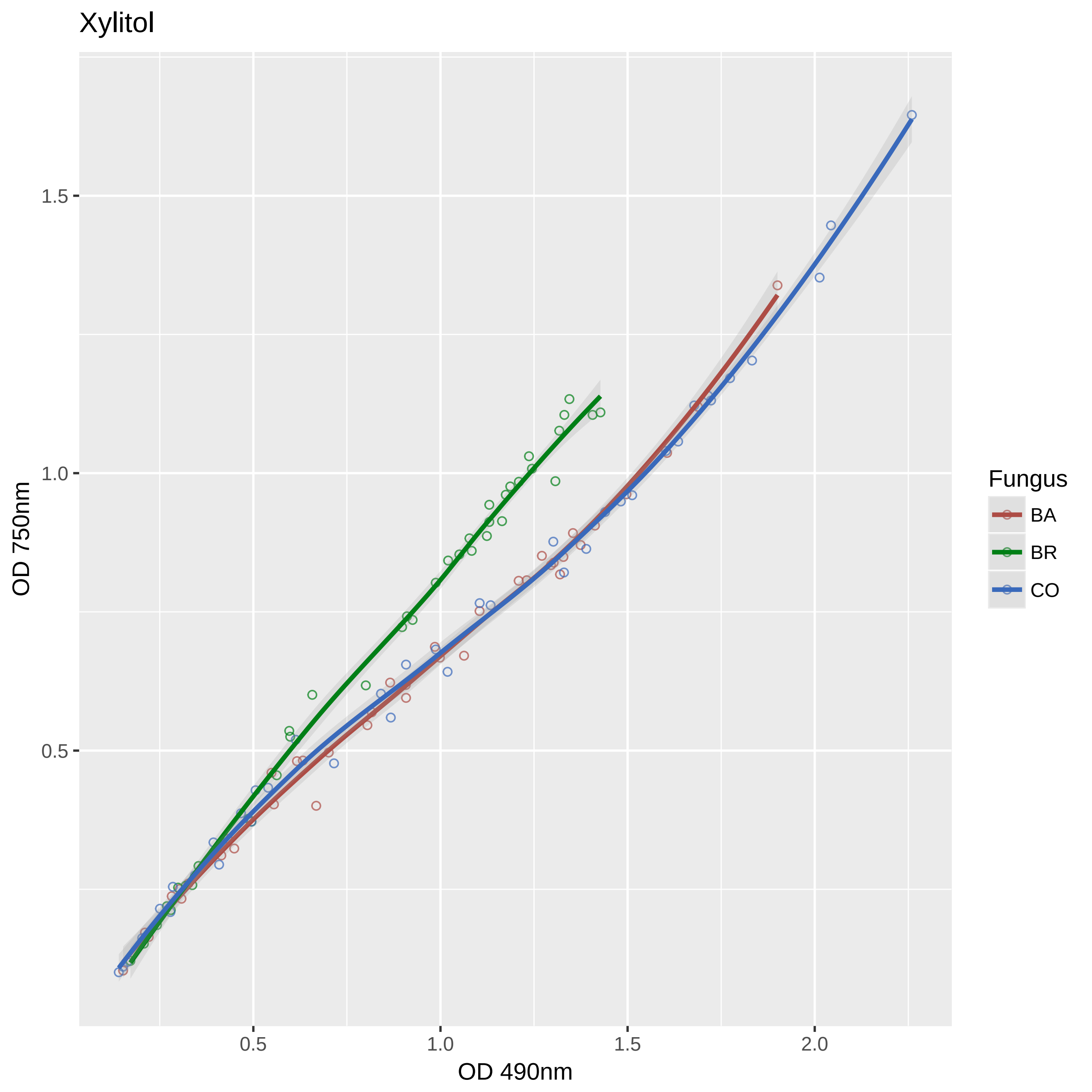

Xylitol
OD 750nm
1.5
1.0
0.5
0.5
1.0
1.5
2.0
OD 490nm
Fungus
BA
BR
CO
